# Identification of Candidate Genes in Breast Cancer Induced by Estrogen Plus Progestogens Using Bioinformatic Analysis

**DOI:** 10.3390/ijms231911892

**Published:** 2022-10-06

**Authors:** Yu Deng, He Huang, Jiangcheng Shi, Hongyan Jin

**Affiliations:** 1 Department of Obstetrics and Gynecology, Peking University First Hospital, No. 8 Xishiku Street, Beijing 100034, China; 2 School of Life Sciences, Tiangong University, Tianjin 300387, China

**Keywords:** menopausal hormone therapy, estrogen plus progestogens treatment, breast cancer, bioinformatic analysis

## Abstract

Menopausal hormone therapy (MHT) was widely used to treat menopause-related symptoms in menopausal women. However, MHT therapies were controversial with the increased risk of breast cancer because of different estrogen and progestogen combinations, and the molecular basis behind this phenomenon is currently not understood. To address this issue, we identified differentially expressed genes (DEGs) between the estrogen plus progestogens treatment (EPT) and estrogen treatment (ET) using the Gene Expression Omnibus (GEO) and The Cancer Genome Atlas (TCGA) data. As a result, a total of 96 upregulated DEGs were first identified. Seven DEGs related to the cell cycle (*CCNE2, CDCA5, RAD51, TCF19, KNTC1, MCM10*, and *NEIL3*) were validated by RT-qPCR. Specifically, these seven DEGs were increased in EPT compared to ET (*p* < 0.05) and had higher expression levels in breast cancer than adjacent normal tissues (*p* < 0.05). Next, we found that estrogen receptor (ER)-positive breast cancer patients with a higher *CNNE2* expression have a shorter overall survival time (*p* < 0.05), while this effect was not observed in the other six DEGs (*p* > 0.05). Interestingly, the molecular docking results showed that CCNE2 might bind to 17β-estradiol (−6.791 kcal/mol), progesterone (−6.847 kcal/mol), and medroxyprogesterone acetate (−6.314 kcal/mol) with a relatively strong binding affinity, respectively. Importantly, CNNE2 protein level could be upregulated with EPT and attenuated by estrogen receptor antagonist, acolbifene and had interactions with cancer driver genes (*AKT1* and *KRAS*) and high mutation frequency gene (*TP53* and *PTEN*) in breast cancer patients. In conclusion, the current study showed that *CCNE2, CDCA5, RAD51, TCF19, KNTC1, MCM10,* and *NEIL3* might contribute to EPT-related tumorigenesis in breast cancer, with *CCNE2* might be a sensitive risk indicator of breast cancer risk in women using MHT.

## 1. Introduction

As the global population ages and life expectancy increases, it is estimated that more than 1.6 billion women worldwide will reach menopause by the year 2050 [1]. Globally, menopausal hormone therapy (MHT) was the most common method to alleviate menopausal symptoms [2,3,4]. In detail, MHT was characterized by using different ingredients, formulations, and administration routes, relieving menopausal symptoms such as hot flashes, sweating, anxiety, and depression associated with menopause [5]. The commonly used MHT therapies were classified into estrogen treatment (ET) and estrogen plus progestogens treatment (EPT) [6,7]. The differences in breast cancer risk caused by estrogen combinations with different progestogens confused people using MHT [8,9,10,11], leading to a growing number of women avoiding MHT [12,13].

Four landmark studies [14,15,16,17] have been conducted regarding ET, EPT, and breast cancer risk. For example, 27347 postmenopausal women were enrolled in the Women’s Health Initiative (WHI)’s randomized clinical trials of hormone therapy, which tested estrogen plus medroxyprogesterone acetate (MPA, a synthetic progestogen) against a placebo in 16,608 women who had an intact uterus at the start of the study and estrogen alone versus placebo in 10,739 women who had a hysterectomy [14,15]. The findings indicated that women using EPT had a significantly increased risk of breast cancer (hazards ratio (HR): 1.26, 95% confidence interval (CI): 1.00–1.59) [14], while women using ET did not increase breast cancer risk (HR: 0.77, 95% CI: 0.59–1.01) [15]. This trial indicated that EPT was associated with an increased risk of breast cancer compared to ET. The latest published WHI analysis [16] reported long-term data from the WHI trial with a mean follow-up of 20.3 years. The follow-up results showed EPT was associated with the statistically significantly increased breast cancer incidence (HR: 1.28; 95% CI: 1.13–1.45) among the 16,608 women, while ET was related to a statistically significantly lower breast cancer incidence (HR: 0.78; 95% CI: 0.65–0.93) among the 10,739 women. These results were in concordance with previous WHI studies. Recently, two studies [1,18] reported that ET and EPT significantly increased the risk of breast cancer. However, these results are based on observational studies, so these results must be interpreted with caution [17]. In another study, the E3N cohort study [18] investigated 80,377 postmenopausal women with a mean follow-up time of 8.1 years, and the results showed that estrogen plus progesterone (P4, a natural progestogen) (relative risk (RR):1.00, 95% CI: 0.83–1.22) had a lower risk of breast cancer than estrogen plus synthetic progestogen (RR:1.69, 95% CI: 1.50–1.91). This large clinical study showed an increased risk of breast cancer among postmenopausal women treated with estrogen plus synthetic progesterone compared to estrogen plus natural progesterone. It was of great clinical value to clarify the molecular mechanism correlated to breast cancer risk using estrogen plus different progestogens.

Although it had been reported that MPA and P4 alone could differentially regulate cell proliferation-related genes such as *AQP3, SGK1, ALCAM, FXYD3/MAT-8, MUCIN, and c-MYB* [19], there was a lack of studies based on omics data, and genes regulated by a combination of estrogen and MPA or P4 were still unclear and needed further investigation. Under these conditions, we aimed to explore the effects of estrogen plus different progestogens on gene expression profiles of breast cancer cells, with a specific focus on genes related to cell proliferation.

This study screened out 96 upregulated differentially expressed genes (DEGs) using the Gene Expression Omnibus (GEO) and the Cancer Genome Atlas (TCGA) database data between EPT and ET. We revealed that seven genes (*cyclin E2* (*CCNE2*)*, cell division cycle-associated 5* (*CDCA5*)*, RAD51 recombinase* (*RAD51*)*, transcription factor 19* (*TCF19*)*, kinetochore-associated 1* (*KNTC1*)*, minichromosome maintenance 10 replication initiation factor* (*MCM10*)*,* and *nei-like DNA glycosylase 3* (*NEIL3*)) were related to cell cycle regulation, and positively associated with tumorigenesis and progression in breast cancer. Noteworthy, molecular docking results suggested that CCNE2 might have a relatively high affinity for the binding of E2 (−6.791 kcal/mol), P4 (−6.847 kcal/mol), and MPA (−6.314 kcal/mol), respectively. We also found that *CCNE2* expression was upregulated by EPT and attenuated by acolbifene, an estrogen receptor antagonist, in breast cancer cells, and the higher expression of *CCNE2* correlated with the progress and poor overall survival in breast cancer patients. Furthermore, we observed that *CCNE2* was closely associated with cancer driver genes (*AKT serine/threonine kinase 1* (*AKT1*) and *KRAS proto-oncogene, GTPase* (*KRAS*)) and high mutation frequency gene (*tumor protein p53* (*TP53*) and *phosphatase and tensin homolog* (*PTEN*)) in breast cancer patients. These data indicated that C*CNE2* might play a more important role than the other six DEGs in the increased breast cancer risk associated with EPT. In summary, these results would provide new insights into our deep understanding of the molecular mechanism involved in EPT regulation and the increased risk of breast cancer with treatments of EPT compared with ET.

## 2. Results

### 2.1. Identification of DEGs in P4, MPA, and BRCA Group

**Figure 1:**
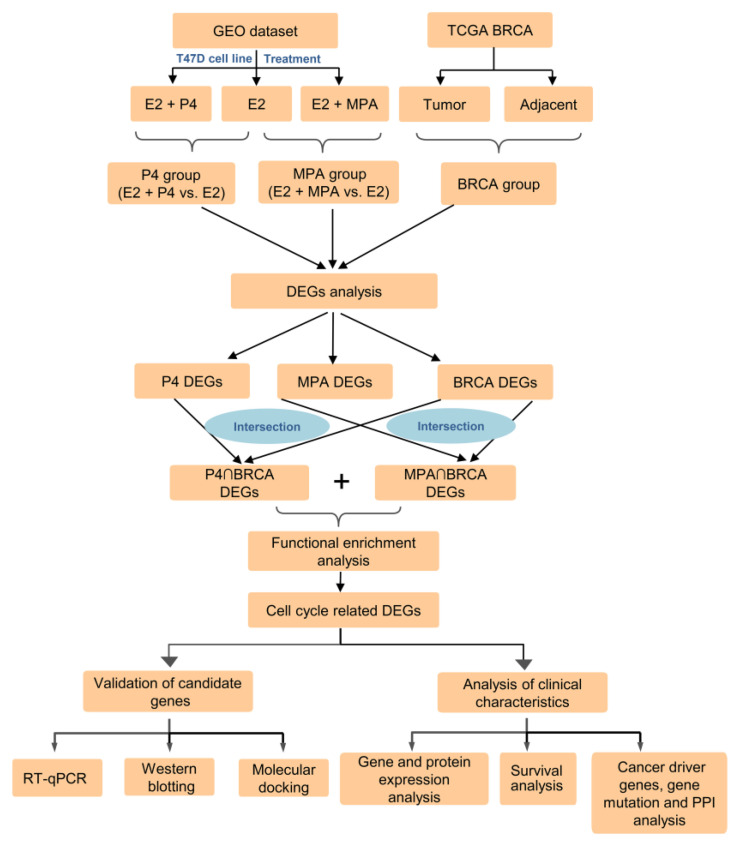
Schematic diagram of the methodology used in the study. GEO, the Gene Expression Omnibus; TCGA-BRCA, The Cancer Genome Atlas Breast Invasive Carcinoma database; E2, 17β-estradiol; P4, progesterone; MPA, medroxyprogesterone acetate; DEGs, differently expressed genes; RT-qPCR, reverse transcription, and quantitative real-time polymerase chain reaction.

**Figure 2:**
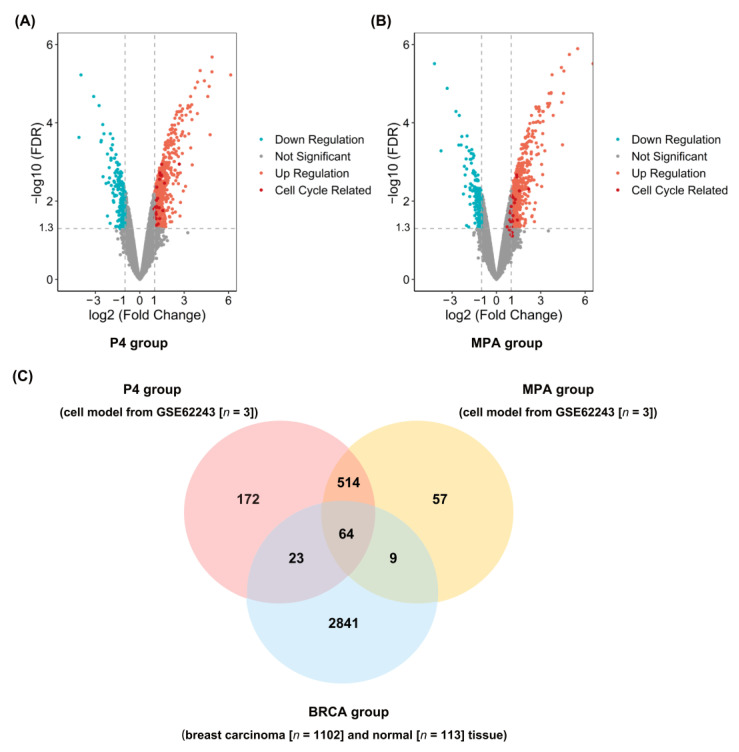
Identify differentially expressed genes (DEGs) among the P4, MPA, and BRCA groups. (**A**) Volcano plots of DEGs of the P4 group (E2 + P4 vs. E2). Upregulated genes are marked in red; downregulated genes are marked in blue. Cell cycle-related DEGs are marked in dark red. (**B**) Volcano plots of DEGs of the MPA group (E2 + MPA vs. E2). Upregulated genes are marked in red; downregulated genes are marked in blue. Cell cycle-related DEGs are marked in dark red. (**C**) Venn diagram of upregulated DEGs among the P4 group, MPA group, and BRCA group, and a total of 96 upregulated DEGs are screened out at the intersection of the three datasets. E2, 17β-estradiol; P4, progesterone; MPA, medroxyprogesterone acetate. BRCA group, gene expression profiles of invasive breast carcinoma downloaded from The Cancer Genome Atlas (TCGA-BRCA). The conditions for DEGs screening were absolute value of log (2) fold-change (|log_2_Fold Change|) > 1.0 and false discovery rate (FDR) < 0.05.

The overall schematic of methods used in this study is shown in Figure 1. DEGs of the P4 and MPA groups were identified from GSE62243 expression profiles. Gene expression profiles of breast cancer cells in response to each progestin versus control revealed that different numbers of genes were expressed in response to P4 and MPA (Supplementary Table S1). The addition of E2 and progestins simultaneously resulted in higher numbers of differentially expressed genes compared to progestins. Of the 552 DEGs in breast cancer cells treated with P4 versus control, 458 DEGs (82.97%) were also differentially expressed in E2 + P4 versus control. Of the 1056 DEGs expressed in breast cancer cells treated with MPA, 898 DEGs (85.03%) were also expressed in the E2+MPA treatment group. We identified 773 overexpression genes in the P4 group (Figure 2A) and 644 in the MPA group (Figure 2B) (|log2 Fold Change (FC)| ≥ 1 and false discovery rate (FDR) < 0.05), which indicated differences in gene expression profiles between ET and EPT treatment. The DEGs among the three groups (P4, MPA, and BRCA groups) were listed in Supplementary Tables S2–S5. To validate DEGs’ reliability, we adopted the Venn diagram to obtain overlapped DEGs between the P4, MPA, and BRCA groups. A total of 96 upregulated DEGs were screened at the three data sets’ intersections (Figure 2C).

### 2.2. Functional Enrichment Analysis and qPCR Validation of DEGs

**Figure 3:**
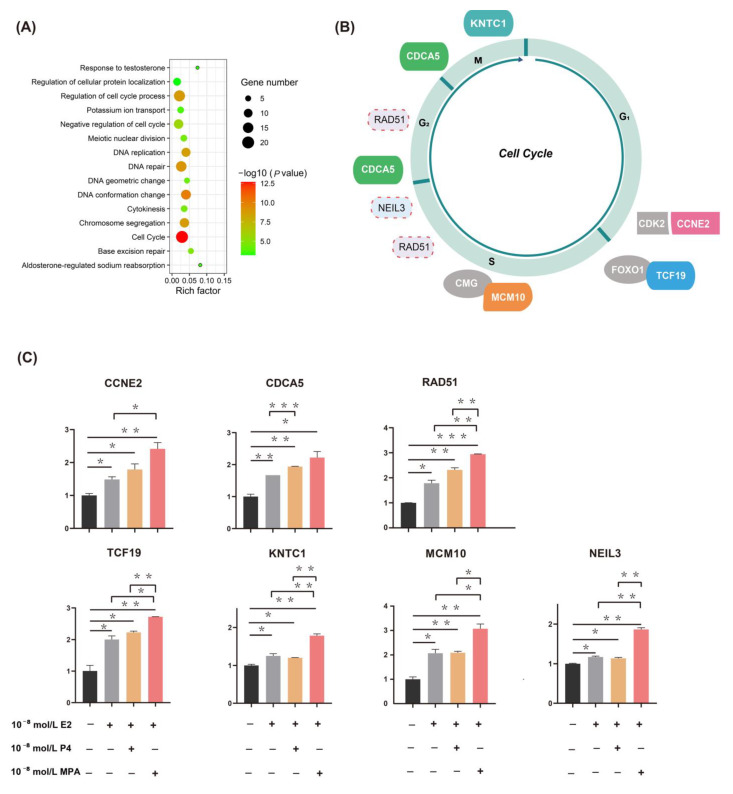
Seven upregulated candidate genes are related to cell cycle and genome stability. (**A**) Top 15 enriched biological functions of intersected differentially expressed genes (DEGs) in which P4 group and MPA group intersect with BRCA group. The color of each point represents the value of -log10 (*p*-value), and the size of each point corresponds to the number of DEGs represented in the functional term. The rich factor is the percentage of all the DEGs found in the given ontology term. Altogether, most of the 96 upregulated DEGs are associated with cell cycle and genome stability. (**B**) A simple model of cell cycle regulation. The cell cycle is divided into four main stages: the gap 1 phase (G1), synthesis phase (S), gap 2 phase (G2), and mitotic phase (M). Seven genes (*CCNE2, CDCA5, RAD51, TCF19, KNTC1, MCM10,* and *NEIL3*) regulated by EPT are indicated with colored boxes and placed near the corresponding cell cycle phases. The gray boxes indicate the proteins they bind. The solid line box represents the effect of genes on the cell cycle has been well documented, and the dotted line box represents the effect of genes on the cell cycle documented in the few studies. (**C**) RT-qPCR validation of candidate genes expression (*CCNE2, CDCA5, RAD51, TCF19, KNTC1, MCM10,* and *NEIL3*) in human breast cancer cells. The mRNA expression of all seven genes increased evidently in EPT compared to the ET (*p* < 0.05). * *p* < 0.05, ** *p* < 0.01, and *** *p* < 0.001. E2, 17β-estradiol; P4, progesterone; MPA, medroxyprogesterone acetate. EPT, estrogen plus progestogens treatment; ET, estrogen treatment.

We performed functional enrichment analysis on 96 upregulated DEGs among the P4 group, MPA group, and BRCA group to detect DEGs’ potential biological functions further. These genes are significantly enriched in functional items, including cell cycle, cell cycle regulation, negative regulation of cell cycle, DNA repair, DNA conformation change, etc. (Figure 3A and Supplementary Table S6). We found seven genes (CCNE2, CDCA5, RAD51, TCF19, KNTC1, MCM10, and NEIL3) related to the cell cycle (Figure 3B). These results suggested that EPT treatments might regulate cell cycle-related genes more intensely than ET.

To further confirm the GEO and BRCA group results, we identified the expression of seven candidate genes (CCNE2, CDCA5, RAD51, TCF19, KNTC1, MCM10, and NEIL3) in T47D cells (Figure 3C) using RT-qPCR. As shown in Figure 3C, compared with the control group, both ET and EPT could increase the expression of seven candidate genes, which was significantly stronger in EPT compared to that of the ET; interestingly, a significantly higher expression of RAD51, TCF19, KNTC1, MCM10, and NEIL3 was noted in E2 plus MPA compared to E2 plus P4 (*p* < 0.05).

### 2.3. Upregulation of Candidate Genes Associated with Mammary Tumorigenesis and Progression

**Figure 4:**
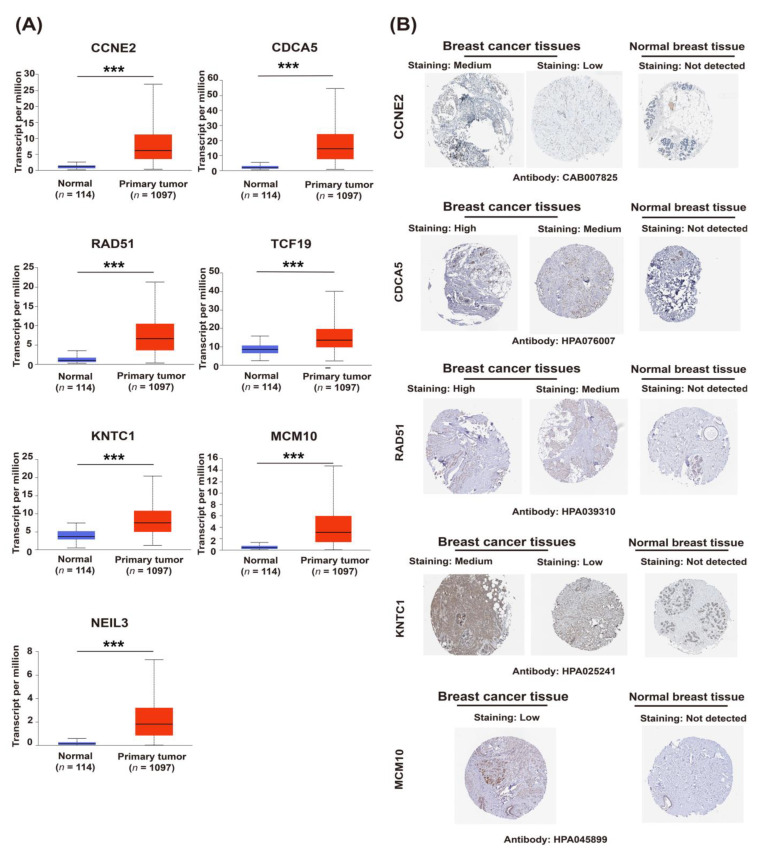
The upregulation of candidate genes (*CCNE2, CDCA5, RAD51, TCF19, KNTC1, MCM10,* and *NEIL3*) is associated with breast tumorigenesis and malignant progression. (**A**) The gene expression of candidate genes in breast cancer tissues and corresponding adjacent breast tissues, based on the The Cancer Genome Atlas (TCGA) database and visualized by the UALCAN portal. *p* < 0.05, *p* < 0.01, and *** *p* < 0.001. (**B**) Immunohistochemical results from the Human Protein Atlas (HPA) database. The protein expression of CCNE2, CDCA5, RAD51, KNTC1, and MCM10 in breast cancer tissues and normal breast tissue.

**Figure 5:**
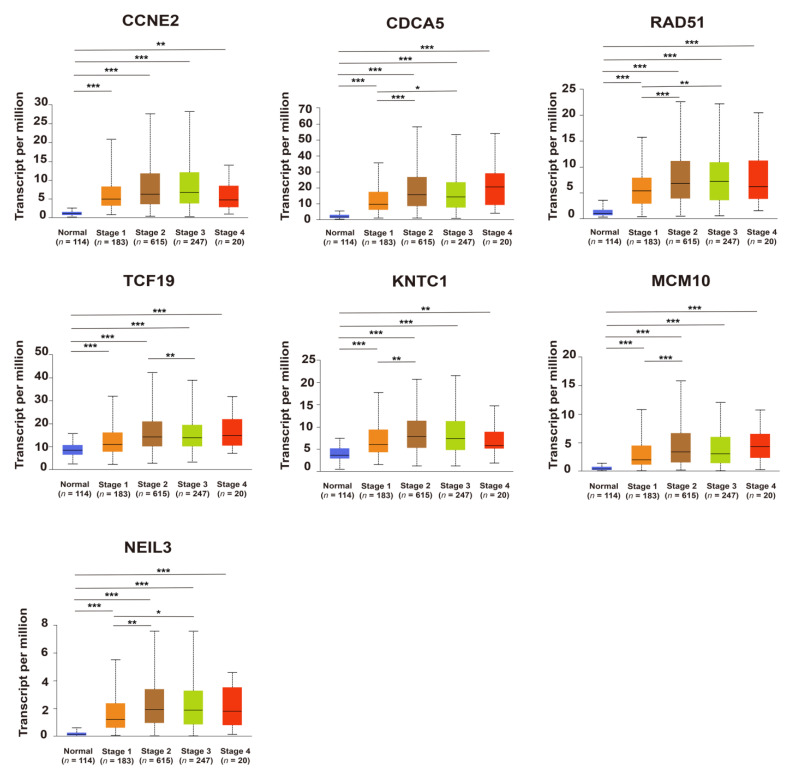
The expression of candidate genes (*CCNE2, CDCA5, RAD51,* *TCF19, KNTC1,* *MCM10,* and *NEIL3*) in different breast cancer stages according to the UALCAN database. * *p* < 0.05, ** *p* < 0.01, and *** *p* < 0.001.

Comparing cancer samples with matched normal tissues was a common strategy to identify genes associated with mammary tumorigenesis and progression. Seven candidate genes expressed significantly higher in breast cancer compared to its adjacent breast tissues, based on the TCGA database (*p* < 0.05) (Figure 4A), this is consistent with the immunohistochemical results from the Human Protein Atlas database (HPA) database, indicating that the expression of CCNE2, CDCA5, RAD51, KNTC1, and MCM10 in breast cancer tissues was higher than in normal breast tissues (Figure 4B). The expression of candidate genes was further analyzed according to the TNM staging. As shown in Figure 5, the relative expression of candidate genes displayed an increasing trend as cancer was at a more advanced stage, which indicated that these genes might be positively associated with tumor progression.

### 2.4. CCNE2 Expression Was Positively Correlated with Poor Outcomes in ER-Positive Breast Cancer Patients

**Figure 6:**
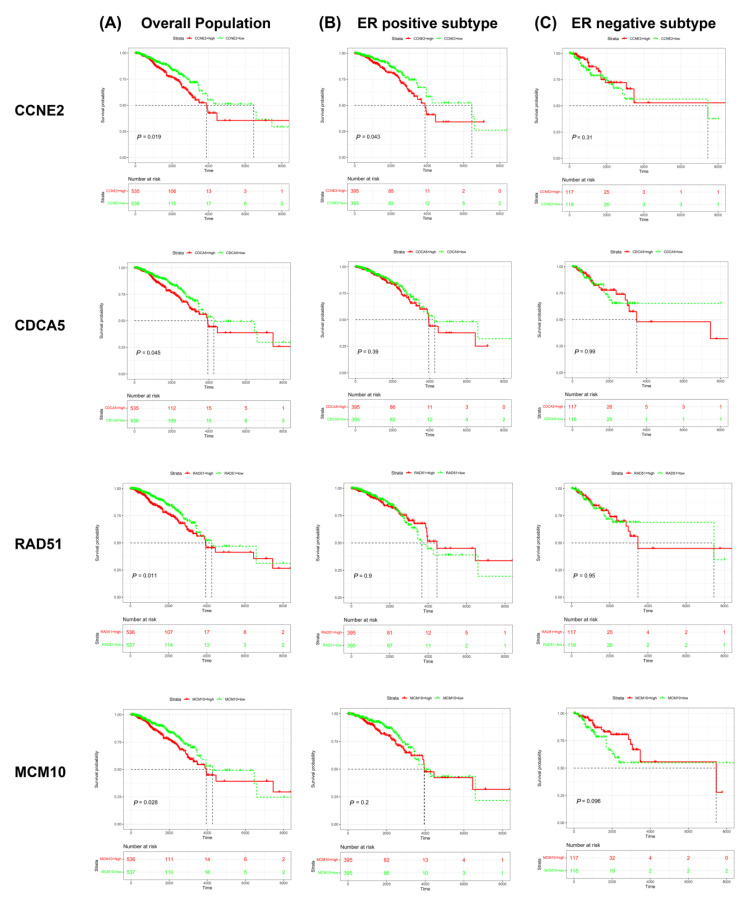
Kaplan–Meier survival curve analysis of the candidate genes (*CCNE2, CDCA5, RAD51,* and *MCM10*) in breast cancer patients. (**A**) Survival curve for all breast cancer patients. (**B**) Survival curve for ER-positive breast cancer patients. (**C**) Survival curve for ER-negative breast cancer patients. Abbreviation: ER, estrogen receptor.

Survival analysis from the TCGA database showed that higher expression of candidate genes (*CCNE2, CDCA5, RAD51,* and *MCM10*) was associated with poor prognosis in the overall population of breast cancer patients (*p* < 0.05) (Figure 6A), whereas *TCF19*, *KNTC1,* and *NEIL3* were not related to poor prognosis in the overall population of breast cancer patients (*p* > 0.05) (Supplementary Figure S1A).

We further analyzed the correlation between the candidate genes and ER expression status of breast cancer. The results indicated that the high expression of *CCNE2* was associated with poor prognosis in ER-positive breast cancer patients (*p* < 0.05) (Figure 6B) but not in ER-negative patients (*p* > 0.05) (Figure 6C). In contrast, the relationship between the expression of *CDCA5*, *RAD51*, and *MCM10* and the poor prognosis does not relate to ER status (*p* > 0.05) (Figure 6B,C). Although the expression of *NEIL3* and the poor prognosis of the overall population of breast cancer lack statistical support, *NEIL3*’s low expression patients show bad prognoses trend in ER-negative patients and have statistical differences (*p* < 0.05). (Supplementary Figure S1B,C)

These findings reveal an association between *CCNE2*, *CDCA5*, *RAD51*, and *MCM10* and worse overall survival in breast cancer patients. *CCNE2* presents a distinct correlation in ER-positive patients.

### 2.5. CCNE2 Expression Was Correlated with ER

**Figure 7:**
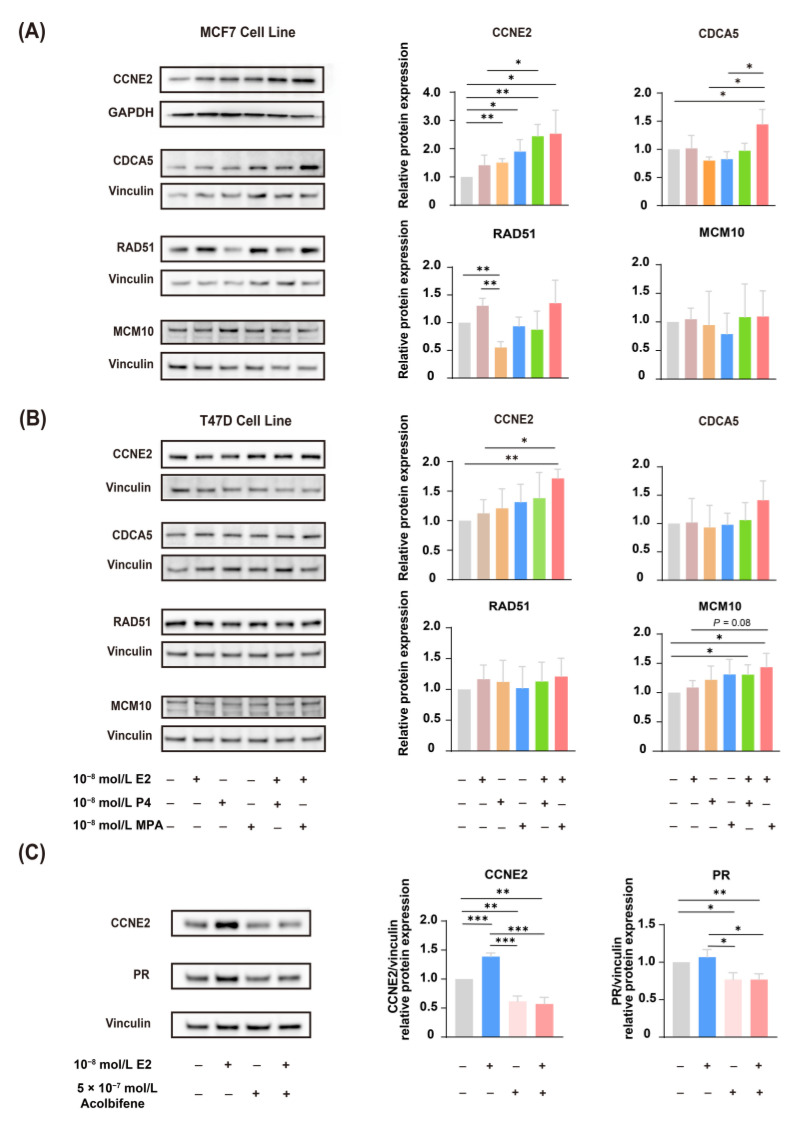
The expression of CCNE2 is correlated with estrogen and progestogen. (**A**) Western blotting analysis validated the expression of CCNE2, CDCA5, RAD51, and MCM10 in MCF-7 human breast cancer cells. The ethanol treatment is used as a control. (**B**) Western blotting analysis validated the expression of CCNE2, CDCA5, RAD51, and MCM10 in T47D human breast cancer cells. The ethanol treatment is used as a control. (**C**) Acolbifene (EM-652) attenuates the expression of CCNE2. Acolbifene, an estrogen receptor antagonist, was used to block the effects of estrogen through ER. T47D cells were treated with 10^−8^ E2 for 16 h with or without pretreatment with acolbifene for 24 h. The DMSO treatment was used as a control. The data are normalized to the control group. Statistical significance is indicated: * *p* < 0.05, ** *p* < 0.01, and *** *p* < 0.001. E2, 17β-estradiol; P4, progesterone; MPA, medroxyprogesterone acetate; ER, estrogen receptor; PR, progesterone receptor.

To further validate RT-qPCR data (Figure 3C), Western blotting analysis was performed to validate the expression of CCNE2, CDCA5, RAD51, and MCM10 in both MCF-7 (Figure 7A) and T47D (Figure 7B) human breast cancer cells. Western blotting results revealed that EPT induced higher expression of CCNE2 compared to ET treatment (*p* < 0.05), which was coincident with the transcript expression (Figure 7A,B).

We next investigated whether the expression of CCNE2 induced by estrogen was through ER activation. We used acolbifene [20,21], the most effective estrogen receptor antagonist in blocking ER. As we know, estrogen-controlled progesterone receptor (PR) synthesis was mediated by ER; thus, PR was always used as a marker of estrogen activity in breast cancer [22,23]. The results showed that estradiol can induce both PR and CCNE2 expression, while pretreatment of acolbifene prevents PR induction by estradiol; CCNE2 was also downregulated after the pretreatment of acolbifene (Figure 7C). These results suggested that ER system should be involved in the expression of CCNE2.

We conducted a molecular docking analysis to investigate whether E2, P4, and MPA would bind to CCNE2; the results showed that CCNE2 might bind to E2 (−6.791 kcal/mol) (Supplementary Figure S2A), P4 (−6.847 kcal/mol) (Supplementary Figure S2B), and MPA (−6.314 kcal/mol) (Supplementary Figure S2C) with a relatively strong binding affinity, respectively. In addition, the results of the docking suggested a hydrogen bond between P4 and Arg294 residue of the CCNE2 (distance: 2.3 Å) (Supplementary Figure S2B) and a hydrogen bond between MPA and Trp107 residue of the CCNE2 (distance: 2.3 Å) (Supplementary Figure S2C).

### 2.6. Interactions between CCNE2 and Breast Cancer Driver Genes

**Figure 8:**
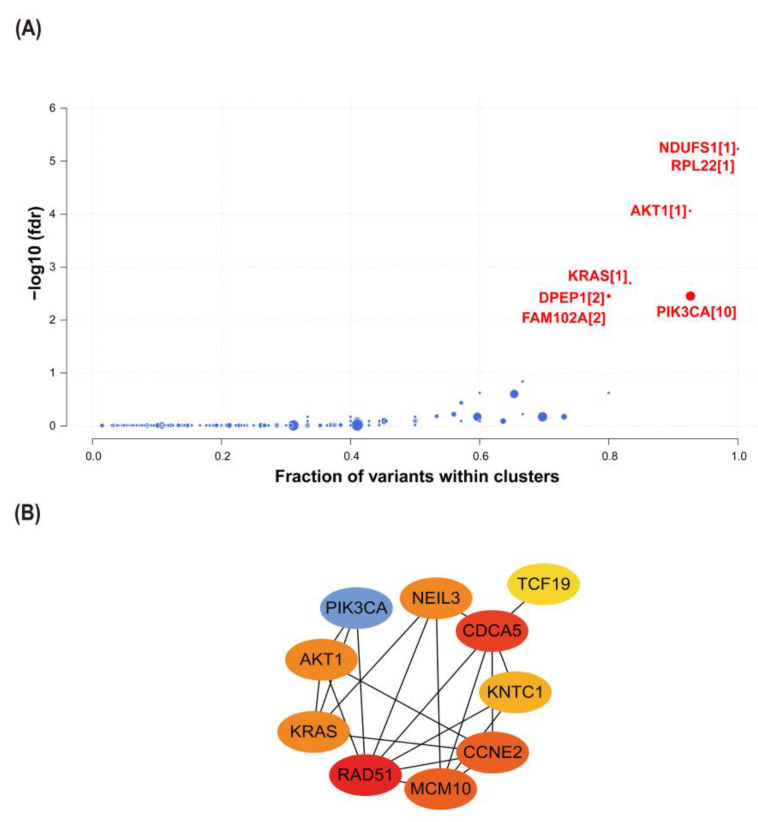
The interactions between *CCNE2* and cancer driver genes. (**A**) The cancer driver genes identified by the OncodriveCLUST algorithm in breast cancer patients from TCGA-BRCA. Red dots represent cancer driver genes with statistical significance. (**B**) The construction of a PPI network between *CCNE2* and cancer driver genes. FDR, false discovery rate.

Cancer driver genes were an important factor in promoting the progression of precancer lesions to cancer [24,25]. Using the Oncodrive CLUST algorithm, we identified seven cancer driver genes from the TCGA-BRCA database, including (*AKT1, KRAS, NADH: ubiquinone oxidoreductase core subunit S1* (NDUFS1)*, ribosomal protein L22* (RPL22)*, dipeptidase 1* (DPEP1)*, phosphatidylinositol-4,5-bisphosphate 3-kinase catalytic subunit alpha* (PIK3CA), and *family with sequence similarity 102 member A* (FAM102A)) (Figure 8A). PPI analysis showed potential interactions between three cancer driver genes (*AKT1, KRAS,* and *PIK3CA*) and candidate genes (*CCNE2, RAD51,* and *NEIL3*) (Figure 8B).

### 2.7. CCNE2 Related to Genomic Stability in Breast Cancer Patients

**Figure 9:**
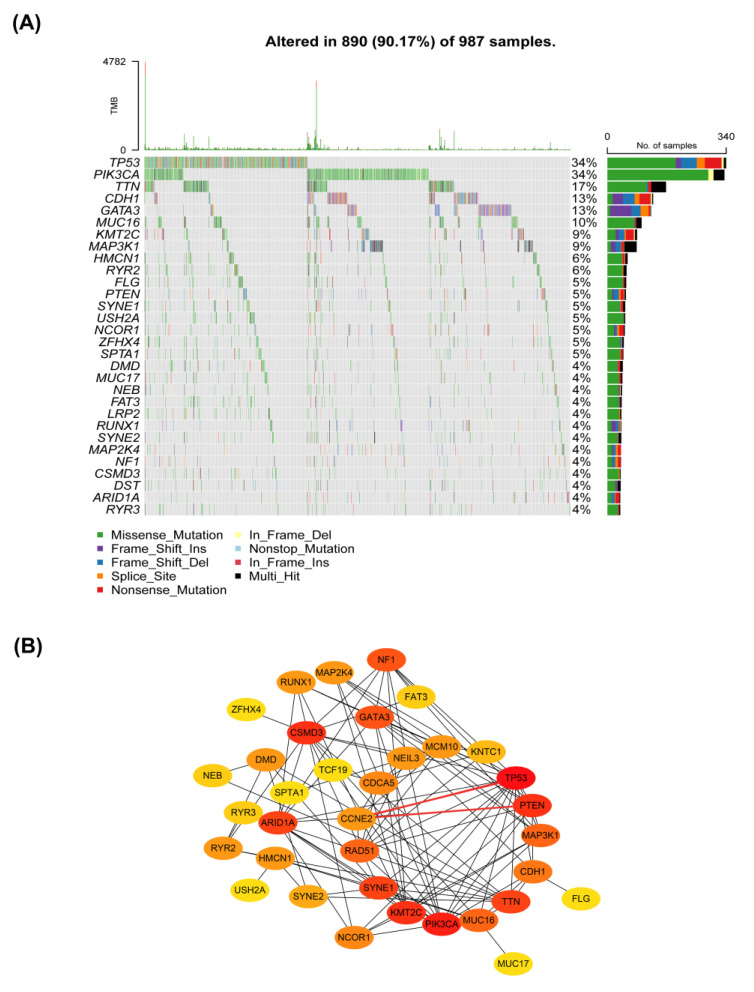
Protein–protein interactions between seven candidate genes (*CCNE2, CDCA5, RAD51, TCF19, KNTC1, MCM10,* and *NEIL3*) and the top 30 most frequently mutated genes in the TCGA-BRCA. (**A**) The waterfall plot of mutation profiles of the top 30 most frequently mutated genes in 987 BRCA samples. (**B**) Interactions between seven candidate genes (*CCNE2, CDCA5, RAD51, TCF19, KNTC1, MCM10,* and *NEIL3*) and the top most frequently mutated genes.

The genomic instability was defined by a high mutation frequency consisting of nucleic acid sequence changes and chromosomal hyper-change, which were tightly associated with cancer development and progression [26,27]. To investigate the relationship between *CCNE2* and genomic stability, we first comprehensively analyzed the mutation landscape in breast cancer patients using the R package “maftools”. The most common type of mutation was missense mutation in breast cancer patients. The most frequent type was single nucleotide polymorphism (SNP), followed by deletion (DEL) (Supplementary Figure S3). Next, we identified the top 30 genes with the highest frequency of mutation in the TCGA-BRCA database (*n* = 987). We found that *TP53* had the highest frequency of the mutations (Figure 9A).

PPI network analysis showed interactions among seven candidate genes (*CCNE2, CDCA5, RAD51, TCF19, KNTC1, MCM10,* and *NEIL3*) and genes with the highest mutation frequency (Figure 9B). *CCNE2* interacted with *TP53* and *PTEN* (Figure 9B). *TP53* and *PTEN* were key tumor suppressors and played a key role in maintaining genomic stability [28,29,30]. Therefore, *CCNE2* might be linked to genomic stability in breast cancer.

## 3. Discussion

Breast cancer remains the most common malignancy in females worldwide [31]. The long-term safety between ET and EPT and breast cancer risk has been discussed over the past decades [9,10,11,32]. Both epidemiological [18] and experimental evidence [33] suggested that EPT significantly promoted cell proliferation assessed by a classical proliferation marker Ki-67/MIB, compared with baseline (before treatment) (*p* = 0.003) in 71 postmenopausal women. We found that the addition of estrogen affected the breast cancer cells’ gene expression profiles in response to P4 and MPA (Supplementary Table S1). However, there was no direct evidence on whether ET and EPT were associated with breast tumorigenesis.

Furthermore, MPA is the most widely used synthetic progestin in EPT. MPA and P4 have different biological effects, such as affinities for PR, unique intracellular signaling pathways, potencies, metabolism, pharmacokinetics, efficacy, side effects, and off-target effects based on their different structures [34], and they also have different risks of breast cancer [8]. Our study pays attention to the DEGs of both P4 and MPA with or without E2. Moreover, it further expanded from the expression level of breast cancer tissue, providing candidate genes for monitoring breast cancer risk in MHT applications. Further study of EPT and ET’s function might gradually clarify its molecular mechanisms in breast tumorigenesis.

In this study, we found that EPT was intended to increase the expression of cell cycle-related genes, such as *CCNE2, CDCA5, RAD51, TCF19, KNTC1, MCM10,* and *NEIL3*, compared to the ET (*p* < 0.05). Western blotting analysis revealed that upregulation of CCNE2 could be significantly suppressed by ER antagonist acolbifene. Horwitz [23] showed that estrogen regulates a progesterone ‘receptor’; thus, PR could be used as an ideal marker of a measurable product of estrogen action on hormone responsiveness in ER-positive tumor cells. Interestingly, CCNE2 might bind to E2 (−6.791 kcal/mol), P4 (−6.847 kcal/mol), and MPA (−6.314 kcal/mol) with a relatively strong binding affinity, respectively. Moreover, *CCNE2* might interact with *AKT1* and *KRAS* (cancer driver genes of breast cancer) and *TP53* and *PTEN* (the two most frequently mutated genes in breast cancer). Consequently, these findings suggested a closer linkage between *CCNE2* expression and EPT. CCNE2 may be a candidate risk indicator of breast cancer in women using MHT.

*CCNE2,* a member of the cyclin E family of proteins, was a key factor in regulating the cell cycle and was mainly responsible for regulating the gap 1/synthesis phase (G1/S) transition of the cell cycle [35,36]. Cyclin E could activate the Cdk2 holoenzyme and phosphorylates many targets, including retinoblastoma protein (Rb) and p21, during the G1/S transition of the cell cycle [36]. Overexpression of *CCNE2* has been found in various types of cancer, such as breast cancer [37] and lung cancer [38]. Sieuwerts AM et al. [37] indicated that high *CCNE2* expression was associated with significantly poor prognosis in female patients with lymph node-negative breast cancer. These were consistent with the results we achieved.

Further, we found CCNE2 potentially bound to E2, P4, and MPA using molecular docking analysis, suggesting a tight connection between CCNE2 and E2, P4, and MPA, although the specific molecular mechanism remained unclear. Our study reported that EPT significantly upregulated the expression levels of CCNE2.

Previous studies [39,40] have shown that the tumor can be latent for 15–20 years in the body before reaching the clinical detection size. Hence, long-term MHT may shorten the time for these tumor cells to achieve clinical testing. CCNE2 may be the key gene contributing to preexisting occult breast cancer progression under MHT treatments, especially in ER-positive patients.

Nevertheless, it remained unclear how overexpressed *CCNE2* promoted tumorigenesis [41,42]. The underlying mechanisms of tumorigenesis might be related to genomic instability. Overexpression of cyclin E led to replication stress in the S phase and chromosome segregation errors in the mitotic phase, contributing to genomic instability [41]. A recent study [43] indicated that high expression levels of *CCNE2* would increase the combination with minichromosome maintenance protein MCM2 and MCM7 of the pre-replication complex, subsequently promoting genomic instability and cell proliferation in breast cancer. Moreover, our results showed that *CCNE2* might interact with cancer driver genes (*AKT1* and *KRAS*), *TP53* (the most frequently mutated gene), and *PTEN* in breast cancer patients, suggesting *CCNE2* might be associated with tumorigenesis and genome stability. As was well known, *AKT1*, *KRAS, TP53,* and *PTEN* played key roles in cell proliferation, survival, and genome stability [29,44,45,46,47,48]. However, it should be noted that these possible interactions among *CCNE2*, driver genes, *TP53,* and *PTEN* need to be further confirmed in the future. In summary, our findings supported that increased expression of *CCNE2* might be relevant to EPT-related tumorigenesis.

Meanwhile, *MCM10* was essential for DNA replication in all eukaryotes [49]. Human *MCM10* protein declined in the late M phase, remained low in the G1 phase, and accumulated and bound chromatin in the S phase [50]. Previous studies indicated *MCM10* was required for the assembly of the Cdc45-Mcm2-7-GINS (CMG) helicase complex involved in the initiation of DNA replication in human cells [51] and promoted DNA elongation by interacting with DNA Pol-α [52]. In addition, the preceding literature [53] reported RBBP6/ZBTB38/MCM10 axis was crucial to genome stability in mammalian cells, and the expression of *MCM10* in RBBP6-depleted cells could prevent DNA damage. Notably, *MCM10* overexpression was observed in breast cancer patients, and knockdown of *MCM10* dramatically reduced tumor cell growth in the mouse model of breast cancer [54]. Survival analysis revealed that high *MCM10* expression was associated with poor prognosis in breast cancer [54].

Besides *CCNE2* and *MCM10*, the other five candidate genes also played an important role in cell cycle progression and genome stability. *CDCA5*, also known as sororin, increased from S to gap 2 phase (G2) and decreased after mitotic exit [55], was required for stable cohesion of sister chromatids in the S and G2/M cell cycle phases [56]. Remarkably, *CDCA5* had been found to have oncogenic activity by disrupting the balance of proliferation and apoptosis in cancer cells [57]. It was reported that *CDCA5* was a negative prognostic marker in multiple cancers, including breast cancer [58], colorectal cancer [57], and hepatocellular carcinoma [59].

*RAD51* recombinase-mediated DNA repair by homologous recombination (HR) [60], with the highest expression in S and G2 phases [61]. *RAD51* was linked to diverse DNA damage sensors, tumor suppressors, and cell cycle regulators [62]. *RAD51* overexpression promoted genome instability [63], and the dynamics of *RAD51* filament formation and stability were vital for tumor suppression by maintaining genomic stability [64]. Maacke H et al. [65] found that wild-type *RAD51* was highly expressed in invasive ductal breast cancer and associated with histological grade. The overexpression of *RAD51* was related to higher tumor grade and characteristics of aggressive tumors (e.g., lack of hormone receptor expression and HER2 amplification) in breast cancer [66].

*TCF19*, also known as SC1, was a critical regulator of the key gluconeogenic genes and played an important role in cell proliferation and survival [67,68]. *TCF19* promoted the proliferation of various cancer cells, such as lung cancer [69] and head and neck cancer [70]. Currently, a study showed that *TCF19* might be linked with the proliferation of breast cancer cells [71].

The *KNTC1* gene, also known as the rough deal (ROD) gene, encoded the protein that was a key component of the spindle assembly checkpoint [72,73] and was enriched in controlling important cellular processes such as DNA damage and repair and cell cycle regulation [74]. The biological functions of *KNTC1* were still unclear in breast cancer. Hence, further studies were needed.

*NEIL3* was a member of the NEIL DNA glycosylase family, and its expression rose in the S phase and peaked in the late S/G2 phase [75]. *NEIL3* was found to protect genomic stability by providing targeted repair of oxidative damage to S/G2 phase telomeres [75]. Of note, *NEIL3* was overexpressed in different types of cancers, including invasive breast carcinoma, pancreatic adenocarcinoma, and lung adenocarcinoma [76]. *NEIL3* overexpression was significantly associated with different stages of tumor progression in breast cancer and poor prognosis in triple-negative breast cancer patients versus non-triple-negative breast cancer [76].

Our results showed that the expression levels of five candidate genes exhibited an upward trend in E2 plus MPA treatment compared with those in the E2 plus P4-treatment. Other studies also reported this differential regulation [19,77]. Probably, it was related to the potency of P4 and MPA. P4 was a C21-steroid hormone in which a pregnane skeleton carried two ketone groups (3,20-dione) at the C3 and C20 positions and had a double bond at the C4–C5 position [78,79]. MPA was a synthetic derivative of P4, and both had progestogen effects [80]. However, MPA showed relatively high progestogen activity when a methyl group was added to C6 compared to P4 [80]. King and Whitehead [81] accessed progestin potency in postmenopausal women who were given different orally administered progestins (MPA and P4) combined with estrogens. The results showed that the MPA is 45 times more effective than the P4 (0.09 and 0.002, respectively) [81]. Hence, we proposed that the difference in drug potency might explain the differential regulation.

In addition, some limitations should be noted in this study. Firstly, as we only analyzed expression profile data from TCGA and GEO databases, larger clinical sample studies based on samples of breast cancer patients with long-term MHT use were needed to confirm these findings. Secondly, the threshold for screening differential expressed genes was artificially determined based on experience and directly taking the intersection of the DEGs of the GEO dataset with the TCGA dataset so that some valuable genes might be missed. Thirdly, in addition to DEGs related to the cell cycle, other DEGs (Supplementary Tables S2 and S6) should also receive extensive attention, such as homologous recombination (HR) repair, DNA conformation change, and DNA replication. We should also caution that the presence of DEG in this study should not be considered the sole explanation for the different BC incidence in ET and EPT treatments. Ward et al. [82] recently verified the hypothesis that estrogen-progestin combinations had a distinct metabolic phenotype over either hormone alone and might increase glycolysis (E2 effects) and promote lipid storage (progestin effects). These results exhibited that combination hormone treatment facilitated metabolism shifting in the breast cancer cell and had a more metabolically adaptive state for cell survival, which was another potential mechanism for tumor progression during MHT with EPT. Fourthly, the specific molecular mechanism of how EPT regulated these genes to promote cell proliferation and affect genome stability required further investigation. Horwitz and Sartorius [83] hypothesize that progestins expand the pre-existent occult malignant cells in the breasts of menopausal women. In the scenario of breast cancers [84], EPT, along with its affected molecular, plays a role in activating dormant disease, generating tumor cell heterogeneity, enhancing the aggressiveness of one or more tumor cell subpopulations, and promoting tumor cell dissemination and metastasis. It would also be interesting to explore whether there were differences in genetic stability, mutation load, or mutation frequency in breast cancer patients between ET and EPT regimens. Additionally, beyond their effects on tumor cells, estrogen and progestins could impact cancer progression through remodeling the tumor microenvironment. Further research in this area was required. Fifth, further validation experiments were needed to verify the results of this study, such as molecular docking results.

In conclusion, we found that seven candidate genes (*CCNE2, CDCA5, RAD51, TCF19, KNTC1, MCM10,* and *NEIL3*), tightly linked to cell cycle regulation, were upregulated by EPT compared with ET (*p* < 0.05). These DEGs genes might be used as candidate indicators for monitoring breast cancer risk in women using MHT. These genes’ biological functions would help us further understand why EPT but not ET increased breast cancer risk in postmenopausal women and the safety of MHT.

## 4. Materials and Methods

### 4.1. Data Sources and Data Preprocessing

In this study, messenger RNA (mRNA) expression profiles were obtained from a GEO dataset (GEO: GSE62243), in which T47D cells were treated with steroid hormones (17β-estradiol [E2], P4, P4+E2 or MPA, MPA + E2) to assess the combined effects of the P4 group (E2 + P4 vs. E2) and MPA group (E2 + MPA vs. E2). The in vitro experiments consisted of three groups: E2 (*n* = 3), E2 + P4 (*n* = 3), and E2 + MPA (*n* = 3), with 24,997 gene expression values measured in each sample. Briefly, we normalized the expression data using the variance stabilization normalization method. The mRNA expression profiles and clinical information of patients with breast cancer were downloaded from The Cancer Genome Atlas Breast Invasive Carcinoma database (TCGA-BRCA), named the BRCA group, which contained 113 adjacent and 1102 tumor tissues, with 19,754 gene expression values measured in each case.

### 4.2. Identification and Functional Enrichment Analysis of DEGs

We normalized the GSE62243 data using variance-stabilized normalization and corrected the data with combat described by Need EF et al. [85]. DEGs in the P4 and MPA groups and the BRCA group (tumor with adjacent normal tissue) were identified with the “Limma” package and the “edgeR” package, respectively, implemented in R (v3.6.1). *p*-values were corrected for multiple hypothesis testing using the Benjamini and Hochberg method provided in the “Limma” package (v3.50.1). The results of the differential expression analysis were presented in Supplementary Tables S2–S6. The absolute value of log (2) fold-change (|log_2_FC|) > 1.0 and FDR < 0.05 were considered as the cutoff values for DEGs screening.

Biological process and pathway enrichment analyses were conducted using Metascape (https://metascape.org/, accessed on 15 May 2022) [86], a comprehensive gene list annotation and analysis resource for experimental biologists, including the following ontology sources: GO Biological Processes, Kyoto Encyclopedia of Genes and Genomes (KEGG) Pathway, and Reactome Gene Sets. FDR < 0.05 was considered to be significant.

### 4.3. Gene Expression, Protein Expression, and Survival Analysis of Candidate Genes

The expression of identified candidate genes in breast cancer patients was assessed using BRCA data from the TCGA cohort. All statistical significances between breast cancer and its adjacent normal breast tissue were calculated by R (v3.6.1). Differences in gene expression among different breast cancer stages were estimated by the Wilcoxon rank-sum test.

The HPA database [87] (https://www.proteinatlas.org/, accessed on 23 May 2022) was used to evaluate the protein expression levels of candidate genes in breast cancer tissues and normal tissues. Kaplan–Meier analysis with log-rank test was performed for survival analysis using R package survival (v3.1.3) and survminer (v0.4.8). *p* < 0.05 was considered to be significant.

### 4.4. Prediction of Cancer Driver Genes

The “maftools” R package (v2.10.5) [88] was applied to the aggregation and visualization of mutation data. Thus, we predicted the driver genes of BRCA, obtained from the TCGA dataset, based on the OncodriveCLUST algorithm [89,90] in the R package “maftools”. OncodriveCLUST was a gene detection method that utilized intra-protein mutation clustering to identify potential cancer driver genes [91]. FDR < 0.05 was considered the cutoff value for identifying driver genes.

### 4.5. Gene Mutation Analysis

Somatic mutation data of BRCA were downloaded from the TCGA dataset and visualized using the “maftools” R package (v2.10.5) [88].

### 4.6. Protein–Protein Interaction (PPI) Network Analysis of Candidate Genes and Top Mutation Genes

The “STRINGdb” R package (v2.6.5) was applied to explore the protein–protein interactions between candidate genes and mutation genes for constructing the PPI network of interested key genes [92]. Then, the gene interactions with a confidence score > 0.4 (medium confidence) were selected for visualization by plug-in cytoHubba in Cytoscape software (v3.7.2, San Diego, CA, USA) [93].

### 4.7. Molecular Docking

The crystal structure of CCNE2 [94] (ID: O96020) was obtained from AlphaFold Protein Structure Database [95]. The 2D structures of E2 (PubChem CID: 5757), P4 (PubChem CID: 5994), and MPA (PubChem CID: 6279) originated from PubChem [96]. All docking simulations were performed by using AutoDock Vina (v1.2.3, Molecular Graphics Laboratory, La Jolla, CA, USA) along with AutoDock Tools (v1.5.7, The Scripps Research Institute, La Jolla, CA, USA) [97], and the structures were visualized with PyMOL open-source (version 2.6.0a0) [98]. The binding energies (kcal/mol) between the receptor and the ligand were calculated to estimate the ligand-binding affinity using AutoDock Vina (v1.2.3).

### 4.8. Reagents

E2 (#E2758-250MG, Sigma Aldrich, St. Louis, MO, USA), P4 (#V900699-5G, Sigma Aldrich, St. Louis, MO, USA), or MPA (#M0250000, Sigma Aldrich, St. Louis, MO, USA) were dissolved in anhydrous ethanol at a concentration of 10^−2^ mol/L stock solutions, stored at −20 °C and prepared the application concentration with the medium. Acolbifene (#HY-16023A, MCE, Monmouth Junction, NJ, USA) was dissolved in DMSO at a concentration of 5 × 10^−4^ mol/L stock solutions, stored at −20 °C, and prepared the application concentration with the medium.

### 4.9. Cell Culture and Treatment

MCF-7 and T47D were obtained from Prof Yu-Mei Feng [99,100]. The cells were grown in RPMI-1640 media (Biological Industries, Beit-Haemek, Israel) with 10% FBS (Biological Industries, Beit-Haemek, Israel) and 1% penicillin/streptomycin in a humidified atmosphere (95% air, 5% CO2) at 37 °C. To avoid background estrogenic stimulation, MCF-7 and T47D cells were cultured in hormone stripped medium (Phenol red-free RPMI1640 (#11835-030, Gibco, Thermo Fisher Scientific, Waltham, MA, USA) supplemented with 10% charcoal-stripped fetal bovine serum (#04-201-1A, Biological Industries, Beit-Haemek, Israel) for 56 h, followed by treatment with 10^−8^ mol/L E2, P4 or MPA for 16 h.

### 4.10. RT-qPCR

Total RNA was extracted from T47D cells using TRIzol reagent (#15596018, Ambion, Austin, TX, USA) according to the manufacturer’s protocol. The A260/A280 ratio of 1.8–2.1 was accepted as pure RNA and used in subsequent experiments. After reverse transcription of mRNA (10 min at 25 °C; 120 min at 37 °C; 5 min at 85 °C and 4 °C for ∞.), cDNA was synthesized using the High-Capacity cDNA Reverse Transcription Kit (#4368814, Applied Biosystems, Foster City, CA, USA). The RT-qPCR reactions were performed with the Power SYBR^®^ Green Master Mix (#A25742, Thermo Fisher, Carlsbad, CA, USA) and tested using ABI Prism 7500 platform (Applied Biosystems, Singapore). The primers used for RT-qPCR were synthesized by BGI TECH SOLUTIONS (BEIJING LIUHE) CO., LIMITED (Beijing, China). Primer sequences are shown in Table 1. The relative expression level by normalizing to 18s levels was calculated using the 2^−ΔΔCt^ method. Unpaired Student’s *t*-test analyzed results, and *p* < 0.05 was accepted as the cutoff value.

### 4.11. Western Blotting

MCF-7 and T47D cells were lysed in lysis buffer (#KGP250/KGP2100, Keygen Biotech, Nanjing, China) containing protease and phosphatase inhibitors following the manufacturer’s protocol. Proteins were subjected to 10% SDS–polyacrylamide gel electrophoresis (SDS-PAGE) gel and transferred onto a PVDF membrane, then incubated with CCNE2 (#ab40890, Abcam; dilution rates of 1:3000), CDCA5 (#67418-1-Ig, Proteintech; dilution rates of 1:1000), RAD51(#14961-1-AP, Proteintech; dilution rates of 1:1000), MCM10 (#12251-1-AP, Proteintech; dilution rates of 1:500), ERα(#ab108398, Abcam; dilution rates of 1:2000), PR(#ab206926, Abcam; dilution rates of 1:1000), Vinculin(#26520-1-AP, Proteintech; dilution rates of 1:2000) and GAPDH antibodies (#T0004, Affinity; dilution rates of 1:5000) at 4 °C overnight, respectively. The next day, the membranes were exposed to secondary antibodies (#S0101, #S0100, Lablead, Beijing, China; 1:5000 dilution) at room temperature for 1 h. Protein bands were measured using Syngene GeneGenius (SYNGENE, GeneGnome XRQ NPC, Cambridge, UK.).

### 4.12. Statistical Analysis

Figures were created, and statistics were calculated using the GraphPad Prism 8 software (GraphPad Software, v8.0.1, San Diego, California, USA) or R software (v3.6.1). The two-tailed unpaired *t*-test was used on RT-qPCR and Western blotting data to compare the differences between the experimental and control groups. Histogram data were presented as mean ± standard deviation (SD) or mean ± standard error of the mean (SEM). *p* < 0.05 was considered significant (*), *p* < 0.01 was considered highly significant (**), and *p* < 0.001 was considered highly significant (***).

## Figures and Tables

**Table 1:** Primer sequences for RT-qPCR.

Gene Symbol	Primer Sequence
*18S*	F: 5′-AGGAATTCCCAGTAAGTGCG-3′ R: 5′-GCCTCACTAAACCATCCAA-3′
*CCNE2*	F: 5′-TCAAGACGAAGTAGCCGTTTAC-3′ R: 5′-TGACATCCTGGGTAGTTTTCCTC-3’
*CDCA5*	F: 5′-AGAAAGTCAGGCGTTCCTACAG-3′ R: 5′-GGGAGATTCCAGGGAGAGTCAT-3′
*RAD51*	F: 5′-TCTCTGGCAGTGATGTCCTGGA-3′ R: 5′-TAAAGGGCGGTGGCACTGTCTA-3′
*TCF19*	F: 5′-AGGCTGGAATTGAGTGATGGAGAC-3′ R: 5′-GTCCTGAGGCTTGACTCGTACTTGTT-3′
*KNTC1*	F: 5′-ATAGTCAACCCAGAGTGGGCTGT-3′ R: 5′-TTTCACGTTTTTCGTCCTGCG-3′
*MCM10*	F: 5′-GAAGAAGGTTACGCCACAGAG-3′ R: 5′-TTTACAGGTTCCCAGGTCAAG-3′
*NEIL3*	F: 5′-GGTCTCCACCCAGCTGTTAAAG-3′ R: 5’-CACGTATCATTTTCATGAGGTGATG-3′

## Data Availability

Publicly available datasets were analyzed in this study. This data can be found here: [https://www.ncbi.nlm.nih.gov/geo/query/acc.cgi?acc=GSE62243].

## Supplementary Materials

The following supporting information can be downloaded at: https://www.mdpi.com/article/10.3390/ijms231911892/s1.

## Abbreviations

*AKT1*	*AKT serine/threonine kinase 1*
*CCNE2*	*Cyclin E2*
*CDCA5*	*Cell division cycle-associated 5*
DEGs	Differentially expressed genes
*DPEP1*	*Dipeptidase 1*
E2	17β-estradiol
ER	Estrogen receptor
EPT	Estrogen plus progestogens treatment
ET	Estrogen treatment
*FAM102A*	*Family with sequence similarity 102 member A*
FDR	False discovery rate
GEO	Gene Expression Omnibus
HPA	The Human Protein Atlas database
HR	Hazards ratio
KEGG	Kyoto Encyclopedia of Genes and Genomes
*KNTC1*	*Kinetochore-associated 1*
*KRAS*	*KRAS proto-oncogene, GTPase*
*MCM10*	*Minichromosome maintenance 10 replication initiation factor*
MHT	Menopausal hormone therapy
MPA	Medroxyprogesterone acetate
MPA group	E2 + MPA vs. E2
*NEIL3*	*Nei-like DNA glycosylase 3*
*NDUFS1*	*NADH:ubiquinone oxidoreductase core subunit S1*
P4	Progesterone
P4 group	E2 + P4 vs. E2
PPI	Protein–protein interaction
PR	Progesterone receptor
*PTEN*	*Phosphatase and tensin homolog*
*RAD51*	*RAD51 recombinase*
*RPL22*	*Ribosomal protein L22*
RR	Relative risk
*TCF19*	*Transcription factor 19*
TCGA	The Cancer Genome Atlas
TCGA-BRCA	The Cancer Genome Atlas Breast Invasive Carcinoma database
*TP53*	*Tumor protein p53*
WHI	Women’s Health Initiative
