# Supplementary material for: Identification of Candidate Genes in Breast Cancer Induced by Estrogen Plus Progestogens Using Bioinformatic Analysis
Source: Int J Mol Sci. 2022 Oct 6;23(19):11892. doi: 10.3390/ijms231911892 (PMC9569986; doi:10.3390/ijms231911892)
Supplement: Supplementary file 1 [file ijms-23-11892-s001.zip › ijms-1813752-supplementary.pdf]

Supplementary Material

1 Supplementary Figure

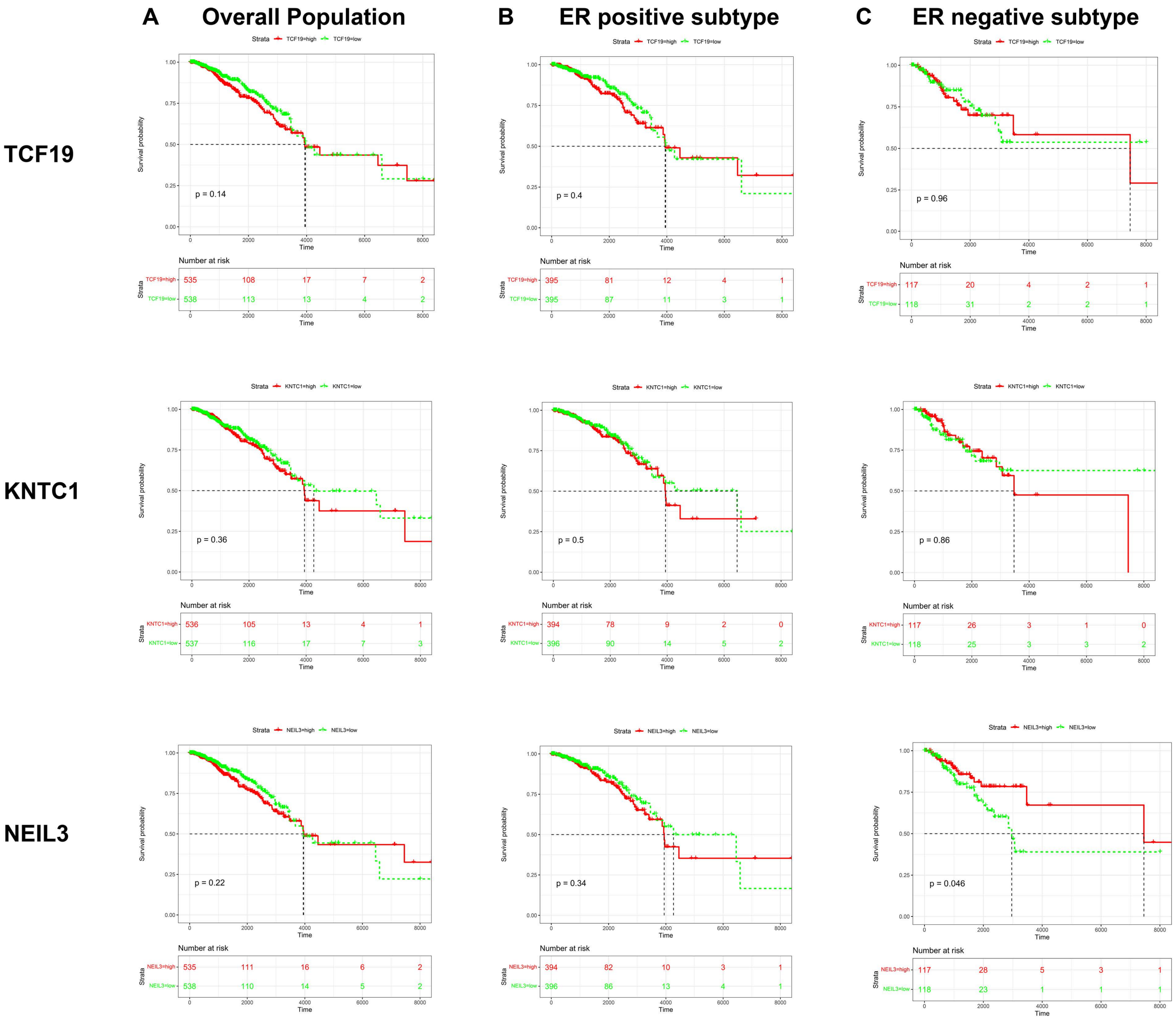

Figure S1. Kaplan-Meier survival curve analysis of the hub genes (*TCF19*, *KNTC1*, and *NEIL3*) in breast cancer patients. (**A**) Survival curve for all breast cancer patients. (**B**) Survival curve for ER-positive breast cancer patients. (**C**) Survival curve for ER-negative breast cancer patients. Abbreviation: ER, estrogen receptor.

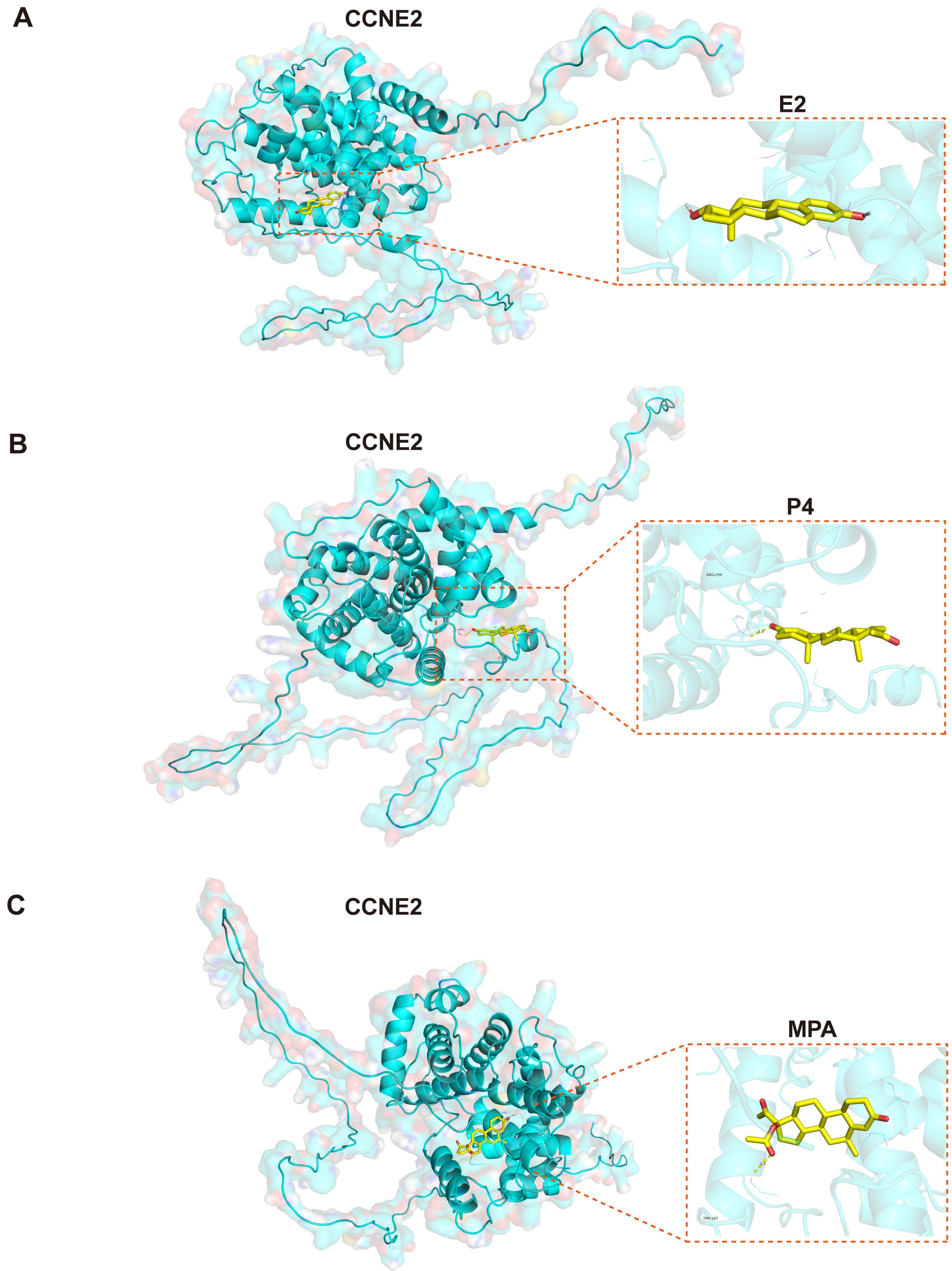

Figure S2. Molecular docking results of CCNE2 binding to E2 (**A**), P4 (**B**), and MPA (**C**).

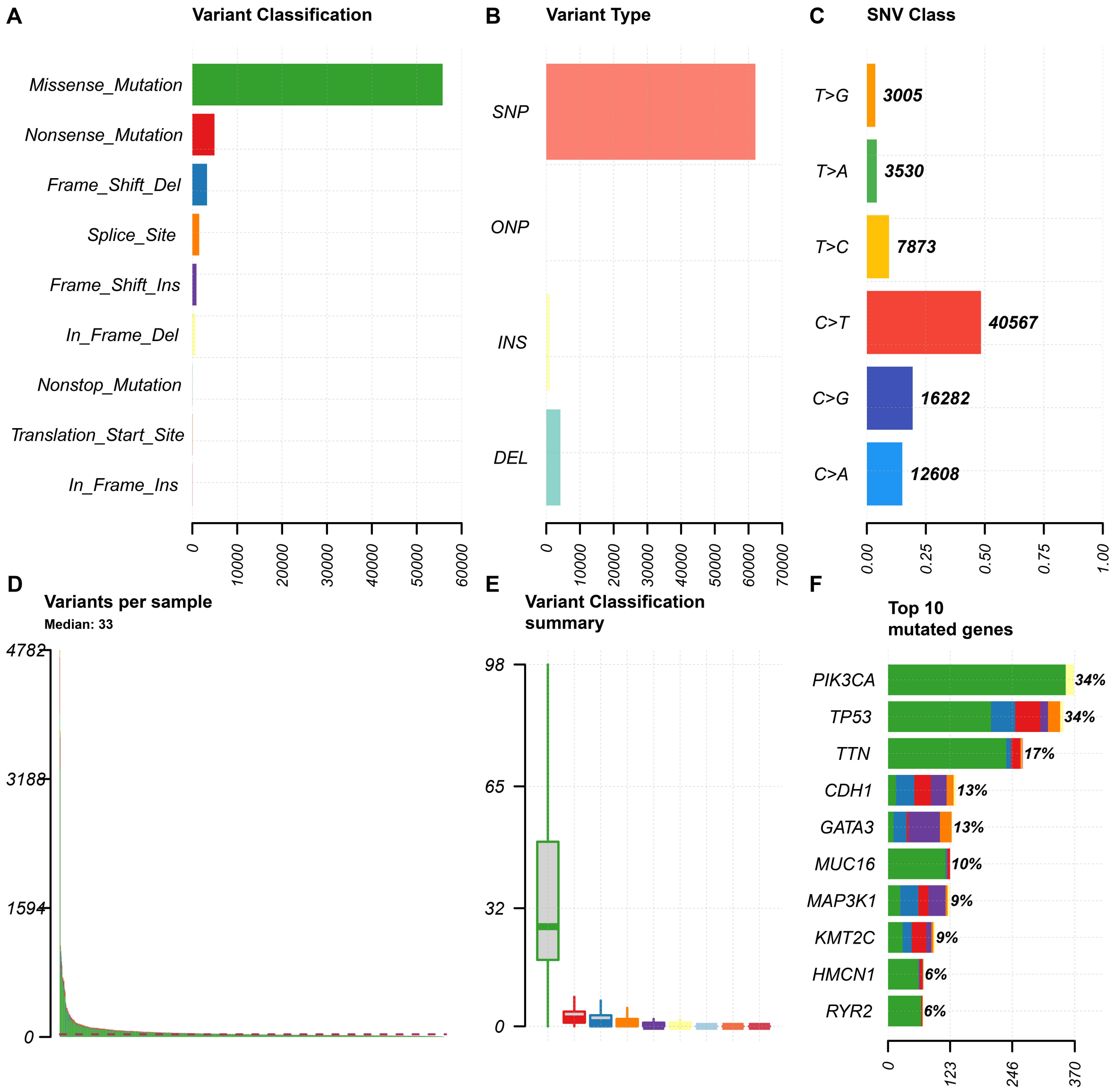

Figure S3. The summary of mutation information in the TCGA-BRCA. (**A**) The variant classifications of gene mutations. (**B**) Types of genome variation. (**C**) Types of single nucleotide changes. (**D**) The median number of mutations per sample. (**E**) The summary of variant classification. (**F**) Top 10 most frequently mutated genes.

2 Supplementary Table

Table S1. The number of DEGs for each progestin compared to a vehicle with or without estradiol, based on GSE62243 analysis.

	Comparison	Up-Regulated DEGs (FC ≥ 1.0 and P.Value < 0.05)	Down-Regulated DEGs (FC ≥ 1.0 and P.Value < 0.05)	Total DEGs
Progestins without E2	P4 vs. Vh	552	131	683
	MPA vs. Vh	1056	302	1358
E2	E2 vs. Vh	196	33	229
Progestins + E2	E2_P4 vs. Vh	1614	437	2051
	E2_MPA vs. Vh	1455	380	1835

Abbreviations: E2: estradiol; P4: progesterone; MPA: medroxyprogesterone acetate; FC: fold change; DEGs: differentially expressed genes.

Table S2. The intersection of the upregulated differential genes among the P4, MPA, and BRCA groups.

No.	Gene names
1	*TCF19*
2	*DEPDC1B*
3	*CDCA5*
4	*PLEKHF2*
5	*CCNE2*
6	*NUSAP1*
7	*KNTC1*
8	*ASPHD2*
9	*ECT2*
10	*NEIL3*
11	*FEN1*

No.	Gene names
12	*CCDC64*
13	*KIF14*
14	*SLC39A6*
15	*HIST1H4C*
16	*POLQ*
17	*SFXN1*
18	*STRBP*
19	*RAD51*
20	*KCNG3*
21	*MCM10*
22	*CKAP2*

No.	Gene names
23	*CHD5*
24	*IQGAP3*
25	*ATAD2*
26	*NELL2*
27	*ATP1A4*
28	*S100P*
29	*KCNJ11*
30	*SAP30*
31	*LYPLA1*
32	*MLPH*
33	*CHRNA5*

No.	Gene names
34	*GALNT6*
35	*GPR89A*
36	*CNTNAP2*
37	*CLIC6*
38	*SCRG1*
39	*PLA2G10*
40	*FXYD3*
41	*CDKN2D*
42	*HMGB3*
43	*HSD11B2*
44	*PTCHD2*
45	*RCC2*
46	*PIK3R2*
47	*PNMT*
48	*LRRC31*
49	*ZBED2*
50	*MFSD7*
51	*ZWINT*
52	*KCNG1*
53	*SCGB1D2*
54	*RAD21*
55	*GPC4*
56	*RAD51AP1*
57	*HIST1H2BF*
58	*GPSM2*
59	*GPR160*
60	*SP6*
61	*HIST1H3B*
62	*PRR11*
63	*HMMR*
64	*ST3GAL4*
65	*PACSIN1*
66	*E2F7*
67	*TMEM63C*
68	*ZIC2*
69	*SLC16A6*
70	*SCUBE2*
71	*EDN2*
72	*FANCD2*
73	*PSPH*
74	*SRP9*
75	*CA12*
76	*GJB2*

No.	Gene names
77	*POLE2*
78	*CENPA*
79	*RDH16*
80	*BRCA1*
81	*KCNK1*
82	*HDAC11*
83	*HMGB2*
84	*RBP2*
85	*H3F3A*
86	*RRM2*
87	*MUC1*
88	*SLC7A8*
89	*KRT4*
90	*KIAA0101*
91	*MMP13*
92	*TCEB1*
93	*APITD1*
94	*CKAP4*
95	*ORM2*
96	*CALR*

Table S3. The 773 DEGs in the P4 group.

Gene names	logFC	AveExpr	t	P.Value	adj.P.Val
MAFB	5.961978	6.488241	40.99148	3.45E-11	4.32E-07
LOC128977	4.887486	1.20182	32.53805	2.50E-10	2.09E-06
SLC25A18	4.076237	5.61993	28.65179	7.43E-10	4.64E-06
NDRG1	4.878863	5.883984	27.67797	9.98E-10	4.99E-06
S100P	6.145261	7.084805	26.17518	1.61E-09	5.98E-06
LOC388610	4.365248	5.398122	24.61498	2.71E-09	8.48E-06
ZBTB16	3.901878	3.743559	24.07292	3.28E-09	9.11E-06
SGK	4.693695	4.969902	23.05439	4.74E-09	1.18E-05
PHACTR3	3.742773	3.358618	22.58274	5.65E-09	1.28E-05
FKBP5	4.59544	7.915282	20.98817	1.05E-08	2.13E-05
TIPARP	3.433586	6.666141	20.71899	1.17E-08	2.13E-05
ATP1A1	3.509602	7.993642	19.375	2.07E-08	3.45E-05
FBXO32	2.720752	7.056045	18.96822	2.47E-08	3.63E-05
PNMT	3.265166	3.73464	18.84539	2.61E-08	3.63E-05
RBP2	3.384705	4.370836	18.54078	3.00E-08	3.95E-05
GPSM2	3.233088	6.852612	18.27874	3.38E-08	4.23E-05
FLJ41603	2.889458	5.717883	18.08099	3.71E-08	4.41E-05
LOC93622	2.805099	7.812527	17.6881	4.46E-08	5.07E-05
SLC26A3	2.7181	2.802675	17.55616	4.75E-08	5.16E-05
TSC22D1	2.510788	6.832355	17.30536	5.36E-08	5.25E-05
RPS6KA5	2.59837	4.078579	17.26885	5.46E-08	5.25E-05
NFKBIA	3.41585	9.562685	17.26754	5.46E-08	5.25E-05
CLDN8	3.890344	5.633089	16.97843	6.29E-08	5.82E-05
FLJ20366	2.45961	4.690229	16.7625	7.01E-08	6.25E-05
ACSL1	3.122502	5.882675	16.65842	7.38E-08	6.36E-05
KCNG1	2.845313	4.416772	16.55978	7.76E-08	6.46E-05

Gene names	logFC	AveExpr	t	P.Value	adj.P.Val
RDH16	2.364535	4.433418	16.14458	9.60E-08	7.65E-05
DSCR6	2.694187	6.896664	16.10614	9.79E-08	7.65E-05
ALPL	3.520135	3.436931	15.89205	1.10E-07	8.30E-05
ATAD4	2.867615	7.393206	15.74754	1.18E-07	8.69E-05
HSD11B2	2.604193	4.277689	15.3372	1.47E-07	0.000105
STAT5A	2.344488	4.324637	15.07074	1.71E-07	0.000115
TSC22D3	2.177554	3.670239	15.03113	1.74E-07	0.000115
MLLT7	2.166394	4.309871	14.61892	2.20E-07	0.000141
STMN3	2.195327	5.869853	14.57123	2.26E-07	0.000141
SERPINA3	2.414096	4.663972	14.38406	2.52E-07	0.00015
TM4SF11	2.425354	4.532835	14.32399	2.60E-07	0.00015
SGPP2	2.136923	2.141253	14.28357	2.67E-07	0.00015
DDIT4	2.126637	6.822588	14.28236	2.67E-07	0.00015
CYP27A1	2.048517	3.876249	14.25977	2.70E-07	0.00015
PACSIN1	2.398944	7.064347	14.2176	2.77E-07	0.000151
ADAMTS8	2.896168	4.320929	13.98663	3.17E-07	0.000169
P4HA2	2.279293	7.318828	13.90156	3.34E-07	0.000174
BNIP3	2.361594	8.37333	13.78681	3.58E-07	0.000182
STOM	3.309304	8.752236	13.61557	3.97E-07	0.00019
ADD3	2.41737	4.540245	13.53854	4.16E-07	0.000192
C6orf85	2.858206	7.464341	13.50191	4.25E-07	0.000193
FLJ14503	1.933482	2.334969	13.36989	4.61E-07	0.000202
ATP1B1	4.750905	8.056857	13.3531	4.66E-07	0.000202
FAM105A	1.932324	3.968372	13.34459	4.68E-07	0.000202
CRIM1	2.790802	4.834842	13.22424	5.05E-07	0.000214
K6IRS3	3.048269	3.239948	13.15512	5.27E-07	0.00022
SERPINA5	2.499051	2.444276	13.00081	5.81E-07	0.000236
PDK4	2.105268	2.559078	12.873	6.30E-07	0.00025
PXMP4	2.608026	7.191444	12.59854	7.53E-07	0.000282

Gene names	logFC	AveExpr	t	P.Value	adj.P.Val
CORO2B	1.909733	1.93223	12.58351	7.60E-07	0.000282
CLIC6	2.307834	7.67961	12.57918	7.62E-07	0.000282
SLC37A2	2.088163	1.923416	12.47312	8.17E-07	0.000296
MYO10	1.968502	6.751909	12.41916	8.47E-07	0.000302
KLF9	1.827789	4.398686	12.3699	8.75E-07	0.000308
RASGEF1A	1.809503	5.320932	12.35018	8.86E-07	0.000308
AKR1D1	2.191231	1.695663	12.32528	9.01E-07	0.000308
NCKIPSD	1.735933	4.956486	12.2332	9.58E-07	0.000319
NEBL	1.86264	5.011349	12.08024	1.06E-06	0.000347
CEBPD	2.307998	6.81648	12.07174	1.07E-06	0.000347
THRSP	2.092599	2.218952	12.04089	1.09E-06	0.00035
SAP30	1.713077	5.018934	11.95256	1.16E-06	0.000365
TXNIP	1.776418	7.79683	11.87893	1.22E-06	0.000374
NELL2	1.96803	2.322251	11.85771	1.24E-06	0.000374
SEC14L2	2.119969	4.047985	11.8515	1.24E-06	0.000374
CG018	1.853903	2.009793	11.7189	1.36E-06	0.000405
CD9	1.882698	8.980429	11.63591	1.44E-06	0.000424
DTX2	1.76568	5.520542	11.58003	1.50E-06	0.000434
ZBED2	1.909221	2.384609	11.57016	1.51E-06	0.000434
AK3L2	3.440492	4.772726	11.54083	1.54E-06	0.000438
SLPI	2.628045	4.187567	11.50118	1.59E-06	0.000446
TPARL	1.673501	4.569616	11.37614	1.73E-06	0.000474
MPHOSPH10	2.298226	7.147673	11.36696	1.75E-06	0.000474
ZFP36	1.720337	7.082115	11.31743	1.81E-06	0.000486
SCRG1	2.452965	3.0187	11.15661	2.03E-06	0.000535
HIG2	1.913741	7.647776	11.05309	2.19E-06	0.000571
ENPP1	2.173785	4.540104	11.0229	2.24E-06	0.000578
CD59	2.321603	4.521792	10.96357	2.34E-06	0.000597

Gene names	logFC	AveExpr	t	P.Value	adj.P.Val
C7orf24	2.869595	7.709396	10.92953	2.40E-06	0.000606
TMEM38B	1.995011	6.309002	10.88754	2.48E-06	0.000619
IL20RA	2.000419	4.509441	10.84371	2.56E-06	0.000627
DPYSL2	2.474892	7.729125	10.78072	2.68E-06	0.000648
CA4	1.594262	2.44818	10.74299	2.76E-06	0.000657
ATAD2	2.601676	7.718216	10.58304	3.12E-06	0.000731
BIRC3	2.016918	2.967346	10.57718	3.13E-06	0.000731
FLJ10847	1.754412	4.801111	10.52622	3.25E-06	0.000753
STEAP2	1.680577	4.429807	10.48248	3.36E-06	0.000772
SASH1	2.129231	5.364169	10.4099	3.56E-06	0.000796
SCNN1A	1.995123	7.138009	10.39573	3.60E-06	0.000796
CHPT1	1.619048	8.129218	10.3838	3.63E-06	0.000796
PGAM1	1.576129	7.545585	10.16575	4.31E-06	0.00092
LOC51136	2.328543	4.963745	10.08252	4.60E-06	0.000972
CDKN1A	1.5676	7.620607	10.07466	4.63E-06	0.000972
MTMR9	2.31474	5.249707	10.05625	4.70E-06	0.000978
C1QDC1	1.824493	5.37932	9.972037	5.02E-06	0.001025
PRR11	1.98705	4.549355	9.957773	5.08E-06	0.001025
STEAP1	1.702529	3.83475	9.932349	5.19E-06	0.001037
TBC1D2	1.533466	5.029482	9.918938	5.24E-06	0.00104
PIK3R1	1.785738	4.155275	9.832996	5.62E-06	0.001106
RRM2	2.664735	5.195801	9.781889	5.86E-06	0.001136
NOTCH2	1.716059	5.175928	9.780696	5.87E-06	0.001136
CENPA	1.476139	6.521509	9.745995	6.03E-06	0.001151
LAMC3	1.771368	1.959508	9.739396	6.07E-06	0.001151
ZIC2	1.942693	4.985585	9.727328	6.13E-06	0.001151
TMEM63C	1.89659	2.356109	9.664583	6.45E-06	0.001189
LAMA3	1.562311	3.904557	9.660602	6.47E-06	0.001189
LOC653994	3.539039	6.421077	9.660498	6.47E-06	0.001189

Gene names	logFC	AveExpr	t	P.Value	adj.P.Val
LSM12	1.835872	4.558279	9.652028	6.52E-06	0.001189
MRFAP1	1.719803	10.7029	9.581235	6.91E-06	0.001242
FAM107B	1.918092	7.08566	9.573019	6.96E-06	0.001242
BCL7C	1.625905	6.85577	9.443594	7.75E-06	0.001375
DEGS1	1.472941	7.217258	9.428671	7.85E-06	0.001382
APM-1	2.112136	2.230457	9.387498	8.13E-06	0.001411
AK3L1	1.786669	4.1509	9.370474	8.25E-06	0.001422
PIM2	1.331975	4.926302	9.351819	8.38E-06	0.001434
TMEM56	1.59715	3.846335	9.324247	8.58E-06	0.001458
LCMT1	1.944425	9.195602	9.30827	8.69E-06	0.001468
PTPNS1	1.355264	4.918528	9.286115	8.86E-06	0.001482
CAMK2N1	1.541216	6.763556	9.281847	8.89E-06	0.001482
GOT1	1.573401	9.600404	9.252782	9.11E-06	0.001509
CCDC34	2.002453	6.696003	9.238729	9.22E-06	0.001517
MAN1A1	2.07752	3.910705	9.186992	9.64E-06	0.001565
CMTM7	1.591277	7.794629	9.163417	9.84E-06	0.001583
PNMA2	1.469352	1.665778	9.159231	9.88E-06	0.001583
TMEM87A	1.670924	8.510614	9.142135	1.00E-05	0.001586
LYPLA1	2.3722	6.223133	9.098456	1.04E-05	0.001624
WWC1	1.603659	8.220228	9.08508	1.05E-05	0.001624
RANBP1	1.63152	7.341906	9.084506	1.05E-05	0.001624
MLPH	1.422023	9.115949	9.081195	1.06E-05	0.001624
LOC652350	1.496151	1.489368	9.07826	1.06E-05	0.001624
ACADM	2.094584	5.053866	9.070274	1.07E-05	0.001626
ATBF1	2.396897	4.933612	9.057789	1.08E-05	0.001626
C9orf152	1.54571	8.901333	9.050172	1.09E-05	0.001626
LOC652838	1.654387	3.935038	9.049324	1.09E-05	0.001626
Pfs2	1.308142	10.37155	9.007327	1.13E-05	0.001677

Gene names	logFC	AveExpr	t	P.Value	adj.P.Val
PEA15	1.468191	8.328134	8.923225	1.21E-05	0.001773
AUTS2	1.492743	7.045976	8.898491	1.24E-05	0.0018
CA12	1.677892	9.533514	8.893022	1.25E-05	0.0018
MAP3K6	1.568533	5.502271	8.857094	1.29E-05	0.001842
C10orf78	1.476009	4.186815	8.854078	1.29E-05	0.001842
HOMER2	1.705853	6.055457	8.818358	1.33E-05	0.001889
LOC641997	1.647615	1.459207	8.812557	1.34E-05	0.001889
GPR160	1.654891	5.304006	8.806548	1.35E-05	0.001889
C6orf81	2.733206	3.035729	8.794935	1.36E-05	0.001898
MCM10	1.351904	5.928947	8.764826	1.40E-05	0.001899
ZNF541	1.758171	3.020876	8.763518	1.40E-05	0.001899
GJE1	1.327342	1.737825	8.753203	1.41E-05	0.001899
GJB2	1.606602	3.92954	8.751602	1.41E-05	0.001899
PGRMC2	1.657448	8.987266	8.745305	1.42E-05	0.001899
EHF	2.121584	4.611244	8.732939	1.44E-05	0.00191
LOC112714	2.138185	2.7196	8.705465	1.47E-05	0.001944
LOC653573	2.552812	4.598333	8.701499	1.48E-05	0.001944
P8	1.79539	10.26644	8.69085	1.49E-05	0.001953
LOC653764	1.288407	3.297277	8.613228	1.60E-05	0.002073
C14orf24	2.586968	4.80284	8.583338	1.64E-05	0.002119
TACC2	1.397552	5.48931	8.570188	1.66E-05	0.002128
F3	2.194144	3.002646	8.555909	1.69E-05	0.002128
CCDC21	1.468875	4.656931	8.551044	1.69E-05	0.002128
AZGP1	2.298053	5.624828	8.550259	1.69E-05	0.002128
IQCB1	1.698754	4.471725	8.548533	1.70E-05	0.002128
OCA2	1.826719	1.48887	8.545206	1.70E-05	0.002128
OACT2	1.71055	4.09721	8.535595	1.72E-05	0.002136
CDKN1B	1.8764	4.68835	8.518752	1.74E-05	0.002158
Ells1	1.660331	5.609438	8.507751	1.76E-05	0.002162

Gene names	logFC	AveExpr	t	P.Value	adj.P.Val
BRCA1	1.370122	3.621142	8.505967	1.76E-05	0.002162
ST3GAL4	1.617078	6.823832	8.499448	1.78E-05	0.002165
NET1	2.302762	6.615416	8.487439	1.79E-05	0.002174
PIK3R2	1.456972	8.034208	8.484261	1.80E-05	0.002174
SCUBE2	1.770764	6.006014	8.469773	1.82E-05	0.002192
FXYD3	1.520758	4.167815	8.455702	1.85E-05	0.002208
PTCHD2	1.63399	2.375112	8.451681	1.85E-05	0.002208
THOC4	1.742498	7.796509	8.42385	1.90E-05	0.002255
LOC650122	1.544913	2.130534	8.417409	1.91E-05	0.002257
ATP1A4	1.221151	1.690481	8.402932	1.94E-05	0.002261
GM2A	1.577786	7.547177	8.40214	1.94E-05	0.002261
RAD21	1.466336	4.560446	8.400489	1.94E-05	0.002261
ZA20D3	1.597825	5.57172	8.383807	1.98E-05	0.002277
LRRC31	1.471537	3.858366	8.369373	2.00E-05	0.002283
LOC653542	1.633708	3.801543	8.356238	2.03E-05	0.002292
BCL6	1.785686	6.402717	8.343909	2.05E-05	0.002308
SRD5A1	1.222628	3.864019	8.334172	2.07E-05	0.002318
FAH	1.491011	7.001513	8.289639	2.16E-05	0.002395
KIAA1904	1.253908	1.693885	8.281347	2.17E-05	0.002403
AHNAK	2.507777	10.61008	8.261648	2.21E-05	0.002433
LOC646299	1.437532	1.445019	8.224555	2.29E-05	0.0025
CCNDBP1	1.239723	5.36784	8.220413	2.30E-05	0.0025
AXUD1	1.24467	5.858614	8.206	2.33E-05	0.002524
TAGLN	1.313722	3.770132	8.191596	2.36E-05	0.00253
VIPR1	1.416244	5.700754	8.189743	2.37E-05	0.00253
ERRFI1	1.977066	6.689496	8.185129	2.38E-05	0.00253
BNIP3L	1.751316	6.319417	8.140575	2.48E-05	0.002617
DCLRE1C	1.343377	4.820844	8.10915	2.56E-05	0.002685

Gene names	logFC	AveExpr	t	P.Value	adj.P.Val
SLC41A2	1.307775	4.502884	8.094268	2.59E-05	0.002712
DKFZP564O0823	1.257414	1.927238	8.089434	2.60E-05	0.002713
LOC644940	1.317737	2.207845	8.062355	2.67E-05	0.002772
RAD51	1.333279	4.057515	8.058008	2.68E-05	0.002772
PARN	1.211506	6.360092	8.051811	2.70E-05	0.002777
ZDHHC14	1.363155	4.891276	8.038887	2.73E-05	0.0028
CALD1	1.852499	4.433795	8.017269	2.79E-05	0.002824
HMGB3	1.899296	5.703732	7.946733	2.99E-05	0.002999
RAD51AP1	1.455024	4.950804	7.939776	3.01E-05	0.003001
LOC652924	2.426791	5.088049	7.93774	3.01E-05	0.003001
AP1B1	1.297408	6.572036	7.928787	3.04E-05	0.003015
LOC652294	1.649515	1.106559	7.920028	3.07E-05	0.003029
KCNK1	1.162696	3.758053	7.896143	3.14E-05	0.003075
LPIN1	1.723408	5.113166	7.887242	3.16E-05	0.00309
FAM46C	1.182593	3.744646	7.839871	3.31E-05	0.003192
SCNN1G	1.719847	2.551398	7.838889	3.32E-05	0.003192
STK39	1.873152	4.609932	7.829068	3.35E-05	0.003208
PRKAB2	1.314013	4.664859	7.824956	3.36E-05	0.003209
C17orf79	1.382381	8.89121	7.79514	3.46E-05	0.003291
SLD5	1.700356	4.955966	7.763416	3.57E-05	0.003357
EFHD1	1.713453	11.28179	7.75801	3.59E-05	0.003362
CREB3L2	1.575135	6.050532	7.754138	3.61E-05	0.003363
CHMP4B	1.204795	6.914581	7.746151	3.63E-05	0.003377
DUSP1	1.38804	6.210247	7.736298	3.67E-05	0.003397
AFMID	1.720231	4.823678	7.729956	3.69E-05	0.003406
MCEE	1.297244	7.569345	7.722348	3.72E-05	0.003419
LOC653105	1.241705	1.449985	7.712361	3.76E-05	0.00344
AP3S1	1.877454	6.998511	7.705649	3.78E-05	0.00345
BTC	1.182394	2.087002	7.687537	3.85E-05	0.003463

Gene names	logFC	AveExpr	t	P.Value	adj.P.Val
HSD17B2	1.466303	2.243495	7.687534	3.85E-05	0.003463
CDKN2D	1.673643	3.941411	7.683846	3.86E-05	0.003463
LOC81691	1.205541	6.488036	7.670559	3.92E-05	0.003496
C15orf38	1.120752	4.16758	7.666495	3.93E-05	0.003498
TMEM16A	1.183121	4.112848	7.64288	4.03E-05	0.003534
C6orf72	1.230108	7.170125	7.640843	4.03E-05	0.003534
SCP2	1.511422	3.130379	7.638547	4.04E-05	0.003534
ZNF652	1.16139	3.672699	7.62454	4.10E-05	0.00357
XPNPEP1	1.138923	7.279742	7.621399	4.11E-05	0.00357
MARVELD3	1.41436	5.540521	7.614324	4.14E-05	0.00357
ABCC8	2.602965	2.939563	7.606428	4.17E-05	0.003586
TAF5L	1.262799	4.869207	7.601618	4.19E-05	0.003591
FMO5	2.252985	3.874355	7.593502	4.23E-05	0.003608
LOC399942	1.093037	7.434319	7.586437	4.26E-05	0.003621
NAP1L4	1.456538	8.7863	7.557034	4.39E-05	0.003705
RHOU	1.726012	2.953965	7.530702	4.50E-05	0.003768
GLUL	1.336477	2.755253	7.530097	4.51E-05	0.003768
RBBP7	1.640832	8.973756	7.515081	4.58E-05	0.00381
GPR124	1.603991	2.582464	7.512483	4.59E-05	0.00381
C9orf52	1.355451	4.685056	7.501553	4.64E-05	0.00382
C22orf19	1.512772	5.546248	7.491439	4.69E-05	0.00382
SLC31A2	1.211022	6.339168	7.486499	4.71E-05	0.00382
CPT2	1.154065	6.64299	7.484252	4.72E-05	0.00382
CDC2	1.467302	7.546642	7.48391	4.72E-05	0.00382
TGOLN2	1.20037	6.332779	7.47839	4.75E-05	0.003827
PLEKHA6	1.423828	5.237043	7.464776	4.82E-05	0.003858
ELF5	1.77998	6.909665	7.456077	4.86E-05	0.003862
RUTBC1	1.359182	9.002787	7.454379	4.87E-05	0.003862

Gene names	logFC	AveExpr	t	P.Value	adj.P.Val
DCK	1.522346	4.637008	7.430345	4.99E-05	0.003932
CDC2L1	1.406028	4.29464	7.409943	5.09E-05	0.003984
CNTNAP2	1.402397	5.974443	7.392718	5.18E-05	0.004035
PDDC1	1.108842	5.094749	7.373093	5.29E-05	0.004104
CDC42EP4	1.707713	8.889899	7.368919	5.31E-05	0.004109
CHD5	1.254157	4.140533	7.355384	5.38E-05	0.004123
CD44	1.39392	5.339362	7.354313	5.39E-05	0.004123
FLJ37587	1.224445	2.10866	7.353687	5.39E-05	0.004123
SLC16A6	1.46207	3.782275	7.336868	5.49E-05	0.004169
LOC648081	3.087991	4.344044	7.330079	5.53E-05	0.004186
PPL	1.571625	5.321612	7.32026	5.58E-05	0.004203
C9orf45	1.561008	4.519568	7.301747	5.69E-05	0.004248
TDP1	1.035549	4.704743	7.300717	5.70E-05	0.004248
RFWD3	1.288811	6.544476	7.295473	5.73E-05	0.004248
MCL1	1.142438	4.664018	7.28394	5.80E-05	0.004274
VDR	1.163312	3.236228	7.283801	5.80E-05	0.004274
ADAM9	1.468933	3.956847	7.277183	5.84E-05	0.004274
SMA4	1.485683	6.137601	7.276826	5.84E-05	0.004274
IFRD1	1.214652	3.457989	7.273462	5.86E-05	0.004274
LOC284998	1.272029	1.845144	7.272501	5.86E-05	0.004274
NUSAP1	1.238963	9.018844	7.26054	5.94E-05	0.004314
STRBP	1.137054	5.777655	7.253369	5.98E-05	0.004334
PPP1R13B	1.203075	6.516927	7.242217	6.05E-05	0.004365
PRRG4	1.486299	3.957547	7.240828	6.06E-05	0.004365
POLE2	1.116774	6.36917	7.232524	6.11E-05	0.004391
ZWINT	1.147828	5.818811	7.226605	6.15E-05	0.004405
GJA1	1.613915	5.259212	7.215678	6.22E-05	0.004436
CEP70	1.542034	5.275748	7.214358	6.23E-05	0.004436
LOC643790	1.339732	5.008213	7.205109	6.29E-05	0.00446

Gene names	logFC	AveExpr	t	P.Value	adj.P.Val
CFLAR	1.683622	4.59681	7.196716	6.34E-05	0.004473
GNPTAB	1.198158	6.771542	7.181078	6.45E-05	0.004528
ALDH3A2	1.163587	6.515251	7.159243	6.60E-05	0.004607
LOC644162	1.584287	4.364937	7.155545	6.62E-05	0.004612
PPP2CB	1.29288	5.708826	7.149945	6.66E-05	0.004626
ACTN1	1.635225	7.850998	7.142169	6.72E-05	0.004651
NFYB	1.176939	4.042196	7.131315	6.79E-05	0.004691
LOC654037	1.311285	3.568107	7.124358	6.84E-05	0.004713
ULK2	1.327192	3.518291	7.116717	6.90E-05	0.004738
TNRC5	1.083716	4.470633	7.113017	6.93E-05	0.004743
SHMT1	1.448556	7.175676	7.106233	6.98E-05	0.004751
GPR137B	1.428587	7.117566	7.096888	7.04E-05	0.004785
GPD1L	1.597755	8.769293	7.070731	7.24E-05	0.004879
STK3	1.127584	6.049689	7.061321	7.31E-05	0.004915
SLC29A2	1.36513	8.316879	7.033568	7.53E-05	0.005021
HDAC11	1.303466	5.092993	7.023174	7.62E-05	0.005063
TRIM22	1.831859	1.765561	7.001096	7.80E-05	0.005147
LY6G5B	1.570617	1.655081	7.000251	7.80E-05	0.005147
LOC648763	1.208073	3.807533	6.988811	7.90E-05	0.005186
NAGK	1.366449	8.206389	6.96814	8.08E-05	0.005246
DDX46	1.680216	5.881681	6.963713	8.11E-05	0.005246
C16orf61	1.173848	9.528509	6.963497	8.12E-05	0.005246
LOC389641	1.365551	4.620577	6.95834	8.16E-05	0.005246
CACYBP	1.275824	6.917886	6.957967	8.16E-05	0.005246
LOC652506	1.227948	5.267535	6.950122	8.23E-05	0.005257
CWF19L1	1.346888	4.534545	6.948963	8.24E-05	0.005257
ADRB2	1.046045	3.043669	6.944976	8.28E-05	0.00526
ANKRD35	1.055973	2.627341	6.94359	8.29E-05	0.00526

Gene names	logFC	AveExpr	t	P.Value	adj.P.Val
C10orf9	1.64677	5.898123	6.935637	8.36E-05	0.005292
PRIM2A	1.290231	3.323622	6.929562	8.42E-05	0.005292
CDKN2C	1.093981	3.377343	6.928448	8.43E-05	0.005292
G6PC	1.37615	2.093475	6.918235	8.52E-05	0.005324
MGAT2	1.059097	4.603676	6.908676	8.61E-05	0.005365
TNPO2	1.422696	7.130425	6.890436	8.78E-05	0.005445
NFIC	1.273748	3.906069	6.890339	8.78E-05	0.005445
ZMAT2	1.128901	7.834425	6.877597	8.90E-05	0.005504
CPEB4	2.938177	4.243796	6.872734	8.95E-05	0.005504
SNAP23	1.365314	4.343783	6.872559	8.95E-05	0.005504
CSNK1A1	1.272574	5.119015	6.870593	8.97E-05	0.005504
IRF1	1.081986	3.807905	6.868393	8.99E-05	0.005504
LFNG	1.641912	4.949446	6.866699	9.01E-05	0.005504
FAM13C1	1.018481	2.525384	6.851101	9.16E-05	0.005584
CCDC64	1.167647	6.649169	6.844074	9.23E-05	0.005613
AKAP10	1.135545	4.185929	6.8337	9.33E-05	0.005649
IRX3	1.514129	10.20833	6.822625	9.45E-05	0.005689
MCCC2	1.230245	4.239475	6.817791	9.50E-05	0.005705
CEP152	1.446565	4.279749	6.815708	9.52E-05	0.005705
CDC2L6	1.445018	5.090917	6.806973	9.61E-05	0.005745
RFC3	1.323836	4.742919	6.798892	9.69E-05	0.005782
KCNJ11	1.050016	3.265539	6.777455	9.92E-05	0.005905
KCNB1	1.618515	4.205399	6.764905	0.000101	0.005972
LOC440093	1.14082	8.191142	6.752941	0.000102	0.006022
ASPHD2	1.094693	3.15188	6.746286	0.000103	0.006037
ARMET	1.221632	9.398527	6.736805	0.000104	0.006078
ACBD7	1.009159	3.608699	6.732127	0.000104	0.006078
NETO2	1.382543	3.311967	6.731622	0.000104	0.006078
POLQ	1.101173	4.849839	6.731153	0.000104	0.006078

Gene names	logFC	AveExpr	t	P.Value	adj.P.Val
ALDOA	1.074386	9.212634	6.729484	0.000105	0.006078
LIN7B	1.230892	4.824204	6.726149	0.000105	0.006086
RBPMS2	1.051624	6.436368	6.711882	0.000107	0.006142
HSPA5	1.145635	6.262515	6.711494	0.000107	0.006142
RAB3B	1.208129	2.608677	6.707376	0.000107	0.006155
CMIP	1.509773	3.79487	6.704086	0.000108	0.006163
CHRNA5	1.281453	6.645268	6.674816	0.000111	0.006301
PPP1R14C	1.718607	1.943461	6.674246	0.000111	0.006301
CCAR1	1.216398	6.593615	6.67172	0.000111	0.006301
PLA2G10	1.122997	3.645908	6.663	0.000112	0.006332
ASB9	1.202737	3.141655	6.636193	0.000116	0.006493
FRK	1.277251	1.495324	6.612292	0.000119	0.006619
PBEF1	1.186321	3.672297	6.61084	0.000119	0.006619
AKR1C3	1.606508	3.415733	6.597365	0.000121	0.006704
EPB41L3	1.004823	5.136785	6.59198	0.000122	0.006724
FLJ35934	1.150756	3.711561	6.584786	0.000123	0.006746
EXT2	1.077306	4.271591	6.576557	0.000124	0.006756
KIF5B	1.05704	4.803647	6.569078	0.000125	0.006793
MANBA	1.10328	6.31373	6.567444	0.000125	0.006793
ARID1A	2.036033	4.382748	6.565777	0.000125	0.006793
MASA	1.050444	6.072095	6.563069	0.000126	0.006799
SNX26	1.107096	6.14544	6.539208	0.000129	0.006967
DKFZp686L1814	1.410259	3.988974	6.527385	0.000131	0.00703
LRRFIP2	1.283931	5.404215	6.520465	0.000132	0.007069
FBXO5	1.875536	6.241063	6.509853	0.000133	0.007123
C1orf168	1.187845	3.397852	6.493122	0.000136	0.007195
CDKN1C	1.273787	2.387271	6.46801	0.00014	0.007356
NUP62	1.170389	9.347663	6.464623	0.00014	0.007369

Gene names	logFC	AveExpr	t	P.Value	adj.P.Val
C20orf14	1.227714	4.861334	6.460555	0.000141	0.007387
LPIN3	1.112594	3.882629	6.45618	0.000142	0.007408
TSPAN33	1.191587	5.637627	6.442419	0.000144	0.007467
TSNAX	2.118876	5.529456	6.440277	0.000144	0.007467
ZNF503	1.279674	2.943905	6.439907	0.000144	0.007467
RRAGC	1.155286	5.061591	6.435306	0.000145	0.007489
C10orf113	1.327786	2.376586	6.42949	0.000146	0.007509
LRP5L	1.25498	4.039559	6.422423	0.000147	0.007554
DSCR1	1.577733	8.911121	6.417995	0.000148	0.007572
CTNNB1	1.542383	3.106096	6.413358	0.000149	0.007572
TRPV4	1.128883	3.412165	6.413255	0.000149	0.007572
HIPK2	1.181544	5.295003	6.412548	0.000149	0.007572
ZNF689	1.835496	5.496529	6.409476	0.000149	0.007572
LOC284023	1.318438	4.313609	6.403782	0.00015	0.007606
TMPO	1.837889	3.65869	6.398577	0.000151	0.007636
LOC653108	1.166388	4.144642	6.393652	0.000152	0.007663
PPP1CB	1.261936	5.811277	6.383351	0.000154	0.007738
DBI	1.220937	11.70331	6.361072	0.000158	0.007904
DPYSL3	1.16562	3.639573	6.355182	0.000159	0.007926
ANXA2	1.263909	7.523015	6.350084	0.00016	0.00794
NUCKS1	1.150934	4.542497	6.335178	0.000163	0.008007
ARRB1	1.203724	4.894086	6.334576	0.000163	0.008007
GADD45B	1.03021	5.303222	6.33415	0.000163	0.008007
MUC1	1.382262	11.10672	6.329374	0.000164	0.008019
UGT2B28	1.271782	1.992074	6.325584	0.000164	0.008022
PRSS16	1.097051	4.83297	6.315477	0.000166	0.0081
EAF2	1.540479	3.02913	6.311738	0.000167	0.008103
FASN	1.242785	11.45353	6.30858	0.000168	0.008117
GMCL1	1.173374	4.306139	6.304403	0.000168	0.008137

Gene names	logFC	AveExpr	t	P.Value	adj.P.Val
GNE	1.833965	5.110068	6.295945	0.00017	0.008166
GREB1	1.623446	4.958656	6.29282	0.000171	0.008166
FAM36A	1.011928	6.510685	6.292592	0.000171	0.008166
LOC643287	1.32867	4.925702	6.289919	0.000171	0.008166
LOC651348	1.774295	5.019179	6.27019	0.000175	0.008274
TRUB1	1.604647	2.491446	6.270131	0.000175	0.008274
MTCH2	1.215555	4.756038	6.255811	0.000178	0.00838
TA-PP2C	1.48343	4.005584	6.252105	0.000179	0.0084
ARL6IP6	1.905483	5.294567	6.24577	0.00018	0.008446
LOC649095	1.51686	2.191759	6.241896	0.000181	0.008464
IQGAP3	1.207876	7.688189	6.240654	0.000181	0.008464
TUBA2	1.676006	1.751212	6.230493	0.000183	0.00854
LOC651559	1.220356	8.467733	6.228736	0.000184	0.00854
C9orf40	1.017784	4.533375	6.225754	0.000184	0.008547
STXBP3	1.603506	4.759936	6.220203	0.000185	0.008554
LOC134147	1.045077	7.115126	6.21905	0.000186	0.008554
LOC255783	1.410054	7.189652	6.218726	0.000186	0.008554
PSPH	1.281846	5.538378	6.202569	0.000189	0.008699
ARHGEF4	1.648348	1.874453	6.197953	0.00019	0.008714
KCNG3	1.156396	1.615369	6.19418	0.000191	0.008734
JARID2	1.3704	4.845266	6.183695	0.000194	0.008792
SFRS9	1.166276	11.1863	6.18281	0.000194	0.008792
IFRG15	1.335065	2.478551	6.176591	0.000195	0.008835
ELOVL5	1.77467	7.401179	6.168705	0.000197	0.008885
HP1BP3	1.041681	3.541816	6.157938	0.000199	0.008943
MRAS	1.134309	3.315585	6.157376	0.0002	0.008943
FLJ25422	1.62967	3.274842	6.156973	0.0002	0.008943
GP1BB	1.458328	7.088188	6.14876	0.000202	0.00896

Gene names	logFC	AveExpr	t	P.Value	adj.P.Val
PPP1R3C	2.23052	3.324875	6.147489	0.000202	0.00896
LRRC50	1.492014	3.338297	6.132753	0.000205	0.009085
NEIL3	1.077328	4.150759	6.106968	0.000212	0.009265
IDH1	1.139221	4.660148	6.093928	0.000215	0.009358
CDCA5	1.115727	8.829617	6.07935	0.000219	0.009487
FAM46B	1.04229	7.620825	6.076411	0.000219	0.009503
MT1F	1.102197	4.311452	6.065951	0.000222	0.009511
NBPF14	1.493266	3.583117	6.065944	0.000222	0.009511
ITGA2B	1.360653	4.668349	6.063379	0.000223	0.009518
SLC2A3	1.389967	1.98503	6.056174	0.000225	0.009564
FKSG44	1.227481	7.395626	6.054932	0.000225	0.009564
F2R	1.514523	1.879248	6.051593	0.000226	0.009585
LOC402571	1.298711	1.235396	6.047332	0.000227	0.009605
LOC645074	1.192255	1.840968	6.043378	0.000228	0.009629
ACSL3	1.797644	3.718645	6.039121	0.000229	0.009649
ARL6IP2	1.066614	4.02017	6.038007	0.00023	0.009649
CLDN15	1.158243	3.781956	6.022977	0.000234	0.009749
CLDN12	1.119021	5.528813	6.015858	0.000236	0.009799
ABHD3	1.488699	3.835767	5.995453	0.000241	0.009939
PPP3CA	1.02781	4.601573	5.967882	0.000249	0.010234
DNAJC9	1.103136	8.029402	5.96665	0.00025	0.010234
THRAP6	1.041179	5.439828	5.956436	0.000253	0.010323
PTPLAD1	1.787057	7.0021	5.945359	0.000256	0.010361
SAT	1.031458	8.47434	5.941002	0.000257	0.010373
MGC16597	1.186456	3.91217	5.93869	0.000258	0.010373
ACOT2	1.09048	5.9899	5.937667	0.000259	0.010373
LOC643196	1.274164	1.588488	5.934342	0.00026	0.010397
NEK7	1.395989	2.42236	5.930699	0.000261	0.010416
PACSIN2	1.274882	7.145247	5.927487	0.000262	0.010416

Gene names	logFC	AveExpr	t	P.Value	adj.P.Val
VCL	1.012821	7.814517	5.916631	0.000265	0.010535
ATP6V0E	1.169982	7.954077	5.913326	0.000266	0.010544
SLC15A2	1.021205	2.390407	5.901221	0.00027	0.010641
AQP3	1.446752	2.516334	5.895127	0.000272	0.010641
FNBP1L	1.236351	6.359772	5.89378	0.000272	0.010641
DEFB32	1.015643	2.157719	5.88011	0.000277	0.010752
ACTB	1.210215	11.59845	5.880007	0.000277	0.010752
LOC648695	2.090024	4.716913	5.878048	0.000278	0.01076
LOC648366	2.746189	2.969791	5.87465	0.000279	0.010787
ADFP	1.068013	6.094526	5.870415	0.00028	0.010826
HPCAL1	1.133107	6.296203	5.864274	0.000282	0.010856
EFCAB3	1.412099	2.769274	5.861731	0.000283	0.010862
LOC642576	1.323264	2.30475	5.852452	0.000286	0.010913
GNB1	1.253058	9.854116	5.850963	0.000287	0.010913
HIATL2	1.447221	3.708985	5.839258	0.000291	0.010958
TMEM20	1.216545	4.177918	5.836726	0.000292	0.010958
AMIGO3	1.193738	1.546293	5.836282	0.000292	0.010958
GBA	1.005758	7.445593	5.822578	0.000297	0.011023
C1orf155	1.15358	5.164411	5.818387	0.000298	0.011023
PIB5PA	1.081379	5.002504	5.817871	0.000299	0.011023
C5	1.278681	4.9944	5.817847	0.000299	0.011023
DENND2C	1.265632	3.402937	5.812366	0.000301	0.01108
C12orf58	1.135118	3.748504	5.801934	0.000304	0.011204
FAM104A	1.055916	6.066361	5.786617	0.00031	0.011364
LOC651316	1.531671	2.328706	5.767287	0.000317	0.011537
SLC36A2	1.129126	1.642285	5.760677	0.00032	0.011575
SAFB2	1.204197	5.430585	5.760102	0.00032	0.011575
MYBPH	1.168661	2.600536	5.759602	0.00032	0.011575

Gene names	logFC	AveExpr	t	P.Value	adj.P.Val
STAT3	1.319575	5.394948	5.75609	0.000322	0.011606
DCUN1D3	1.018243	4.731879	5.72518	0.000334	0.011965
EFEMP1	1.402141	6.541472	5.715093	0.000338	0.012092
PNPO	1.086415	8.740661	5.714247	0.000339	0.012092
TCF19	1.069661	3.975189	5.699065	0.000345	0.012248
PRPS2	1.024619	4.478778	5.694486	0.000347	0.0123
EDN2	1.069232	2.563329	5.678006	0.000354	0.012498
MMACHC	1.418383	4.7535	5.658686	0.000362	0.012697
LCE2A	1.258732	1.884195	5.6573	0.000363	0.012697
SLC25A17	1.023279	3.708062	5.639243	0.000371	0.01289
REEP3	1.16633	3.152114	5.634758	0.000373	0.012943
ARL2BP	1.048528	7.465737	5.632774	0.000374	0.012949
FLJ22313	1.307611	3.651586	5.631038	0.000375	0.012949
CENPB	1.145747	8.866305	5.611879	0.000384	0.013222
STK24	1.301354	7.345043	5.608331	0.000386	0.013262
SLC6A6	1.181875	2.895619	5.590904	0.000394	0.01346
SLC35E3	1.121126	7.253188	5.588692	0.000395	0.013465
UBE2G1	1.178822	5.102404	5.588337	0.000395	0.013465
TERF1	1.387521	4.571298	5.573487	0.000403	0.013657
FLJ40629	1.397722	5.843555	5.570383	0.000404	0.013657
HIST1H2BF	1.137592	2.306974	5.563838	0.000408	0.013716
LOC153561	1.517132	5.532294	5.558022	0.000411	0.013794
KIAA0232	1.03525	3.709449	5.553851	0.000413	0.013847
IMAA	1.2658	2.147097	5.549665	0.000415	0.013901
POLS	1.298301	5.609563	5.54723	0.000416	0.013924
FKSG14	1.198084	4.921758	5.54384	0.000418	0.013928
CDC42EP3	1.489102	2.635686	5.534429	0.000423	0.013998
FANCD2	1.074592	3.978084	5.524804	0.000428	0.014097
TIRAP	1.09834	3.752885	5.524541	0.000428	0.014097

Gene names	logFC	AveExpr	t	P.Value	adj.P.Val
HADHSC	1.004398	8.739278	5.518666	0.000431	0.014145
SFXN1	1.009569	6.092337	5.513348	0.000434	0.014208
HIST1H4C	1.053698	12.58641	5.511111	0.000435	0.014222
SLC35A3	1.29894	6.149959	5.506711	0.000438	0.014265
RAB1A	1.110034	6.483577	5.498934	0.000442	0.014329
UBE2A	1.325039	1.279677	5.492785	0.000445	0.014403
KNTC1	1.136229	6.777858	5.485556	0.000449	0.014479
NEK8	1.099702	4.176122	5.48518	0.00045	0.014479
SNTB2	1.090004	3.920985	5.482586	0.000451	0.014494
GRHL2	1.941328	4.916444	5.478386	0.000453	0.014524
CALM2	1.057217	10.70919	5.477866	0.000454	0.014524
KIAA0355	1.168077	4.809843	5.475467	0.000455	0.014531
HMMR	1.304616	7.689492	5.464543	0.000461	0.014696
LOC652274	1.247381	1.559687	5.442775	0.000474	0.014938
SEPP1	1.526459	6.713076	5.436267	0.000478	0.014943
GPR153	1.361381	2.174889	5.412568	0.000493	0.015205
LOC649536	1.283308	2.137806	5.405941	0.000497	0.015276
PCAF	1.054073	4.985714	5.404992	0.000497	0.015276
RHOQ	1.128347	5.925996	5.394938	0.000504	0.015411
ALS2CR15	1.221812	2.549504	5.393185	0.000505	0.015411
UTP14C	1.09291	4.805833	5.38233	0.000512	0.015586
ACYP1	1.121475	5.971866	5.374073	0.000517	0.015683
CBFA2T3	1.08581	4.065055	5.369187	0.000521	0.015742
LOC653650	2.192189	3.01388	5.358676	0.000528	0.015884
KLF15	1.122526	2.704757	5.35469	0.00053	0.015884
SV2B	1.498616	1.861189	5.354036	0.000531	0.015884
CPD	1.081286	7.106242	5.352218	0.000532	0.015884
CS	1.097957	9.095588	5.351325	0.000532	0.015884

Gene names	logFC	AveExpr	t	P.Value	adj.P.Val
ARHGAP26	1.424374	1.859157	5.337484	0.000542	0.016051
TMEM41B	1.032759	6.333732	5.333391	0.000545	0.016097
FAIM	1.118226	3.708457	5.318301	0.000555	0.016275
LDLRAD3	1.613647	4.816042	5.315202	0.000558	0.016319
SH3BP2	1.002634	1.962327	5.305197	0.000565	0.016435
HFE	1.222585	2.46966	5.299668	0.000569	0.01646
LOC643367	1.345484	5.501673	5.298671	0.000569	0.01646
SPFH1	1.111001	4.495068	5.29585	0.000572	0.016498
ZFYVE21	1.14925	8.637189	5.290164	0.000576	0.016542
CAP2	1.21932	8.617301	5.272129	0.000589	0.016756
SRP9	1.355139	4.708158	5.264917	0.000595	0.016835
MFSD7	1.019526	4.136653	5.263094	0.000596	0.016841
TESC	1.300359	2.851437	5.262017	0.000597	0.016841
USP22	1.017676	2.379282	5.254215	0.000603	0.016915
LOC652726	1.130784	3.434104	5.244291	0.000611	0.017055
HSPA2	1.536417	3.211007	5.239163	0.000615	0.017055
EIF5	1.872914	3.845135	5.235233	0.000618	0.017055
EMP1	1.273346	2.317733	5.234012	0.000619	0.017055
SHRM	1.323384	4.399434	5.230349	0.000622	0.017082
ANKRD20A2	1.539166	2.303376	5.229906	0.000622	0.017082
LOC653712	1.285761	2.19293	5.222433	0.000628	0.017169
LASS6	1.462826	7.329299	5.222001	0.000628	0.017169
IMPDH1	1.37237	6.546645	5.220881	0.000629	0.017175
LOC196752	1.010642	4.786563	5.219172	0.000631	0.017194
PDPK1	1.829784	3.784712	5.217664	0.000632	0.017209
DJ122O8.2	1.292084	5.629604	5.213743	0.000635	0.017259
PDGFC	1.237499	3.737018	5.195048	0.000651	0.017459
ECT2	1.557681	5.315436	5.194143	0.000652	0.017459
EIF1AX	1.373207	6.24246	5.192572	0.000653	0.017459

Gene names	logFC	AveExpr	t	P.Value	adj.P.Val
PLEKHB2	1.248376	3.5618	5.192312	0.000653	0.017459
RBBP5	1.444468	4.986945	5.187958	0.000657	0.017502
ACOX1	1.40291	4.890404	5.183747	0.00066	0.017538
TNFAIP8L3	1.191997	3.034	5.183223	0.000661	0.017538
LOC644330	1.174754	5.04871	5.183093	0.000661	0.017538
ANKRD38	1.135543	1.345469	5.177359	0.000666	0.017594
FJX1	1.431509	8.798068	5.168041	0.000674	0.017696
SCML1	1.112412	4.14003	5.163931	0.000678	0.017734
IGF2R	1.215772	7.722157	5.161276	0.00068	0.017759
LOC389816	1.243561	7.418257	5.152933	0.000687	0.017896
LOC653357	1.369493	2.113683	5.149016	0.000691	0.01795
SPTBN1	1.673032	3.325926	5.144916	0.000695	0.018
ERBB2IP	1.102204	3.069955	5.141594	0.000698	0.018049
SCGB1D2	1.119206	2.47118	5.138336	0.0007	0.018089
MT1X	1.508933	5.397395	5.133123	0.000705	0.018193
GPR89A	1.607565	5.08858	5.123812	0.000714	0.018359
GULP1	1.486383	2.414639	5.119285	0.000718	0.018443
GSTM3	1.108369	5.760798	5.109603	0.000727	0.018569
C17orf55	1.064055	3.145723	5.108644	0.000728	0.018573
PROK2	1.638842	2.202391	5.099761	0.000737	0.018676
E2F7	1.308926	4.795906	5.096509	0.00074	0.018717
SOD2	1.129993	5.063614	5.094194	0.000742	0.018755
KIAA1671	1.270225	5.704461	5.092638	0.000744	0.018774
LOC641825	1.154407	1.434282	5.087717	0.000748	0.018858
ADHFE1	1.007797	5.002508	5.086156	0.00075	0.018859
HNRPH1	2.401707	8.446034	5.078982	0.000757	0.018941
RRP22	1.374199	2.781726	5.065531	0.00077	0.019117
H3F3A	1.080043	12.31177	5.064204	0.000772	0.01912

Gene names	logFC	AveExpr	t	P.Value	adj.P.Val
LOC652640	1.390036	1.82572	5.059455	0.000777	0.019192
TRA2A	1.453461	5.366969	5.031499	0.000806	0.019592
GUSBL1	1.354391	5.679168	5.0082	0.000831	0.020009
LOC654174	2.211955	3.034428	5.002187	0.000838	0.020111
PHACTR4	1.439126	7.006731	4.992867	0.000848	0.020263
SSBP2	1.176157	7.269814	4.990926	0.00085	0.020296
KIAA0251	1.096123	2.599776	4.985105	0.000857	0.020418
IRX2	1.058923	6.032372	4.982194	0.00086	0.020473
RCP9	1.256487	3.154349	4.975628	0.000867	0.020613
PLEKHF2	1.476921	5.80591	4.966069	0.000879	0.020718
NFIA	1.416587	5.636498	4.964644	0.00088	0.020738
HNRPD	1.061927	9.223028	4.957992	0.000888	0.020804
PNLIP	1.030481	2.002775	4.947284	0.000901	0.021003
FLJ23191	1.069968	2.137347	4.946608	0.000902	0.021003
GALNT6	1.069418	4.064449	4.936866	0.000913	0.021229
ALDH9A1	1.153216	10.24132	4.932687	0.000918	0.021297
NBEA	1.633434	3.311246	4.922439	0.000931	0.02151
DEPDC1B	1.112105	3.620434	4.913505	0.000942	0.021708
INCENP	1.502704	2.332732	4.910142	0.000946	0.021785
VPS35	1.065895	7.657678	4.904407	0.000954	0.021933
TDRD10	1.013649	1.594221	4.895092	0.000966	0.022025
KIAA0889	1.112448	4.030815	4.866992	0.001003	0.022559
EMP2	1.00169	2.657222	4.864684	0.001006	0.022559
LOC650141	1.644716	2.568429	4.863388	0.001008	0.022569
HSPCAL3	1.028508	1.671201	4.855839	0.001018	0.022738
MTSS1	1.260661	2.95104	4.853733	0.001021	0.022782
LOC653767	1.073848	2.316005	4.846748	0.00103	0.022915
C18orf45	1.198145	4.601472	4.837401	0.001043	0.023122
IMPAD1	1.216988	5.050702	4.836003	0.001045	0.023145

Gene names	logFC	AveExpr	t	P.Value	adj.P.Val
LOC652773	1.348925	4.613226	4.829896	0.001054	0.023295
LOC642984	1.144487	1.450486	4.823163	0.001064	0.02341
RAB2B	1.345459	5.73151	4.818027	0.001071	0.023464
LOC644614	1.670015	2.535946	4.811079	0.001081	0.023581
M6PR	1.116327	4.694995	4.810076	0.001083	0.023589
LOC440934	1.074229	1.890802	4.796861	0.001102	0.023922
LOC644422	2.713947	5.884776	4.792155	0.001109	0.023952
CYR61	1.02473	6.167702	4.787385	0.001116	0.023979
NCOA2	1.266854	2.400419	4.787169	0.001117	0.023979
CANX	1.121103	4.666443	4.777151	0.001132	0.024258
ZNF217	1.998319	6.169332	4.760572	0.001158	0.024549
MGC40579	1.046184	4.805746	4.759572	0.001159	0.024549
NEDD4L	1.020443	4.140804	4.75638	0.001164	0.024549
WTAP	1.055511	3.719422	4.73506	0.001198	0.025047
TBC1D8	1.058948	2.91762	4.722899	0.001218	0.025269
KIAA1794	1.066432	5.660323	4.722488	0.001219	0.025269
ZMYND11	1.006496	6.114682	4.720245	0.001223	0.025325
LOC645719	1.428138	4.969195	4.712112	0.001236	0.025411
TBL1X	1.248896	5.818606	4.711112	0.001238	0.025411
LOC650520	1.051427	0.777999	4.70846	0.001243	0.025482
LOC644063	2.01839	6.518499	4.703171	0.001252	0.02562
LOC400987	1.675398	2.553358	4.692617	0.00127	0.025891
CDCA1	1.006295	3.678125	4.684221	0.001284	0.026102
RIOK3	1.299547	4.766527	4.683598	0.001286	0.026102
LMO7	1.241511	2.232058	4.679542	0.001293	0.026102
MDM4	1.708055	2.805509	4.678062	0.001295	0.026113
KIF14	1.028852	5.204946	4.651576	0.001343	0.026439
XBP1	1.057019	9.818643	4.650081	0.001346	0.026473

Gene names	logFC	AveExpr	t	P.Value	adj.P.Val
LOC652612	1.490164	2.055006	4.640909	0.001363	0.026662
MYO5C	1.3333	8.034519	4.633247	0.001378	0.02686
SCD	1.233838	10.93776	4.630982	0.001382	0.026902
U2AF2	1.221371	7.021749	4.629802	0.001384	0.026909
CCNI	1.311594	9.695689	4.62966	0.001384	0.026909
TMEM16F	1.581593	5.926331	4.628298	0.001387	0.026938
YWHAB	1.404206	9.353787	4.618843	0.001405	0.027207
SLC39A8	1.109503	5.384905	4.600147	0.001442	0.027618
PFN2	1.168277	6.405211	4.593888	0.001454	0.027794
RARG	1.103597	2.29423	4.590137	0.001462	0.027853
CCNE2	1.345639	5.128727	4.585487	0.001471	0.027952
C14orf138	1.00052	4.00772	4.583071	0.001476	0.027995
RCC2	1.108806	8.493499	4.577191	0.001488	0.028108
CD82	1.223422	5.685179	4.571415	0.0015	0.028251
NUDT19	1.425881	1.695376	4.566033	0.001511	0.028254
LOC619190	1.73464	2.080944	4.565752	0.001512	0.028254
PIGA	1.133516	4.754287	4.556002	0.001533	0.028421
IL6ST	1.500911	2.476616	4.552075	0.001541	0.028454
PRICKLE1	1.483173	2.354787	4.546795	0.001552	0.028596
FLJ38101	1.318319	4.577999	4.544225	0.001558	0.028656
PLEKHF1	1.539235	5.812493	4.542191	0.001562	0.028716
PICALM	1.334671	3.464813	4.510914	0.001632	0.02958
C9orf88	1.342711	9.073888	4.509896	0.001634	0.0296
COL7A1	1.029536	4.119859	4.508514	0.001637	0.029636
SLC39A9	1.031033	4.053772	4.505402	0.001644	0.029743
LOC643909	1.145479	2.355706	4.50431	0.001647	0.029745
GALNACT-2	1.018154	3.331173	4.492491	0.001674	0.030045
WHDC1	1.218434	2.608075	4.474486	0.001717	0.030402
LOC652840	1.361861	1.91325	4.47148	0.001724	0.030459

Gene names	logFC	AveExpr	t	P.Value	adj.P.Val
LOC441425	1.310159	2.269266	4.465563	0.001739	0.030583
FLJ36874	1.129293	7.255431	4.449354	0.001778	0.030873
ATXN3	1.180748	1.945348	4.440189	0.001801	0.031099
GFPT1	1.088739	5.11071	4.439407	0.001803	0.031101
LOC653281	1.191587	1.985819	4.428967	0.00183	0.031398
LOC648434	1.068733	2.661354	4.424339	0.001842	0.031571
LOC643300	1.245331	3.583964	4.422413	0.001847	0.031624
CAMKK1	1.085995	2.025249	4.408809	0.001883	0.032082
ABCB10	1.057992	5.546383	4.407482	0.001886	0.032098
RSPRY1	1.203571	6.345636	4.40543	0.001892	0.032147
LOC653133	1.622311	3.256214	4.403934	0.001896	0.032193
ERO1LB	1.355282	2.513529	4.39708	0.001914	0.032346
SEC23A	1.25779	3.173171	4.388281	0.001938	0.032645
CAPN7	1.132275	4.319449	4.382738	0.001953	0.032813
GPC4	1.096636	3.126804	4.378888	0.001964	0.032904
LOC642817	1.692256	9.212915	4.375248	0.001974	0.032985
LOC643668	1.286591	9.17732	4.367474	0.001996	0.033217
EGF	1.201536	2.903976	4.364633	0.002004	0.033284
SNRPD3	1.054377	4.586484	4.357915	0.002023	0.033446
NOMO3	2.15297	2.541066	4.356735	0.002026	0.03348
LOC650766	1.373201	2.04581	4.350309	0.002045	0.033737
AFF4	2.051902	4.706384	4.345676	0.002058	0.033863
FOXO1A	1.335468	3.100747	4.328537	0.002109	0.034481
SLC39A6	1.208672	4.583344	4.321963	0.002129	0.034646
TIA1	1.127644	4.298883	4.314757	0.002151	0.03489
LOC653080	1.455518	4.292326	4.307309	0.002174	0.035114
HMGB2	1.192318	6.612317	4.301836	0.002191	0.035286
TMTC3	1.578307	2.755937	4.297102	0.002206	0.03541

Gene names	logFC	AveExpr	t	P.Value	adj.P.Val
CDK2	1.437619	6.216565	4.294502	0.002214	0.035473
INSIG1	1.270387	5.519868	4.291274	0.002224	0.035523
LOC115648	1.168377	2.309108	4.287393	0.002236	0.035651
TKTL1	1.025607	1.171986	4.275957	0.002273	0.036008
ANKRD20A3	1.014003	3.369208	4.270418	0.002291	0.036157
TNRC6C	1.238834	2.298101	4.269636	0.002294	0.036174
TMEM107	1.327057	2.69503	4.264228	0.002312	0.036357
ZNF69	1.145385	4.844808	4.257632	0.002333	0.036601
RING1	1.16105	8.553493	4.257469	0.002334	0.036601
SLC4A7	1.745807	2.950774	4.213119	0.002487	0.038036
HIST1H3B	1.242116	1.486399	4.208062	0.002505	0.038257
LOC649722	1.018621	1.863036	4.202632	0.002525	0.038463
MT2A	1.630596	8.453193	4.199571	0.002536	0.038586
FXR1	1.469061	6.602724	4.18555	0.002588	0.039063
CUEDC1	1.091154	8.27413	4.176078	0.002623	0.039481
TM2D1	1.050227	5.087264	4.174214	0.002631	0.039529
CKAP2	1.070061	5.328482	4.163457	0.002672	0.039895
VPS53	1.131881	1.844767	4.153344	0.002711	0.040265
LOC647603	1.168439	1.538158	4.152854	0.002713	0.04027
LOC51315	1.243849	4.11349	4.142058	0.002756	0.04059
GTF2I	1.116927	2.793734	4.140626	0.002761	0.040627
GNB4	1.071365	4.063381	4.129912	0.002805	0.04094
QSCN6	1.297643	2.291921	4.114238	0.002869	0.041301
C14orf169	1.353143	5.175959	4.113374	0.002873	0.041301
LOC641807	1.238253	2.290547	4.112952	0.002874	0.041301
FEN1	1.11797	6.715347	4.108401	0.002893	0.041471
LOC644037	1.539678	10.0894	4.093371	0.002957	0.041805
SPCS3	1.148737	3.051072	4.084313	0.002997	0.042039
LOC440944	1.239544	2.008981	4.080905	0.003011	0.042161

Gene names	logFC	AveExpr	t	P.Value	adj.P.Val
LOC650257	1.194049	2.326975	4.079538	0.003017	0.042188
FBXO28	1.138105	5.463717	4.059345	0.003108	0.042849
C10orf46	1.017416	2.850713	4.051572	0.003143	0.043094
RAB11A	1.041005	9.835212	4.042298	0.003186	0.043452
YAP1	1.220848	7.819845	4.034342	0.003223	0.043625
UGP2	1.042518	4.212002	4.032246	0.003233	0.043661
LOC647743	1.233664	5.198209	4.029305	0.003247	0.043825
SUZ12	1.150145	8.89266	4.015017	0.003316	0.044367
C2orf13	1.062803	1.636961	4.013584	0.003323	0.044367
APAF1	1.456978	5.319884	4.012025	0.00333	0.044435
HMG1L1	1.028352	4.964825	4.011795	0.003331	0.044435
ACTR2	1.491019	7.994787	4.01145	0.003333	0.044435
DCUN1D1	1.57556	4.305046	4.003753	0.003371	0.044632
LOC644823	1.674451	2.384896	4.003293	0.003373	0.044636
DKFZP434A0131	1.753669	5.953905	3.996596	0.003406	0.044887
INHBB	1.070834	6.628282	3.988183	0.003449	0.04523
LOC651518	1.158184	1.509426	3.974292	0.00352	0.045734
LOC644799	1.283259	6.927729	3.971388	0.003535	0.045808
SAPS2	1.138366	3.856581	3.963313	0.003577	0.045997
ADARB1	1.100211	3.315821	3.960457	0.003592	0.046097
TCEA1	1.627452	2.943476	3.959477	0.003598	0.046123
SP6	1.01281	2.575062	3.952474	0.003635	0.046357
KCNN4	1.015963	1.235124	3.949621	0.00365	0.04648
LRIG3	1.142701	6.422736	3.945953	0.00367	0.046544
CYP4A11	1.031333	2.265245	3.94533	0.003673	0.046545
GPHN	1.186812	3.747644	3.936142	0.003723	0.046937
LOC641913	1.108762	2.364668	3.924722	0.003787	0.047402
TMED2	1.199433	8.995805	3.922991	0.003797	0.047437

Gene names	logFC	AveExpr	t	P.Value	adj.P.Val
HOOK1	1.053355	5.295477	3.918493	0.003822	0.047507
APBB2	1.012226	3.036122	3.913198	0.003852	0.047737
HNRPA3	1.436006	6.3208	3.910715	0.003866	0.047795
KLF6	1.114607	6.79944	3.899032	0.003934	0.048413
NEK9	1.058075	1.682885	3.880689	0.004042	0.049345

Table S4. The 644 DEGs in the MPA group.

Gene names	logFC	AveExpr	t	P.Value	adj.P.Val
MAFB	5.490728	6.252615	37.16153	1.02E-10	1.28E-06
LOC128977	4.927725	1.22194	34.01542	2.16E-10	1.80E-06
S100P	6.5264	7.275375	30.59446	5.30E-10	3.09E-06
LOC388610	4.390265	5.410631	28.61797	9.30E-10	3.88E-06
NDRG1	4.559706	5.724406	27.41499	1.34E-09	4.77E-06
SLC25A18	3.763501	5.463562	26.29795	1.90E-09	5.92E-06
PHACTR3	3.700196	3.33733	22.59328	6.79E-09	1.70E-05
FKBP5	4.530078	7.8826	22.03026	8.39E-09	1.78E-05
RBP2	3.559631	4.458299	21.80315	9.15E-09	1.78E-05
ZBTB16	3.687409	3.636325	21.77659	9.24E-09	1.78E-05
SGK	4.416435	4.831272	20.28826	1.67E-08	2.98E-05
ATP1A1	3.607756	8.042719	19.95829	1.92E-08	3.19E-05
TIPARP	3.453032	6.675865	19.78822	2.06E-08	3.21E-05
PNMT	3.54084	3.872478	19.35138	2.48E-08	3.64E-05
FBXO32	2.671739	7.031538	19.03826	2.84E-08	3.93E-05
SCRG1	3.486085	3.53526	18.86977	3.06E-08	3.93E-05
SLC26A3	2.877197	2.882223	18.80869	3.14E-08	3.93E-05
STMN3	2.707067	6.125723	18.47659	3.64E-08	4.34E-05
LOC93622	2.915334	7.867644	18.2411	4.05E-08	4.61E-05

Gene names	logFC	AveExpr	t	P.Value	adj.P.Val
SLPI	3.13309	4.440089	17.72777	5.14E-08	5.13E-05
NFKBIA	3.169766	9.439643	17.65299	5.32E-08	5.13E-05
FLJ41603	2.870247	5.708278	17.64811	5.34E-08	5.13E-05
GPSM2	2.945512	6.708824	16.94554	7.47E-08	6.46E-05
CLDN8	3.839648	5.607741	16.91412	7.59E-08	6.46E-05
TSC22D1	2.370314	6.762118	16.86949	7.76E-08	6.46E-05
ATAD4	2.80987	7.364334	16.0587	1.17E-07	9.20E-05
ADAMTS8	3.156658	4.451174	16.03875	1.18E-07	9.20E-05
KCNG1	2.674714	4.331473	15.87619	1.28E-07	9.71E-05
HSD11B2	2.495074	4.22313	15.79507	1.34E-07	9.83E-05
ACSL1	2.944628	5.793737	15.73398	1.38E-07	9.86E-05
TM4SF11	2.260782	4.450549	15.57892	1.50E-07	0.000104
DDIT4	2.179669	6.849104	14.95406	2.10E-07	0.000142
DSCR6	2.442383	6.770762	14.63838	2.50E-07	0.000161
PXMP4	2.642666	7.208763	14.60478	2.55E-07	0.000161
STEAP1	2.105053	4.036012	14.58837	2.57E-07	0.000161
ALPL	3.133786	3.243757	14.25876	3.10E-07	0.000189
MLLT7	2.191112	4.322229	14.18375	3.24E-07	0.000192
SERPINA3	2.457169	4.685509	14.1525	3.30E-07	0.000192
RDH16	2.086968	4.294635	13.81817	4.01E-07	0.00022
CEBPD	2.39622	6.860591	13.80151	4.05E-07	0.00022
TSC22D3	2.145122	3.654023	13.69868	4.31E-07	0.000224
CYP27A1	1.979407	3.841694	13.58647	4.61E-07	0.000235
CA4	1.951775	2.626936	13.52772	4.77E-07	0.000235
SERPINA5	2.541382	2.465441	13.51712	4.80E-07	0.000235
SGPP2	1.96069	2.053137	13.41948	5.10E-07	0.000245
THRSP	2.262724	2.304015	13.12026	6.13E-07	0.000289
P4HA2	2.223479	7.290921	13.06133	6.35E-07	0.000294

Gene names	logFC	AveExpr	t	P.Value	adj.P.Val
FLJ20366	2.295264	4.608057	13.02125	6.52E-07	0.000296
PACSIN1	2.181349	6.955549	12.96715	6.74E-07	0.000301
STAT5A	2.36882	4.336803	12.92436	6.92E-07	0.000304
CRIM1	2.69013	4.784505	12.76087	7.68E-07	0.000327
SEC14L2	2.062928	4.019464	12.75183	7.72E-07	0.000327
CG018	1.847115	2.006399	12.52792	8.92E-07	0.00036
KLF9	1.789568	4.379575	12.51199	9.01E-07	0.00036
RASGEF1A	1.723861	5.278111	12.48485	9.17E-07	0.00036
FAM105A	1.75982	3.88212	12.48194	9.19E-07	0.00036
MYO10	1.956256	6.745786	12.47762	9.21E-07	0.00036
IL20RA	1.801282	4.409873	12.42137	9.56E-07	0.000366
ATP1B1	4.461091	7.91195	12.38871	9.76E-07	0.000366
HIG2	1.863857	7.622834	12.38011	9.82E-07	0.000366
C6orf85	3.074585	7.57253	12.33383	1.01E-06	0.00037
FLJ14503	1.82626	2.281358	12.30557	1.03E-06	0.00037
PDK4	1.963505	2.488197	12.25463	1.07E-06	0.00037
BNIP3	2.097047	8.241057	12.11137	1.17E-06	0.000402
AKR1D1	2.100092	1.650094	12.05763	1.22E-06	0.000407
STOM	2.787742	8.491456	11.9685	1.29E-06	0.000419
SLC37A2	2.000089	1.879379	11.91194	1.34E-06	0.00043
CORO2B	1.800284	1.877505	11.82079	1.43E-06	0.00045
CLIC6	2.017877	7.534631	11.8087	1.44E-06	0.00045
NEBL	1.67142	4.915738	11.74272	1.51E-06	0.000465
AK3L2	3.2742	4.68958	11.5105	1.77E-06	0.00052
CD9	1.957475	9.017817	11.4009	1.91E-06	0.000555
CD59	2.359137	4.540559	11.38296	1.93E-06	0.000556
ADD3	2.022682	4.342901	11.22375	2.17E-06	0.000608
NELL2	1.785069	2.230771	11.20388	2.20E-06	0.00061
C7orf24	2.83951	7.694354	11.14532	2.29E-06	0.000622

Gene names	logFC	AveExpr	t	P.Value	adj.P.Val
RPS6KA5	2.346569	3.952678	11.12772	2.32E-06	0.000623
K6IRS3	2.722597	3.077112	11.04311	2.47E-06	0.000656
ZBED2	1.730361	2.295179	10.78236	2.98E-06	0.000777
MRFAP1	1.726017	10.706	10.68548	3.21E-06	0.000818
TXNIP	1.580753	7.698997	10.65784	3.27E-06	0.000827
LOC51136	2.368675	4.98381	10.63218	3.34E-06	0.000834
PIM2	1.597915	5.059271	10.57577	3.48E-06	0.000853
VDR	1.519313	3.414228	10.54565	3.56E-06	0.000859
CDKN1B	2.082787	4.791543	10.44634	3.84E-06	0.000906
MPHOSPH10	2.212112	7.104616	10.30947	4.27E-06	0.000996
OCA2	1.867575	1.509298	10.2479	4.47E-06	0.001035
SASH1	2.043579	5.321343	10.20717	4.62E-06	0.001059
C10orf78	1.514958	4.20629	10.15712	4.80E-06	0.001072
LOC402571	1.788162	1.480121	10.15385	4.81E-06	0.001072
FLJ10847	1.706609	4.777209	10.14808	4.83E-06	0.001072
TKTL1	1.746747	1.532555	10.14453	4.85E-06	0.001072
DTX2	1.500563	5.387984	10.10357	5.01E-06	0.001088
C6orf81	3.053381	3.195816	10.00826	5.39E-06	0.001159
ACADM	2.163126	5.088138	10.00085	5.43E-06	0.001159
DPYSL2	2.289778	7.636567	9.981272	5.51E-06	0.001168
SCNN1A	1.796135	7.038514	9.967886	5.57E-06	0.00117
STEAP2	1.64914	4.414088	9.894504	5.90E-06	0.00123
CHPT1	1.50594	8.072664	9.845633	6.14E-06	0.001267
AK3L1	1.663029	4.08908	9.826847	6.23E-06	0.001267
TPARL	1.418246	4.441989	9.826153	6.24E-06	0.001267
PIK3R1	1.77048	4.147646	9.805519	6.34E-06	0.001271
P8	2.073582	10.40554	9.80225	6.36E-06	0.001271
Ells1	1.662464	5.610505	9.760964	6.57E-06	0.001292

Gene names	logFC	AveExpr	t	P.Value	adj.P.Val
LAMC3	1.763048	1.955347	9.760605	6.57E-06	0.001292
TMEM38B	1.724547	6.17377	9.689072	6.97E-06	0.00135
C1QDC1	1.762693	5.34842	9.642282	7.24E-06	0.001392
CA12	1.623897	9.506517	9.544074	7.84E-06	0.001497
TBC1D2	1.381765	4.953632	9.528922	7.94E-06	0.001504
TMEM63C	1.799821	2.307725	9.494385	8.17E-06	0.001515
EPB41L3	1.455999	5.362373	9.492897	8.18E-06	0.001515
ATP6V0E	1.458568	8.09837	9.473763	8.31E-06	0.001528
PRR11	1.927298	4.519479	9.409541	8.77E-06	0.0016
KIAA1904	1.561581	1.847721	9.363722	9.11E-06	0.00165
ZFP36	1.402828	6.923361	9.332927	9.35E-06	0.001669
WWC1	1.607798	8.222298	9.332531	9.35E-06	0.001669
CMTM7	1.576546	7.787263	9.305357	9.56E-06	0.001673
PARN	1.361094	6.434886	9.30471	9.57E-06	0.001673
ZA20D3	1.638582	5.592099	9.296434	9.64E-06	0.001673
LAMA3	1.73888	3.992841	9.278075	9.79E-06	0.001685
MTMR9	2.176248	5.180461	9.271379	9.84E-06	0.001685
MCEE	1.379123	7.610284	9.24759	1.00E-05	0.001708
TMEM87A	1.612813	8.481558	9.129759	1.11E-05	0.001863
GJE1	1.399273	1.773791	9.127373	1.11E-05	0.001863
ENPP1	1.886087	4.396255	9.116556	1.12E-05	0.001863
CACYBP	1.377135	6.968541	9.11083	1.13E-05	0.001863
APM-1	2.089249	2.219014	9.097399	1.14E-05	0.001863
ZIC2	1.758413	4.893445	9.0929	1.15E-05	0.001863
VIPR1	1.556527	5.770895	9.071359	1.17E-05	0.001881
LOC284998	1.361399	1.889829	9.063934	1.17E-05	0.001881
PTPNS1	1.301554	4.891673	9.027566	1.21E-05	0.001917
TACC2	1.460942	5.521005	8.998921	1.24E-05	0.001952
PEA15	1.476634	8.332356	8.981227	1.26E-05	0.00197

Gene names	logFC	AveExpr	t	P.Value	adj.P.Val
FXYD3	1.565722	4.190296	8.941277	1.31E-05	0.002027
DEGS1	1.380817	7.171196	8.914403	1.34E-05	0.002049
DBI	1.348876	11.76728	8.865392	1.39E-05	0.002106
TMEM56	1.403975	3.749748	8.863684	1.40E-05	0.002106
LOC653994	3.125666	6.214391	8.862263	1.40E-05	0.002106
CHMP4B	1.354345	6.989356	8.854508	1.41E-05	0.002108
HSD17B2	1.674438	2.347563	8.809591	1.46E-05	0.002155
BCL7C	1.555803	6.820719	8.808992	1.47E-05	0.002155
CENPA	1.364502	6.46569	8.7966	1.48E-05	0.002161
C17orf79	1.483221	8.94163	8.703938	1.61E-05	0.00231
LOC652838	1.562385	3.889038	8.662041	1.67E-05	0.002375
LOC112714	2.200288	2.750652	8.659927	1.67E-05	0.002375
PIK3R2	1.378641	7.995042	8.639506	1.70E-05	0.002405
ATAD2	2.365783	7.600269	8.618545	1.74E-05	0.002437
LRRC50	1.998467	3.591523	8.561789	1.83E-05	0.00255
CCNDBP1	1.269185	5.382571	8.539124	1.86E-05	0.002587
CCDC34	1.816732	6.603143	8.533492	1.87E-05	0.002587
LSM12	1.65717	4.468929	8.46899	1.99E-05	0.002727
GPR124	1.811137	2.686037	8.437418	2.04E-05	0.00279
LCMT1	1.707087	9.076933	8.42194	2.07E-05	0.0028
AFMID	1.819567	4.873346	8.406914	2.10E-05	0.002821
LYPLA1	2.164587	6.119327	8.402324	2.11E-05	0.002821
LOC653573	2.420749	4.532301	8.380924	2.15E-05	0.002861
TAF5L	1.278887	4.87725	8.371951	2.17E-05	0.002869
PLEKHA6	1.438874	5.244566	8.354362	2.20E-05	0.002886
AHNAK	2.537399	10.62489	8.345012	2.22E-05	0.002886
ZNF541	1.773922	3.028752	8.330581	2.25E-05	0.002889
PGAM1	1.372906	7.443974	8.324946	2.27E-05	0.002889

Gene names	logFC	AveExpr	t	P.Value	adj.P.Val
AZGP1	2.305327	5.628465	8.275193	2.37E-05	0.002991
NEK8	1.316735	4.284639	8.271032	2.38E-05	0.002991
ARL4	1.165802	3.962396	8.26388	2.40E-05	0.002996
BCL6	1.779363	6.399555	8.249116	2.43E-05	0.003002
GOT1	1.335848	9.481627	8.245607	2.44E-05	0.003002
PPP1R13B	1.245346	6.538063	8.228146	2.48E-05	0.003013
LOC440093	1.179093	8.210278	8.217893	2.50E-05	0.003013
ATP1A4	1.170734	1.665272	8.216388	2.50E-05	0.003013
Pfs2	1.133933	10.28445	8.212073	2.51E-05	0.003013
DJ122O8.2	1.615597	5.791361	8.210305	2.52E-05	0.003013
PTCHD2	1.524715	2.320475	8.165312	2.63E-05	0.003127
BIRC3	1.458939	2.688356	8.158271	2.64E-05	0.003133
ST3GAL4	1.479947	6.755267	8.143084	2.68E-05	0.003163
PGRMC2	1.479867	8.898475	8.100146	2.79E-05	0.003277
HOMER2	1.534795	5.969928	8.092732	2.81E-05	0.003278
F3	2.106674	2.958911	8.089917	2.82E-05	0.003278
ERRFI1	1.888886	6.645406	8.07143	2.87E-05	0.00332
MFSD7	1.195167	4.224474	8.041786	2.95E-05	0.003392
FADS1	1.164741	6.258118	8.039058	2.96E-05	0.003392
HMGB3	1.998339	5.753253	8.025418	3.00E-05	0.003415
SLD5	1.726517	4.969047	8.022336	3.01E-05	0.003415
NCKIPSD	1.280551	4.728795	7.99525	3.08E-05	0.003488
LOC653542	1.477976	3.723678	7.988471	3.10E-05	0.003495
AP3S1	2.033579	7.076574	7.977208	3.14E-05	0.003517
FAH	1.426141	6.969077	7.939838	3.25E-05	0.003611
ATBF1	1.937874	4.7041	7.93931	3.25E-05	0.003611
ABCC8	2.74081	3.008485	7.870218	3.48E-05	0.003828
C9orf52	1.354974	4.684817	7.862567	3.50E-05	0.003839
TSPYL2	1.19644	2.650572	7.826771	3.62E-05	0.003945

Gene names	logFC	AveExpr	t	P.Value	adj.P.Val
IQCB1	1.521284	4.38299	7.804821	3.70E-05	0.004007
AP1B1	1.152021	6.499343	7.790212	3.76E-05	0.004029
C22orf19	1.501704	5.540714	7.790061	3.76E-05	0.004029
ZDHHC14	1.437858	4.928627	7.764935	3.85E-05	0.004111
SCUBE2	1.763455	6.002359	7.757778	3.88E-05	0.004122
CREB3L2	1.576354	6.051142	7.744306	3.93E-05	0.004159
OACT2	1.575872	4.029872	7.734949	3.96E-05	0.00418
LOC648081	3.062069	4.331083	7.717772	4.03E-05	0.004229
TRIM22	1.975032	1.837148	7.710171	4.06E-05	0.004229
SOD2	1.53913	5.268183	7.663836	4.25E-05	0.004389
HSPA5	1.300971	6.340183	7.656811	4.28E-05	0.004401
GNE	2.119577	5.252873	7.617019	4.45E-05	0.004558
LOC653764	1.159927	3.233037	7.554682	4.73E-05	0.004725
GM2A	1.608629	7.562598	7.54449	4.78E-05	0.004725
TDP1	1.054533	4.714235	7.537856	4.81E-05	0.004725
ZMAT2	1.243784	7.891867	7.534989	4.83E-05	0.004725
SCNN1G	1.656161	2.519555	7.52853	4.86E-05	0.004725
PRIM2A	1.205765	3.281389	7.493563	5.03E-05	0.004826
ZNF652	1.234224	3.709116	7.491143	5.04E-05	0.004826
CEP70	1.581045	5.295254	7.48842	5.06E-05	0.004826
LOC650122	1.275248	1.995702	7.483643	5.08E-05	0.004826
NOTCH2	1.529359	5.082578	7.482695	5.08E-05	0.004826
C16orf61	1.262664	9.572917	7.475518	5.12E-05	0.004826
ELF5	1.769694	6.904522	7.4735	5.13E-05	0.004826
LOC652294	1.894334	1.228968	7.46951	5.15E-05	0.004826
PRKAB2	1.241706	4.628705	7.469134	5.15E-05	0.004826
C14orf24	1.985624	4.502168	7.451021	5.25E-05	0.004878
ORMDL3	1.051999	5.521266	7.443156	5.29E-05	0.004896

Gene names	logFC	AveExpr	t	P.Value	adj.P.Val
PPL	1.665219	5.368409	7.438066	5.32E-05	0.004896
CALD1	1.703979	4.359535	7.436282	5.33E-05	0.004896
RRM2	2.18774	4.957304	7.430683	5.36E-05	0.004906
MAN1A1	1.633249	3.688569	7.412461	5.46E-05	0.004978
FAM46C	1.077647	3.692173	7.403368	5.51E-05	0.004984
LOC650230	1.105999	5.491164	7.399392	5.53E-05	0.004984
MT1X	1.867179	5.576518	7.398868	5.53E-05	0.004984
TCEB1	1.24084	8.077593	7.394846	5.55E-05	0.004984
LIN7B	1.374432	4.895974	7.393313	5.56E-05	0.004984
SLC41A2	1.049417	4.373705	7.38051	5.64E-05	0.005028
ORM2	1.291463	2.17714	7.377659	5.65E-05	0.005028
SLC31A2	1.17148	6.319397	7.32538	5.96E-05	0.005218
SCP2	1.344404	3.04687	7.325179	5.96E-05	0.005218
BTC	1.118225	2.054918	7.318826	6.00E-05	0.005218
CDC2L1	1.351755	4.267504	7.317142	6.01E-05	0.005218
LPIN1	1.604086	5.053505	7.309351	6.06E-05	0.005218
AXUD1	1.158832	5.815695	7.308692	6.06E-05	0.005218
KCNB1	1.713411	4.252847	7.307179	6.07E-05	0.005218
KCNJ11	1.105714	3.293388	7.29957	6.12E-05	0.005218
ARMET	1.414313	9.494867	7.299418	6.12E-05	0.005218
BCMP11	1.142404	8.545368	7.293246	6.16E-05	0.005218
ASB9	1.283368	3.18197	7.250937	6.43E-05	0.005394
MAP3K6	1.234613	5.335312	7.244949	6.47E-05	0.005409
CPT2	1.081307	6.606611	7.24121	6.50E-05	0.005412
LRRC31	1.076795	3.660995	7.235736	6.53E-05	0.005424
IRX3	1.174384	10.03845	7.227379	6.59E-05	0.005453
LOC134147	1.033864	7.109519	7.222813	6.62E-05	0.005455
CDKN2D	1.550455	3.879818	7.218737	6.65E-05	0.005455
FLJ37587	1.19276	2.092817	7.215934	6.67E-05	0.005455

Gene names	logFC	AveExpr	t	P.Value	adj.P.Val
LOC644162	1.631162	4.388375	7.204917	6.74E-05	0.005455
HDAC11	1.336569	5.109544	7.204165	6.75E-05	0.005455
C9orf152	1.401767	8.829361	7.201624	6.77E-05	0.005455
CCDC21	1.088895	4.466941	7.174105	6.96E-05	0.005541
SRD5A1	1.203786	3.854597	7.167219	7.01E-05	0.005563
GNPTAB	1.261656	6.803291	7.156128	7.09E-05	0.005609
MGC16597	1.148421	3.893152	7.142418	7.19E-05	0.005657
RBBP7	1.393933	8.850306	7.141822	7.20E-05	0.005657
MARVELD3	1.327579	5.49713	7.138457	7.22E-05	0.005659
CTSB	1.130508	4.758672	7.129402	7.29E-05	0.005691
FMO5	2.216232	3.855978	7.127045	7.31E-05	0.005691
LOC644940	1.21857	2.158262	7.11924	7.37E-05	0.005719
CNTNAP2	1.349169	5.947829	7.113462	7.41E-05	0.005727
FAM107B	1.444614	6.848921	7.111188	7.43E-05	0.005727
RFWD3	1.140238	6.47019	7.10665	7.46E-05	0.005727
LPIN3	1.11979	3.886226	7.106019	7.47E-05	0.005727
EDG7	1.081767	1.430067	7.10307	7.49E-05	0.005727
LRP5L	1.296084	4.06011	7.097259	7.54E-05	0.005728
MLPH	1.194527	9.002201	7.071885	7.74E-05	0.005863
TSPAN33	1.307762	5.695715	7.065088	7.79E-05	0.005869
LOC643790	1.294368	4.985531	7.04265	7.98E-05	0.005972
STK39	1.598665	4.472688	6.986924	8.46E-05	0.006285
GJB2	1.28007	3.766275	6.985569	8.47E-05	0.006285
CFLAR	1.547916	4.528957	6.966245	8.65E-05	0.006377
KRT5	1.112664	2.497691	6.9606	8.70E-05	0.006396
NAGK	1.301439	8.173884	6.953678	8.76E-05	0.006424
EHF	1.87392	4.487412	6.94723	8.82E-05	0.006424
CLDN15	1.263699	3.834684	6.940359	8.89E-05	0.006424

Gene names	logFC	AveExpr	t	P.Value	adj.P.Val
THOC4	1.478509	7.664515	6.939954	8.89E-05	0.006424
SAT	1.064981	8.491101	6.931995	8.97E-05	0.006446
CDC42EP4	1.555788	8.813936	6.92805	9.00E-05	0.00645
CDKN1A	1.251825	7.462719	6.920199	9.08E-05	0.00648
NFYB	1.214783	4.061118	6.918296	9.10E-05	0.00648
GLUL	1.19407	2.68405	6.890709	9.37E-05	0.006579
PNLIP	1.177746	2.076407	6.871868	9.56E-05	0.006675
C6orf72	1.187723	7.148932	6.868248	9.60E-05	0.006682
KRT4	1.037154	2.851756	6.859077	9.69E-05	0.006729
NET1	1.970329	6.4492	6.84634	9.82E-05	0.006802
ANKRD35	1.064073	2.631391	6.822457	0.000101	0.006929
C15orf38	1.014797	4.114602	6.821327	0.000101	0.006929
PDHA1	1.032999	9.328679	6.800543	0.000103	0.007066
RHOU	1.53659	2.859254	6.785547	0.000105	0.007161
LGALS3	1.081104	7.72406	6.775293	0.000106	0.007218
ZWINT	1.045322	5.767558	6.766292	0.000107	0.007251
G6PC	1.361825	2.086313	6.762761	0.000107	0.007251
GPR137B	1.342056	7.074301	6.758991	0.000108	0.007251
GAJ	1.151557	4.15894	6.751127	0.000109	0.007273
POLE2	1.000437	6.311002	6.744031	0.00011	0.007292
C9orf45	1.558105	4.518117	6.743852	0.00011	0.007292
ANXA2	1.281556	7.531839	6.736747	0.000111	0.0073
TNPO2	1.362071	7.100113	6.729499	0.000111	0.007309
PLA2G10	1.249495	3.709156	6.713024	0.000113	0.007379
EFHD1	1.393412	11.12177	6.71106	0.000114	0.007379
NFIC	1.081552	3.80997	6.706062	0.000114	0.0074
LOC652924	2.128283	4.938796	6.691392	0.000116	0.007461
SLC36A2	1.01214	1.583792	6.675889	0.000118	0.007499
CDC2L6	1.356135	5.046475	6.674511	0.000118	0.007499

Gene names	logFC	AveExpr	t	P.Value	adj.P.Val
GPD1L	1.421982	8.681407	6.667405	0.000119	0.007514
TESC	1.648023	3.025269	6.66617	0.000119	0.007514
SNAP23	1.211209	4.26673	6.660033	0.00012	0.007527
DPYSL3	1.181801	3.647664	6.642354	0.000122	0.007608
BNIP3L	1.568805	6.228162	6.631178	0.000124	0.007636
RBPMS2	1.000525	6.410819	6.625766	0.000125	0.007636
GREB1	1.746988	5.020427	6.6232	0.000125	0.007636
TAGLN	1.262109	3.744325	6.622634	0.000125	0.007636
LY6G5B	1.504502	1.622023	6.61976	0.000126	0.007636
DCLRE1C	1.119659	4.708985	6.612748	0.000127	0.007676
ACBD7	1.009012	3.608625	6.58	0.000131	0.007899
LOC653105	1.090195	1.37423	6.57639	0.000132	0.007912
CDKN1C	1.341066	2.420911	6.573798	0.000132	0.007915
LOC649095	1.592515	2.229586	6.563275	0.000134	0.007989
SLC29A2	1.165311	8.21697	6.545225	0.000136	0.008072
BRCA1	1.124782	3.498472	6.540474	0.000137	0.008095
LOC649362	1.131172	1.312481	6.537889	0.000137	0.008099
FLJ35934	1.033481	3.652923	6.517756	0.00014	0.008224
IFRD1	1.011146	3.356236	6.501833	0.000143	0.008334
RANBP1	1.49019	7.271241	6.489302	0.000145	0.008389
CD44	1.214953	5.249879	6.483694	0.000146	0.008403
NAP1L4	1.43605	8.776056	6.462547	0.000149	0.008544
LFNG	1.537073	4.897026	6.459355	0.00015	0.008555
TGOLN2	1.00132	6.233254	6.45346	0.000151	0.008572
GABARAPL1	1.139003	5.349587	6.447232	0.000152	0.008612
PDDC1	1.011916	5.046285	6.435047	0.000154	0.00871
GJA1	1.339031	5.121771	6.423133	0.000156	0.008805
PRRG4	1.280934	3.854865	6.413016	0.000158	0.008868

Gene names	logFC	AveExpr	t	P.Value	adj.P.Val
CDC2	1.250543	7.438262	6.399983	0.00016	0.008946
LOC650737	1.113818	4.231721	6.399148	0.00016	0.008946
MRAS	1.215012	3.355937	6.391738	0.000162	0.008969
SMA4	1.29784	6.04368	6.390995	0.000162	0.008969
CCDC28A	1.0445	4.71087	6.384735	0.000163	0.00901
ARID1A	1.979666	4.354564	6.379879	0.000164	0.00901
RRAGC	1.118859	5.043378	6.376693	0.000164	0.009014
LCE2A	1.372951	1.941304	6.374176	0.000165	0.00902
IQGAP3	1.276158	7.72233	6.364404	0.000167	0.009093
LOC646299	1.297223	1.374864	6.362951	0.000167	0.009093
RAB3B	1.132111	2.570668	6.359326	0.000168	0.009093
RAD51AP1	1.156536	4.80156	6.345448	0.00017	0.009161
CCAR1	1.095944	6.533388	6.345053	0.00017	0.009161
PPP2CB	1.13911	5.631941	6.339178	0.000172	0.009182
SLN	1.130451	2.063981	6.330996	0.000173	0.009208
TA-PP2C	1.502436	4.015086	6.320981	0.000175	0.009265
CEP152	1.379021	4.245977	6.320615	0.000175	0.009265
AKR1C3	1.564169	3.394563	6.317537	0.000176	0.009265
ACTN1	1.36731	7.71704	6.302116	0.000179	0.009374
SAP30	1.583807	4.954299	6.297937	0.00018	0.009399
ACYP1	1.088833	5.955545	6.291379	0.000181	0.009415
ALDH3A2	1.019721	6.443318	6.290434	0.000181	0.009415
TUBA2	1.700702	1.76356	6.273609	0.000185	0.009523
CAMK2N1	1.321155	6.653525	6.255465	0.000189	0.009662
STK3	1.001077	5.986435	6.248794	0.00019	0.009686
RUTBC1	1.245908	8.94615	6.247937	0.00019	0.009686
TMEM16A	1.030008	4.036291	6.243514	0.000191	0.009715
EAF2	1.556155	3.036968	6.232752	0.000194	0.009815
HIPK2	1.158431	5.283447	6.219774	0.000196	0.009912

Gene names	logFC	AveExpr	t	P.Value	adj.P.Val
ARL6IP6	1.870973	5.277312	6.219107	0.000197	0.009912
MGC72104	1.292607	5.359138	6.218887	0.000197	0.009912
LOC651348	1.605155	4.934609	6.197982	0.000201	0.010132
FLJ25422	1.421256	3.170635	6.195831	0.000202	0.010136
ST3GAL1	1.075148	3.468536	6.186506	0.000204	0.010184
LOC255783	1.303989	7.13662	6.180422	0.000206	0.010215
LOC641997	1.1019	1.18635	6.170161	0.000208	0.010315
LOC654037	1.189011	3.50697	6.146449	0.000214	0.010543
ZNF503	1.188443	2.898289	6.146281	0.000214	0.010543
C5	1.306425	5.008272	6.14601	0.000214	0.010543
DUSP1	1.086255	6.059355	6.124981	0.000219	0.010718
C1orf168	1.114186	3.361023	6.120371	0.00022	0.010751
NETO2	1.239136	3.240263	6.118957	0.000221	0.010751
SLC6A6	1.270082	2.939723	6.111776	0.000222	0.01082
CPEB4	2.631552	4.090483	6.089502	0.000228	0.011039
HIST1H2BF	1.221433	2.348894	6.088857	0.000228	0.011039
LOC196752	1.124493	4.843489	6.087785	0.000229	0.011039
DCUN1D3	1.018998	4.732256	6.073079	0.000233	0.011164
TSNAX	2.002513	5.471275	6.065936	0.000235	0.011221
STAT3	1.428993	5.449657	6.058533	0.000237	0.011279
SLC2A3	1.395889	1.987991	6.049499	0.000239	0.011279
GRHL2	2.236432	5.063996	6.049223	0.000239	0.011279
SLC15A2	1.038349	2.398979	6.048179	0.00024	0.011279
ADHFE1	1.059755	5.028487	6.043792	0.000241	0.011292
ARHGEF4	1.599949	1.850253	6.040521	0.000242	0.011304
PTPLAD1	1.772144	6.994643	5.990867	0.000256	0.011824
MT1F	1.222485	4.371596	5.990114	0.000256	0.011824
JARID2	1.342525	4.831328	5.988109	0.000257	0.01183

Gene names	logFC	AveExpr	t	P.Value	adj.P.Val
AQP3	1.500651	2.543284	5.969045	0.000263	0.012053
DKFZp686L1814	1.387573	3.977631	5.948936	0.000269	0.012297
TNRC6B	1.045909	3.679564	5.945326	0.00027	0.012304
AUTS2	1.278608	6.938909	5.94078	0.000272	0.012348
PPP1R14C	1.54866	1.858488	5.907431	0.000283	0.01266
GBA	1.008618	7.447022	5.902594	0.000284	0.012688
SRP9	1.375145	4.718161	5.895935	0.000286	0.012734
KIAA0101	1.051884	8.689492	5.859341	0.000299	0.013031
C10orf9	1.400814	5.775145	5.839947	0.000306	0.013142
LOC652506	1.077822	5.192471	5.831443	0.000309	0.01321
MANBA	1.08222	6.3032	5.830509	0.00031	0.01321
LOC389641	1.291669	4.583636	5.827935	0.000311	0.013217
C10orf113	1.197774	2.31158	5.826582	0.000311	0.013217
PSPH	1.211999	5.503455	5.814034	0.000316	0.013354
CLDN12	1.096041	5.517323	5.801125	0.000321	0.013394
LRRFIP2	1.172717	5.348608	5.799951	0.000321	0.013394
UGT2B28	1.215864	1.964115	5.799487	0.000321	0.013394
DDX46	1.457497	5.770321	5.79783	0.000322	0.013394
ARHGAP26	1.54188	1.917911	5.788033	0.000326	0.013507
APITD1	1.001209	5.812258	5.775021	0.000331	0.013652
SCGB1D2	1.206991	2.515072	5.770326	0.000333	0.013662
CALR	1.071376	7.620536	5.769275	0.000333	0.013662
SH3BP2	1.101004	2.011511	5.766842	0.000334	0.013662
SHMT1	1.222523	7.06266	5.765601	0.000335	0.013662
LOC652726	1.128368	3.432896	5.741857	0.000344	0.014
CMIP	1.353776	3.716871	5.736603	0.000347	0.014066
DENND2C	1.237972	3.389107	5.732912	0.000348	0.014083
PNPO	1.128304	8.761605	5.727474	0.00035	0.014153
SEPP1	1.624269	6.761981	5.725502	0.000351	0.014164

Gene names	logFC	AveExpr	t	P.Value	adj.P.Val
LOC643523	1.044037	1.257456	5.718365	0.000354	0.014264
ABHD3	1.357485	3.77016	5.700594	0.000362	0.01448
ELOVL5	1.518135	7.272912	5.697861	0.000363	0.014505
CSNK1A1	1.126117	5.045787	5.68805	0.000368	0.014562
AKAP10	1.006882	4.121598	5.684529	0.000369	0.014579
NICN1	1.02501	5.995554	5.681645	0.00037	0.014607
TM2D1	1.31326	5.218781	5.661775	0.00038	0.014893
C10orf41	1.070357	2.027106	5.651275	0.000384	0.014982
SNRPD3	1.265217	4.691904	5.646669	0.000387	0.014982
RBBP5	1.591952	5.060687	5.644408	0.000388	0.014982
TRUB1	1.399919	2.389081	5.639351	0.00039	0.015046
MTCH2	1.065575	4.681048	5.631718	0.000394	0.015135
STXBP3	1.4919	4.704132	5.612516	0.000403	0.015202
PPP1R3C	2.034687	3.226958	5.61231	0.000403	0.015202
EDN2	1.066486	2.561956	5.585159	0.000417	0.015619
FANCD2	1.055382	3.968479	5.583916	0.000417	0.015619
F2R	1.402016	1.822994	5.582557	0.000418	0.015622
CRIPT	1.006501	5.661942	5.580882	0.000419	0.015631
IFRG15	1.21525	2.418644	5.579391	0.00042	0.015636
TRA2A	1.594391	5.437435	5.573865	0.000423	0.015696
LOC643287	1.294969	4.908851	5.572349	0.000423	0.015702
DCK	1.218176	4.484924	5.561119	0.000429	0.015856
FKSG44	1.12106	7.342415	5.5608	0.000429	0.015856
ZFYVE21	1.010252	8.56769	5.553894	0.000433	0.015944
GPR160	1.211359	5.082239	5.545477	0.000438	0.016039
FBXO5	1.526791	6.06669	5.543414	0.000439	0.016039
HMGB2	1.292104	6.66221	5.543109	0.000439	0.016039
PBEF1	1.065643	3.611958	5.538833	0.000441	0.016078

Gene names	logFC	AveExpr	t	P.Value	adj.P.Val
FRK	1.151874	1.432635	5.537067	0.000442	0.016078
CWF19L1	1.176516	4.449359	5.525747	0.000448	0.016185
LOC648366	2.565304	2.879348	5.516449	0.000454	0.016215
CKAP4	1.170673	7.41545	5.5144	0.000455	0.016215
LOC651316	1.442943	2.284341	5.506224	0.000459	0.016282
LOC644844	1.295419	1.237555	5.501858	0.000462	0.016329
PCAF	1.011864	4.96461	5.499302	0.000463	0.016334
ZNF689	1.590213	5.373887	5.488159	0.00047	0.016538
LOC284023	1.181875	4.245327	5.484564	0.000472	0.016541
FLJ40629	1.365966	5.827677	5.482837	0.000473	0.016541
ADFP	1.021724	6.071381	5.482397	0.000473	0.016541
ANXA11	1.096306	3.817149	5.469922	0.00048	0.016728
THRAP6	1.171108	5.504793	5.465582	0.000483	0.016795
FAIM	1.182834	3.740761	5.461534	0.000486	0.016833
TDRD10	1.104878	1.639836	5.448556	0.000493	0.017013
RAB1A	1.062584	6.459852	5.442592	0.000497	0.017096
GLRX	1.117376	5.701408	5.431813	0.000504	0.017247
PPARA	1.002139	1.835059	5.409482	0.000518	0.017596
GPR153	1.372468	2.180432	5.400928	0.000524	0.017714
ITGA2B	1.166807	4.571426	5.368908	0.000545	0.018025
SLC16A6	1.126371	3.614425	5.36664	0.000547	0.018045
EMP1	1.276888	2.319504	5.353042	0.000556	0.01822
NPHP3	1.134975	3.956512	5.344443	0.000562	0.018394
PFN2	1.321555	6.48185	5.310034	0.000587	0.018996
CTNNB1	1.351884	3.010847	5.309637	0.000587	0.018996
GPC4	1.366681	3.261827	5.297422	0.000597	0.019186
TMEM41B	1.019192	6.326948	5.291457	0.000601	0.019215
DSCR1	1.285104	8.764807	5.285755	0.000605	0.019269
FASN	1.12513	11.3947	5.284729	0.000606	0.019269

Gene names	logFC	AveExpr	t	P.Value	adj.P.Val
PNMA2	1.079674	1.470939	5.284683	0.000606	0.019269
LOC440934	1.066425	1.886901	5.282264	0.000608	0.019269
ALS2CR15	1.177761	2.527478	5.276631	0.000613	0.019333
C17orf55	1.065278	3.146334	5.267275	0.00062	0.019395
GNB1	1.111501	9.783337	5.261868	0.000624	0.019407
RDH10	1.080711	5.100614	5.261796	0.000624	0.019407
MUC1	1.100509	10.96584	5.23812	0.000643	0.019852
KLF15	1.095924	2.691456	5.237458	0.000644	0.019852
GSTM3	1.1433	5.778263	5.237163	0.000644	0.019852
MMP13	1.052211	1.334077	5.232462	0.000648	0.019947
CDC2L2	1.199688	4.045803	5.210481	0.000666	0.02029
LOC649536	1.229405	2.110855	5.196042	0.000679	0.020593
SLC7A8	1.013654	3.066135	5.19125	0.000683	0.02067
MMACHC	1.393989	4.741303	5.189125	0.000685	0.020676
LOC648695	1.798074	4.570938	5.180968	0.000692	0.020868
LOC153561	1.420763	5.48411	5.179284	0.000694	0.020888
STK24	1.189985	7.289359	5.177738	0.000695	0.020905
EFCAB3	1.245204	2.685827	5.165632	0.000706	0.021055
LOC400879	1.208259	4.632947	5.156591	0.000714	0.0212
UBE2G1	1.131912	5.078949	5.135248	0.000734	0.021535
HMG1L1	1.279277	5.090287	5.098906	0.000769	0.022028
MYBPH	1.025229	2.52882	5.078408	0.00079	0.0223
CEACAM3	1.033083	0.661002	5.076338	0.000792	0.0223
CHRNA5	1.022072	6.515578	5.076282	0.000792	0.0223
HFE	1.155503	2.436119	5.061477	0.000808	0.022555
KCNK1	1.006407	3.679909	5.060338	0.000809	0.022563
FNBP1L	1.047122	6.265158	5.054903	0.000815	0.022698
GALNT6	1.101203	4.080341	5.041598	0.000829	0.022891

Gene names	logFC	AveExpr	t	P.Value	adj.P.Val
PACSIN2	1.057006	7.036309	5.034506	0.000836	0.022974
RRP22	1.344726	2.766989	5.033608	0.000837	0.022974
SPFH1	1.02612	4.452627	5.032069	0.000839	0.022974
CCDC58	1.60894	5.071186	5.014289	0.000859	0.023309
FLJ23191	1.023998	2.114362	5.010136	0.000863	0.023386
RAD21	1.119717	4.387136	5.005519	0.000869	0.0234
CHD1L	1.001022	4.767566	4.997611	0.000878	0.023574
AFF4	2.411751	4.886309	4.99237	0.000884	0.023628
HIATL2	1.243518	3.607133	4.990574	0.000886	0.023634
PLEKHB2	1.199126	3.537175	4.981913	0.000896	0.023726
CDC42EP3	1.351173	2.566721	4.980434	0.000898	0.023747
ADAM9	1.06956	3.75716	4.966412	0.000914	0.024015
NGFR	1.091038	1.058436	4.95935	0.000923	0.024158
LEPROT	1.019429	6.768757	4.933266	0.000955	0.024666
ALDH9A1	1.086021	10.20773	4.931576	0.000957	0.024686
LOC643367	1.247153	5.452508	4.923945	0.000967	0.024767
HNRPH1	2.395399	8.44288	4.914548	0.000979	0.024974
LDLRAD3	1.43451	4.726473	4.907187	0.000989	0.025141
ACSL3	1.47188	3.555763	4.904875	0.000992	0.025192
SPTBN1	1.614843	3.296831	4.89082	0.00101	0.025535
MTSS1	1.262536	2.951978	4.889257	0.001012	0.025535
SAFB2	1.069799	5.363386	4.878807	0.001027	0.025737
SV2B	1.381315	1.802539	4.863454	0.001048	0.026162
NEK7	1.133903	2.291317	4.857962	0.001055	0.026254
POLS	1.130355	5.52559	4.857823	0.001056	0.026254
LOC641825	1.15302	1.433588	4.850604	0.001066	0.026402
ALAD	1.028398	4.620601	4.833913	0.00109	0.026652
IMAA	1.110025	2.06921	4.833058	0.001091	0.026656
ANKRD20A2	1.414317	2.240952	4.830005	0.001095	0.026687

Gene names	logFC	AveExpr	t	P.Value	adj.P.Val
TMPO	1.426524	3.453007	4.827083	0.0011	0.026765
MGC14376	1.016616	5.535553	4.804835	0.001133	0.027306
LOC400987	1.655462	2.54339	4.792164	0.001152	0.02756
GPR89A	1.494046	5.03182	4.790746	0.001154	0.02756
M6PR	1.053059	4.663361	4.783852	0.001165	0.02771
MGC40579	1.060253	4.81278	4.768705	0.001189	0.027986
EIF5	1.742494	3.779925	4.766223	0.001193	0.028008
SP6	1.215764	2.676539	4.742286	0.001232	0.028625
GP1BB	1.257219	6.987634	4.725943	0.001259	0.028962
UBE2A	1.058189	1.146252	4.718051	0.001273	0.029157
U2AF2	1.251633	7.03688	4.71757	0.001274	0.029157
LOC389816	1.065366	7.329159	4.716388	0.001276	0.029177
GULP1	1.371519	2.357207	4.700793	0.001303	0.029531
LOC643668	1.341208	9.204629	4.698067	0.001308	0.029531
LOC653650	1.954341	2.894956	4.696324	0.001311	0.029531
RAB2B	1.325893	5.721727	4.689121	0.001324	0.029622
EFEMP1	1.100327	6.390565	4.679664	0.001341	0.029923
H3F3A	1.01554	12.27952	4.675206	0.001349	0.030051
ANGPTL5	1.038011	1.425501	4.663349	0.001371	0.030263
HIST1H3B	1.416297	1.57349	4.656361	0.001384	0.030344
TBL1X	1.108874	5.748596	4.652239	0.001392	0.030446
LASS6	1.304796	7.250284	4.650315	0.001395	0.030487
INCENP	1.417611	2.290186	4.648508	0.001399	0.030513
LOC642477	1.467552	4.172073	4.647058	0.001401	0.030526
C12orf58	1.075918	3.718904	4.643457	0.001408	0.030594
LOC644799	1.427186	6.999693	4.641055	0.001413	0.030659
ZNF217	1.962164	6.151254	4.617619	0.001459	0.031274
PABPN1	1.548733	5.72096	4.600379	0.001494	0.031776

Gene names	logFC	AveExpr	t	P.Value	adj.P.Val
TIRAP	1.011428	3.709429	4.599156	0.001496	0.031789
LOC650766	1.430516	2.074467	4.595164	0.001504	0.031811
KIAA1671	1.121845	5.630271	4.592218	0.00151	0.031855
IMPDH1	1.229554	6.475237	4.589539	0.001516	0.031895
SSBP2	1.051716	7.207593	4.575964	0.001544	0.032223
HMMR	1.098461	7.586414	4.559629	0.001579	0.032719
LOC643196	1.110186	1.506499	4.5514	0.001597	0.03297
LOC653357	1.244024	2.050949	4.543261	0.001615	0.033202
EGF	1.253637	2.930026	4.540237	0.001622	0.033233
LOC644422	2.569409	5.812507	4.532716	0.001639	0.033414
IGF2R	1.060145	7.644343	4.523839	0.001659	0.033657
SLC35A3	1.060958	6.030968	4.506637	0.001699	0.034191
RCP9	1.089505	3.070858	4.503952	0.001705	0.034285
LOC440422	1.013636	2.288055	4.50146	0.001711	0.034354
CAMKK1	1.105469	2.034986	4.497584	0.00172	0.034479
ZNF69	1.242456	4.893344	4.494024	0.001729	0.034554
NEDD4L	1.081226	4.171195	4.485705	0.001749	0.034775
MT1A	1.482185	7.589415	4.480006	0.001763	0.034904
PER2	1.024568	5.137651	4.473583	0.001778	0.035085
GUSBL1	1.225165	5.614555	4.469179	0.001789	0.035161
LOC653712	1.171608	2.135854	4.467031	0.001795	0.035223
PROK2	1.505252	2.135597	4.464898	0.0018	0.035232
FLJ22313	1.089778	3.54267	4.450519	0.001836	0.035723
TNFAIP8L3	1.038063	2.957033	4.449293	0.001839	0.035723
UPF2	1.18628	5.399766	4.447855	0.001843	0.035728
LOC115648	1.213936	2.331887	4.446519	0.001846	0.03575
ERO1LB	1.374883	2.523329	4.435371	0.001875	0.036141
IMPAD1	1.123998	5.004207	4.434751	0.001877	0.036144
PRICKLE1	1.45908	2.342741	4.430563	0.001888	0.036271

Gene names	logFC	AveExpr	t	P.Value	adj.P.Val
KIAA0889	1.009255	3.979219	4.427032	0.001897	0.036338
LOC644063	1.929685	6.474147	4.421223	0.001913	0.036391
FXR1	1.496212	6.6163	4.420062	0.001916	0.036391
LOC654174	1.958202	2.907551	4.417813	0.001922	0.036445
PDPK1	1.554486	3.647063	4.409076	0.001945	0.036727
LOC641807	1.337592	2.340217	4.404195	0.001958	0.036808
C18orf45	1.078938	4.541868	4.383356	0.002016	0.037301
PHACTR4	1.266769	6.920552	4.379607	0.002027	0.037365
TIA1	1.062262	4.266192	4.359189	0.002086	0.038089
LOC648434	1.064659	2.659317	4.350043	0.002113	0.038324
RING1	1.197846	8.571891	4.349476	0.002114	0.038327
CHIC1	1.257316	1.341693	4.348774	0.002116	0.038334
IFNGR1	1.150259	4.940477	4.314061	0.002222	0.039767
E2F7	1.106366	4.694626	4.30394	0.002254	0.040155
NBPF14	1.128517	3.400742	4.298173	0.002273	0.040263
WHDC1	1.172252	2.584984	4.297627	0.002274	0.040266
SRRM2	1.162289	8.185661	4.290852	0.002296	0.040435
FJX1	1.138362	8.651495	4.290654	0.002297	0.040435
RARG	1.040542	2.262703	4.282242	0.002324	0.040832
ACOX1	1.199496	4.788697	4.278393	0.002337	0.041026
MT2A	1.635793	8.455792	4.273606	0.002353	0.041218
LOC644614	1.491095	2.446486	4.271832	0.002359	0.041264
SHRM	1.068841	4.272162	4.26789	0.002372	0.041466
HSPA2	1.252682	3.06914	4.266872	0.002376	0.041496
C9orf88	1.18073	8.992898	4.247863	0.00244	0.041935
LOC653942	1.062082	8.471147	4.247833	0.002441	0.041935
KIR2DL3	1.169352	1.957272	4.23642	0.00248	0.042322
RIOK3	1.147897	4.690702	4.232908	0.002493	0.042389

Gene names	logFC	AveExpr	t	P.Value	adj.P.Val
TERF1	1.222991	4.489033	4.222783	0.002529	0.042819
DKFZP434A0131	1.857856	6.005999	4.221523	0.002533	0.042819
LOC642576	1.049815	2.168026	4.215656	0.002555	0.043003
LMO7	1.118656	2.170631	4.207921	0.002583	0.043304
LOC645719	1.262558	4.886405	4.205613	0.002591	0.043331
LOC652612	1.354337	1.987093	4.202446	0.002603	0.04344
IL6ST	1.398837	2.425579	4.195779	0.002628	0.04367
LOC652773	1.232228	4.554878	4.190559	0.002648	0.04386
MDM4	1.533895	2.71843	4.182871	0.002677	0.04411
LOC619190	1.601322	2.014285	4.169494	0.002729	0.044489
LOC441425	1.235129	2.231751	4.169094	0.00273	0.044489
FOXO1A	1.273059	3.069543	4.163148	0.002753	0.044694
LOC650141	1.459979	2.476061	4.149034	0.00281	0.045196
RCC2	1.027471	8.452832	4.13405	0.002871	0.045794
DHX33	1.350903	4.666472	4.109732	0.002973	0.046937
LOC643909	1.044035	2.304984	4.098112	0.003023	0.047404
LOC645580	1.050954	2.114837	4.098042	0.003023	0.047404
LOC51315	1.196558	4.089844	4.091071	0.003054	0.047655
LOC652640	1.218799	1.740101	4.086018	0.003076	0.047849
THRAP3	1.157455	2.836917	4.084056	0.003085	0.047955
LOC649456	1.510414	2.24974	4.07422	0.003129	0.04828
PLEKHF1	1.385214	5.735483	4.067196	0.003161	0.048414
PIGA	1.002826	4.688942	4.06166	0.003186	0.048594
LOC647743	1.175119	5.168937	4.05523	0.003216	0.04893
TMEM89	1.003687	1.978535	4.044906	0.003264	0.049335
LYSMD1	1.462014	3.173432	4.038454	0.003295	0.049569

Table S5. The 2937 DEGs in the BRCA group.

Gene names	logFC	log2CPM	P.Value	adj.P.Val
CGA	8.7456539	4.683034	6.22E-33	3.54E-32
CST4	8.3204922	2.415999	3.19E-51	3.20E-50
CSAG1	8.2959413	0.470341	1.58E-33	9.22E-33
IBSP	8.0634148	2.713122	2.53E-80	4.82E-79
MAGEA12	8.0527783	0.91114	1.95E-21	6.85E-21
CST5	7.9451542	3.62691	2.41E-27	1.09E-26
ACTL8	7.8871937	2.430987	1.08E-40	8.03E-40
MAGEA1	7.766988	1.165443	9.49E-25	3.86E-24
KLHL1	7.6524218	1.427917	1.34E-15	3.62E-15
MAGEA3	7.6345171	2.091348	2.71E-16	7.57E-16
SPDYC	7.3682361	2.699592	1.53E-32	8.60E-32
COL10A1	7.1522343	7.232031	4.57E-168	3.04E-166
MAGEA6	7.0033039	1.916001	2.28E-14	5.81E-14
SPANXB2	6.9600963	-0.06347	9.19E-22	3.27E-21
CST1	6.9539976	3.664878	7.73E-69	1.20E-67
CARTPT	6.8806654	7.089233	3.35E-12	7.59E-12
CHRNA9	6.769467	2.889416	1.88E-31	1.01E-30
CASP14	6.7527933	5.310898	5.47E-29	2.66E-28
MAGEC2	6.678575	0.286994	3.74E-17	1.09E-16
MMP1	6.5243353	5.087819	9.71E-65	1.37E-63
PCSK1	6.5209111	5.928433	2.08E-33	1.21E-32
CPLX2	6.4572749	3.191768	1.39E-17	4.13E-17
TRPA1	6.3412831	4.003363	3.05E-41	2.32E-40
RTBDN	6.3132483	1.176403	1.64E-43	1.33E-42
MMP13	6.3035133	4.982848	6.41E-81	1.23E-79
MMP11	6.2845791	8.234613	1.02E-167	6.75E-166

Gene names	logFC	log2CPM	P.Value	adj.P.Val
COL11A1	6.2045223	7.717564	8.23E-110	2.53E-108
CBLN2	6.1002305	3.937289	1.65E-34	1.00E-33
OLFM3	6.0926484	0.562558	1.33E-15	3.59E-15
EPYC	6.0380438	2.017668	6.35E-68	9.59E-67
PRAC2	5.9343444	-1.32312	2.95E-29	1.45E-28
DSCR8	5.9230746	-0.64064	1.68E-23	6.43E-23
LHX1	5.9164733	0.094323	1.57E-20	5.28E-20
HMP19	5.9068784	2.473223	1.45E-31	7.85E-31
POTEC	5.8861415	0.1373	1.27E-20	4.28E-20
SLC30A8	5.8795707	5.939246	1.11E-27	5.11E-27
MAGEA4	5.8629849	2.144685	3.36E-12	7.62E-12
TLX1	5.8500453	1.041259	3.79E-32	2.10E-31
BPIFA1	5.8314589	1.691416	5.21E-11	1.11E-10
GABRA5	5.7980205	-0.7383	4.23E-25	1.74E-24
TRH	5.7810688	4.691281	8.72E-26	3.72E-25
GABRQ	5.7678099	2.050727	9.67E-32	5.26E-31
CLEC3A	5.7330998	7.765186	8.61E-19	2.68E-18
HOXB13	5.6882877	2.451852	1.30E-27	5.95E-27
TLX3	5.6009436	-1.90557	1.87E-15	5.02E-15
LRTM2	5.5994625	0.930233	1.26E-38	8.74E-38
NKX2_2	5.5836308	0.086867	2.77E-22	1.01E-21
GNG13	5.5545139	0.149005	7.03E-53	7.33E-52
KRT1	5.5487818	4.264583	2.81E-21	9.81E-21
C20orf85	5.5319116	0.328965	4.09E-17	1.19E-16
HIST1H3B	5.5284432	-0.43293	1.69E-39	1.21E-38
TLX1NB	5.4909756	-2.36434	2.14E-33	1.24E-32
GRM4	5.4126319	3.143164	9.11E-66	1.32E-64
CNGA2	5.385402	-1.319	3.58E-21	1.24E-20
ORM1	5.3850568	2.648021	7.72E-27	3.43E-26

Gene names	logFC	log2CPM	P.Value	adj.P.Val
IL19	5.3500065	0.093435	1.43E-32	8.04E-32
LIN28A	5.3441735	0.58616	1.70E-19	5.46E-19
HAPLN1	5.3338336	2.606735	1.36E-49	1.30E-48
INSM1	5.3152861	2.951967	6.06E-30	3.07E-29
CYP2A7	5.2892258	4.499741	2.18E-18	6.66E-18
CST2	5.2786198	1.915098	8.31E-58	9.85E-57
KCNJ3	5.246022	5.851008	2.88E-28	1.36E-27
ASCL1	5.216226	2.605186	7.11E-20	2.32E-19
CDC20B	5.203937	2.849105	2.87E-25	1.19E-24
DCD	5.1874453	6.387524	1.27E-11	2.80E-11
METTL11B	5.1710402	-2.05017	1.35E-66	1.99E-65
ERVV_2	5.1576959	-1.67422	1.48E-21	5.23E-21
AMER3	5.1513361	-0.67953	6.38E-18	1.92E-17
CHGA	5.148421	6.330839	2.67E-15	7.11E-15
HIST1H2AJ	5.1011721	-2.04376	8.03E-34	4.74E-33
HIST1H1B	5.0886225	-0.00031	2.93E-32	1.63E-31
SYT13	5.0511751	6.083751	6.67E-41	5.00E-40
PPAPDC1A	5.0205303	3.00816	6.62E-118	2.36E-116
PVALB	5.0079019	3.843736	2.64E-28	1.25E-27
GATA4	4.9981427	0.539737	8.08E-21	2.76E-20
ZFP42	4.9780725	0.228544	9.87E-16	2.69E-15
SLC24A2	4.968833	2.516197	4.85E-93	1.12E-91
CD177	4.9614082	3.253062	8.82E-31	4.63E-30
PRAME	4.9597677	4.872051	5.47E-27	2.45E-26
DMP1	4.9262418	-0.97892	5.30E-32	2.91E-31
TCL1B	4.9214819	-2.06654	9.55E-19	2.97E-18
XKR7	4.9084106	0.458752	5.77E-25	2.36E-24
IGFL3	4.9078912	-0.80037	3.40E-41	2.57E-40

Gene names	logFC	log2CPM	P.Value	adj.P.Val
CDK5R2	4.9052735	-0.27128	7.40E-20	2.41E-19
SMR3B	4.901273	5.699474	1.35E-08	2.54E-08
CLDN6	4.8719552	1.301414	7.00E-26	3.00E-25
CHGB	4.8338507	6.803246	1.40E-15	3.77E-15
CPB1	4.8229291	10.71096	2.27E-17	6.68E-17
CA9	4.8200727	2.708511	1.84E-31	9.91E-31
CSMD3	4.8115648	1.299191	1.68E-16	4.73E-16
TUBA3C	4.8112669	-1.58127	4.34E-19	1.37E-18
S100P	4.7982078	5.81394	3.69E-52	3.78E-51
TEX19	4.786474	-1.17207	2.10E-35	1.31E-34
PLAC1	4.7625851	0.580713	1.82E-73	3.07E-72
S100G	4.7615438	1.08101	6.62E-14	1.64E-13
HIST1H2AI	4.7558791	1.05065	2.90E-58	3.47E-57
CRYBA2	4.7557833	-1.49918	3.81E-25	1.57E-24
NCAN	4.7556548	2.641902	1.42E-16	4.02E-16
NDST4	4.7335054	0.497841	2.07E-14	5.28E-14
CT83	4.7332079	0.419592	1.12E-09	2.24E-09
MUC5AC	4.7059603	3.202157	2.91E-15	7.76E-15
ENSG00000260220	4.7028932	-1.19185	2.03E-31	1.09E-30
SBSN	4.6969735	2.653028	2.06E-20	6.91E-20
HIST1H3F	4.6925978	-1.24538	2.25E-29	1.12E-28
HIST1H4B	4.6649935	-1.50207	2.62E-24	1.04E-23
GNGT1	4.6647078	-1.15805	6.38E-18	1.92E-17
GPR26	4.6453674	2.352007	6.16E-21	2.11E-20
PDX1	4.6260783	-2.86062	2.84E-14	7.20E-14
S100A7A	4.616641	3.268017	2.14E-11	4.65E-11
WT1	4.5948037	2.422374	3.58E-36	2.28E-35
HIST1H2BO	4.5816258	-0.5197	5.74E-57	6.66E-56
NELL1	4.5711754	1.499692	6.31E-17	1.81E-16

Gene names	logFC	log2CPM	P.Value	adj.P.Val
CELF3	4.5587112	-0.0606	1.17E-30	6.13E-30
BPIFB6	4.5392946	-2.53055	6.84E-17	1.96E-16
MYT1	4.5341836	2.001842	4.27E-41	3.22E-40
ZDHHC22	4.5308453	-0.46678	1.79E-21	6.29E-21
COMP	4.5252302	7.05571	4.86E-85	9.97E-84
FOXJ1	4.5248186	2.759621	2.96E-36	1.89E-35
HIST1H4C	4.5170306	-0.456	2.30E-29	1.14E-28
GABRA3	4.5132117	0.193632	7.10E-20	2.32E-19
OR2B6	4.5034069	-0.69921	5.97E-67	8.84E-66
HMX2	4.4945473	-1.91043	2.80E-20	9.31E-20
PYDC1	4.4909642	2.656776	7.69E-26	3.29E-25
DPYSL5	4.4629562	1.11995	3.24E-18	9.85E-18
EIF4E1B	4.4383442	-1.27134	1.15E-19	3.72E-19
MAEL	4.4269248	1.094996	6.40E-27	2.85E-26
CEACAM5	4.3967771	5.689748	3.07E-27	1.38E-26
HIST1H3J	4.3765921	-1.80386	4.67E-55	5.16E-54
LHX2	4.3749392	-0.17126	7.60E-47	6.75E-46
CHRNA6	4.3668739	0.260548	2.37E-61	3.08E-60
CAMP	4.3648562	0.838521	9.82E-45	8.25E-44
ACAN	4.3447985	4.076335	1.59E-40	1.18E-39
CEACAM6	4.3375601	7.726444	1.78E-32	9.96E-32
FAM25A	4.3370123	-0.90506	1.05E-17	3.15E-17
ADAMTS20	4.3369837	-1.87222	4.66E-28	2.18E-27
GBX2	4.3362927	-2.11809	1.01E-47	9.24E-47
HIST1H4D	4.3333075	-0.57269	3.02E-41	2.30E-40
KIF1A	4.3331349	4.137372	4.13E-27	1.85E-26
HIST1H3H	4.3318902	3.514457	2.25E-67	3.36E-66
GJB2	4.315565	5.445637	4.60E-87	9.78E-86

Gene names	logFC	log2CPM	P.Value	adj.P.Val
FABP6	4.3026133	0.048275	6.65E-28	3.10E-27
ORM2	4.3025121	1.866743	2.47E-24	9.82E-24
IFNL2	4.2978829	-3.28818	2.26E-28	1.07E-27
SYT4	4.2938093	1.069602	2.58E-09	5.05E-09
BMPR1B	4.2876589	7.591945	1.16E-33	6.78E-33
SERPINB4	4.284084	0.624578	4.27E-10	8.73E-10
NEK2	4.2831511	4.69843	2.11E-161	1.31E-159
FOXG1	4.2815034	-1.75006	3.82E-17	1.11E-16
WBSCR28	4.2669058	-1.0478	4.74E-62	6.30E-61
LIN28B	4.2620516	-2.17678	1.44E-10	3.00E-10
KRT75	4.2495185	0.933351	3.26E-22	1.18E-21
SYT5	4.242939	0.676589	1.02E-33	6.01E-33
MS4A15	4.2260465	1.97789	4.73E-31	2.51E-30
UBE2C	4.2244114	5.313288	1.58E-114	5.33E-113
KISS1R	4.2213275	0.450405	4.18E-39	2.95E-38
STRA8	4.2067278	-1.35567	3.07E-15	8.15E-15
TBX10	4.1824828	0.357167	4.58E-14	1.15E-13
HIST1H1E	4.1803303	1.353941	9.35E-33	5.29E-32
NPW	4.1769448	0.754121	8.88E-38	5.98E-37
CCL11	4.1751387	0.504565	9.32E-84	1.87E-82
TYRP1	4.1642328	1.181691	1.09E-28	5.24E-28
HORMAD1	4.1620347	1.890209	5.61E-21	1.93E-20
HIST1H2AL	4.157988	-2.1043	1.40E-35	8.79E-35
POU3F2	4.1540026	-0.33487	1.39E-21	4.93E-21
HOXC12	4.1240365	2.535424	9.64E-27	4.27E-26
MSMB	4.1208114	4.471303	1.26E-23	4.87E-23
SLC1A6	4.1141572	0.638225	1.97E-17	5.80E-17
HIST1H4E	4.1082837	0.644568	3.28E-38	2.25E-37
HIST1H2AM	4.102266	-0.42518	2.32E-62	3.10E-61

Gene names	logFC	log2CPM	P.Value	adj.P.Val
NXPH1	4.0937071	2.919136	2.85E-15	7.58E-15
C1orf105	4.0935619	-1.17724	4.77E-22	1.72E-21
HIST2H2AB	4.0853373	-1.51283	5.07E-25	2.08E-24
IFNL3	4.0836569	-3.26044	1.49E-27	6.81E-27
IVL	4.0809497	0.392772	1.12E-16	3.19E-16
MSLNL	4.0733187	-1.27491	7.29E-30	3.68E-29
CCKBR	4.0653262	0.180554	2.43E-16	6.82E-16
GAL3ST2	4.0592299	0.025599	5.81E-34	3.45E-33
MAGEC1	4.0572836	-0.28955	2.92E-14	7.39E-14
SBK2	4.0524357	-1.94357	2.97E-21	1.04E-20
NKAIN1	4.0509391	5.558319	7.87E-45	6.64E-44
CRISP3	4.0365407	6.107477	2.15E-16	6.05E-16
PKMYT1	4.0332204	4.299486	1.93E-137	9.02E-136
VSTM2A	4.0305531	4.679267	6.59E-13	1.56E-12
BPIFB1	4.0197928	5.510656	1.82E-27	8.28E-27
MEPE	4.0194169	0.109018	2.51E-16	7.03E-16
PGLYRP3	4.0154825	-2.25163	2.98E-12	6.78E-12
CNTNAP2	4.0045981	5.289257	2.71E-39	1.92E-38
SLC5A8	3.997463	3.440613	9.42E-16	2.57E-15
UNC13A	3.9961377	2.219897	1.10E-36	7.14E-36
PRDM13	3.9958625	-2.24182	1.09E-11	2.40E-11
CAPSL	3.9898535	0.415758	3.50E-35	2.16E-34
KREMEN2	3.9885231	1.843539	4.36E-63	5.92E-62
TMEM215	3.984329	-0.89425	8.46E-32	4.62E-31
GPR139	3.9588882	-1.23611	2.82E-15	7.51E-15
KCNH6	3.9449689	-0.71995	4.58E-23	1.72E-22
MSLN	3.9431837	4.802495	3.21E-21	1.12E-20
DQX1	3.9232688	1.232965	4.66E-50	4.51E-49

Gene names	logFC	log2CPM	P.Value	adj.P.Val
EPO	3.9099044	0.036051	2.61E-28	1.23E-27
PRSS33	3.9039879	0.891649	1.09E-11	2.41E-11
HIST1H2BF	3.8989286	0.053744	2.20E-38	1.52E-37
TGM4	3.8921402	0.50288	2.50E-17	7.34E-17
UGT2B4	3.8920251	3.037676	2.70E-12	6.15E-12
CLDN25	3.8861935	-2.81054	2.16E-27	9.85E-27
ROS1	3.8850951	0.448675	1.82E-18	5.59E-18
TMEM82	3.8822412	-2.26593	2.34E-20	7.82E-20
KIF4A	3.8749776	4.784345	9.84E-139	4.68E-137
MYBL2	3.8710388	5.862946	3.40E-81	6.55E-80
FGF5	3.8592497	-1.58245	4.73E-28	2.21E-27
PITX1	3.8590698	5.065144	8.00E-67	1.18E-65
VAX1	3.8504644	-1.90683	2.27E-11	4.93E-11
SLC6A4	3.847254	3.881045	2.97E-26	1.29E-25
PSAPL1	3.8439528	-1.02798	2.08E-08	3.88E-08
LRRC15	3.8439159	7.1266	1.91E-82	3.75E-81
TMEM145	3.8437561	2.171157	2.83E-47	2.56E-46
PGLYRP4	3.8360419	-0.8427	8.90E-20	2.89E-19
VGF	3.832961	0.76677	5.79E-33	3.30E-32
INA	3.8311529	0.552283	5.81E-20	1.90E-19
LHFPL5	3.8208272	-0.45673	1.37E-34	8.32E-34
IRS4	3.8194149	1.498859	1.28E-10	2.68E-10
NUF2	3.8087768	4.147862	9.54E-118	3.39E-116
GABBR2	3.7901127	1.423566	1.22E-18	3.77E-18
GLYATL3	3.7874747	-0.88015	2.83E-07	4.93E-07
TDO2	3.7850848	2.286074	1.07E-63	1.48E-62
HIST1H3C	3.7731561	-1.8011	1.30E-31	7.07E-31
SCT	3.7719637	-0.56499	1.44E-65	2.09E-64
PRSS1	3.7674805	0.057105	7.76E-14	1.92E-13

Gene names	logFC	log2CPM	P.Value	adj.P.Val
FAM196A	3.762859	3.84598	1.79E-30	9.30E-30
CXCL11	3.760373	3.864089	4.57E-55	5.06E-54
TMEM151A	3.7567504	-0.96371	4.29E-30	2.19E-29
SOHLH1	3.7436256	-1.93231	1.94E-15	5.21E-15
COL9A1	3.7390226	1.683992	6.48E-16	1.78E-15
IGF2BP1	3.7381467	-0.11052	6.15E-30	3.11E-29
NMU	3.7367887	1.073181	2.25E-32	1.26E-31
IQGAP3	3.7351715	5.305744	8.56E-127	3.46E-125
SPINK4	3.7257334	-0.72329	1.51E-25	6.35E-25
HCN2	3.7255975	0.892709	9.70E-58	1.15E-56
OR56A3	3.7253092	-2.93865	6.97E-10	1.41E-09
TRPM5	3.7246082	-1.51923	1.35E-24	5.45E-24
HIST1H2BE	3.7224179	-1.24781	6.85E-53	7.14E-52
SEZ6	3.7203506	1.479549	6.41E-18	1.93E-17
LIX1	3.7161013	-1.53732	1.58E-26	6.91E-26
GATA5	3.7154208	0.299516	2.14E-17	6.29E-17
EPHA8	3.7071394	0.420608	1.02E-27	4.70E-27
TUBB3	3.7044875	1.942402	1.02E-61	1.34E-60
CCL7	3.6915339	-0.69866	1.79E-24	7.18E-24
HTR1E	3.6710892	-2.46751	1.79E-16	5.04E-16
MELK	3.6696368	4.390357	4.44E-105	1.25E-103
APOBEC4	3.6654472	-2.3275	6.20E-16	1.70E-15
HTR1D	3.644509	0.347401	3.83E-55	4.24E-54
TNNI3	3.6415121	0.801111	2.08E-25	8.70E-25
HIST1H1D	3.6378296	0.554559	2.85E-30	1.47E-29
CXCL10	3.6255098	5.766052	2.00E-53	2.11E-52
AMTN	3.6243149	0.092725	2.30E-13	5.54E-13
SERPINB3	3.623838	0.604102	3.17E-07	5.49E-07

Gene names	logFC	log2CPM	P.Value	adj.P.Val
SERPINB7	3.6217971	1.217552	1.55E-23	5.96E-23
ASIC5	3.6174451	-3.71494	3.40E-18	1.04E-17
HIST1H2AD	3.6141185	0.811723	2.90E-48	2.69E-47
EMX1	3.613301	1.392321	2.98E-30	1.53E-29
LHX9	3.6107569	-1.37515	3.23E-17	9.43E-17
BIRC5	3.6095554	5.593623	1.01E-91	2.29E-90
TROAP	3.6082415	3.86929	7.97E-113	2.60E-111
TPX2	3.60745	6.297519	7.22E-122	2.70E-120
KIF18B	3.6044621	3.613083	2.13E-104	6.00E-103
HOXC11	3.5958522	3.187827	1.06E-42	8.42E-42
VIL1	3.5918223	-0.23429	2.10E-22	7.70E-22
BPIFB4	3.5905589	-1.59592	4.80E-12	1.08E-11
CYP2A6	3.5895132	5.71599	1.52E-13	3.69E-13
MAGEA11	3.5852433	-1.47791	7.19E-13	1.69E-12
S100A8	3.579513	6.256147	7.65E-21	2.61E-20
ENSG00000274997	3.5771556	-2.00195	4.59E-20	1.51E-19
HIST1H2BL	3.5746164	-1.36819	1.64E-34	9.94E-34
CDC20	3.5735874	5.330325	2.62E-92	6.00E-91
TTR	3.5735406	-1.47732	4.47E-14	1.12E-13
CCDC78	3.5681627	2.964913	3.52E-68	5.37E-67
AOC1	3.566302	2.027948	1.24E-29	6.20E-29
ISG15	3.5659117	6.954037	2.44E-55	2.72E-54
ASPM	3.5653592	5.033877	3.53E-101	9.32E-100
DIO1	3.5641103	3.636374	1.70E-28	8.12E-28
HJURP	3.5596805	3.946204	9.51E-119	3.44E-117
DMRT1	3.5573232	-1.96174	1.96E-14	5.00E-14
SEZ6L	3.5540551	3.449609	8.20E-22	2.92E-21
HIST1H1T	3.5539639	-2.91358	1.35E-30	7.03E-30
WISP1	3.5537057	4.317411	2.04E-107	6.05E-106

Gene names	logFC	log2CPM	P.Value	adj.P.Val
C11orf86	3.5491606	-1.06454	1.82E-11	3.97E-11
SPRR2D	3.5475786	-0.47542	3.01E-06	4.91E-06
KIF14	3.544271	3.646894	1.00E-93	2.35E-92
TOP2A	3.5411952	7.365687	6.39E-91	1.43E-89
PPP2R2C	3.5392032	4.527827	3.49E-36	2.23E-35
FAM83D	3.5367316	4.259198	2.15E-92	4.93E-91
A2ML1	3.5303846	3.861314	3.09E-21	1.08E-20
OFCC1	3.5274054	-1.85453	3.37E-13	8.07E-13
C2orf54	3.5269225	4.129683	5.78E-27	2.58E-26
HIST1H2BH	3.5182919	0.959406	8.64E-51	8.57E-50
IFITM5	3.5182273	-1.14553	9.22E-13	2.16E-12
C6orf222	3.5177261	-2.8028	1.67E-22	6.15E-22
RAD21L1	3.5073241	-1.66263	3.63E-14	9.14E-14
HAGHL	3.5048154	3.669664	1.95E-88	4.21E-87
FOXM1	3.5005323	5.535286	7.64E-94	1.79E-92
PBK	3.4941858	3.87155	2.87E-109	8.74E-108
CDH7	3.492292	1.016986	1.51E-20	5.10E-20
HIST1H3D	3.4886702	1.228754	5.52E-53	5.76E-52
IL20	3.486274	2.389951	1.06E-25	4.51E-25
CILP2	3.481239	4.047444	6.50E-86	1.36E-84
PSORS1C2	3.479532	0.146396	4.12E-18	1.25E-17
EXO1	3.4747082	3.860925	2.94E-102	7.93E-101
CCDC60	3.4701475	-1.17551	1.77E-16	4.99E-16
PCDH10	3.4659271	3.406018	5.54E-17	1.60E-16
CEP55	3.4650389	4.575039	6.07E-111	1.92E-109
PLK1	3.4576159	4.942108	9.24E-111	2.91E-109
SVOP	3.4510954	-1.16584	8.89E-13	2.08E-12
CYP2A13	3.4464812	-1.17338	2.37E-12	5.43E-12

Gene names	logFC	log2CPM	P.Value	adj.P.Val
RRM2	3.4462466	5.789649	1.85E-103	5.14E-102
INHBA	3.4355931	6.284377	3.43E-112	1.11E-110
CYP2F1	3.4347475	-1.85611	1.04E-15	2.83E-15
NEIL3	3.4315751	1.836703	1.72E-106	4.98E-105
AURKB	3.4283642	3.782321	2.45E-88	5.26E-87
NPY	3.4213498	-0.78372	6.38E-09	1.22E-08
CDC25C	3.4212472	2.606257	1.02E-127	4.18E-126
KIF20A	3.414135	4.897167	3.22E-111	1.02E-109
EFNA2	3.411069	-2.72552	1.64E-23	6.29E-23
SKA1	3.4090176	3.070539	1.33E-99	3.42E-98
KRTDAP	3.4063592	1.609078	3.76E-13	8.99E-13
ANLN	3.4048897	5.522175	2.62E-98	6.58E-97
KRT2	3.4043914	-1.65699	1.06E-19	3.43E-19
CLVS2	3.4040615	1.015227	4.85E-10	9.89E-10
ZPLD1	3.400571	1.753017	3.10E-23	1.18E-22
COL2A1	3.3982695	6.260137	1.38E-16	3.92E-16
VCX	3.3951024	-2.25481	2.45E-12	5.60E-12
GPR115	3.3926548	-0.17491	1.12E-27	5.14E-27
CLPSL2	3.3889376	0.628154	2.94E-46	2.58E-45
UHRF1	3.3858009	4.452183	2.94E-124	1.15E-122
ITPKA	3.3829939	0.592547	5.99E-54	6.42E-53
RIMS4	3.3806572	4.97561	8.65E-28	4.00E-27
RIIAD1	3.3795734	-0.015	7.56E-40	5.46E-39
ONECUT2	3.3787425	2.001226	4.19E-26	1.80E-25
SCN1A	3.3773564	0.036847	2.05E-12	4.70E-12
CKAP2L	3.3700514	3.618921	1.07E-114	3.62E-113
CNIH2	3.3676138	2.310654	1.19E-78	2.20E-77
GRIA2	3.3643799	4.564683	2.98E-15	7.93E-15
CRISP1	3.3624692	-2.77809	3.53E-13	8.44E-13

Gene names	logFC	log2CPM	P.Value	adj.P.Val
SOX2	3.3599578	1.19254	3.39E-17	9.88E-17
KIF2C	3.3599355	4.813602	1.66E-102	4.52E-101
KIAA0101	3.3484393	4.275102	8.65E-134	3.85E-132
MYO3B	3.3484274	2.638398	2.54E-24	1.01E-23
MMP9	3.3458204	7.340071	9.05E-35	5.53E-34
IFNB1	3.3437664	-3.62482	6.58E-23	2.47E-22
NAT16	3.3417373	-2.74482	5.95E-19	1.86E-18
TEX15	3.3415506	-0.94862	3.11E-11	6.71E-11
GAD1	3.3401526	1.608995	6.43E-26	2.75E-25
DLGAP5	3.3356291	4.168861	3.60E-107	1.06E-105
CADPS	3.3352442	1.072343	3.83E-33	2.20E-32
KRT37	3.3331654	-1.42499	2.82E-29	1.39E-28
ELAVL3	3.3322125	-0.72016	1.83E-15	4.92E-15
BUB1	3.3271975	4.7612	4.34E-111	1.38E-109
CENPA	3.308081	2.942023	1.06E-85	2.22E-84
TMEM179	3.3074044	0.554599	1.25E-15	3.37E-15
NELL2	3.2998288	5.679236	5.72E-33	3.26E-32
SGOL1	3.2994411	2.657683	3.76E-102	1.01E-100
CENPF	3.2986609	6.848016	2.63E-111	8.39E-110
CLEC5A	3.2940666	2.766499	2.25E-76	4.01E-75
DEPDC1	3.2887474	3.79017	1.07E-85	2.24E-84
LECT1	3.286675	0.123894	1.73E-10	3.60E-10
MISP	3.2796214	5.404319	4.58E-56	5.20E-55
MS4A8	3.2788527	1.047232	3.88E-18	1.18E-17
NAT1	3.2783433	7.108365	1.68E-35	1.05E-34
POU4F1	3.2683887	-1.05897	6.17E-12	1.38E-11
ATP1A3	3.2681951	2.087646	1.21E-36	7.82E-36
AIM1L	3.2618625	1.681693	1.29E-65	1.87E-64

Gene names	logFC	log2CPM	P.Value	adj.P.Val
GABRD	3.2604365	1.941009	6.13E-103	1.68E-101
UBE2T	3.2587044	4.558047	1.65E-123	6.34E-122
HAO1	3.2525564	-2.227	1.10E-08	2.08E-08
SALL3	3.2517388	-2.13737	2.65E-21	9.26E-21
PTTG1	3.2501319	4.593944	1.36E-106	3.95E-105
TFR2	3.2497421	1.881387	6.61E-61	8.47E-60
ARTN	3.2490777	2.259954	4.40E-58	5.24E-57
FADS6	3.2484729	-1.56932	1.23E-16	3.48E-16
ZNF695	3.2458732	1.337643	3.73E-60	4.71E-59
NDC80	3.2386875	4.019861	5.43E-101	1.43E-99
HHIPL2	3.2379807	1.719763	2.93E-30	1.50E-29
CDH18	3.236179	-0.56911	1.70E-12	3.92E-12
IGFL2	3.2339464	0.858396	3.83E-41	2.90E-40
FCRLB	3.2335951	2.971113	1.49E-28	7.13E-28
RUNDC3A	3.2311144	2.322882	7.66E-25	3.12E-24
FGG	3.2291189	3.495278	4.28E-08	7.82E-08
CTNNA2	3.2268983	0.167257	2.77E-15	7.38E-15
UNC93A	3.2211578	-2.92479	1.81E-14	4.64E-14
CCR8	3.2146456	-0.33211	1.73E-46	1.53E-45
GFY	3.213727	-2.40925	2.03E-13	4.91E-13
MNX1	3.2119444	0.864652	2.22E-30	1.15E-29
KRT3	3.2114059	-2.94964	9.72E-20	3.15E-19
CFHR4	3.2098227	-3.4026	8.34E-10	1.68E-09
CEMIP	3.2093771	4.449241	1.87E-61	2.44E-60
SYNGR3	3.2064441	1.652972	5.70E-61	7.33E-60
RIPPLY3	3.2062042	1.796954	5.89E-57	6.83E-56
NBPF4	3.2055379	1.15981	7.02E-23	2.63E-22
ERVV_1	3.2031223	-1.9906	9.58E-17	2.73E-16
SPRR1B	3.1978404	0.890997	4.75E-07	8.17E-07

Gene names	logFC	log2CPM	P.Value	adj.P.Val
KIFC1	3.1928938	5.153666	2.96E-109	9.01E-108
NKX2_3	3.19053	-2.42364	5.24E-11	1.12E-10
SPC24	3.1903412	3.52118	9.98E-111	3.14E-109
CDK1	3.1898075	5.531286	2.78E-117	9.69E-116
HIST3H2BB	3.1821104	1.529169	4.19E-46	3.67E-45
CXCL9	3.1776477	6.694854	3.09E-37	2.05E-36
MCM10	3.174869	3.82452	3.13E-75	5.47E-74
SCRT1	3.1736226	-1.24786	6.88E-23	2.58E-22
DMBX1	3.1695407	-0.42669	9.39E-37	6.10E-36
CST9L	3.1691555	-1.20131	3.75E-12	8.48E-12
LPPR5	3.1634953	-1.61488	2.51E-36	1.61E-35
RAB26	3.1585351	3.385602	1.04E-68	1.61E-67
IL17REL	3.1553758	0.077043	7.88E-32	4.31E-31
NBPF6	3.1536781	0.608186	1.71E-22	6.27E-22
AURKA	3.152959	4.693811	7.74E-106	2.23E-104
KLK12	3.1517377	1.525433	2.35E-10	4.87E-10
CAMK2N2	3.1515933	0.051744	1.45E-50	1.43E-49
TGFBR3L	3.1498207	-0.56652	4.61E-37	3.04E-36
CSTL1	3.1486733	-3.02863	2.30E-18	7.04E-18
ZIC3	3.1466968	-2.41211	2.22E-11	4.83E-11
SYNGR4	3.1460853	-1.50154	1.44E-46	1.27E-45
MMP10	3.1453667	2.395615	1.73E-28	8.23E-28
TUBA3E	3.1411875	0.946158	5.04E-22	1.82E-21
AKAP14	3.1408944	-2.18211	3.47E-17	1.01E-16
TCF24	3.1398037	-1.81191	1.90E-17	5.61E-17
CDCA5	3.1304241	4.84449	1.89E-99	4.86E-98
ADAMTS14	3.1249598	2.783043	3.43E-86	7.22E-85
TK1	3.1228621	6.096078	1.54E-100	4.03E-99

Gene names	logFC	log2CPM	P.Value	adj.P.Val
MKI67	3.1212427	6.693326	2.86E-92	6.51E-91
ENSG00000260234	3.1200103	-2.97981	1.15E-21	4.09E-21
ITLN2	3.1183546	-2.82501	3.72E-13	8.89E-13
PAX7	3.1172283	1.613248	1.76E-09	3.48E-09
COL22A1	3.1167355	3.178241	1.33E-35	8.37E-35
BRINP3	3.1155509	3.422808	2.14E-09	4.22E-09
SKA3	3.113025	3.38058	1.24E-100	3.26E-99
OTOR	3.1081336	-1.30231	3.65E-22	1.32E-21
KLK4	3.1067996	1.726884	1.96E-42	1.54E-41
KCNK9	3.1067924	-1.24331	6.80E-29	3.30E-28
ULBP1	3.1067663	0.782075	3.61E-40	2.64E-39
NCAPG	3.1015654	4.264526	1.54E-100	4.03E-99
CXorf22	3.0989574	0.096634	9.04E-32	4.93E-31
MUC13	3.0978289	0.342122	1.16E-15	3.15E-15
ASF1B	3.0972936	4.759642	2.71E-130	1.15E-128
CDCA3	3.0950219	3.986207	4.46E-97	1.09E-95
S100A7	3.0948398	5.940913	2.56E-09	5.01E-09
FBN2	3.0907448	3.705229	1.97E-44	1.64E-43
RDM1	3.0895983	0.08209	2.65E-82	5.20E-81
NUSAP1	3.0856795	5.496546	2.20E-123	8.38E-122
FAM64A	3.0842076	2.921829	9.16E-81	1.76E-79
CIB3	3.0837803	-0.64372	1.71E-40	1.27E-39
FOXD3	3.0811605	-0.58	2.10E-25	8.78E-25
DTL	3.0810075	4.927645	2.75E-116	9.48E-115
HIST2H2AC	3.0783814	0.650514	8.73E-37	5.69E-36
HMMR	3.0782064	3.933608	9.12E-112	2.95E-110
TBR1	3.0770553	-2.09716	2.28E-13	5.51E-13
CCNB2	3.0769785	4.632488	2.54E-99	6.50E-98
GTSE1	3.0747218	3.674791	1.22E-98	3.08E-97

Gene names	logFC	log2CPM	P.Value	adj.P.Val
DMRTA2	3.0746611	-1.96398	5.58E-14	1.39E-13
NT5DC4	3.0744606	-1.98823	1.64E-34	9.92E-34
SIX3	3.0740438	1.588413	1.29E-18	3.98E-18
PSG9	3.0720633	-1.68767	8.69E-12	1.93E-11
CNTD2	3.0685144	3.035155	1.36E-44	1.14E-43
HIST1H3G	3.0665563	0.615231	1.38E-30	7.18E-30
ZIC5	3.0663889	-0.66588	8.85E-11	1.87E-10
SOX11	3.0611165	4.053435	2.46E-26	1.07E-25
CDCA8	3.0610316	4.541371	3.17E-102	8.54E-101
MALRD1	3.0598063	2.740396	1.33E-17	3.96E-17
ZNF716	3.0589417	-2.45475	2.57E-09	5.03E-09
IL36RN	3.0556803	-1.73014	2.40E-13	5.79E-13
SSTR5	3.05402	-2.78988	1.07E-11	2.37E-11
CLPSL1	3.051064	1.465602	4.13E-30	2.10E-29
KCNH1	3.0467154	1.585932	7.42E-32	4.06E-31
DYDC1	3.0430371	-3.13315	9.02E-20	2.93E-19
IRG1	3.0424519	-3.39941	7.24E-20	2.36E-19
HRH3	3.0407376	-1.85815	4.46E-14	1.12E-13
CDC6	3.0381471	4.93392	3.52E-66	5.16E-65
HSD17B6	3.027632	2.42319	4.45E-135	2.00E-133
CLPS	3.0189662	-1.23155	1.16E-26	5.12E-26
NGB	3.009205	-2.22472	1.90E-15	5.08E-15
IFNL1	3.0007677	-2.45393	7.44E-23	2.78E-22
HOXC13	2.9978246	3.526575	2.31E-45	1.98E-44
FOXD1	2.9933174	0.832949	1.59E-25	6.66E-25
SYT1	2.9921364	5.217616	2.78E-24	1.10E-23
BUB1B	2.9887354	4.340553	4.66E-107	1.37E-105
NEU4	2.9877034	-0.09792	3.12E-20	1.04E-19

Gene names	logFC	log2CPM	P.Value	adj.P.Val
ESM1	2.9876078	3.424591	1.12E-86	2.38E-85
KCNJ6	2.9835207	0.259218	1.91E-27	8.69E-27
GDF9	2.981166	3.038154	9.06E-23	3.37E-22
DEPDC1B	2.9766497	2.685176	3.02E-94	7.14E-93
LAMP5	2.9747543	3.90645	5.92E-64	8.24E-63
NPBWR1	2.9739299	-2.80678	1.66E-13	4.04E-13
CD1A	2.9732739	0.865965	4.57E-23	1.72E-22
FAM72D	2.9666032	-0.47776	4.08E-61	5.27E-60
PCSK1N	2.9645086	2.247959	9.60E-20	3.11E-19
CDCA2	2.9619846	3.296118	1.69E-70	2.72E-69
ZBBX	2.9618977	-1.4736	2.64E-19	8.41E-19
CARD17	2.9599178	-2.35443	1.39E-24	5.60E-24
TNFRSF18	2.9597413	3.242169	1.76E-51	1.78E-50
RET	2.9593165	6.111499	5.46E-34	3.25E-33
ZIC2	2.9587617	1.297709	2.38E-19	7.61E-19
GRM8	2.9577982	-0.18789	2.90E-68	4.44E-67
NKX3_2	2.9570624	0.259745	2.01E-47	1.82E-46
NKX2_5	2.9474209	0.064543	3.49E-11	7.51E-11
PAX1	2.9455689	0.07854	5.49E-21	1.89E-20
CDKN3	2.944302	3.365767	1.69E-110	5.29E-109
CA10	2.9442749	-0.72595	1.44E-08	2.71E-08
SLURP1	2.9416829	-0.58243	8.32E-15	2.16E-14
CDC45	2.9374272	3.456632	3.57E-84	7.25E-83
KLHDC7B	2.9372844	4.362252	2.33E-26	1.01E-25
TUBA3D	2.9331237	2.776668	1.84E-24	7.35E-24
PCSK2	2.9288819	1.475416	2.14E-09	4.22E-09
GNG3	2.9286012	-0.25776	7.64E-30	3.85E-29
ENSG00000146197	2.9272994	4.508129	4.81E-35	2.96E-34
KRT24	2.9271452	-0.29963	1.81E-10	3.77E-10

Gene names	logFC	log2CPM	P.Value	adj.P.Val
TMPRSS4	2.9252433	3.103103	2.42E-19	7.72E-19
TNR	2.9244453	0.687602	6.52E-28	3.03E-27
IFI6	2.9230645	8.570212	1.40E-45	1.21E-44
CHRNA4	2.9213016	-1.54386	3.75E-11	8.07E-11
GPRIN1	2.9209861	3.362501	3.48E-100	9.06E-99
HES6	2.9195511	3.241198	5.64E-65	8.05E-64
E2F7	2.9185308	2.494833	7.96E-73	1.33E-71
CTXN1	2.9127308	4.679813	1.28E-79	2.40E-78
TFF1	2.9092841	7.682038	8.86E-16	2.42E-15
FSD1	2.9079633	-0.25168	1.90E-25	7.94E-25
GDPD2	2.9066671	0.195684	1.91E-24	7.64E-24
CLSPN	2.9046224	3.036173	2.57E-79	4.81E-78
IGFALS	2.9027917	2.187415	7.53E-31	3.97E-30
PADI3	2.9023478	1.463845	4.83E-16	1.33E-15
TTK	2.9021735	4.030685	1.29E-79	2.43E-78
HIST2H4A	2.9009098	-0.7162	6.29E-49	5.92E-48
CCNA2	2.900081	4.619125	2.02E-91	4.57E-90
FAM57B	2.9000341	1.248184	5.60E-39	3.93E-38
PRLH	2.8988945	-3.20477	2.43E-23	9.23E-23
MUC5B	2.8986056	6.549274	4.86E-20	1.60E-19
MKRN3	2.8981769	0.614564	1.28E-17	3.80E-17
WDR38	2.8963754	-1.28729	2.65E-21	9.26E-21
ARMC3	2.8960061	1.449234	1.83E-35	1.14E-34
FAM83A	2.8954736	3.069346	1.92E-20	6.44E-20
ARL14	2.8925337	-2.92837	1.86E-14	4.75E-14
FN1	2.8896091	12.22124	2.72E-77	4.92E-76
BRSK2	2.8893806	1.470608	4.44E-30	2.26E-29
F12	2.8863104	3.172921	5.94E-65	8.49E-64

Gene names	logFC	log2CPM	P.Value	adj.P.Val
CALHM1	2.8846796	-1.48003	9.04E-28	4.18E-27
HS6ST3	2.8833631	3.664103	8.58E-25	3.49E-24
ERCC6L	2.8811993	2.825744	9.72E-103	2.67E-101
C16orf59	2.8765774	2.946628	3.47E-109	1.05E-107
ASCL4	2.8747572	-1.88115	8.02E-18	2.41E-17
MATN3	2.8743869	4.743913	3.07E-59	3.78E-58
SHH	2.8720611	-1.17742	1.46E-17	4.33E-17
UMODL1	2.8689505	0.761524	3.53E-33	2.03E-32
MTFR2	2.866889	2.034397	2.80E-86	5.91E-85
S100A9	2.8628313	7.488531	6.37E-18	1.92E-17
SYNDIG1	2.8591972	2.921046	7.14E-53	7.44E-52
SCG2	2.8589264	3.237286	1.79E-22	6.59E-22
GNG4	2.8584281	3.497106	5.27E-22	1.90E-21
ENTHD1	2.8577801	-1.30272	6.25E-41	4.69E-40
CASC5	2.8573697	3.884987	1.32E-101	3.50E-100
CENPM	2.8502874	3.172393	2.80E-93	6.48E-92
LKAAEAR1	2.849142	-2.49394	3.68E-29	1.81E-28
GPR6	2.8481743	-2.62119	2.09E-09	4.11E-09
CCDC185	2.8478677	-2.18121	2.08E-24	8.30E-24
SPC25	2.8456441	2.886919	1.24E-131	5.41E-130
BEX1	2.8428605	3.772502	8.50E-13	1.99E-12
SPAG5	2.8428481	5.196114	2.97E-97	7.32E-96
FAM111B	2.8426795	4.665044	1.12E-73	1.90E-72
FAM216B	2.8422671	-2.39957	3.19E-15	8.47E-15
OPRD1	2.8422534	1.70197	2.33E-28	1.11E-27
SLC18A1	2.8380958	-1.39498	7.79E-16	2.13E-15
ELFN2	2.8379892	0.660289	9.37E-23	3.48E-22
RECQL4	2.8361439	4.900213	1.55E-90	3.45E-89
LGALS9B	2.8356215	-1.11756	1.09E-20	3.71E-20

Gene names	logFC	log2CPM	P.Value	adj.P.Val
CCNE1	2.8353672	3.154711	4.15E-45	3.53E-44
KIF26B	2.8350851	5.152525	1.89E-80	3.60E-79
COX6C	2.8341927	9.229103	1.44E-52	1.49E-51
F7	2.8322268	3.064901	1.90E-28	9.07E-28
SALL4	2.8294801	1.055785	1.86E-72	3.09E-71
KCNG3	2.8241975	-0.77349	5.48E-35	3.37E-34
RAD54L	2.823505	2.903333	4.37E-84	8.87E-83
NCAPH	2.8206994	4.008964	2.49E-95	5.98E-94
TERT	2.8192693	-0.88124	2.23E-30	1.15E-29
GCNT3	2.8189882	0.683211	3.60E-20	1.19E-19
NTNG1	2.8178511	2.233255	3.83E-16	1.06E-15
C19orf33	2.8170929	4.173668	2.94E-31	1.57E-30
DLL3	2.8109111	-0.46532	2.29E-21	8.01E-21
NKX6_1	2.806846	1.195105	9.26E-30	4.65E-29
ADAMDEC1	2.7991369	3.222372	5.47E-33	3.12E-32
CDKN2A	2.7987594	4.395499	2.68E-39	1.90E-38
PRR11	2.798636	4.451813	3.82E-56	4.34E-55
LRFN2	2.798512	0.003748	3.79E-38	2.59E-37
JAKMIP1	2.7927949	0.945862	7.27E-45	6.14E-44
SLITRK1	2.7895579	0.238643	3.41E-09	6.63E-09
FGB	2.7867587	3.85177	1.17E-06	1.97E-06
CLDN9	2.7859278	0.820102	4.30E-32	2.37E-31
TMEM190	2.7848332	-1.88844	3.02E-32	1.68E-31
PEX5L	2.7830851	2.456777	1.53E-19	4.90E-19
SNTN	2.7810232	-1.11708	4.94E-19	1.55E-18
PAPL	2.7803657	-1.1577	5.44E-17	1.57E-16
C4orf51	2.7790141	-2.42735	1.63E-15	4.39E-15
ESPL1	2.771852	4.240301	2.85E-92	6.49E-91

Gene names	logFC	log2CPM	P.Value	adj.P.Val
STXBP5L	2.7695944	-0.70803	3.80E-18	1.15E-17
MAPK8IP2	2.7676107	5.08521	5.80E-50	5.61E-49
AMER2	2.7675797	-0.92556	5.46E-09	1.05E-08
CCDC64	2.7671637	4.614178	1.46E-105	4.19E-104
CDSN	2.7550198	-0.36222	1.72E-16	4.84E-16
KISS1	2.7469816	-1.50923	5.91E-29	2.88E-28
MT3	2.7446253	-1.81112	5.79E-17	1.67E-16
AMH	2.7437028	1.188888	3.99E-32	2.21E-31
ESCO2	2.7435771	2.933644	7.00E-90	1.54E-88
ROBO2	2.7422735	3.677128	5.03E-19	1.58E-18
CLEC6A	2.7414545	-2.54402	2.30E-19	7.35E-19
CAPN9	2.7409044	3.109743	4.84E-36	3.07E-35
MCIDAS	2.737798	1.011928	6.97E-25	2.85E-24
FSIP1	2.7373485	4.20106	3.70E-29	1.82E-28
PACSIN1	2.7328409	2.234485	2.10E-75	3.68E-74
LYPD4	2.7312449	-2.62074	1.30E-11	2.86E-11
FAM72C	2.7302359	-1.50118	1.08E-38	7.51E-38
HIST2H2BF	2.7293994	1.83037	5.83E-38	3.96E-37
MAPK15	2.7291674	4.050232	5.61E-38	3.81E-37
LYPD8	2.7291229	-0.04092	7.27E-17	2.09E-16
PNMA5	2.72889	-0.61448	8.72E-12	1.94E-11
HIST1H2BC	2.7282156	3.048924	8.54E-44	6.99E-43
CBX2	2.7246784	4.686665	2.35E-43	1.90E-42
CFAP45	2.724076	2.622054	2.37E-60	3.00E-59
HIST1H2BD	2.7217597	5.43301	9.51E-66	1.38E-64
MND1	2.7202237	2.022428	5.07E-71	8.26E-70
PLA2G2F	2.7168835	-2.85027	1.02E-14	2.64E-14
C1orf94	2.7166739	-2.16182	1.75E-07	3.08E-07
GSTA3	2.7157345	-0.87819	4.03E-11	8.66E-11

Gene names	logFC	log2CPM	P.Value	adj.P.Val
SAMD11	2.7152341	3.056375	1.86E-33	1.08E-32
ENTPD8	2.7145393	2.053085	1.54E-30	8.03E-30
RDH16	2.7123433	1.769835	3.88E-36	2.47E-35
IL4I1	2.7116912	3.479872	4.63E-57	5.39E-56
TNFRSF9	2.7093133	1.760212	6.54E-50	6.30E-49
PYY	2.7080166	0.211865	8.89E-22	3.16E-21
HSH2D	2.7079387	4.394554	3.40E-48	3.14E-47
HTR3A	2.7069838	-0.6121	4.97E-14	1.24E-13
GP2	2.702112	5.476546	1.49E-14	3.81E-14
S100A14	2.7009044	7.76147	3.75E-47	3.38E-46
VSTM2L	2.7004444	2.555976	1.59E-25	6.68E-25
RHBG	2.6992317	0.385855	3.78E-34	2.26E-33
KIAA1211	2.6983216	2.742321	1.14E-49	1.09E-48
AMZ1	2.6971207	2.158597	7.52E-37	4.92E-36
RMI2	2.6962968	4.097904	8.40E-97	2.05E-95
RAMP1	2.693353	4.285059	3.44E-34	2.06E-33
TRIM15	2.6903092	-2.85881	5.76E-12	1.29E-11
CXCL17	2.6900947	4.785338	8.06E-17	2.31E-16
CKS2	2.6892351	5.064424	2.07E-112	6.71E-111
UPK1A	2.6888695	0.956523	9.69E-17	2.76E-16
E2F1	2.6885275	4.540814	2.60E-83	5.18E-82
E2F8	2.6884582	2.728615	2.29E-55	2.56E-54
CALML5	2.6862652	6.325344	6.82E-14	1.69E-13
DMRTC2	2.6858248	-1.28992	4.75E-11	1.02E-10
HMX3	2.6839158	-2.69749	4.58E-09	8.84E-09
PRC1	2.6837674	5.808422	6.48E-99	1.65E-97
SLC8A2	2.6794183	0.887727	5.72E-21	1.97E-20
HIST1H4H	2.6778141	3.059591	1.17E-37	7.87E-37

Gene names	logFC	log2CPM	P.Value	adj.P.Val
SERPINA9	2.6738343	0.174219	2.40E-21	8.41E-21
CLGN	2.6727857	4.342227	1.40E-32	7.86E-32
MOG	2.6725015	-1.21283	2.58E-09	5.05E-09
KIF23	2.6655318	4.819919	1.52E-102	4.15E-101
USP41	2.6647467	-1.65918	7.17E-23	2.68E-22
TRIML2	2.6642284	-2.28644	7.69E-15	2.00E-14
CST6	2.6637708	2.050618	2.85E-30	1.46E-29
NPHS1	2.6636521	-1.47851	5.73E-20	1.88E-19
HOXB9	2.6614621	1.434056	8.75E-31	4.60E-30
ELAVL2	2.6609943	1.804905	1.12E-33	6.55E-33
APLP1	2.6582163	3.936081	1.28E-31	6.94E-31
HMGA2	2.6578273	0.066995	1.27E-24	5.13E-24
FDCSP	2.6530867	7.458842	1.68E-08	3.15E-08
CACNA1B	2.6526706	0.246236	2.27E-21	7.96E-21
IGF2BP3	2.6511664	0.187041	1.06E-17	3.16E-17
RHBDL1	2.6511319	2.197846	3.25E-48	3.00E-47
IL17C	2.6507782	-3.04081	1.10E-19	3.57E-19
EME1	2.6495025	2.446786	2.04E-76	3.64E-75
KCNE4	2.6483535	6.294648	2.20E-29	1.09E-28
LYZL2	2.6483065	-2.57625	1.88E-12	4.32E-12
CENPE	2.648005	4.355108	3.17E-85	6.52E-84
POLQ	2.6478471	3.105565	6.13E-82	1.19E-80
IL21R	2.6468161	2.333943	5.91E-57	6.85E-56
TRPM8	2.6437831	0.668415	6.92E-14	1.71E-13
PRMT8	2.6391151	-1.40842	2.69E-16	7.53E-16
SHCBP1	2.637696	3.613647	1.67E-85	3.46E-84
SLC7A5	2.6325632	7.017351	3.14E-44	2.61E-43
CPNE7	2.6316384	3.310882	7.84E-37	5.12E-36
CST9	2.6296064	3.892413	1.27E-08	2.40E-08

Gene names	logFC	log2CPM	P.Value	adj.P.Val
TFF3	2.6271135	8.070318	1.06E-19	3.45E-19
MAGEA8	2.6244345	-0.52749	7.70E-15	2.00E-14
ZWINT	2.6200764	5.400588	1.03E-111	3.33E-110
NEURL1	2.619821	3.909112	3.10E-25	1.28E-24
E2F2	2.6173397	3.443652	6.00E-65	8.56E-64
HIST1H2BJ	2.6155838	1.860689	9.41E-43	7.47E-42
SHISA8	2.6152783	-2.08962	3.99E-23	1.51E-22
RAD51	2.6148176	3.317196	1.73E-99	4.45E-98
PAQR4	2.6143926	5.112732	5.65E-103	1.56E-101
TMEM249	2.6143414	-1.54676	3.03E-36	1.94E-35
TMEM92	2.614168	0.43548	6.99E-44	5.74E-43
PNCK	2.6129123	1.368846	6.51E-30	3.30E-29
APOBEC3B	2.6127569	3.462161	3.46E-41	2.62E-40
GAL	2.6121665	1.834521	8.59E-21	2.93E-20
AKR7A3	2.6052217	4.766405	7.60E-27	3.38E-26
CCNB1	2.6046037	5.697473	2.32E-115	7.95E-114
POTEJ	2.6015532	-1.72072	3.39E-15	8.98E-15
TRIP13	2.6012846	4.381222	1.62E-78	2.99E-77
GCGR	2.595912	-2.38138	3.92E-15	1.03E-14
ZNF280A	2.5927442	-3.02131	1.81E-09	3.58E-09
MC5R	2.5924669	-2.86211	1.36E-12	3.15E-12
C1orf167	2.5922165	-0.8621	2.57E-16	7.18E-16
SOX3	2.5900245	-2.66851	5.96E-09	1.14E-08
SLC44A4	2.5887054	7.000235	1.67E-34	1.01E-33
CDH2	2.5872917	3.659217	3.46E-27	1.56E-26
SLITRK6	2.5866567	6.402549	4.75E-17	1.38E-16
RALYL	2.5828413	-1.1305	2.33E-07	4.07E-07
ORC6	2.5809957	2.997198	1.15E-67	1.73E-66

Gene names	logFC	log2CPM	P.Value	adj.P.Val
ERMN	2.5809681	1.040853	4.62E-41	3.47E-40
HTR2C	2.5788973	-2.26381	1.60E-07	2.82E-07
DLX5	2.5786432	1.424901	1.06E-26	4.69E-26
FAM135B	2.5784615	1.68676	2.51E-16	7.02E-16
KIF15	2.5772859	3.634422	1.70E-83	3.39E-82
PNMT	2.5749927	3.518928	1.77E-12	4.08E-12
CFB	2.5744343	5.906559	1.34E-39	9.64E-39
LHFPL4	2.5713016	-0.28656	7.05E-12	1.57E-11
TMPRSS6	2.5703969	3.184905	8.36E-23	3.12E-22
OTX1	2.566356	2.048877	6.50E-36	4.11E-35
KCNF1	2.5630319	3.156482	1.33E-19	4.29E-19
ZMYND10	2.5624957	3.885097	7.31E-42	5.65E-41
DEGS2	2.5624285	6.252715	2.29E-37	1.52E-36
UBE2S	2.5610465	4.557898	3.99E-83	7.92E-82
SMIM22	2.5609082	5.712262	2.96E-46	2.59E-45
MUC1	2.5595567	9.158421	1.26E-42	9.94E-42
MUC21	2.5594928	-0.91572	8.39E-08	1.51E-07
PAX2	2.5524574	1.031055	4.95E-19	1.56E-18
GBP5	2.5518604	4.847339	3.35E-33	1.93E-32
KIF11	2.54635	5.308765	3.57E-103	9.84E-102
KCNK15	2.5462676	4.105952	5.48E-34	3.25E-33
EDN2	2.5456743	2.890383	6.03E-34	3.57E-33
MMP12	2.5409546	2.833782	5.36E-18	1.62E-17
COL6A5	2.5397665	-1.6819	2.27E-17	6.68E-17
LGALS9C	2.53931	-0.88703	2.40E-21	8.41E-21
LINGO1	2.536562	4.598834	6.53E-40	4.74E-39
FGA	2.5362807	2.107477	0.0001163	0.00017183
NOTUM	2.5347163	0.152254	6.60E-25	2.70E-24
ZBED2	2.5345437	0.302558	1.05E-27	4.85E-27

Gene names	logFC	log2CPM	P.Value	adj.P.Val
OTP	2.5342419	-3.30991	1.88E-15	5.04E-15
PROC	2.53353	-0.12857	1.81E-75	3.18E-74
CELSR3	2.5308246	4.001733	1.31E-47	1.19E-46
FEZF1	2.5295717	-2.98386	2.61E-10	5.38E-10
GINS1	2.5274488	4.291184	3.65E-98	9.16E-97
HUS1B	2.5246806	-0.7878	1.98E-17	5.83E-17
CYP2W1	2.5215345	-2.19728	5.82E-15	1.52E-14
GLDC	2.5210335	3.021473	2.13E-17	6.27E-17
PAFAH1B3	2.5191284	5.963251	4.36E-114	1.46E-112
CDT1	2.5147456	4.132211	7.23E-66	1.05E-64
SLC16A6	2.5136793	5.39471	6.53E-30	3.31E-29
HOXC10	2.508749	5.308587	7.18E-34	4.24E-33
TPSG1	2.508191	1.918694	3.52E-16	9.79E-16
C6orf118	2.5060309	-2.54918	1.25E-10	2.62E-10
IGFBPL1	2.5051374	-0.77843	2.10E-24	8.36E-24
MAGEB1	2.5010315	-2.12246	2.82E-06	4.60E-06
PLEKHD1	2.4991215	2.513464	2.12E-27	9.67E-27
SPIB	2.4988461	2.637598	5.43E-17	1.57E-16
NR5A1	2.4976786	-1.42544	6.00E-09	1.15E-08
ISL2	2.4943227	-1.95644	1.21E-14	3.11E-14
CCNO	2.4920577	3.725056	2.25E-24	8.94E-24
APOA1	2.4894303	-1.03013	6.79E-22	2.43E-21
SLC5A5	2.4890123	-0.20831	6.17E-18	1.86E-17
EDDM3B	2.4890071	-1.28431	2.78E-10	5.73E-10
HIST1H4K	2.4882144	-1.53745	4.87E-37	3.20E-36
TSPAN1	2.4880704	6.63794	7.99E-29	3.86E-28
SPAG6	2.4870798	2.66052	1.60E-11	3.51E-11
CTAG2	2.4868566	-0.88427	0.0002478	0.00035809

Gene names	logFC	log2CPM	P.Value	adj.P.Val
CCL20	2.4861921	0.529048	1.34E-23	5.13E-23
LMNB1	2.4852167	5.959587	1.64E-103	4.56E-102
BCAS1	2.4827173	5.45645	6.15E-26	2.63E-25
DRC7	2.4806708	-1.57367	4.79E-21	1.66E-20
TLCD1	2.480604	3.762602	5.63E-73	9.42E-72
KRT78	2.4787979	-1.84508	7.99E-08	1.44E-07
SPEF1	2.4772734	1.521409	4.89E-38	3.33E-37
NXPH4	2.4756231	2.842893	1.66E-25	6.94E-25
P4HA3	2.4755515	3.31024	3.33E-73	5.59E-72
DPEP1	2.4713507	0.660481	2.30E-45	1.97E-44
OIP5	2.4710421	1.705132	1.53E-96	3.72E-95
KIAA0319	2.4690223	0.819696	5.38E-22	1.94E-21
BARX1	2.4678842	0.897082	2.20E-14	5.60E-14
ZIC1	2.4671132	2.081382	7.29E-13	1.72E-12
DNAAF3	2.4667697	1.957289	1.68E-39	1.21E-38
HMGB3	2.4652668	6.349948	1.93E-76	3.44E-75
MLC1	2.4629637	1.63144	1.56E-14	4.00E-14
SLIT1	2.4621172	2.567784	3.19E-15	8.47E-15
KIF18A	2.4596491	3.149571	7.26E-78	1.33E-76
CCNE2	2.4584123	3.139931	4.18E-68	6.36E-67
AGR2	2.4550445	9.215433	1.13E-22	4.17E-22
GPR158	2.452801	1.471159	1.05E-20	3.55E-20
JPH3	2.452114	0.698943	2.31E-26	1.01E-25
CTHRC1	2.4472071	6.557418	2.82E-82	5.52E-81
CACNG4	2.4456134	6.56745	5.56E-29	2.71E-28
C1orf158	2.4455592	-2.3047	1.49E-10	3.12E-10
SLC4A10	2.4453672	1.16427	6.69E-13	1.58E-12
FGFR3	2.4452561	5.016323	2.44E-31	1.31E-30
COL9A3	2.4446429	4.032096	3.70E-18	1.13E-17

Gene names	logFC	log2CPM	P.Value	adj.P.Val
HIST1H4J	2.4404131	-1.93166	7.82E-36	4.94E-35
SPERT	2.4393615	-2.14127	3.06E-11	6.62E-11
KRTAP3_1	2.4393017	-3.24976	1.53E-11	3.37E-11
REG4	2.4359654	-2.37274	3.84E-16	1.07E-15
FAM72A	2.4353875	0.566868	2.46E-63	3.37E-62
NEUROD2	2.433289	-0.92914	2.63E-10	5.43E-10
BMP8A	2.4320092	0.417283	5.33E-75	9.24E-74
KRT81	2.4317547	6.386649	1.64E-11	3.59E-11
GDF15	2.4259094	4.47005	4.94E-29	2.41E-28
FBXO43	2.4258377	0.30893	7.13E-69	1.11E-67
NTS	2.4211622	4.800099	2.18E-05	3.37E-05
METRN	2.4205771	5.815244	6.87E-48	6.29E-47
PRR15	2.4198246	5.258549	6.22E-36	3.94E-35
NLRP7	2.4158762	-0.70514	4.82E-16	1.33E-15
DLX6	2.4157491	-0.4384	3.13E-12	7.11E-12
RUFY4	2.4124186	0.419306	4.57E-28	2.14E-27
HIST1H2BG	2.4107112	2.720266	3.73E-30	1.90E-29
SLC17A8	2.4092592	-2.50566	2.63E-10	5.43E-10
B4GALNT2	2.4066813	2.423694	2.22E-12	5.08E-12
CENPU	2.4065678	4.664997	9.70E-92	2.20E-90
LRRC31	2.4063943	2.648082	5.28E-13	1.25E-12
KCNJ10	2.4059878	0.353	5.41E-37	3.55E-36
GPRC5A	2.4044978	8.430332	8.93E-54	9.54E-53
FCAMR	2.4035866	-1.11293	5.07E-11	1.08E-10
STMND1	2.4024417	3.447931	7.03E-22	2.52E-21
LY6H	2.3993579	-0.14255	3.69E-32	2.04E-31
PYCR1	2.3959615	6.696488	3.14E-105	8.92E-104
PSCA	2.394791	3.652549	1.65E-15	4.45E-15

Gene names	logFC	log2CPM	P.Value	adj.P.Val
OASL	2.3935647	4.371497	4.27E-39	3.01E-38
SRMS	2.392246	2.496486	4.84E-46	4.22E-45
AGR3	2.3890102	6.811216	1.00E-18	3.11E-18
CACNA1H	2.3882036	5.3691	1.48E-24	5.95E-24
KCNG1	2.3836842	2.539359	1.08E-15	2.92E-15
PIF1	2.3819621	1.735507	7.03E-51	6.99E-50
PAH	2.379457	1.134493	8.10E-15	2.10E-14
PLA2G4D	2.3787055	-1.31326	3.31E-24	1.31E-23
TACC3	2.3777661	5.581312	1.04E-99	2.69E-98
KRT6A	2.3774219	5.389276	2.21E-08	4.10E-08
RCOR2	2.3728585	2.690575	1.76E-30	9.14E-30
PIH1D3	2.3716962	-3.11511	2.47E-13	5.96E-13
MFAP2	2.370405	5.449636	1.27E-68	1.97E-67
SIX2	2.3665322	3.224101	9.30E-29	4.48E-28
FAM72B	2.3662916	0.376049	2.46E-51	2.47E-50
C12orf56	2.3656061	-1.18439	2.67E-12	6.10E-12
ZG16	2.3644632	-2.36939	4.11E-10	8.41E-10
SERPINA6	2.3616785	5.556801	7.03E-07	1.20E-06
ATP2B3	2.3605703	-2.03248	6.78E-12	1.52E-11
KRT31	2.3600309	0.072523	1.51E-10	3.15E-10
KCNH2	2.3581956	2.502425	8.51E-23	3.17E-22
ZAN	2.3572337	-1.40941	2.37E-19	7.57E-19
BGN	2.3564899	9.565368	1.41E-82	2.79E-81
RGS8	2.3557986	-1.2359	6.65E-28	3.09E-27
GPR19	2.3556941	0.840471	7.34E-47	6.52E-46
PPM1E	2.3556578	1.714609	2.95E-25	1.22E-24
DOK7	2.3518843	3.326416	2.13E-23	8.11E-23
TUBB4A	2.3502939	0.918222	1.78E-16	5.02E-16
RNASE10	2.3499083	-2.61325	1.40E-23	5.39E-23

Gene names	logFC	log2CPM	P.Value	adj.P.Val
SQLE	2.3497883	6.725301	1.23E-68	1.90E-67
CYP21A2	2.3496014	1.634852	1.62E-28	7.75E-28
CABP7	2.3494847	-0.36553	2.41E-26	1.05E-25
UNC5A	2.3460252	3.11908	1.53E-23	5.88E-23
FCRL4	2.345004	-2.3469	4.20E-20	1.39E-19
TGM3	2.344593	0.437969	1.76E-17	5.21E-17
IGSF23	2.3436224	-2.08355	9.10E-14	2.24E-13
EZH2	2.3433542	4.732953	2.27E-97	5.60E-96
TBX20	2.3424474	-1.55012	1.75E-09	3.46E-09
DNASE1L2	2.3401425	0.661434	6.83E-47	6.08E-46
SPP1	2.3371331	8.063879	2.37E-34	1.43E-33
UGT1A6	2.3370319	-1.04221	8.45E-05	0.00012597
SERPINA12	2.3349959	-0.12418	2.08E-17	6.14E-17
RHPN1	2.3343881	5.591496	2.42E-67	3.60E-66
TAGLN3	2.3319648	-1.42425	1.52E-09	3.02E-09
GHRH	2.3296929	-0.71046	1.49E-08	2.79E-08
CREB3L1	2.3279503	6.262114	1.46E-49	1.40E-48
SMC1B	2.325602	1.114336	3.56E-23	1.35E-22
SBK1	2.325382	5.380455	2.24E-74	3.84E-73
HIST1H1C	2.3222459	6.507779	1.24E-36	8.06E-36
HIST2H3D	2.3217393	-2.51176	2.70E-21	9.44E-21
NCCRP1	2.3199398	4.046806	2.49E-14	6.31E-14
CYSRT1	2.3191892	1.277491	9.13E-49	8.56E-48
TRIM67	2.3183493	0.517903	2.40E-14	6.10E-14
WFDC6	2.3183443	-0.91759	9.55E-10	1.91E-09
ADAMTS19	2.3180569	1.223789	4.86E-16	1.34E-15
RIMS2	2.3171732	2.166102	3.01E-15	8.00E-15
CDHR4	2.3169782	-0.37186	4.14E-13	9.88E-13

Gene names	logFC	log2CPM	P.Value	adj.P.Val
MMP3	2.3163179	3.888926	1.61E-29	8.05E-29
ORC1	2.3152491	2.781974	3.21E-61	4.16E-60
SRD5A2	2.3144192	-0.28584	6.42E-23	2.41E-22
DIAPH3	2.3143638	3.524835	2.25E-62	3.01E-61
RAD51AP1	2.3134303	3.657488	6.05E-71	9.81E-70
SLC1A1	2.3109856	5.601842	1.12E-20	3.80E-20
ADAM8	2.3075709	4.530427	1.28E-68	1.98E-67
LACRT	2.3069553	3.814671	0.0006404	0.00089896
COL11A2	2.2984038	3.123429	6.40E-14	1.59E-13
ILDR2	2.2980758	0.916913	2.66E-49	2.53E-48
C1orf194	2.2964127	-0.29813	3.57E-26	1.54E-25
C4orf50	2.2947904	-2.3529	8.43E-22	3.00E-21
KRT6C	2.2945415	1.547922	2.09E-07	3.66E-07
WNT7A	2.2929581	-2.10058	1.61E-11	3.52E-11
HIST1H2AG	2.2922208	2.17715	9.14E-28	4.23E-27
SLC16A3	2.2915577	5.547393	3.28E-56	3.73E-55
SAPCD2	2.2909267	4.477686	1.47E-60	1.87E-59
C16orf92	2.2905937	-3.41131	7.74E-19	2.41E-18
GPR111	2.2898785	-2.67099	3.86E-14	9.71E-14
SLC5A12	2.2867152	-0.84289	8.99E-11	1.90E-10
FOXH1	2.2851457	-1.47133	4.36E-39	3.07E-38
GSTA2	2.2822589	-1.27086	8.37E-10	1.68E-09
PTCHD2	2.2816814	0.913974	2.21E-24	8.78E-24
COL1A1	2.2784858	12.74124	1.36E-49	1.31E-48
NUAK2	2.2780677	3.506916	1.17E-103	3.26E-102
SDS	2.2780129	3.543078	7.13E-65	1.01E-63
SIM2	2.2753435	3.26561	2.40E-30	1.24E-29
CTSE	2.2752438	-0.28269	7.47E-09	1.43E-08
GJA3	2.2737284	-1.37735	6.47E-28	3.01E-27

Gene names	logFC	log2CPM	P.Value	adj.P.Val
HS3ST6	2.273545	-1.12282	1.33E-09	2.65E-09
EN1	2.2715487	3.930941	4.40E-16	1.22E-15
SLC15A1	2.2710527	1.592432	1.60E-10	3.34E-10
LYPD1	2.2660155	0.709126	1.82E-39	1.30E-38
HPN	2.2618779	5.502595	6.63E-36	4.19E-35
ARHGAP11A	2.2591426	4.464626	2.63E-75	4.59E-74
NIPAL4	2.2588471	0.268354	2.03E-19	6.48E-19
CHST6	2.2587067	1.854008	9.74E-33	5.50E-32
CD80	2.257531	0.468534	9.37E-53	9.71E-52
C2CD4A	2.2564428	2.48961	5.08E-13	1.21E-12
ANKRD30B	2.2553552	3.600574	1.13E-13	2.77E-13
RLBP1	2.2545927	-1.37916	5.56E-08	1.01E-07
UGT2B10	2.2518476	-2.18202	2.57E-06	4.22E-06
AUNIP	2.2518243	2.128777	8.15E-68	1.23E-66
OLR1	2.2499931	4.369259	2.48E-60	3.14E-59
C10orf82	2.2495625	2.462022	5.55E-13	1.31E-12
SLC7A11	2.2479498	3.365908	3.43E-33	1.98E-32
ENSG00000183248	2.2477252	5.240897	3.51E-55	3.89E-54
HN1	2.2475789	6.893883	2.60E-78	4.78E-77
HDC	2.2435064	3.591618	4.27E-12	9.64E-12
PGLYRP2	2.2427611	3.00157	5.66E-14	1.41E-13
CENPI	2.2423544	2.670276	1.18E-72	1.96E-71
C1QTNF6	2.2374536	5.522145	3.60E-91	8.11E-90
BRIP1	2.2356739	3.61465	2.05E-60	2.59E-59
GPR37L1	2.2333308	0.692677	6.13E-25	2.51E-24
EPN3	2.228409	5.591827	2.03E-50	1.99E-49
MT1H	2.2279265	-1.51793	5.24E-16	1.45E-15
GRIN2D	2.2274442	2.202413	8.12E-42	6.27E-41

Gene names	logFC	log2CPM	P.Value	adj.P.Val
PARD6B	2.2239124	5.777738	6.73E-34	3.98E-33
FOXA3	2.2212017	0.250881	1.63E-13	3.96E-13
TMEM171	2.2199281	-0.79334	3.59E-22	1.30E-21
PADI1	2.2190372	-2.01559	9.99E-14	2.46E-13
MIOX	2.2174617	-2.19778	7.76E-28	3.60E-27
CCDC151	2.2150137	0.974166	1.67E-30	8.69E-30
ADM2	2.2145513	3.959218	6.19E-59	7.56E-58
TMEM132A	2.2144557	6.717845	7.24E-60	9.05E-59
CDC25A	2.2108683	3.106943	7.96E-56	8.99E-55
AARD	2.2100725	5.922148	7.62E-21	2.61E-20
MAD2L1	2.2094739	4.646231	1.95E-78	3.59E-77
CAPS	2.2067987	5.598	5.95E-42	4.61E-41
CNGA3	2.2020644	0.167278	1.02E-13	2.49E-13
SORCS3	2.1996832	-1.14837	3.09E-10	6.35E-10
KPNA2	2.1956548	7.299823	3.82E-78	7.02E-77
KIRREL2	2.1953656	-0.50952	8.77E-14	2.16E-13
SPOCD1	2.193826	2.45933	7.05E-33	4.00E-32
DKK1	2.1926119	2.726597	1.50E-13	3.66E-13
TNIP3	2.1909103	-0.2468	1.46E-21	5.17E-21
TRIM43	2.1908433	-2.71221	3.51E-05	5.36E-05
WDR72	2.1900177	2.491307	6.66E-10	1.35E-09
TEKT1	2.1872172	-0.69757	2.80E-12	6.37E-12
GINS2	2.185388	4.40569	1.11E-63	1.54E-62
MGAT5B	2.1852905	1.671647	1.07E-20	3.61E-20
CFAP99	2.1851727	-1.74569	4.92E-26	2.11E-25
LTB	2.184713	3.408122	4.14E-23	1.56E-22
TAS1R3	2.1843489	1.360849	5.57E-32	3.06E-31
TACR3	2.1797996	-2.15065	3.59E-12	8.14E-12
STARD10	2.1797373	7.93601	3.46E-44	2.86E-43

Gene names	logFC	log2CPM	P.Value	adj.P.Val
CRABP2	2.179713	9.093513	2.09E-38	1.44E-37
OLIG1	2.1793001	-1.07318	2.58E-09	5.06E-09
TMEM132E	2.1778928	0.719641	3.48E-18	1.06E-17
MYBL1	2.1774131	4.269191	2.94E-36	1.89E-35
NKAIN4	2.1761834	-1.22893	7.01E-20	2.29E-19
ZMAT4	2.1751711	1.142541	5.15E-14	1.29E-13
KCNQ2	2.1737402	-0.80778	9.64E-09	1.83E-08
NETO1	2.1730203	0.167867	8.96E-19	2.79E-18
MYT1L	2.1724877	-1.86481	5.54E-09	1.06E-08
DYDC2	2.1649908	0.215413	5.33E-23	2.00E-22
FOXP3	2.1640395	2.5944	3.18E-57	3.71E-56
MAP3K19	2.1637153	-0.92908	5.43E-17	1.57E-16
ALG1L	2.1595476	1.150641	3.51E-20	1.16E-19
POSTN	2.1582933	10.19171	2.81E-48	2.60E-47
RNFT2	2.1581587	2.823941	2.71E-69	4.25E-68
WFDC13	2.1570904	-3.01609	4.21E-11	9.03E-11
ENSG00000226321	2.1549839	-0.98624	3.56E-12	8.07E-12
INHA	2.1543364	1.033119	1.15E-22	4.27E-22
POC1A	2.1474309	3.674011	9.80E-113	3.20E-111
TONSL	2.1463246	4.641009	6.04E-72	9.99E-71
UTS2	2.1457882	-1.71718	1.46E-18	4.50E-18
DLX2	2.144047	1.74386	1.54E-11	3.39E-11
RGS7	2.1418886	-0.56168	4.42E-14	1.11E-13
ANKRD22	2.1408731	2.849818	3.98E-44	3.29E-43
LRRC46	2.1401791	2.578002	2.44E-42	1.91E-41
SHISA9	2.1400875	3.147505	1.17E-18	3.63E-18
SUSD3	2.1399832	5.85265	1.15E-25	4.85E-25
KIFC2	2.139261	4.56336	2.28E-54	2.48E-53

Gene names	logFC	log2CPM	P.Value	adj.P.Val
ZFR2	2.1380871	-0.41051	2.09E-11	4.55E-11
C3orf67	2.1362647	2.293049	2.22E-53	2.35E-52
LRRIQ4	2.1362248	-1.45708	1.22E-18	3.78E-18
SPTSSB	2.1349309	4.824017	1.49E-17	4.42E-17
CAMKV	2.1345176	-1.27528	4.43E-09	8.56E-09
SYN1	2.1343835	1.70554	3.67E-24	1.45E-23
HIST1H2BK	2.13207	5.085358	6.49E-56	7.35E-55
KRT77	2.1320593	0.066181	2.80E-05	4.31E-05
STIL	2.1302203	4.176753	6.29E-72	1.04E-70
SYT14	2.1262299	-0.16498	3.26E-12	7.40E-12
CALY	2.1255076	-1.73775	6.77E-14	1.68E-13
CHST1	2.1254011	4.615207	1.98E-24	7.91E-24
LRRC73	2.1229081	1.615281	4.05E-41	3.06E-40
ECEL1	2.1225244	3.25504	2.69E-10	5.55E-10
CCDC108	2.1219361	0.037203	1.39E-16	3.94E-16
CSMD2	2.1206987	1.944455	7.98E-39	5.57E-38
LRG1	2.1187406	5.609928	5.71E-21	1.97E-20
IL12B	2.1161207	-1.4891	3.02E-22	1.10E-21
BFSP2	2.1159571	-0.9797	1.00E-19	3.24E-19
DNAJC12	2.1157691	5.516481	1.22E-23	4.72E-23
TLX2	2.1142498	-2.35133	7.88E-20	2.57E-19
C6orf15	2.1126242	2.130451	1.50E-07	2.66E-07
SSTR2	2.109846	4.250098	1.63E-22	5.99E-22
C1orf189	2.1096767	-1.68698	4.62E-24	1.81E-23
POTEI	2.1068124	-0.08119	7.16E-15	1.86E-14
CNTN5	2.1031749	-0.59389	1.50E-08	2.82E-08
TICRR	2.1016534	3.33162	5.19E-49	4.91E-48
RTKN2	2.1014746	3.35837	2.76E-63	3.77E-62
MAST1	2.0998503	1.078043	1.24E-25	5.23E-25

Gene names	logFC	log2CPM	P.Value	adj.P.Val
ASCL2	2.0995371	2.2564	1.74E-24	6.97E-24
SCX	2.0990417	0.386119	3.64E-30	1.86E-29
DMBT1	2.0922567	2.598038	1.31E-07	2.32E-07
BPIFB2	2.0904365	4.448361	1.87E-08	3.48E-08
MEX3A	2.0884536	5.680533	1.38E-35	8.63E-35
IDO1	2.0873955	4.260022	9.99E-17	2.85E-16
SPRR2E	2.0852272	-0.71276	0.0021971	0.00296988
MIEN1	2.0832026	6.544828	3.00E-33	1.73E-32
ARNT2	2.0830082	6.134932	4.24E-34	2.53E-33
PROZ	2.0821737	-1.151	4.36E-23	1.65E-22
FGD3	2.0801112	5.921195	2.40E-34	1.45E-33
HIST1H3A	2.0799349	-2.19545	4.97E-14	1.24E-13
DLX4	2.079268	0.978965	1.70E-29	8.46E-29
HIST3H2A	2.0792659	4.159974	7.27E-30	3.67E-29
HCRTR2	2.0778979	-2.11542	1.19E-06	1.99E-06
NPTX2	2.0774284	1.955237	6.36E-22	2.28E-21
KPNA7	2.0760637	-0.95845	7.53E-23	2.81E-22
SPACA4	2.0760637	-1.51487	2.21E-24	8.80E-24
KLK14	2.0758206	3.224758	6.61E-09	1.26E-08
C9orf172	2.0747736	2.476412	6.12E-59	7.48E-58
LPO	2.0743005	-0.27309	1.47E-12	3.40E-12
TIGD3	2.0725335	0.118474	3.87E-56	4.40E-55
C9orf116	2.070663	3.206476	1.64E-52	1.69E-51
GTSF1	2.0706568	0.478743	1.36E-15	3.69E-15
IL9R	2.0695183	-1.97137	2.26E-36	1.45E-35
TIGIT	2.0688116	2.82865	8.00E-28	3.71E-27
HPDL	2.0648968	1.423577	3.08E-19	9.81E-19
RAET1L	2.0647536	-0.55706	2.05E-10	4.26E-10

Gene names	logFC	log2CPM	P.Value	adj.P.Val
SYT9	2.0628074	3.30714	3.34E-14	8.42E-14
BCAS4	2.0619977	5.044275	2.76E-55	3.08E-54
HIST1H4I	2.0618409	4.023453	1.77E-54	1.93E-53
FBXL16	2.0596649	5.105592	2.75E-33	1.59E-32
TEX14	2.0578703	2.271877	2.49E-19	7.95E-19
CPA6	2.056962	1.799027	5.65E-10	1.15E-09
PTK6	2.0561551	4.448785	2.29E-47	2.07E-46
TYMS	2.0553738	4.798393	1.01E-62	1.37E-61
LCN12	2.0543711	1.53403	1.23E-24	4.98E-24
GSG2	2.0536873	1.570983	1.26E-57	1.49E-56
HIST2H2BE	2.0522278	6.350645	1.16E-42	9.17E-42
PHGR1	2.0505878	1.168012	1.62E-08	3.03E-08
FUT7	2.0473416	-0.4162	2.31E-29	1.14E-28
LEF1	2.0472908	4.75993	1.64E-48	1.53E-47
TPRN	2.0472355	5.362313	1.80E-72	2.99E-71
MROH6	2.0446676	3.746649	9.27E-38	6.24E-37
NUPR1L	2.0445435	2.011404	1.52E-21	5.36E-21
RHCG	2.0441176	2.388173	1.26E-08	2.38E-08
FIBCD1	2.043614	1.787316	4.31E-13	1.03E-12
NTSR1	2.0428518	-1.02645	1.04E-12	2.42E-12
SMKR1	2.0362318	1.832019	3.65E-42	2.84E-41
MTL5	2.0352503	5.220518	2.95E-52	3.03E-51
C4orf48	2.034904	1.823513	4.68E-32	2.58E-31
VMO1	2.0329706	1.95176	1.27E-19	4.10E-19
CCDC175	2.0313269	-2.82799	1.51E-09	3.01E-09
C19orf45	2.030649	0.338131	6.30E-40	4.57E-39
CEACAM7	2.0293757	0.154313	2.32E-13	5.60E-13
UNC80	2.0258922	-0.02884	3.37E-10	6.93E-10
ZG16B	2.0256382	6.458011	2.20E-29	1.09E-28

Gene names	logFC	log2CPM	P.Value	adj.P.Val
FAM71F1	2.0252814	-2.18997	7.13E-20	2.33E-19
FRMPD3	2.0249221	2.234536	1.02E-25	4.33E-25
RNF224	2.0249039	-0.78951	3.73E-33	2.14E-32
BAI2	2.0245464	5.134937	3.28E-31	1.76E-30
ETV7	2.0240586	2.806036	4.24E-38	2.89E-37
TRIM59	2.0211627	3.759016	7.26E-108	2.17E-106
CIT	2.0190187	4.571806	5.00E-82	9.76E-81
LAG3	2.0176407	2.578314	6.14E-28	2.86E-27
PRB3	2.0173826	-1.79988	1.93E-17	5.69E-17
CLIC6	2.0169881	6.571293	5.70E-12	1.28E-11
CHP2	2.0168387	-1.29699	4.42E-06	7.13E-06
VTN	2.0164911	0.984653	6.36E-15	1.66E-14
BLM	2.0146604	3.417839	1.19E-61	1.56E-60
SLC17A9	2.0134087	3.445976	9.62E-31	5.05E-30
LAGE3	2.0129333	4.90582	6.68E-70	1.06E-68
NPNT	2.0129313	7.587412	1.69E-32	9.49E-32
HOXB1	2.011622	-2.0005	1.95E-12	4.47E-12
AC234582.1	2.0112148	-1.63277	7.02E-23	2.63E-22
CCDC83	2.0104847	-2.14485	8.48E-16	2.32E-15
SPPL2C	2.0089182	-3.38122	1.93E-10	4.01E-10
KRT86	2.0079763	2.896279	2.78E-15	7.41E-15
IGSF9	2.0045476	5.776989	5.87E-60	7.36E-59
FANCI	2.0029756	5.58956	2.56E-86	5.40E-85
S100A2	2.0020204	4.690218	2.62E-11	5.67E-11
UNC5B	2.0016518	6.295604	1.85E-79	3.46E-78
PRRT3	2.0004508	4.176328	8.13E-50	7.82E-49
KRT16	2.0002187	6.017549	3.85E-09	7.46E-09
KRT79	1.9993968	1.965687	1.02E-06	1.72E-06

Gene names	logFC	log2CPM	P.Value	adj.P.Val
ATAD2	1.9993823	6.469949	1.25E-61	1.64E-60
ANKRD34B	1.9990662	-1.45236	6.21E-11	1.32E-10
FOXD4L1	1.9970299	-2.42023	1.98E-34	1.20E-33
NKX6_3	1.9948984	-2.46546	8.51E-07	1.44E-06
GINS4	1.9891204	3.350374	8.09E-45	6.81E-44
LIPM	1.987065	-0.88923	1.33E-31	7.22E-31
CLLU1OS	1.9851241	-2.31626	5.00E-10	1.02E-09
ECE2	1.9847134	3.76124	4.71E-60	5.94E-59
AICDA	1.9833204	-1.17632	4.26E-08	7.79E-08
NDP	1.983208	3.195936	2.04E-11	4.43E-11
PXDNL	1.9826002	3.287653	3.29E-13	7.88E-13
SMCO2	1.9745507	-1.50709	1.03E-37	6.95E-37
IRF7	1.9743863	5.452173	1.11E-60	1.41E-59
TPH1	1.9734998	2.435354	9.11E-12	2.02E-11
PLA2G3	1.9710346	0.567484	1.20E-09	2.40E-09
HOXB5	1.9707839	2.643468	4.66E-15	1.22E-14
SLC39A4	1.9707174	4.629951	1.13E-41	8.65E-41
ENSG00000180044	1.9685216	2.163065	4.57E-67	6.78E-66
NME1	1.9684347	6.17956	1.64E-62	2.20E-61
B4GALNT4	1.9663667	5.00618	5.31E-47	4.76E-46
HPX	1.9638143	4.614341	7.38E-17	2.12E-16
SLC50A1	1.9632972	6.641237	6.58E-105	1.85E-103
GSDMC	1.9624649	2.030842	3.88E-16	1.08E-15
HES2	1.9604294	2.656372	2.63E-18	8.03E-18
SCG3	1.9593196	1.535623	2.38E-07	4.15E-07
KCNK1	1.9578818	4.929646	1.30E-26	5.71E-26
CWH43	1.9575651	0.604241	4.01E-09	7.77E-09
AWAT1	1.957545	-2.69611	4.59E-08	8.38E-08
GRIN2C	1.9574862	1.735071	1.62E-17	4.80E-17

Gene names	logFC	log2CPM	P.Value	adj.P.Val
GPR141	1.9567158	0.505273	2.22E-32	1.24E-31
KIAA1257	1.956707	1.533552	4.48E-37	2.95E-36
KRT19	1.9560223	10.65187	7.41E-43	5.89E-42
CCDC167	1.9555745	4.105603	2.09E-85	4.32E-84
SLC25A48	1.9532962	2.057141	4.78E-12	1.08E-11
PRR19	1.9517491	1.69914	2.94E-85	6.06E-84
HS3ST3A1	1.9515667	0.312382	2.27E-40	1.67E-39
EFNA3	1.9513485	4.091215	2.45E-36	1.57E-35
PRR7	1.9502464	1.933203	1.71E-40	1.27E-39
KIR2DL4	1.9484927	-1.83979	8.15E-15	2.11E-14
ERBB2	1.9484529	9.750844	4.02E-24	1.58E-23
C20orf144	1.9483493	-0.96497	1.47E-45	1.27E-44
GPHA2	1.9474439	-3.20706	7.83E-11	1.66E-10
FAM132B	1.9471991	-0.42916	4.82E-25	1.98E-24
SDC1	1.9470476	8.63007	9.61E-48	8.78E-47
RACGAP1	1.9465836	5.592606	1.91E-85	3.96E-84
H2AFX	1.9457445	5.362164	1.32E-71	2.17E-70
NUP210	1.9453496	7.058639	1.17E-90	2.61E-89
WDR62	1.9442265	3.786114	2.38E-50	2.33E-49
TMEM238	1.942909	2.38795	5.80E-40	4.21E-39
COL5A1	1.9425989	8.96447	6.09E-47	5.43E-46
SYCE3	1.9417762	-0.02531	8.30E-29	4.01E-28
FAM83E	1.9388525	3.692113	1.22E-17	3.63E-17
FANCA	1.9385136	3.597714	4.41E-53	4.62E-52
RBP2	1.9378264	-0.9698	8.84E-17	2.52E-16
SDR16C5	1.9377113	4.209861	1.51E-14	3.89E-14
DNAH11	1.9370272	1.485102	2.17E-14	5.52E-14
LGSN	1.9369314	-2.23347	1.61E-08	3.01E-08

Gene names	logFC	log2CPM	P.Value	adj.P.Val
CHST8	1.9363089	3.561539	3.34E-13	8.01E-13
DBNDD1	1.9352404	5.492038	3.36E-55	3.73E-54
CENPK	1.9350953	2.520452	1.09E-70	1.76E-69
SHROOM1	1.934778	5.861569	6.27E-19	1.96E-18
AIM2	1.9340913	1.263084	4.54E-22	1.64E-21
BRDT	1.9314124	-0.86146	2.43E-10	5.03E-10
LY6D	1.9311143	3.431557	2.81E-06	4.60E-06
LAMP3	1.9281571	3.846499	1.90E-21	6.69E-21
TPD52	1.927874	8.116488	3.20E-63	4.37E-62
KLHL35	1.9272455	2.987395	4.40E-36	2.80E-35
UNC5D	1.926815	-0.63597	6.25E-06	1.00E-05
C7orf57	1.9263917	-1.77996	1.12E-11	2.47E-11
TUBB8	1.922164	-0.73771	5.87E-34	3.48E-33
GPR84	1.9216596	1.013908	3.61E-39	2.55E-38
ECT2	1.9187173	5.86861	1.76E-81	3.41E-80
PYCARD	1.9170168	5.352292	4.01E-40	2.92E-39
XRCC2	1.9163586	3.022791	2.56E-60	3.23E-59
BRINP2	1.9157674	2.995406	2.02E-09	3.98E-09
SULF1	1.9150175	8.186754	3.51E-46	3.07E-45
C15orf48	1.9137006	4.846572	1.12E-26	4.93E-26
CORIN	1.9122645	2.742318	1.02E-28	4.91E-28
TTC16	1.9096498	0.083603	1.01E-36	6.57E-36
IFI27	1.9086764	8.178172	1.87E-22	6.86E-22
KIAA1524	1.9081286	3.562341	1.63E-58	1.96E-57
MDGA2	1.9077513	-1.49958	2.32E-07	4.06E-07
GPC2	1.9064161	1.477931	2.60E-24	1.03E-23
FER1L6	1.9057436	-1.30116	6.51E-14	1.62E-13
ENSG00000135747	1.9055439	-1.37397	3.13E-37	2.07E-36
FUT3	1.9036526	2.513429	1.26E-12	2.94E-12

Gene names	logFC	log2CPM	P.Value	adj.P.Val
SLC9A3R1	1.9033247	8.512798	1.95E-44	1.63E-43
VWA5B2	1.9022616	0.411883	4.16E-18	1.26E-17
OBP2B	1.9021217	3.041453	4.90E-08	8.93E-08
LBP	1.8979304	4.675633	6.11E-08	1.10E-07
PSRC1	1.8973762	3.542797	1.73E-67	2.58E-66
ODAM	1.8961198	0.783744	3.08E-06	5.02E-06
C3orf36	1.8959988	-1.00982	6.91E-47	6.15E-46
SLC12A5	1.8956035	0.339695	1.91E-24	7.65E-24
C9orf135	1.8952084	-1.99069	3.65E-06	5.94E-06
OBP2A	1.8948725	-0.0194	4.19E-13	9.98E-13
PPM1J	1.8926677	3.401921	9.10E-37	5.92E-36
CEACAM16	1.891235	-2.73026	1.25E-08	2.36E-08
CXCR5	1.8907439	-1.97965	3.16E-14	7.97E-14
SAMD14	1.8900852	1.133732	9.72E-68	1.46E-66
FOXD2	1.8897029	1.435482	1.23E-38	8.56E-38
HOXD13	1.8896378	-0.89472	1.07E-09	2.14E-09
SYT10	1.8894698	-2.23486	1.55E-06	2.58E-06
GLYATL1	1.8887558	2.986428	3.98E-16	1.10E-15
ICOS	1.8873148	1.163288	2.66E-22	9.71E-22
GTSF1L	1.8872295	-3.64879	3.00E-14	7.58E-14
SPATA17	1.8839703	2.650917	1.28E-48	1.20E-47
KLRG2	1.878723	2.720059	2.09E-19	6.68E-19
TSACC	1.8776222	-0.20524	3.22E-57	3.76E-56
ZNF703	1.8774635	7.710345	2.30E-28	1.09E-27
PKIB	1.8763137	5.719008	4.87E-27	2.19E-26
N4BP3	1.8756928	5.257332	4.52E-53	4.74E-52
P2RX2	1.875566	-0.32029	4.83E-09	9.31E-09
TNFSF4	1.8753722	2.975073	9.05E-53	9.40E-52

Gene names	logFC	log2CPM	P.Value	adj.P.Val
SLC34A3	1.8736222	-1.20804	1.13E-25	4.77E-25
CAPN13	1.8736202	5.255266	6.07E-24	2.37E-23
KRT8	1.8732683	10.3279	8.67E-47	7.69E-46
WNT7B	1.8727039	4.518199	6.43E-29	3.12E-28
ART3	1.8721771	2.876619	8.06E-07	1.37E-06
COMMD3_BMI1	1.8720891	-3.05971	1.12E-12	2.61E-12
FOXA1	1.870649	8.215274	7.30E-24	2.84E-23
GATA3	1.8700182	9.177097	2.16E-29	1.07E-28
LPPR3	1.869941	3.110018	9.26E-14	2.28E-13
OGDHL	1.8695274	0.521354	3.47E-11	7.48E-11
UBD	1.8681473	3.319034	4.01E-15	1.06E-14
CHTF18	1.8656271	4.472021	2.32E-56	2.65E-55
HIST1H2AE	1.8591574	2.355149	5.68E-20	1.86E-19
KCNC1	1.8576488	0.884393	3.81E-10	7.82E-10
NEFL	1.857525	-0.05639	4.10E-13	9.78E-13
PYCRL	1.8573403	5.083101	1.36E-66	2.01E-65
TMEM244	1.8571413	-2.56552	4.19E-22	1.52E-21
TMEM72	1.8564734	-2.57505	3.19E-09	6.22E-09
TTBK1	1.8563118	-0.37207	1.24E-26	5.45E-26
SOX12	1.8555818	5.843122	1.30E-88	2.83E-87
LBX2	1.8555756	0.525107	7.97E-50	7.67E-49
DCDC1	1.8545171	2.098831	5.38E-18	1.62E-17
ESPN	1.8544142	4.650032	4.05E-28	1.90E-27
TYMP	1.8524809	6.693955	5.02E-50	4.86E-49
CLIC3	1.8518733	2.487788	2.51E-22	9.15E-22
CCNI2	1.8512699	0.973276	3.89E-21	1.35E-20
C2orf50	1.8512592	0.834735	5.30E-18	1.60E-17
RIBC2	1.8508333	1.945098	4.32E-53	4.54E-52
ASPN	1.8504747	6.876272	1.78E-23	6.81E-23

Gene names	logFC	log2CPM	P.Value	adj.P.Val
FAM177B	1.8500639	0.647105	3.08E-14	7.77E-14
RSPH1	1.8498008	3.06757	7.60E-32	4.15E-31
UGT3A2	1.8496406	0.431079	1.79E-10	3.72E-10
DNAH8	1.8488429	-1.48761	3.99E-18	1.21E-17
PCDHA1	1.8477142	-0.12094	1.29E-14	3.32E-14
LDLRAD1	1.8469205	0.246567	4.10E-14	1.03E-13
SRRM4	1.8455303	-2.06682	7.55E-07	1.28E-06
KNDC1	1.8433758	2.144999	1.03E-10	2.17E-10
CBS	1.841912	-0.68326	5.75E-21	1.98E-20
THPO	1.8403227	1.48187	2.72E-19	8.67E-19
SLC52A2	1.8389644	6.290025	1.90E-69	3.00E-68
PLA2G10	1.838818	-0.5982	2.07E-24	8.26E-24
MCM4	1.8376207	7.203908	6.12E-64	8.51E-63
RNASEH2A	1.8365096	5.070498	1.72E-90	3.82E-89
MCM2	1.8353478	6.092914	1.60E-65	2.31E-64
PCP2	1.8347872	1.909037	9.89E-20	3.21E-19
TSTA3	1.8340735	6.886493	7.62E-79	1.42E-77
FSTL4	1.8338943	2.324984	7.06E-22	2.53E-21
FAM92B	1.8337687	-1.3333	1.15E-13	2.82E-13
REM2	1.8312235	0.82075	3.26E-37	2.16E-36
LTA	1.8304611	0.113772	7.44E-24	2.89E-23
CCDC74A	1.8298448	5.377847	1.65E-19	5.29E-19
PGBD5	1.8297268	3.328493	6.13E-17	1.76E-16
C5orf46	1.8282349	2.095433	1.73E-17	5.10E-17
ZBP1	1.8270377	2.19064	3.20E-22	1.16E-21
PDCD1	1.8258782	1.077821	5.00E-22	1.80E-21
SLC26A9	1.8246971	0.416788	5.84E-08	1.06E-07
FAM173A	1.8241884	3.864773	2.39E-44	1.98E-43

Gene names	logFC	log2CPM	P.Value	adj.P.Val
GUCA1A	1.8239739	-2.85568	2.96E-17	8.66E-17
ZIC4	1.8230884	0.226879	8.09E-10	1.63E-09
SOWAHA	1.8208124	3.226196	2.04E-13	4.95E-13
TMSB15A	1.8199432	1.580556	1.78E-15	4.79E-15
FAM83H	1.8197952	7.265806	1.99E-77	3.60E-76
PANX2	1.8193612	2.572883	1.92E-25	8.02E-25
CYP4F2	1.8181743	-1.60994	9.65E-07	1.63E-06
NEFH	1.8177785	3.096443	1.13E-16	3.21E-16
ADGB	1.8175733	-2.33206	2.93E-11	6.34E-11
STMN1	1.8165468	7.744233	9.84E-55	1.08E-53
CCL17	1.8165008	0.046679	1.49E-19	4.79E-19
PMAIP1	1.8163481	4.19272	2.20E-36	1.42E-35
TH	1.8163067	-0.41438	5.43E-09	1.04E-08
IFNG	1.8158536	-1.13127	4.91E-13	1.17E-12
TCL1A	1.8126176	0.856056	4.27E-09	8.27E-09
RLN2	1.8118988	1.304255	8.14E-15	2.11E-14
FGFR4	1.8102438	4.315467	3.50E-16	9.73E-16
ROPN1L	1.810041	0.350626	1.04E-18	3.22E-18
FBXL6	1.8099584	4.802025	2.06E-66	3.03E-65
ULBP2	1.8084697	0.505361	1.35E-22	4.97E-22
PTPRN	1.8077765	1.251063	1.24E-12	2.89E-12
RGS4	1.8068748	3.696935	2.36E-31	1.27E-30
B4GALNT3	1.8043467	5.008056	7.85E-29	3.80E-28
C10orf67	1.8036591	-2.34134	1.68E-10	3.50E-10
GALR2	1.8005997	-3.04336	3.96E-16	1.10E-15
COL20A1	1.7989226	-2.73629	1.98E-10	4.11E-10
SNAP25	1.7983481	2.42235	3.19E-10	6.56E-10
LLGL2	1.7966874	6.674718	1.97E-73	3.32E-72
IDO2	1.7962551	-1.96413	1.02E-13	2.50E-13

Gene names	logFC	log2CPM	P.Value	adj.P.Val
USH1G	1.795298	-1.40769	2.07E-14	5.29E-14
ARHGEF39	1.7951334	2.745563	5.09E-60	6.41E-59
GRB14	1.7935075	3.718729	6.49E-11	1.38E-10
DAZL	1.7930729	-2.54489	4.11E-09	7.96E-09
CEND1	1.7926494	-1.76386	1.37E-25	5.77E-25
CDC7	1.7917027	3.706959	6.51E-53	6.79E-52
TEKT5	1.7890411	-0.26162	9.79E-28	4.52E-27
C8orf86	1.7884745	-1.15974	1.39E-11	3.05E-11
PLK4	1.7874901	3.556595	1.18E-65	1.72E-64
SLC38A5	1.7870001	3.189778	7.48E-33	4.24E-32
C16orf93	1.7856183	0.841961	8.12E-41	6.07E-40
ANXA9	1.7855163	5.389233	1.22E-33	7.12E-33
MX1	1.7838329	7.716232	3.18E-24	1.26E-23
CHRDL2	1.7829261	2.193385	8.34E-10	1.68E-09
CCNF	1.7813821	4.985922	1.80E-93	4.18E-92
MYRFL	1.7811343	0.486833	1.06E-20	3.60E-20
C1orf233	1.7801546	4.231582	1.95E-46	1.72E-45
ECM1	1.7800693	7.119053	4.76E-19	1.50E-18
KMO	1.779452	3.267413	1.33E-21	4.72E-21
COL5A2	1.7784241	9.047435	1.04E-39	7.48E-39
KIAA1644	1.778117	2.2642	1.89E-21	6.63E-21
HELLS	1.777673	4.21662	1.62E-62	2.18E-61
TMEFF1	1.7766185	-2.92651	3.21E-13	7.70E-13
C16orf71	1.7760188	1.967386	1.51E-41	1.16E-40
SNCB	1.7755042	-2.79846	3.82E-10	7.82E-10
CA12	1.7749118	8.816586	1.13E-21	4.02E-21
ZNF730	1.7732564	0.368714	5.53E-22	1.99E-21
IER5L	1.7731962	4.227385	4.03E-52	4.13E-51

Gene names	logFC	log2CPM	P.Value	adj.P.Val
GPR68	1.7725735	3.699083	1.05E-59	1.30E-58
ALX3	1.7698175	-2.30208	2.23E-08	4.13E-08
SORD	1.7696278	5.959126	2.93E-39	2.07E-38
UGT2B17	1.7695668	1.357009	5.13E-06	8.26E-06
TPRXL	1.7673134	0.459031	6.48E-13	1.53E-12
PCSK9	1.7652205	0.691667	4.16E-14	1.04E-13
PODXL2	1.7644797	5.678058	2.33E-37	1.55E-36
ASPHD1	1.763778	2.430067	5.05E-19	1.59E-18
FOXE3	1.7620995	-3.15707	3.39E-11	7.30E-11
TJP3	1.7616756	5.882535	3.91E-49	3.71E-48
NAT14	1.7614035	4.418768	1.60E-54	1.75E-53
C21orf58	1.7597006	3.314893	3.25E-64	4.56E-63
SLC7A13	1.7596152	-0.62287	2.37E-14	6.01E-14
KLF17	1.7593335	-1.69996	3.16E-11	6.82E-11
COMTD1	1.7583534	3.412467	2.12E-37	1.41E-36
LGALS7B	1.7577138	1.177679	1.30E-06	2.18E-06
MYCN	1.7567389	1.569167	7.40E-17	2.12E-16
PAQR6	1.7566092	2.818246	3.31E-29	1.63E-28
VSIG1	1.7554222	0.23915	4.64E-21	1.61E-20
PHF21B	1.7511508	0.941369	3.39E-09	6.59E-09
GPR143	1.7507705	2.487732	1.90E-30	9.82E-30
LRRC10B	1.7498483	1.840197	1.58E-18	4.87E-18
SUSD4	1.7493716	5.174779	1.37E-24	5.51E-24
RASSF7	1.7493601	5.013274	4.88E-47	4.39E-46
UPK2	1.7487618	-0.39475	7.19E-16	1.97E-15
GCM1	1.7476313	-0.46609	9.75E-12	2.16E-11
ATG9B	1.7474099	1.865985	7.33E-31	3.86E-30
CBX4	1.747404	5.843662	4.59E-68	6.97E-67
DDIAS	1.7468428	3.143553	2.18E-48	2.03E-47

Gene names	logFC	log2CPM	P.Value	adj.P.Val
CATIP	1.7460517	-0.5267	3.53E-20	1.17E-19
ESR1	1.7456491	9.019752	2.72E-15	7.24E-15
DNA2	1.7446202	3.3375	5.57E-63	7.56E-62
CENPW	1.7434715	3.552912	3.68E-29	1.81E-28
TREML4	1.7420615	-3.44338	1.27E-10	2.66E-10
SLC23A1	1.7418739	1.056511	4.07E-26	1.76E-25
RASL11B	1.7418513	2.973996	2.54E-40	1.87E-39
CCDC154	1.7408738	0.991183	8.46E-26	3.61E-25
DRP2	1.7381356	0.265364	6.37E-36	4.03E-35
KRT4	1.7374469	3.185958	0.0001507	0.00022098
PLAUR	1.735892	5.283144	8.48E-53	8.81E-52
CASP16	1.7350285	0.662965	3.56E-19	1.13E-18
KLK13	1.7339397	1.50365	1.16E-06	1.95E-06
SLC39A6	1.7338395	10.28045	2.16E-21	7.57E-21
HEPHL1	1.7335624	0.073842	9.13E-13	2.14E-12
BOLA2B	1.7330677	-0.94392	1.57E-37	1.05E-36
SH3GL2	1.7326137	-0.22957	1.23E-10	2.58E-10
F2RL2	1.7311078	4.937544	1.06E-23	4.09E-23
IL27	1.7286957	-1.84177	1.38E-30	7.21E-30
MAT1A	1.726702	1.620228	2.57E-11	5.55E-11
PRTN3	1.7266719	-1.4692	1.91E-08	3.56E-08
TMEM121	1.7266169	2.471049	3.42E-30	1.75E-29
ACMSD	1.726313	0.294337	1.24E-11	2.73E-11
CKMT1A	1.7238776	2.196556	1.10E-18	3.41E-18
ARHGAP39	1.7224967	4.765394	2.51E-73	4.22E-72
MRPS30	1.7220925	7.094114	2.40E-26	1.05E-25
ENSG00000159753	1.7214152	2.359651	4.84E-22	1.75E-21
NPY1R	1.7211887	7.963681	3.48E-08	6.39E-08

Gene names	logFC	log2CPM	P.Value	adj.P.Val
CCR4	1.7204339	1.943532	1.58E-24	6.36E-24
ELOVL2	1.7198345	5.457238	2.41E-12	5.52E-12
EVPL	1.7178987	5.791684	1.65E-52	1.70E-51
PCDH8	1.7175726	-0.43375	4.32E-07	7.45E-07
ZNF367	1.7163091	3.479139	3.50E-57	4.09E-56
SPRR2A	1.7137471	-0.16466	0.0102632	0.0131645
C4orf26	1.7129776	-2.57345	3.38E-14	8.51E-14
ADAM19	1.7118782	4.59707	3.14E-51	3.15E-50
CREB3L4	1.7118548	7.036004	2.76E-48	2.56E-47
ZNF804B	1.7113418	-1.9678	0.000195	0.00028368
TRIB3	1.7098905	5.593307	1.25E-62	1.69E-61
SMYD3	1.7089124	2.649149	1.73E-58	2.08E-57
LRRC56	1.7084156	3.353347	7.42E-40	5.37E-39
TMEM158	1.7074184	3.446194	1.14E-18	3.53E-18
KLF1	1.7072384	-2.53384	2.91E-31	1.56E-30
FOXO6	1.7064743	2.72051	1.18E-45	1.02E-44
KRT18	1.7054909	9.678788	4.33E-40	3.16E-39
GALNT5	1.7054779	4.487232	6.68E-17	1.92E-16
SIAH2	1.7047263	6.772568	1.58E-46	1.40E-45
SYP	1.7047089	3.023945	2.61E-15	6.97E-15
FCGR1A	1.7041768	2.475348	9.45E-48	8.64E-47
FBN3	1.7028918	1.815494	2.19E-09	4.30E-09
C9orf171	1.7025749	-1.40438	1.40E-11	3.08E-11
OAS2	1.7016969	6.98902	6.55E-29	3.18E-28
C6orf141	1.6996971	3.947817	1.12E-15	3.04E-15
PAX9	1.6995863	3.217523	1.83E-22	6.72E-22
MAL2	1.697737	8.335789	1.61E-48	1.50E-47
KIAA1467	1.697688	6.786474	8.43E-21	2.88E-20
PPP1R1C	1.6971766	-0.13401	1.03E-08	1.95E-08

Gene names	logFC	log2CPM	P.Value	adj.P.Val
ASIC2	1.6966848	0.23121	2.05E-08	3.80E-08
SLC35D3	1.6964831	-2.43014	5.17E-10	1.05E-09
ABCC12	1.693904	1.288546	5.74E-07	9.81E-07
CAPNS2	1.6932506	-1.17397	2.21E-06	3.65E-06
SHD	1.6916658	-2.92971	1.87E-11	4.09E-11
SLC4A8	1.6907706	4.887942	3.54E-21	1.23E-20
CALCB	1.6898798	-2.31166	7.93E-08	1.43E-07
MXD3	1.6884189	3.165698	2.20E-45	1.89E-44
FAM189A1	1.6860953	0.782602	1.58E-13	3.83E-13
SERPINA11	1.6850877	3.910033	1.65E-09	3.27E-09
SH2D2A	1.6849101	2.176895	2.12E-28	1.01E-27
LRP8	1.6848748	3.836734	3.14E-33	1.81E-32
FOXD4	1.6846464	-0.69034	3.16E-32	1.75E-31
CELF4	1.6843508	0.916546	4.80E-13	1.14E-12
SMIM6	1.6821453	-1.72729	6.25E-13	1.48E-12
C5orf49	1.6812352	1.903844	8.97E-23	3.34E-22
CXCL13	1.6806841	5.764504	5.67E-08	1.03E-07
MFSD3	1.6803352	4.927881	1.03E-45	8.97E-45
HAO2	1.6782546	0.432353	6.78E-06	1.08E-05
SULT4A1	1.6772323	0.755165	1.11E-11	2.46E-11
ADAMTSL2	1.6770576	3.295503	8.35E-47	7.41E-46
SH2D5	1.6766598	-1.92543	5.27E-17	1.52E-16
FUT2	1.676139	3.290722	6.83E-28	3.17E-27
KLHDC9	1.6758775	3.489748	7.99E-32	4.37E-31
TEX101	1.6736526	-2.11769	1.61E-09	3.19E-09
WFDC10B	1.6717089	-2.90369	2.46E-09	4.82E-09
TMEM63C	1.6714059	4.925731	2.03E-25	8.51E-25
MUC6	1.6672986	5.9503	1.59E-05	2.48E-05

Gene names	logFC	log2CPM	P.Value	adj.P.Val
LIMD2	1.6645878	4.379217	1.18E-32	6.65E-32
CCDC87	1.6644448	1.07211	2.88E-37	1.91E-36
ASIC3	1.6636705	1.223703	6.75E-29	3.27E-28
LRFN1	1.6621325	2.752722	2.58E-38	1.78E-37
FEN1	1.661174	5.391533	4.22E-72	6.99E-71
FADS2	1.6610119	7.587994	5.56E-17	1.60E-16
FAP	1.6605159	5.480761	1.47E-38	1.02E-37
ESRP1	1.6598275	7.368073	4.13E-64	5.78E-63
CDHR2	1.6575622	-0.61965	3.09E-30	1.58E-29
FAM155B	1.6564038	2.102595	2.01E-21	7.05E-21
BCL2A1	1.6555387	2.479788	5.76E-17	1.66E-16
HIST1H2BN	1.6553954	1.801149	8.31E-33	4.71E-32
CKMT1B	1.6539464	2.396949	2.08E-16	5.84E-16
SIX4	1.65389	4.618796	5.81E-45	4.93E-44
WFDC5	1.6534213	-1.87336	0.0001849	0.00026948
BIK	1.6532732	3.760592	2.26E-34	1.37E-33
GALE	1.6522067	5.303999	1.45E-59	1.79E-58
MANEAL	1.6509992	4.417376	2.09E-40	1.54E-39
CCR7	1.6509752	3.001747	1.05E-17	3.13E-17
CXCR3	1.6489567	2.253133	4.89E-21	1.69E-20
TLL2	1.6489205	0.245218	6.26E-27	2.79E-26
C1orf106	1.6485198	3.508003	1.50E-13	3.65E-13
FAM195A	1.6484156	4.929239	7.41E-49	6.97E-48
SEC14L4	1.6482143	-1.27185	2.72E-07	4.74E-07
SLC45A2	1.6479327	-2.23429	7.31E-20	2.38E-19
CASKIN1	1.647544	1.426528	8.33E-26	3.56E-25
ARHGAP11B	1.6466429	2.46298	2.47E-50	2.42E-49
MORN3	1.6464971	2.007713	3.31E-35	2.05E-34
TLE6	1.6451977	2.335879	6.43E-29	3.12E-28

Gene names	logFC	log2CPM	P.Value	adj.P.Val
SCGB2A2	1.6443753	10.22621	6.68E-06	1.07E-05
ZNF296	1.6440947	2.67785	8.53E-40	6.16E-39
EGLN3	1.6439294	5.58929	3.93E-25	1.62E-24
EXOC3L4	1.6415117	-0.01964	2.05E-16	5.77E-16
RBBP8NL	1.6414794	3.176564	1.34E-43	1.10E-42
MIF	1.6407488	5.348299	8.30E-37	5.41E-36
DNAH14	1.6405213	3.802629	4.66E-61	6.01E-60
GOLT1A	1.6401903	2.815384	3.13E-33	1.80E-32
PRSS27	1.6401865	2.252798	4.75E-25	1.95E-24
TSPAN13	1.6392367	8.305284	2.70E-43	2.18E-42
AZU1	1.638762	0.313865	4.00E-11	8.59E-11
C19orf81	1.6373781	-2.18975	6.32E-10	1.28E-09
NRSN1	1.6373607	-1.90385	1.61E-05	2.51E-05
C1orf186	1.6371852	2.258901	4.67E-11	9.99E-11
IL36G	1.636717	-3.10122	1.87E-06	3.09E-06
ATP2A3	1.6355263	6.695243	7.30E-28	3.39E-27
CD19	1.6347522	1.112231	7.74E-11	1.64E-10
ERP27	1.6335524	4.420001	1.63E-20	5.48E-20
ACRV1	1.6330134	-1.75395	5.49E-27	2.45E-26
CRB3	1.6325897	3.961207	2.31E-67	3.44E-66
IL11	1.6324955	0.16847	1.78E-31	9.61E-31
SPAG4	1.6303707	2.495712	3.84E-34	2.29E-33
ADORA2A	1.6300505	-0.88228	2.61E-22	9.51E-22
RSAD2	1.6296603	5.354071	2.48E-24	9.85E-24
PRRT2	1.6272529	2.644755	1.36E-26	6.00E-26
SP5	1.6257524	1.127459	4.13E-10	8.46E-10
SIRPG	1.6254196	1.357719	4.20E-18	1.27E-17
NAT2	1.624731	0.182827	3.53E-10	7.24E-10

Gene names	logFC	log2CPM	P.Value	adj.P.Val
HOXB6	1.6242075	3.137963	6.04E-14	1.50E-13
FABP7	1.6229154	5.465055	0.0001987	0.0002889
SDR9C7	1.6217361	-2.28964	5.64E-08	1.02E-07
ENKUR	1.6203879	-0.36613	1.85E-20	6.22E-20
LSR	1.6194781	7.26202	1.29E-62	1.73E-61
PARPBP	1.6184044	2.917049	5.87E-60	7.36E-59
RNF223	1.6180525	1.897189	2.21E-23	8.39E-23
SERPINB10	1.6177076	-3.08689	1.55E-06	2.59E-06
KIF24	1.6169353	3.37692	2.50E-74	4.27E-73
RELL2	1.6155044	1.095646	8.77E-42	6.76E-41
CATSPER1	1.614462	-0.79614	4.91E-37	3.23E-36
GJB6	1.6135224	0.820021	5.78E-06	9.27E-06
KRTAP5_10	1.611423	-1.40266	3.04E-16	8.47E-16
AP1M2	1.6112726	6.796085	1.16E-57	1.38E-56
P3H4	1.6109016	5.419221	1.72E-69	2.72E-68
HID1	1.6101406	7.368317	6.67E-38	4.52E-37
PRDM12	1.6096738	-2.49303	5.12E-24	2.00E-23
GALNT6	1.6095407	7.764384	1.51E-25	6.35E-25
DOC2A	1.6083253	2.240078	1.72E-18	5.28E-18
RP1	1.6079277	-0.1499	5.60E-10	1.14E-09
WDR34	1.6074561	6.543496	3.41E-69	5.34E-68
DDN	1.6071521	-0.59827	5.80E-12	1.30E-11
DLGAP3	1.6066396	0.156394	1.58E-18	4.86E-18
C3orf52	1.6030982	4.077705	3.76E-28	1.77E-27
RAB31	1.6030648	7.613787	2.45E-36	1.57E-35
SLAMF8	1.6024075	4.044496	4.71E-30	2.39E-29
LEMD1	1.6017546	0.491495	1.39E-06	2.32E-06
SLC2A6	1.6003091	2.780867	3.69E-32	2.04E-31
SLC6A9	1.5996029	4.885556	6.70E-32	3.67E-31

Gene names	logFC	log2CPM	P.Value	adj.P.Val
PPP1R14B	1.5982245	6.209747	5.14E-50	4.97E-49
HIST1H2AC	1.5981592	6.725759	1.05E-35	6.63E-35
VCAN	1.5980998	8.765375	2.21E-32	1.23E-31
COL1A2	1.5977745	12.28705	1.62E-29	8.08E-29
CD24	1.5975712	10.14905	1.81E-20	6.09E-20
CACNA1E	1.5973516	-0.71775	2.84E-19	9.05E-19
SLC22A10	1.5970514	-2.12426	1.09E-08	2.06E-08
HRASLS2	1.5967731	2.190136	6.18E-16	1.70E-15
HMGA1	1.5963762	7.152199	1.92E-45	1.65E-44
GGH	1.5963568	5.060939	2.69E-27	1.22E-26
BOP1	1.5962543	6.065755	5.49E-41	4.13E-40
CCKAR	1.5952249	-3.09508	1.45E-09	2.89E-09
HGFAC	1.5949368	-1.39829	7.49E-14	1.85E-13
NME2	1.5948152	0.183439	1.20E-26	5.29E-26
TEX35	1.5938001	-3.37108	1.46E-09	2.91E-09
CLEC4D	1.5924951	-1.59388	2.25E-08	4.18E-08
RASGRP1	1.5897715	4.244091	7.88E-23	2.94E-22
DPP10	1.5883245	-0.17098	4.83E-05	7.30E-05
LRRN2	1.5881158	5.028317	1.92E-27	8.75E-27
CALHM3	1.5872688	-3.11543	2.12E-10	4.40E-10
SLC25A22	1.5858653	5.146811	2.96E-68	4.53E-67
OR13J1	1.5852698	-2.76532	3.75E-13	8.96E-13
HMX1	1.5832534	-2.38688	8.43E-07	1.43E-06
LRRC26	1.5823376	2.110049	1.55E-11	3.40E-11
TRIM46	1.5822706	2.609383	1.30E-30	6.80E-30
MLPH	1.5819842	8.395538	1.01E-20	3.44E-20
CELSR1	1.581404	7.887943	2.68E-31	1.44E-30
KAZALD1	1.5805448	4.049611	5.97E-24	2.33E-23

Gene names	logFC	log2CPM	P.Value	adj.P.Val
CHEK1	1.5789885	4.175957	5.88E-50	5.68E-49
MAG	1.5762337	0.408856	4.08E-10	8.35E-10
OAS3	1.5756897	7.191284	6.64E-33	3.77E-32
POLE2	1.5748972	2.734636	5.47E-71	8.89E-70
LRRN4	1.5747162	-0.39294	2.93E-08	5.41E-08
LCTL	1.5732799	-0.88779	2.63E-25	1.10E-24
CYP27B1	1.5724029	2.129916	3.38E-23	1.28E-22
POLR3K	1.5720621	4.759952	7.78E-83	1.54E-81
ENO2	1.5718512	5.644236	2.31E-28	1.10E-27
RAD54B	1.5708059	2.274231	3.04E-52	3.12E-51
DNAJC1	1.5707213	6.550023	7.41E-40	5.37E-39
HSPA6	1.5703116	3.163452	5.09E-20	1.67E-19
PTPRN2	1.5697991	4.830039	8.08E-12	1.80E-11
ARSI	1.5668085	2.345069	1.96E-27	8.91E-27
BRCA2	1.5668055	3.594559	5.98E-48	5.49E-47
CDH11	1.566732	7.108812	3.47E-38	2.38E-37
HSPB1	1.5664854	9.141771	3.18E-27	1.43E-26
FAM183A	1.5664306	0.500163	6.10E-18	1.84E-17
TPRG1	1.5663619	6.00624	4.34E-11	9.31E-11
CCK	1.565959	-2.63656	2.16E-05	3.35E-05
GJB3	1.5644958	2.034473	1.30E-08	2.45E-08
SPDEF	1.5639752	7.559428	3.41E-19	1.08E-18
TMEM97	1.5635402	5.381097	2.59E-42	2.03E-41
SEZ6L2	1.5621923	6.331148	2.19E-21	7.69E-21
ENSG00000141854	1.5606692	3.858962	4.82E-32	2.65E-31
REPS2	1.5605106	6.240236	1.49E-21	5.26E-21
NPFFR2	1.5603072	-0.60201	1.82E-08	3.39E-08
TIMELESS	1.5601441	5.704699	1.69E-81	3.28E-80
SLC12A8	1.5597717	4.551622	2.54E-69	4.00E-68

Gene names	logFC	log2CPM	P.Value	adj.P.Val
CHAD	1.559209	5.332609	7.29E-10	1.47E-09
TAP1	1.5587541	7.153551	2.99E-32	1.66E-31
HCN4	1.5581061	-1.21273	1.83E-09	3.62E-09
PCP4	1.5580976	0.369932	5.27E-08	9.59E-08
EVA1B	1.5577457	4.70325	3.34E-23	1.26E-22
P2RY6	1.5576605	2.514725	8.51E-30	4.28E-29
ANKRD62	1.5566763	-3.31078	5.11E-09	9.83E-09
ZNF467	1.5565084	4.525535	3.05E-43	2.45E-42
HS3ST5	1.556382	-0.92748	8.57E-06	1.36E-05
SORCS1	1.5557592	3.18042	7.96E-08	1.43E-07
CHRNB4	1.5551048	-1.34117	1.77E-10	3.69E-10
GDAP1L1	1.5534183	-1.4567	2.69E-10	5.56E-10
C5AR2	1.5532625	4.40405	3.26E-22	1.19E-21
SLC2A14	1.5532427	0.16062	2.59E-08	4.79E-08
PRSS8	1.5529387	7.766577	1.47E-38	1.02E-37
SLC2A1	1.5522938	6.928218	9.54E-39	6.64E-38
PCSK4	1.5522319	2.445297	4.00E-24	1.58E-23
SLC5A7	1.5518589	-0.59183	0.0001755	0.00025607
NME3	1.5504225	5.818752	1.21E-35	7.59E-35
CLDN14	1.5503453	-0.51432	3.19E-20	1.06E-19
TOX3	1.5496104	4.804828	3.85E-15	1.02E-14
PHLDA2	1.549179	3.246374	7.25E-24	2.82E-23
LRRC45	1.5491709	4.870951	1.07E-47	9.74E-47
EXOSC4	1.5487432	4.751477	1.81E-42	1.42E-41
ROGDI	1.5482437	5.499004	1.32E-59	1.64E-58
ENSG00000271810	1.5480536	-2.76595	2.23E-17	6.57E-17
DPP3	1.5478316	6.067358	1.44E-79	2.70E-78
DDC	1.5477759	0.828159	1.35E-05	2.12E-05

Gene names	logFC	log2CPM	P.Value	adj.P.Val
APOBEC3A	1.5473201	0.509732	6.95E-13	1.64E-12
RTN4RL2	1.5466624	2.204583	2.51E-40	1.85E-39
AGT	1.5459867	4.455412	5.40E-12	1.21E-11
RELT	1.5457712	3.081923	8.47E-60	1.05E-58
LEFTY1	1.5438277	-1.25498	4.16E-12	9.40E-12
CCDC74B	1.542972	2.973891	2.92E-16	8.15E-16
DLX3	1.5428564	2.387308	5.07E-17	1.46E-16
DSCC1	1.5423155	3.509161	3.16E-38	2.17E-37
SDF2L1	1.5421985	4.365012	6.12E-43	4.88E-42
RD3	1.5414911	-2.77666	3.22E-11	6.94E-11
MTCL1	1.5410131	4.105981	1.24E-25	5.22E-25
SELL	1.5408979	4.352233	1.94E-17	5.73E-17
STX1A	1.5401333	3.054333	4.43E-46	3.87E-45
DDX39A	1.540027	6.048354	4.42E-66	6.46E-65
SECTM1	1.538978	5.185526	9.44E-26	4.02E-25
MAGEB17	1.5384745	-0.24779	6.49E-09	1.24E-08
SMC4	1.5378867	6.034633	2.13E-47	1.93E-46
RP11_514O12.4	1.5371834	-1.5591	1.75E-31	9.43E-31
DNMT3B	1.5369107	3.26914	9.03E-37	5.88E-36
ADAM2	1.535537	-1.21408	0.0051493	0.0067636
FCRL3	1.5344801	1.378199	8.44E-12	1.88E-11
EVL	1.5336832	8.095765	7.59E-31	4.00E-30
MESP2	1.533633	1.265429	1.35E-16	3.84E-16
CD207	1.5335053	1.924873	6.23E-12	1.40E-11
PLEK2	1.5326161	3.165754	1.03E-38	7.16E-38
CALCA	1.5319716	-1.6448	0.0006601	0.00092553
ZYG11A	1.5310458	2.652057	1.29E-25	5.44E-25
TUFT1	1.5298873	5.992203	2.77E-69	4.34E-68
DNAJC22	1.5291895	4.196482	1.08E-19	3.50E-19

Gene names	logFC	log2CPM	P.Value	adj.P.Val
MICAL2	1.5281336	6.706716	8.53E-58	1.01E-56
CBX8	1.5269806	3.664564	2.37E-57	2.78E-56
FAM110A	1.526156	5.165493	1.02E-53	1.08E-52
CRIP1	1.5261204	2.167515	1.24E-24	5.00E-24
HGH1	1.5259602	5.600456	1.33E-60	1.69E-59
COL12A1	1.5257516	9.228851	6.91E-28	3.21E-27
SLC6A17	1.5243358	1.529043	1.51E-11	3.31E-11
RAB25	1.5240239	6.919941	6.65E-52	6.79E-51
HIST4H4	1.5230416	1.898753	8.69E-20	2.83E-19
OAS1	1.5225735	6.223407	2.73E-27	1.24E-26
AXDND1	1.5222013	-1.37938	2.44E-18	7.44E-18
UCN	1.5218047	0.281209	6.73E-27	3.00E-26
DLX1	1.5215783	1.93569	1.01E-06	1.70E-06
NCAM2	1.5208751	4.353143	6.53E-14	1.62E-13
QPRT	1.5202847	4.759677	1.67E-21	5.89E-21
KCNK6	1.5202424	5.952121	1.71E-36	1.10E-35
RND1	1.5184401	4.332402	7.20E-22	2.58E-21
TMEM184A	1.518238	4.586549	1.31E-34	7.96E-34
SLC1A2	1.5174226	4.433653	1.25E-13	3.05E-13
C6orf211	1.5166666	6.9778	1.28E-21	4.53E-21
RAB19	1.51666	0.525924	7.02E-29	3.40E-28
H2AFZ	1.5164048	7.589715	1.10E-79	2.08E-78
TRIM71	1.5162734	-1.80651	1.78E-09	3.52E-09
GRB7	1.515838	6.062043	5.56E-17	1.60E-16
TMC3	1.5139825	1.219848	1.79E-08	3.34E-08
NOD2	1.513687	3.355188	3.47E-33	2.00E-32
VNN3	1.5129497	-0.93494	7.22E-07	1.23E-06
FKBP4	1.5128513	8.676594	1.18E-60	1.51E-59

Gene names	logFC	log2CPM	P.Value	adj.P.Val
TEKT4	1.5124357	-1.75559	1.02E-10	2.16E-10
ETNK2	1.5119248	5.739181	3.35E-26	1.45E-25
FLVCR1	1.5114975	4.804891	1.14E-77	2.07E-76
SLC6A15	1.5114199	1.477457	4.91E-05	7.41E-05
ANO9	1.5108053	4.186598	3.80E-34	2.27E-33
GRK1	1.5106979	-2.75097	1.08E-09	2.16E-09
FANCD2	1.5092277	4.634177	1.80E-62	2.41E-61
TMEM79	1.5084684	4.815015	1.19E-51	1.20E-50
STRA13	1.5083745	5.446888	3.00E-48	2.78E-47
BHLHA15	1.5078734	0.00515	1.72E-15	4.63E-15
MTHFD2	1.5064848	6.729399	1.74E-69	2.75E-68
KIF22	1.5053863	6.238229	1.80E-84	3.66E-83
POTEH	1.5047421	-3.3232	1.94E-08	3.62E-08
DHDH	1.5044941	0.053051	2.15E-20	7.20E-20
EME2	1.5019345	4.209824	9.36E-36	5.90E-35
CACNA1I	1.5007374	1.339131	1.02E-14	2.63E-14
RIMKLA	1.4995485	2.64786	2.17E-18	6.66E-18
PLA2G4F	1.4985067	2.676317	6.59E-19	2.06E-18
RUSC1	1.4983837	6.12089	4.54E-92	1.03E-90
CYP2B6	1.4977694	-1.37265	8.90E-06	1.41E-05
EGR4	1.4938295	-1.29872	6.03E-08	1.09E-07
MARCKSL1	1.493246	8.570148	8.80E-68	1.32E-66
GZMB	1.493118	2.049553	1.91E-12	4.40E-12
ANO7	1.4929496	1.597348	5.56E-21	1.92E-20
SH2D6	1.4929171	-1.45919	1.27E-24	5.11E-24
EVX1	1.4920171	-2.08479	5.35E-07	9.16E-07
MRPL12	1.4916427	4.288049	3.76E-30	1.92E-29
HES7	1.4909473	-2.55394	4.79E-11	1.02E-10
CREG2	1.4903168	-0.8276	2.26E-20	7.56E-20

Gene names	logFC	log2CPM	P.Value	adj.P.Val
CHAC1	1.4892364	1.669554	1.05E-20	3.55E-20
STXBP2	1.4879935	5.828728	1.03E-56	1.18E-55
C5orf38	1.4874006	4.33721	5.47E-14	1.36E-13
PRSS22	1.4868357	3.830569	6.91E-33	3.92E-32
MBOAT2	1.4853997	6.522653	2.49E-39	1.78E-38
MST1R	1.484562	4.400819	5.72E-36	3.63E-35
CBLC	1.4838187	4.338841	1.22E-26	5.37E-26
DRAXIN	1.4822024	1.147219	1.78E-18	5.48E-18
SYCP2	1.4806272	5.375472	8.04E-22	2.87E-21
PSMD3	1.4792651	7.930684	6.19E-27	2.76E-26
OPRK1	1.4791505	-0.24444	5.16E-05	7.78E-05
WFIKKN1	1.4782152	0.27956	1.04E-22	3.85E-22
GZMK	1.4781405	2.543125	1.85E-14	4.74E-14
DNAH3	1.4780935	0.119765	4.08E-11	8.77E-11
C9orf117	1.4778075	2.192292	7.52E-45	6.34E-44
MDK	1.4777528	7.510181	3.86E-27	1.74E-26
SAC3D1	1.4771633	3.829077	7.49E-44	6.14E-43
SLC39A11	1.4749148	6.647111	2.95E-48	2.73E-47
DRD4	1.4739668	-0.19969	4.42E-24	1.74E-23
SERPINC1	1.4739347	-1.68003	5.94E-19	1.86E-18
GSG1L	1.4736215	-1.17994	1.22E-07	2.16E-07
SPATC1	1.473331	-1.55735	1.06E-25	4.51E-25
ENSG00000276418	1.4720217	-1.39877	5.90E-31	3.13E-30
ZNF552	1.4717225	6.053129	4.97E-26	2.13E-25
SHISA7	1.4709983	-2.06893	6.58E-09	1.26E-08
APOBR	1.4703109	4.183287	9.83E-26	4.18E-25
CLDN3	1.4701904	6.377174	2.24E-20	7.47E-20
C2CD4D	1.4698467	1.115814	7.03E-28	3.26E-27

Gene names	logFC	log2CPM	P.Value	adj.P.Val
CACFD1	1.4692434	5.679806	1.56E-61	2.03E-60
CACNB3	1.4687694	5.502891	6.12E-62	8.12E-61
IFIT1	1.468557	6.640241	2.04E-16	5.74E-16
SERPINB2	1.4673137	1.064885	2.61E-06	4.27E-06
MROH9	1.4657384	-2.7873	1.97E-07	3.46E-07
PNPLA1	1.4656893	-2.70374	6.31E-13	1.49E-12
BSND	1.465551	-2.64887	3.51E-09	6.82E-09
SPRR1A	1.4641783	-0.70609	0.0021154	0.00286329
MBOAT7	1.463333	7.011493	3.11E-69	4.87E-68
TG	1.4619915	0.191515	4.86E-19	1.53E-18
CHPF	1.4600798	7.150431	6.67E-57	7.72E-56
KRT72	1.4600195	-2.90009	4.43E-06	7.16E-06
SLC12A3	1.4598745	-0.58678	5.41E-10	1.10E-09
NACC1	1.4587789	6.685523	8.35E-104	2.34E-102
ST18	1.4582639	-1.10469	4.44E-14	1.11E-13
COL24A1	1.4577712	1.685017	1.02E-34	6.21E-34
FXYD3	1.4577498	7.817084	2.49E-28	1.18E-27
TUBA1C	1.4573666	6.631849	5.50E-71	8.94E-70
HIST1H3E	1.4568123	0.715709	8.54E-23	3.18E-22
EEF1A2	1.4558787	6.771386	9.96E-09	1.89E-08
FAM155A	1.455419	2.285476	5.20E-15	1.36E-14
YDJC	1.4549834	4.252498	1.76E-50	1.73E-49
SAG	1.4544984	-1.78725	4.15E-12	9.38E-12
NECAB3	1.4535983	5.537166	7.49E-40	5.42E-39
CLEC11A	1.45257	4.092932	1.48E-27	6.77E-27
C4A	1.4524807	6.106692	2.03E-14	5.19E-14
IGFL1	1.4493557	-0.16977	3.98E-07	6.87E-07
TRIM58	1.4486398	1.780316	1.29E-11	2.83E-11
CORO2A	1.4479763	5.801517	4.51E-43	3.61E-42

Gene names	logFC	log2CPM	P.Value	adj.P.Val
CHRD	1.4479161	4.823922	4.53E-16	1.25E-15
CDK5	1.4478893	4.34948	1.96E-75	3.45E-74
TMEM206	1.4471426	3.83927	1.52E-75	2.68E-74
RIMS1	1.4458231	1.874326	3.29E-09	6.41E-09
RAB3A	1.4454895	2.815318	3.96E-39	2.79E-38
RPL39L	1.4449779	4.001279	2.31E-32	1.29E-31
EPCAM	1.4441605	7.924734	1.46E-41	1.12E-40
PLA2G2D	1.4434027	2.482799	1.99E-08	3.71E-08
HMSD	1.4418191	-1.79284	1.78E-11	3.88E-11
ZSCAN1	1.4405244	0.524177	1.59E-11	3.48E-11
OSM	1.4402436	1.490613	8.42E-21	2.87E-20
PCNA	1.4401824	6.930942	2.39E-74	4.09E-73
LIME1	1.4400846	1.217638	2.52E-29	1.25E-28
ACOT7	1.439987	4.980305	1.10E-48	1.03E-47
EPHA10	1.4387367	3.057769	1.10E-25	4.67E-25
CCDC150	1.4380235	1.176029	3.39E-31	1.81E-30
PRR15L	1.4377731	5.77891	1.48E-24	5.95E-24
CYP4Z1	1.43766	6.2905	1.15E-05	1.81E-05
PGAP3	1.436748	6.521518	1.76E-19	5.63E-19
ENSG00000274897	1.4366286	-0.4329	3.53E-38	2.42E-37
TIFAB	1.4359236	-0.08084	2.30E-13	5.55E-13
SPINT2	1.4359179	9.126868	2.67E-54	2.90E-53
SGOL2	1.4357377	3.802093	5.63E-58	6.71E-57
MRPS34	1.4344991	6.232181	7.61E-59	9.27E-58
LSM4	1.4337415	6.665549	6.12E-70	9.76E-69
SCRG1	1.4333368	2.401681	1.67E-06	2.78E-06
TTC39A	1.4330477	7.023023	2.64E-25	1.10E-24
AKAP5	1.4329244	3.438127	3.88E-22	1.41E-21

Gene names	logFC	log2CPM	P.Value	adj.P.Val
C4B	1.4322437	6.245494	4.98E-14	1.25E-13
KRT10	1.4321704	5.373727	1.98E-18	6.08E-18
NR2E1	1.4319308	-1.72917	0.0001731	0.00025275
SYT17	1.4306678	4.540883	1.28E-18	3.96E-18
VPREB3	1.4282702	-0.02719	2.10E-11	4.57E-11
CHAF1B	1.4281173	4.495795	3.61E-52	3.71E-51
GGCT	1.4276139	6.384179	1.46E-53	1.55E-52
REEP4	1.4269878	4.85892	7.66E-58	9.09E-57
MCEMP1	1.4269877	-1.20416	6.31E-12	1.41E-11
ABHD17C	1.4267598	5.066209	6.60E-44	5.43E-43
VAV3	1.4265285	7.579649	4.75E-24	1.86E-23
LMNB2	1.4255137	6.2387	4.12E-61	5.31E-60
CREB3L3	1.4254681	-3.34149	1.60E-10	3.34E-10
RXFP1	1.4241344	-1.83595	6.59E-15	1.72E-14
RPUSD1	1.4229692	4.917168	2.24E-62	3.00E-61
IGLL1	1.4222635	-2.28313	2.81E-06	4.60E-06
RGAG1	1.4217051	0.330286	1.31E-08	2.47E-08
RARRES3	1.4216834	6.968545	9.40E-21	3.20E-20
MKX	1.4211102	3.408484	5.80E-07	9.91E-07
SERINC2	1.4209288	7.631982	2.24E-37	1.49E-36
C12orf42	1.4191557	-2.07195	4.39E-10	8.98E-10
ITIH6	1.4190631	2.922059	3.27E-13	7.83E-13
DONSON	1.4187745	4.174739	3.40E-60	4.29E-59
TBC1D3D	1.4184891	-2.85306	0.0029633	0.00396294
LOXL1	1.4175565	5.861033	1.10E-38	7.65E-38
EPS8L1	1.4170798	5.314036	1.66E-30	8.61E-30
APOF	1.4163009	-0.49103	1.43E-08	2.69E-08
CKAP2	1.4162105	5.257266	1.76E-60	2.23E-59
CACNA1D	1.4158269	4.686267	5.33E-18	1.61E-17

Gene names	logFC	log2CPM	P.Value	adj.P.Val
WNK4	1.4153503	5.089796	9.45E-10	1.90E-09
TDRD1	1.4150208	2.125573	1.91E-06	3.16E-06
LAMTOR2	1.4143462	5.968662	7.53E-60	9.40E-59
CDCA7	1.4136974	3.453389	2.58E-13	6.21E-13
TTLL12	1.4134506	6.78087	2.26E-39	1.61E-38
EIF4EBP1	1.4131791	5.84062	1.83E-23	6.98E-23
UMOD	1.4127432	-1.06806	3.84E-08	7.05E-08
TRIM11	1.4119752	5.456818	5.42E-86	1.14E-84
FZD2	1.4119306	2.96768	2.43E-40	1.79E-39
NLRP6	1.4117771	-0.39027	3.39E-14	8.56E-14
LAIR2	1.4116933	-1.40349	4.06E-11	8.71E-11
COL3A1	1.4116516	12.1465	2.76E-23	1.05E-22
HOXB2	1.4090752	5.316995	5.27E-16	1.46E-15
C9orf84	1.4081899	-0.44288	1.12E-11	2.47E-11
NRIP3	1.4078501	4.906077	4.82E-10	9.84E-10
LMNTD2	1.4066593	2.020005	4.11E-23	1.55E-22
BAMBI	1.4064132	6.465325	5.08E-16	1.40E-15
CRYM	1.4063083	1.855657	5.38E-08	9.78E-08
PDIA2	1.4056068	-0.15651	7.07E-07	1.20E-06
RAC3	1.4052695	4.429097	5.04E-32	2.77E-31
SCGB1D2	1.405036	8.181386	0.0001047	0.00015508
ACOXL	1.4043674	-0.47732	4.32E-17	1.25E-16
ABRACL	1.4033985	5.407516	3.88E-40	2.83E-39
PPFIA3	1.4032925	4.009128	3.75E-30	1.91E-29
ATP6V0B	1.4029613	7.186045	1.62E-75	2.85E-74
CYFIP2	1.4028763	5.555936	1.73E-26	7.57E-26
BST2	1.4028097	7.837112	9.54E-22	3.39E-21
DHRS2	1.4026616	7.402181	3.30E-05	5.05E-05

Gene names	logFC	log2CPM	P.Value	adj.P.Val
CENPL	1.4006284	3.527562	4.32E-68	6.57E-67
SLC11A1	1.3994104	2.931945	2.30E-28	1.09E-27
BBC3	1.398485	3.680872	3.07E-37	2.04E-36
MAGEB4	1.3977222	-1.43835	0.0014952	0.00204545
CENPN	1.3969937	3.428524	5.68E-39	3.98E-38
GPR18	1.3969467	0.273339	4.16E-15	1.09E-14
STK31	1.3962516	-0.13691	1.59E-11	3.48E-11
ABHD11	1.3947208	5.881942	5.48E-46	4.77E-45
SLC52A3	1.3917529	4.600813	5.93E-31	3.14E-30
ZNF469	1.3912094	4.827008	2.23E-28	1.06E-27
C1orf64	1.390802	4.487265	4.80E-07	8.25E-07
CR1L	1.3900869	-2.22602	1.39E-12	3.23E-12
SPAG1	1.3900085	4.290972	2.56E-45	2.19E-44
APOC2	1.3885213	-1.99328	3.11E-20	1.03E-19
MZT2A	1.3883606	5.284116	3.59E-34	2.15E-33
RHCE	1.3882184	0.488741	2.26E-25	9.42E-25
ENSG00000271698	1.3881303	-3.18543	2.63E-17	7.71E-17
SLC22A31	1.3880065	-0.38876	2.01E-06	3.32E-06
SEMA7A	1.3868941	3.359778	1.20E-37	8.01E-37
C19orf26	1.3868722	0.313212	8.25E-29	3.99E-28
MMEL1	1.3867783	1.864791	4.22E-24	1.66E-23
PLAU	1.3850468	6.562706	6.14E-28	2.86E-27
PNLDC1	1.3837966	0.889324	4.55E-07	7.83E-07
LYZ	1.3836814	7.31229	5.53E-14	1.38E-13
GADD45G	1.3830946	3.789919	3.55E-19	1.13E-18
ADCK5	1.3823924	3.552276	5.52E-38	3.75E-37
FGL1	1.3819862	0.301378	8.91E-05	0.00013266
SNRNP25	1.3816787	5.325325	3.66E-61	4.74E-60
SRCIN1	1.3804846	4.847923	5.01E-22	1.81E-21

Gene names	logFC	log2CPM	P.Value	adj.P.Val
RASGRF1	1.3802544	2.045022	1.70E-12	3.93E-12
ATRNL1	1.3795966	3.378743	1.07E-07	1.91E-07
MZB1	1.3793994	4.75091	1.76E-12	4.06E-12
TEX22	1.3777175	0.018473	2.46E-24	9.80E-24
SNX22	1.3774029	3.107305	3.40E-18	1.03E-17
ADAP1	1.3765176	2.430768	1.05E-24	4.28E-24
CPA4	1.3757071	2.740285	6.24E-09	1.20E-08
MMP25	1.3755052	2.60553	7.41E-16	2.03E-15
LMTK3	1.3752089	3.675334	2.39E-20	7.98E-20
DNAI1	1.3749123	1.148182	1.22E-12	2.83E-12
CTPS1	1.3742916	5.25075	2.13E-45	1.84E-44
RAPGEFL1	1.3738979	4.565861	1.54E-16	4.36E-16
PPP4C	1.3737703	6.985197	2.54E-76	4.52E-75
LRFN4	1.3735655	4.384485	1.44E-27	6.61E-27
RPRM	1.3723393	-0.97671	3.63E-07	6.27E-07
CDK5R1	1.371229	2.693917	4.83E-34	2.87E-33
PCDH17	1.3707158	3.581329	7.46E-47	6.62E-46
GPR160	1.3689151	5.684711	1.24E-29	6.19E-29
NUDT16L1	1.3686785	5.287826	6.06E-61	7.78E-60
RUNX2	1.3673718	4.452834	9.22E-32	5.02E-31
APOA1BP	1.3666842	6.995004	3.78E-69	5.90E-68
HES4	1.366643	2.932767	3.76E-21	1.31E-20
MICB	1.3662553	3.336172	2.57E-28	1.22E-27
AIFM3	1.3662037	1.098677	1.26E-27	5.80E-27
CLEC7A	1.3661318	3.732847	2.20E-21	7.70E-21
GOLM1	1.3654176	8.061521	2.62E-35	1.63E-34
MZT2B	1.3643493	6.111801	1.83E-28	8.73E-28
C12orf74	1.363811	-2.78974	2.79E-06	4.57E-06

Gene names	logFC	log2CPM	P.Value	adj.P.Val
CCDC155	1.3637689	-1.86977	5.60E-07	9.59E-07
CLCN2	1.3636082	3.38517	1.30E-58	1.57E-57
DGAT2L6	1.363381	-2.96002	2.51E-05	3.88E-05
ZNF729	1.3627733	-1.57586	0.0001414	0.00020787
NVL	1.3613327	5.614115	1.18E-71	1.94E-70
CMPK2	1.3608524	4.270056	3.84E-21	1.33E-20
CCDC114	1.3603937	1.050817	1.72E-19	5.52E-19
LRRC52	1.3597975	-3.23471	1.67E-05	2.60E-05
TMED3	1.359684	7.452576	1.11E-63	1.54E-62
IL24	1.3594046	2.034118	3.05E-20	1.01E-19
SFXN5	1.3591192	5.044063	5.40E-31	2.86E-30
C14orf80	1.3589486	3.693171	1.47E-33	8.56E-33
C1orf112	1.3585167	3.791007	1.82E-61	2.37E-60
C11orf88	1.3583325	-1.46072	6.46E-10	1.31E-09
SLC35A2	1.3579297	5.807045	1.15E-100	3.01E-99
AP006285.2	1.3552246	-0.14295	3.14E-20	1.04E-19
OR8G5	1.3551957	-3.32394	0.0012875	0.00176964
PRKCG	1.3551552	-2.47879	4.55E-09	8.79E-09
HN1L	1.3550473	7.986217	4.39E-81	8.46E-80
SYNE4	1.3547385	4.347255	1.04E-33	6.10E-33
CCND1	1.354265	9.208945	4.01E-19	1.27E-18
BGLAP	1.3540572	-0.04362	2.90E-12	6.60E-12
TMEM8A	1.3536977	6.209823	3.65E-70	5.84E-69
CYB561	1.353337	7.736248	2.69E-45	2.30E-44
EN2	1.3517322	-0.34238	3.00E-06	4.90E-06
NFE2	1.3504905	0.516777	2.65E-12	6.05E-12
RETN	1.3504591	-2.29555	6.71E-09	1.28E-08
QPCT	1.3503559	3.954047	4.95E-19	1.56E-18
CERS2	1.3499089	8.399731	6.55E-63	8.86E-62

Gene names	logFC	log2CPM	P.Value	adj.P.Val
MRPL14	1.348983	5.614853	8.97E-54	9.56E-53
C19orf84	1.348818	-2.4628	1.59E-15	4.28E-15
NCR3LG1	1.3478866	1.790273	6.09E-16	1.68E-15
KCNG2	1.3475308	-2.03309	2.46E-16	6.90E-16
GRPR	1.3472946	3.584738	6.01E-07	1.03E-06
TMEM63B	1.346866	6.34161	1.01E-82	2.00E-81
PFKFB4	1.3464918	3.733576	2.52E-58	3.02E-57
LENEP	1.3463711	-2.79359	2.49E-13	6.01E-13
SLC30A2	1.3462929	1.20797	2.86E-08	5.28E-08
LAPTM4B	1.3455975	7.855693	1.13E-22	4.18E-22
MMP14	1.3451985	8.846221	6.47E-35	3.97E-34
NHLH2	1.3445684	-1.16703	5.61E-06	9.01E-06
CENPP	1.344482	3.541101	4.35E-40	3.17E-39
SLC2A10	1.3442258	6.505909	1.06E-27	4.89E-27
ENHO	1.344166	2.535997	6.18E-10	1.25E-09
ERN2	1.3422755	-1.32774	7.19E-05	0.00010768
WDR76	1.3421858	3.938867	1.64E-54	1.79E-53
PVRL2	1.3421341	7.865062	1.01E-59	1.25E-58
FLT3	1.3421019	2.818708	1.56E-10	3.26E-10
GAS2L3	1.342042	3.250905	2.46E-37	1.63E-36
C9orf142	1.3416738	4.920696	8.19E-37	5.35E-36
ANGPTL6	1.3414002	-1.0867	5.29E-33	3.02E-32
CTSD	1.3403247	10.03579	7.30E-30	3.68E-29
TMPRSS11A	1.3401006	-1.87648	0.0005125	0.00072423
GPR137C	1.3399711	2.04662	1.28E-33	7.48E-33
P2RX5	1.3393831	0.682605	3.42E-11	7.37E-11
FOXN4	1.3392152	-1.17263	5.46E-06	8.78E-06
LRRC59	1.3388621	7.715938	6.03E-47	5.39E-46

Gene names	logFC	log2CPM	P.Value	adj.P.Val
CANT1	1.3387372	7.406984	3.86E-52	3.96E-51
PGP	1.3386105	4.461742	2.35E-56	2.69E-55
SLC27A2	1.3376675	4.517339	4.14E-09	8.02E-09
CRABP1	1.33759	5.031568	5.87E-05	8.84E-05
PTPN7	1.3368906	3.322527	3.25E-22	1.18E-21
NKX1_2	1.3364937	0.618941	0.0004982	0.00070477
RTN4R	1.3363869	2.472213	1.22E-28	5.87E-28
TSTD1	1.3358261	6.159026	3.71E-39	2.62E-38
TRAIP	1.335391	2.755787	3.86E-59	4.74E-58
GRIK4	1.3353214	1.530684	7.43E-14	1.84E-13
SPIRE2	1.3346111	3.5812	3.53E-33	2.03E-32
LCN2	1.3335548	4.807731	3.56E-06	5.79E-06
DPF1	1.3328717	-0.20055	1.61E-24	6.45E-24
LRRC6	1.3323419	3.399291	4.08E-22	1.48E-21
NLRP2	1.3322002	5.041513	4.72E-08	8.60E-08
RFPL2	1.3321008	-2.67895	2.15E-13	5.21E-13
COL26A1	1.3318456	0.301973	2.07E-08	3.86E-08
LAD1	1.3312163	6.148383	3.43E-15	9.09E-15
SMIM1	1.3300699	1.476422	4.52E-23	1.70E-22
KRT83	1.3293181	0.159124	5.30E-05	8.00E-05
EFR3B	1.3286356	3.463841	6.72E-20	2.20E-19
IQCD	1.3284428	1.798632	7.76E-41	5.81E-40
LCT	1.3269663	-0.16341	6.89E-06	1.10E-05
ACE2	1.3267623	2.115715	4.01E-05	6.09E-05
TMEM54	1.3261165	5.734211	1.97E-44	1.64E-43
KCNV1	1.3256153	-2.79105	0.0007425	0.00103686
MGAM	1.3247134	2.287361	9.17E-08	1.64E-07
EFNA4	1.3240346	4.628484	3.22E-35	2.00E-34
EZR	1.3239917	8.702969	4.03E-68	6.14E-67

Gene names	logFC	log2CPM	P.Value	adj.P.Val
PAGR1	1.3231128	3.473529	4.35E-48	4.01E-47
ADAM12	1.3226481	6.739978	1.05E-22	3.91E-22
FAM81B	1.3226164	-0.1653	6.00E-08	1.09E-07
FAM133A	1.3218859	-0.47933	1.35E-07	2.40E-07
CD1B	1.3218434	-0.90736	4.29E-08	7.84E-08
OVOL1	1.3212822	3.482535	6.96E-27	3.10E-26
CLDN7	1.3204267	6.776467	1.19E-39	8.54E-39
ABAT	1.32	6.11147	8.28E-16	2.26E-15
OIT3	1.3198909	-1.59458	1.11E-14	2.87E-14
FAM129C	1.3196677	0.623008	6.77E-10	1.37E-09
PIK3R2	1.3196189	-0.17389	1.40E-40	1.04E-39
PKP3	1.3175729	6.198576	4.35E-40	3.17E-39
ANKRD18B	1.3171185	-1.80602	2.31E-05	3.56E-05
SP140	1.3170625	2.271494	4.57E-20	1.51E-19
FNDC1	1.3153939	6.310205	3.19E-17	9.30E-17
CFAP221	1.3150595	0.41022	5.01E-11	1.07E-10
NKD2	1.3145586	2.989224	3.74E-21	1.30E-20
GALNT7	1.314138	6.944903	2.10E-25	8.78E-25
PCDHA12	1.3141188	0.350125	6.21E-08	1.12E-07
IL12RB2	1.313854	0.494547	4.42E-08	8.08E-08
MAZ	1.3129138	6.232258	2.28E-88	4.91E-87
KCNK10	1.3128012	-2.16154	3.61E-09	7.00E-09
HSD17B7	1.3127894	3.926531	8.64E-56	9.74E-55
TRPM2	1.3125641	4.357134	3.13E-32	1.74E-31
FKBP10	1.3125379	7.416766	1.73E-38	1.19E-37
PPAPDC1B	1.3124004	6.231523	2.15E-25	8.99E-25
SLC34A1	1.3111166	-3.38624	1.79E-09	3.53E-09
BLK	1.310412	0.690705	1.06E-07	1.89E-07

Gene names	logFC	log2CPM	P.Value	adj.P.Val
IER3	1.3097981	6.815064	3.91E-23	1.48E-22
SLC12A1	1.3094739	1.06919	1.71E-06	2.85E-06
TTYH3	1.3094604	6.793068	1.58E-47	1.44E-46
TBC1D3B	1.3093324	-2.35815	1.18E-09	2.36E-09
TMEM45A	1.3093309	5.234112	1.29E-17	3.84E-17
SOWAHD	1.3085632	-0.00696	9.36E-30	4.70E-29
CENPO	1.3084806	3.820067	7.60E-61	9.75E-60
MCOLN2	1.3065199	2.05627	5.19E-25	2.13E-24
COA6	1.306472	4.562888	8.06E-61	1.03E-59
AK8	1.3062337	2.393946	1.55E-28	7.42E-28
PTCRA	1.3061593	-1.99394	4.18E-14	1.05E-13
PPP1R35	1.3060802	3.997867	1.81E-40	1.34E-39
ATP2C2	1.3060212	4.322861	7.16E-20	2.34E-19
KCNC2	1.3059762	3.235383	0.0035002	0.00465479
OAZ3	1.3052182	2.697674	5.46E-35	3.36E-34
ABCA12	1.3049766	4.762149	9.40E-08	1.68E-07
SKAP1	1.304715	4.043778	1.23E-18	3.79E-18
P2RY10	1.3045951	1.483835	2.66E-13	6.39E-13
FBXL19	1.3040998	4.971388	3.06E-66	4.48E-65
ARL9	1.304048	1.05165	1.50E-11	3.29E-11
MTFP1	1.3035221	1.781021	1.30E-35	8.14E-35
TNFRSF4	1.3032007	1.959194	2.89E-24	1.15E-23
ENSG00000187695	1.30307	0.442891	3.77E-17	1.10E-16
ENPP5	1.3029142	5.881362	2.64E-15	7.05E-15
SLC52A1	1.3027451	1.120044	1.08E-12	2.53E-12
HTR6	1.3024563	-2.61103	9.63E-08	1.72E-07
LRP1B	1.301327	3.853968	5.99E-05	9.01E-05
MAGED2	1.3011502	9.605871	2.96E-29	1.46E-28
SNRPB	1.3009397	7.451113	5.86E-61	7.53E-60

Gene names	logFC	log2CPM	P.Value	adj.P.Val
RABEP2	1.2993281	5.190248	1.99E-42	1.56E-41
HYAL3	1.2992102	2.396588	3.35E-40	2.45E-39
CES5A	1.2991942	-3.06835	0.0003639	0.00051993
GNG8	1.298817	-3.01833	1.93E-12	4.44E-12
SLC6A11	1.298597	1.896459	1.04E-06	1.74E-06
DCTPP1	1.2983377	5.776403	3.25E-67	4.84E-66
ALYREF	1.2983076	5.951136	9.45E-44	7.71E-43
SSX2IP	1.2978105	5.077149	7.02E-65	9.99E-64
OSCAR	1.2976897	2.438418	1.10E-31	6.00E-31
WDHD1	1.2976542	4.124309	2.34E-52	2.41E-51
CTB_102L5.4	1.2974997	-0.49439	2.45E-10	5.06E-10
FBXO16	1.2972543	2.111147	1.13E-26	4.99E-26
FAM171A2	1.2945763	1.97701	1.41E-18	4.34E-18
CDCA4	1.2944628	4.396375	8.31E-60	1.04E-58
NR2F6	1.2938587	6.109713	3.76E-59	4.62E-58
BCL2L12	1.2935881	4.042986	6.60E-43	5.26E-42
GHRHR	1.2934352	-2.58551	5.79E-05	8.71E-05
TSEN54	1.2930199	5.332338	5.21E-61	6.72E-60
SLC22A18	1.2917061	4.695926	1.63E-30	8.47E-30
LPCAT1	1.2915982	6.687252	1.57E-52	1.62E-51
NFE2L3	1.2901967	5.00587	4.90E-25	2.01E-24
C8G	1.2899361	-0.66308	8.80E-21	3.00E-20
MRPS12	1.2897794	5.01434	2.50E-44	2.08E-43
IQCJ	1.2891479	-1.97298	0.000187	0.00027235
FTCD	1.2890407	-0.66835	3.10E-09	6.04E-09
NTHL1	1.2885472	4.249427	9.14E-32	4.98E-31
DNAJC5B	1.2883835	0.303376	2.05E-14	5.22E-14
KRT36	1.2877631	-3.0343	1.60E-12	3.70E-12

Gene names	logFC	log2CPM	P.Value	adj.P.Val
RAB39B	1.2875964	1.894011	2.76E-12	6.29E-12
C8orf46	1.2868571	2.233584	1.43E-08	2.68E-08
B4GALNT1	1.2865032	2.597165	1.77E-15	4.75E-15
PAK4	1.2858815	6.673413	5.40E-57	6.27E-56
PSME2	1.2856796	7.145246	9.02E-49	8.47E-48
BRICD5	1.2854136	1.592693	1.90E-24	7.58E-24
FLAD1	1.2853713	6.035128	3.77E-69	5.89E-68
UQCC3	1.2843571	4.541619	1.65E-32	9.26E-32
DCAF13	1.2831677	6.187543	5.09E-52	5.20E-51
SLC7A2	1.2827412	7.983888	1.35E-08	2.54E-08
KRTAP5_1	1.2816708	-1.39929	4.56E-17	1.32E-16
UCP1	1.2815024	0.657823	0.0005984	0.0008419
GPSM2	1.2811771	4.712681	8.63E-39	6.02E-38
TMC4	1.2811106	6.791254	1.74E-26	7.62E-26
FOXI3	1.2808876	-0.24993	3.76E-05	5.73E-05
RP11_1035H13.3	1.2808873	-2.27845	1.71E-23	6.56E-23
CNFN	1.2808707	2.278974	1.04E-18	3.22E-18
FCRL1	1.2803228	0.096869	1.42E-05	2.23E-05
CABLES2	1.2799761	4.282489	4.57E-51	4.57E-50
TMEM141	1.2799072	5.9213	8.75E-43	6.95E-42
FDXR	1.279443	4.176922	2.94E-30	1.51E-29
BAIAP2L2	1.2793982	1.236668	1.26E-13	3.09E-13
BATF	1.2792707	3.794494	1.02E-21	3.64E-21
CTLA4	1.2789498	1.236405	3.54E-13	8.48E-13
TRPS1	1.2782178	9.042435	3.97E-27	1.78E-26
STARD3	1.2779973	6.393479	4.54E-19	1.43E-18
TCF19	1.2778572	5.087875	1.41E-36	9.09E-36
ST6GAL2	1.2778067	3.602608	1.78E-17	5.24E-17
STRA6	1.2767274	3.309833	3.54E-22	1.28E-21

Gene names	logFC	log2CPM	P.Value	adj.P.Val
HOMER3	1.2766859	5.068817	7.61E-30	3.83E-29
UGCG	1.2765004	7.355241	4.54E-22	1.64E-21
TUBAL3	1.2756362	0.642466	9.35E-13	2.19E-12
RFC4	1.2755265	4.587175	4.85E-47	4.36E-46
CEL	1.2754253	2.441209	4.63E-06	7.48E-06
RDH12	1.2747094	-0.9913	7.58E-15	1.97E-14
FATE1	1.2746507	-1.66694	3.68E-07	6.37E-07
AGXT2	1.274623	-1.82148	2.17E-09	4.27E-09
TOMM40	1.2737929	6.041962	3.33E-54	3.60E-53
MMP8	1.2736444	-1.13324	8.08E-06	1.29E-05
ASCL5	1.2730677	-2.70384	4.37E-13	1.04E-12
CYP4F8	1.2729451	4.046636	0.0006106	0.00085875
AP1S1	1.2727634	5.803261	9.03E-71	1.46E-69
RARA	1.2725906	6.955908	1.91E-28	9.09E-28
CACNA2D2	1.2723364	4.445717	5.51E-14	1.37E-13
PIGQ	1.27219	6.345512	1.84E-45	1.59E-44
ZBTB32	1.2717296	-0.1392	6.28E-19	1.96E-18
ZNF726	1.2714503	2.239727	3.28E-24	1.30E-23
BCL9	1.2713006	6.075615	9.90E-65	1.40E-63
RABIF	1.270573	4.665365	1.37E-94	3.26E-93
CCDC24	1.270322	4.276617	5.24E-34	3.11E-33
TEX11	1.2699847	-1.45207	8.14E-16	2.22E-15
C16orf13	1.2690428	5.59611	1.28E-40	9.50E-40
NDUFAF6	1.2682432	4.997277	9.40E-45	7.90E-44
ACTL10	1.2663314	1.609312	3.36E-25	1.39E-24
CR2	1.2660123	2.261602	5.88E-05	8.84E-05
ATP6AP1	1.2655061	8.245005	8.69E-70	1.38E-68
OLFML2B	1.2652968	6.187178	2.10E-29	1.04E-28

Gene names	logFC	log2CPM	P.Value	adj.P.Val
CLEC4C	1.265181	-2.15975	3.16E-06	5.15E-06
SLC37A1	1.2650262	5.263185	3.88E-64	5.44E-63
FAM222A	1.2649961	3.421155	2.72E-25	1.13E-24
GFRA1	1.2649703	8.541221	6.82E-08	1.23E-07
TBX1	1.2646387	3.255186	6.75E-12	1.51E-11
HDGF	1.2646071	8.763018	2.82E-73	4.74E-72
C15orf59	1.2641003	2.591446	3.06E-11	6.62E-11
IKBKE	1.2635626	4.283758	3.40E-44	2.82E-43
ACSL6	1.2628082	1.600131	9.89E-11	2.08E-10
DIRC1	1.2624234	-2.18138	3.65E-13	8.72E-13
LHX8	1.2615125	-1.39957	0.0004376	0.00062105
MRPL13	1.2611425	5.648885	6.17E-48	5.66E-47
PUSL1	1.2610225	3.709039	4.75E-43	3.80E-42
B4GALT3	1.2594952	6.340271	6.68E-69	1.04E-67
PLEKHG7	1.2581554	-1.37856	1.21E-06	2.03E-06
GREB1L	1.2580506	4.244711	1.71E-12	3.94E-12
SAMD10	1.256971	3.642341	2.58E-45	2.21E-44
LINGO3	1.2566916	-1.01937	1.63E-15	4.39E-15
TMEM105	1.256497	0.212533	8.56E-11	1.81E-10
CD5	1.2564139	2.997552	3.36E-15	8.89E-15
ZNF239	1.2560867	3.763791	9.12E-44	7.46E-43
RHOF	1.254433	0.607566	5.53E-30	2.81E-29
NREP	1.2542722	6.208186	7.35E-45	6.20E-44
INTS7	1.2540716	5.626091	6.90E-78	1.26E-76
CHRNA5	1.2531117	1.254279	5.74E-13	1.36E-12
TCTEX1D2	1.2527519	2.536457	9.20E-48	8.42E-47
FAM159A	1.2526623	-0.33014	4.02E-21	1.39E-20
SDSL	1.2526545	4.087567	2.94E-35	1.83E-34
CCT3	1.2519397	8.810946	7.24E-81	1.39E-79

Gene names	logFC	log2CPM	P.Value	adj.P.Val
C1orf35	1.2516472	4.650133	3.01E-46	2.64E-45
TDRD12	1.2510486	2.246405	1.48E-05	2.31E-05
ATP1A4	1.2503863	2.327552	6.77E-08	1.22E-07
IDH2	1.2497003	7.861712	3.79E-34	2.26E-33
DBF4	1.2493726	3.986494	6.84E-54	7.32E-53
SHROOM2	1.2486068	4.452133	1.13E-33	6.60E-33
PAX5	1.2483536	1.690621	3.25E-06	5.29E-06
PRLR	1.2481472	8.016393	6.43E-21	2.20E-20
FAM19A5	1.2479115	2.556026	9.80E-14	2.41E-13
CERS1	1.2465341	-0.27412	1.84E-09	3.63E-09
MT1G	1.2454067	3.308537	1.26E-08	2.37E-08
TBC1D31	1.2449775	3.599815	1.67E-40	1.24E-39
RAB11FIP4	1.2441241	5.746142	5.92E-37	3.88E-36
FCGR3A	1.2441183	6.267893	5.07E-27	2.27E-26
NEK5	1.2438135	2.779159	4.91E-19	1.55E-18
COLEC10	1.2436383	-2.15784	2.12E-16	5.96E-16
SEPHS2	1.24337	7.225238	1.94E-51	1.96E-50
PAEP	1.2432601	-0.36133	6.06E-05	9.10E-05
REEP1	1.2427856	4.447865	2.46E-14	6.24E-14
EMC9	1.2426802	3.770747	8.17E-42	6.30E-41
FLYWCH2	1.2415587	4.986121	5.66E-44	4.66E-43
KCNJ11	1.2408632	4.130791	1.73E-16	4.88E-16
GALNT14	1.2403484	3.207272	8.43E-12	1.87E-11
H2AFJ	1.2395583	7.208673	6.73E-18	2.03E-17
WDR90	1.2388204	5.375069	4.92E-39	3.46E-38
DHRS13	1.2384944	2.641479	5.57E-31	2.96E-30
IL18	1.2382691	3.424056	4.33E-26	1.86E-25
DENND1B	1.2378304	5.903952	1.37E-20	4.62E-20

Gene names	logFC	log2CPM	P.Value	adj.P.Val
RSPO4	1.2376079	0.376447	3.57E-07	6.18E-07
TFPT	1.2374629	4.321844	1.28E-31	6.93E-31
UQCC2	1.2367998	5.291961	2.32E-50	2.27E-49
CISD3	1.2363912	5.455038	1.48E-26	6.51E-26
REEP6	1.2361481	6.14901	1.50E-12	3.47E-12
BASP1	1.2359169	5.402233	2.69E-28	1.27E-27
ADAMTS6	1.2356373	1.962249	1.68E-25	7.05E-25
GRHL3	1.235551	2.223624	1.96E-15	5.26E-15
PARP1	1.2350603	8.193956	5.19E-84	1.05E-82
RNF208	1.2349041	3.158926	9.52E-27	4.22E-26
TCHH	1.2344415	-0.09464	1.92E-12	4.41E-12
TFF2	1.2342501	-1.18438	0.0002416	0.00034934
ARX	1.2341616	0.298755	3.72E-05	5.67E-05
SREBF1	1.2338489	8.384647	2.93E-25	1.22E-24
STAT1	1.2334776	8.588168	7.02E-28	3.26E-27
PFDN6	1.2330905	5.238101	1.77E-48	1.65E-47
ZDHHC12	1.2326486	5.201684	5.15E-49	4.87E-48
PGC	1.2320403	1.437751	0.0009316	0.00129377
PLEKHN1	1.2317978	2.342739	4.77E-26	2.05E-25
FOXD4L4	1.2314532	-3.46807	1.99E-07	3.50E-07
DHCR7	1.2306933	6.619224	2.86E-24	1.13E-23
FAIM2	1.2305384	3.495958	4.71E-08	8.59E-08
TIMM17B	1.2299732	5.375776	1.10E-51	1.12E-50
HSF2BP	1.2299431	0.408243	3.19E-42	2.49E-41
PFDN2	1.2298472	5.631731	3.49E-36	2.23E-35
PRR4	1.2296623	2.229665	7.93E-07	1.34E-06
C19orf48	1.2295711	5.861755	2.68E-40	1.97E-39
HSD11B2	1.2289717	3.829084	1.71E-19	5.49E-19
DOPEY2	1.2286333	6.408365	3.90E-46	3.42E-45

Gene names	logFC	log2CPM	P.Value	adj.P.Val
TRAF2	1.2282884	4.986233	1.36E-62	1.83E-61
NPAS1	1.2281867	0.828535	6.31E-17	1.81E-16
TREM1	1.2278464	1.7929	5.54E-14	1.38E-13
PZP	1.2269959	0.866099	1.85E-05	2.88E-05
SYNGR2	1.2267608	8.138513	3.95E-51	3.95E-50
CAGE1	1.2267452	-2.29019	2.49E-08	4.62E-08
FCRLA	1.2263532	1.007648	3.47E-08	6.37E-08
TBC1D3E	1.2256487	-3.13752	0.0001346	0.00019814
ACY3	1.2255391	1.339524	3.97E-06	6.43E-06
GDAP1	1.2255113	5.052685	1.97E-12	4.51E-12
CA2	1.2254474	6.364743	1.77E-07	3.12E-07
CDKN2D	1.2246398	3.475339	1.70E-37	1.14E-36
FAM71E1	1.2243281	1.887139	3.79E-23	1.43E-22
NCAPG2	1.2239307	5.191339	2.17E-45	1.87E-44
ZP2	1.2235151	-0.31844	0.0069277	0.00900991
RMI1	1.2231155	4.417524	1.03E-58	1.25E-57
RHEBL1	1.2230972	0.747896	6.04E-33	3.44E-32
PPP1R37	1.2229686	6.036592	2.03E-50	2.00E-49
PPDPF	1.2229526	8.240818	2.24E-34	1.35E-33
EPGN	1.2222696	-1.26288	2.44E-06	4.01E-06
PCDHA5	1.2213818	-0.54737	6.07E-09	1.16E-08
NANS	1.2212162	6.23591	8.62E-60	1.07E-58
ENTPD7	1.2200443	4.156732	1.14E-50	1.13E-49
KIF12	1.2189754	5.515396	2.27E-10	4.70E-10
S100A11	1.2189184	9.036129	2.50E-32	1.40E-31
KNSTRN	1.2187739	4.26934	5.86E-48	5.39E-47
ROMO1	1.2187631	5.552061	2.62E-27	1.19E-26
NOL4	1.2186176	0.164697	8.51E-05	0.00012681

Gene names	logFC	log2CPM	P.Value	adj.P.Val
KCNK5	1.2176424	4.156741	2.89E-09	5.64E-09
ARHGEF16	1.2175172	4.686614	7.17E-37	4.69E-36
PSMG3	1.2169987	5.023653	1.07E-44	8.94E-44
PARP10	1.2168368	6.520434	1.19E-27	5.47E-27
CHST11	1.2168254	5.011214	5.73E-40	4.16E-39
E2F5	1.2163533	4.030311	5.03E-37	3.30E-36
GFER	1.2159772	4.554313	1.01E-47	9.22E-47
PDIA4	1.2155966	8.041735	1.01E-60	1.29E-59
PPEF1	1.2155137	1.708953	4.89E-15	1.28E-14
PRR22	1.2141576	1.192515	4.98E-21	1.72E-20
S100A16	1.2113466	7.894416	1.01E-23	3.91E-23
FANCB	1.2107739	0.931784	1.95E-28	9.29E-28
FAM131C	1.2107416	-0.91054	4.58E-08	8.36E-08
EMID1	1.2107132	3.594575	1.08E-13	2.64E-13
VWA3A	1.2105928	-0.07515	1.24E-08	2.33E-08
TPBGL	1.210531	-0.12975	4.65E-21	1.61E-20
C2orf91	1.2104139	-2.5295	1.34E-10	2.81E-10
C1QL4	1.2103325	-0.24074	1.02E-05	1.61E-05
ANAPC11	1.2095316	6.127181	5.78E-36	3.67E-35
RALGPS2	1.2089465	6.320814	8.99E-24	3.49E-23
CPN2	1.2066119	-1.04047	1.66E-09	3.29E-09
SUMO4	1.2064539	-2.39715	2.99E-12	6.80E-12
ST20_MTHFS	1.206379	-3.2206	1.02E-12	2.39E-12
TPSD1	1.2050127	1.662584	1.98E-05	3.08E-05
SULT1E1	1.2047846	-0.32595	0.00204	0.00276452
TMC5	1.2045029	5.861982	5.20E-09	1.00E-08
TOR2A	1.2044931	4.175193	3.22E-55	3.58E-54
ADAT3	1.2042603	1.042177	4.32E-22	1.56E-21
OR13A1	1.2042167	-2.82123	2.32E-07	4.06E-07

Gene names	logFC	log2CPM	P.Value	adj.P.Val
ISG20	1.2035245	4.481702	3.21E-24	1.27E-23
SAMD15	1.2033931	1.931601	4.83E-20	1.59E-19
SLC25A39	1.2031436	7.287009	3.80E-55	4.21E-54
SIGIRR	1.2029389	5.559386	4.86E-32	2.67E-31
C19orf40	1.2029173	2.718112	1.06E-71	1.75E-70
JAKMIP2	1.2024292	0.34021	4.58E-15	1.20E-14
ARL11	1.2022796	2.194175	1.23E-23	4.74E-23
NFKBIE	1.2018552	4.437186	2.03E-41	1.55E-40
CPNE9	1.2017056	-1.84387	1.74E-15	4.67E-15
RCC1	1.2015585	5.870929	2.54E-51	2.56E-50
PSMB3	1.2010703	6.971525	1.47E-31	7.96E-31
NMRAL1	1.200359	5.848171	2.36E-53	2.50E-52
MLLT11	1.1998448	3.80463	1.07E-27	4.94E-27
TSGA10IP	1.1996961	-2.84094	5.12E-09	9.85E-09
C11orf16	1.1995539	-0.83888	3.13E-08	5.76E-08
CDC25B	1.1983633	6.033572	5.34E-27	2.39E-26
MCCD1	1.1982526	-0.53056	0.0022015	0.00297524
PCGF2	1.1981093	6.026773	3.68E-30	1.88E-29
MS4A14	1.1980028	2.80614	3.88E-11	8.35E-11
HELZ2	1.197497	6.09298	6.86E-31	3.62E-30
BARD1	1.1973836	4.230428	2.91E-49	2.77E-48
BAX	1.1958118	5.421073	2.10E-55	2.35E-54
ARRDC1	1.1956855	5.965692	4.99E-47	4.48E-46
FOXS1	1.193949	1.703616	3.31E-26	1.43E-25
XKRX	1.1939329	-0.32335	1.59E-19	5.11E-19
TMEM125	1.1935678	4.695234	1.06E-31	5.79E-31
SLC25A10	1.1933844	4.621929	2.91E-29	1.43E-28
KDM4B	1.1914545	7.236931	4.66E-29	2.28E-28

Gene names	logFC	log2CPM	P.Value	adj.P.Val
TMPRSS3	1.1910912	5.483086	1.39E-08	2.61E-08
C6orf1	1.190582	4.838784	2.84E-45	2.43E-44
RHOD	1.1897491	4.923696	3.54E-31	1.89E-30
HSPE1	1.1892144	6.570914	2.67E-50	2.61E-49
SCAND1	1.188814	5.438538	2.24E-25	9.35E-25
OXLD1	1.1880671	4.464644	3.46E-34	2.07E-33
ZNF707	1.1878469	4.152998	7.48E-45	6.31E-44
ZNHIT2	1.1877938	3.683765	1.11E-28	5.34E-28
FBP1	1.1875776	6.411327	7.46E-18	2.24E-17
SUSD2	1.1871168	5.903224	5.90E-12	1.32E-11
PLEKHF2	1.1868072	6.588308	3.04E-30	1.56E-29
PHKG2	1.1866935	5.251055	8.07E-61	1.03E-59
SLC3A1	1.1863819	-0.52768	1.04E-06	1.75E-06
MOXD1	1.1862646	3.693087	4.05E-16	1.12E-15
EPHX4	1.1861228	1.787465	1.95E-10	4.06E-10
C1QL1	1.1861124	0.677421	2.73E-09	5.33E-09
PAQR5	1.1860176	3.484552	3.15E-17	9.18E-17
SLC35F2	1.1856829	4.366714	3.82E-25	1.58E-24
SNRPE	1.1855731	6.203557	7.80E-59	9.49E-58
SLC10A4	1.1855489	-2.07692	1.03E-11	2.27E-11
SLC7A8	1.1852204	7.213034	2.70E-18	8.24E-18
LRGUK	1.1847144	0.726025	1.03E-16	2.95E-16
LRRC71	1.1846536	-1.41289	8.59E-09	1.63E-08
SMCO3	1.1845996	1.107193	5.07E-06	8.17E-06
RRS1	1.18459	5.066598	1.33E-33	7.80E-33
CHD5	1.1837684	-0.34104	9.58E-08	1.71E-07
MAGIX	1.1836846	2.397845	2.23E-27	1.01E-26
SAMD1	1.1832291	5.75157	5.96E-60	7.47E-59
AEBP1	1.1825877	9.531545	1.29E-22	4.75E-22

Gene names	logFC	log2CPM	P.Value	adj.P.Val
PSENEN	1.1824151	5.98615	7.14E-54	7.63E-53
TDRKH	1.1822335	4.822563	3.10E-48	2.87E-47
LRWD1	1.1820396	4.337066	2.83E-60	3.58E-59
TBC1D30	1.1817532	4.371391	2.42E-29	1.20E-28
IFITM1	1.1815221	7.651725	9.38E-18	2.81E-17
EBP	1.181099	5.470329	1.90E-40	1.41E-39
TREX2	1.1810489	-0.28418	7.31E-16	2.00E-15
LPAR2	1.179763	4.490219	2.38E-45	2.04E-44
NRCAM	1.1795518	3.910937	1.12E-09	2.23E-09
ABCC5	1.1792331	6.376639	3.77E-26	1.63E-25
SEMA3F	1.1786823	6.754301	3.83E-37	2.53E-36
RNASE2	1.1786667	-0.40085	6.51E-17	1.87E-16
SPATA12	1.1783311	-1.17197	8.10E-15	2.10E-14
HGD	1.1774108	3.457042	2.20E-07	3.85E-07
SYTL5	1.1774052	3.77868	3.06E-07	5.31E-07
APOO	1.1771995	4.140225	8.88E-51	8.81E-50
PDRG1	1.1768839	5.024509	1.83E-48	1.70E-47
YIF1B	1.1759911	5.259302	3.65E-49	3.47E-48
IFITM10	1.1752653	4.283393	6.97E-13	1.64E-12
THBS2	1.1735863	8.566285	3.63E-19	1.15E-18
CCL19	1.1729799	4.340513	4.35E-07	7.50E-07
EOMES	1.1729798	1.411636	4.27E-11	9.16E-11
FBXO41	1.1723936	4.202042	5.23E-38	3.56E-37
DUSP15	1.172345	1.958068	5.99E-11	1.28E-10
RAB11FIP1	1.1719375	7.161628	2.15E-13	5.19E-13
MRPL55	1.170841	5.3068	5.73E-37	3.76E-36
ATP8B3	1.1708222	1.708752	3.69E-18	1.12E-17
SLC20A1	1.1707254	6.343127	1.47E-90	3.28E-89

Gene names	logFC	log2CPM	P.Value	adj.P.Val
C1orf53	1.1703488	0.921482	1.25E-23	4.82E-23
TIMP1	1.1689507	8.352646	7.09E-29	3.43E-28
CTNND2	1.1679988	4.069807	1.72E-09	3.41E-09
PLXNA3	1.1677656	6.191505	1.06E-55	1.19E-54
HMGB2	1.1673955	7.029069	4.66E-36	2.96E-35
TCEB2	1.1671778	7.231271	9.59E-37	6.24E-36
TTC24	1.1667341	-1.85899	1.19E-07	2.13E-07
PLA2G2C	1.1661255	-1.89776	1.11E-08	2.09E-08
LY6G6C	1.1660308	1.140032	1.27E-06	2.12E-06
F5	1.165879	1.922817	6.13E-11	1.30E-10
FAM19A4	1.1652931	-2.53981	5.73E-05	8.63E-05
MFSD6L	1.1648744	1.733283	3.50E-09	6.79E-09
SLC5A2	1.1640881	-1.69155	2.87E-14	7.27E-14
ALG3	1.1639007	5.774666	2.94E-51	2.95E-50
TMEM9	1.1636134	7.246183	4.93E-56	5.59E-55
CIB4	1.1631891	-3.25518	5.13E-05	7.74E-05
SFXN2	1.1631713	5.105006	3.07E-25	1.27E-24
CXCR4	1.1631246	6.523517	2.07E-25	8.67E-25
GPC4	1.1630408	6.027757	4.82E-18	1.46E-17
CD6	1.162148	3.400892	1.95E-15	5.23E-15
AMBP	1.1617467	1.23405	4.49E-07	7.72E-07
C20orf195	1.1617056	0.064643	4.19E-15	1.10E-14
VAX2	1.1614659	1.255513	5.97E-12	1.34E-11
PPP1CA	1.1611859	7.481564	3.04E-56	3.47E-55
DSN1	1.1610172	4.86302	1.93E-53	2.04E-52
ASPSCR1	1.1609792	5.106774	2.58E-41	1.97E-40
APOC4_APOC2	1.1608801	-2.9825	1.03E-13	2.53E-13
STAP1	1.1599721	0.341967	4.35E-12	9.82E-12
AGMAT	1.1595745	1.412873	8.06E-27	3.59E-26

Gene names	logFC	log2CPM	P.Value	adj.P.Val
IKZF3	1.1590836	4.36573	7.96E-12	1.77E-11
ERMP1	1.1588783	6.937182	4.54E-37	2.99E-36
ABHD12	1.1588717	6.50068	6.99E-50	6.73E-49
HLA_DQB2	1.1588417	4.22046	2.55E-13	6.15E-13
PASK	1.1587712	4.238485	3.16E-57	3.69E-56
PVRL4	1.1583583	6.439411	5.49E-26	2.36E-25
MTBP	1.1576964	3.245689	4.73E-29	2.31E-28
ZWILCH	1.1573695	4.477525	1.20E-63	1.66E-62
BNIPL	1.1573186	5.161464	4.27E-13	1.02E-12
PAK1	1.1568749	6.747132	1.58E-20	5.33E-20
TIMM17A	1.1562969	6.315901	7.33E-75	1.27E-73
P2RY4	1.1562659	-1.77813	2.43E-06	3.99E-06
USP18	1.1562066	3.748479	3.95E-24	1.56E-23
ZNF804A	1.1556095	-0.53938	2.60E-13	6.25E-13
STRBP	1.1552899	6.092126	2.26E-77	4.09E-76
GSG1	1.1543006	-1.85729	2.32E-08	4.31E-08
CD3EAP	1.1542168	3.058729	2.99E-57	3.50E-56
ATP6V0D2	1.1537344	0.893377	2.45E-09	4.81E-09
SYT7	1.1535083	6.793964	1.08E-19	3.50E-19
BAIAP3	1.1533791	4.382598	1.57E-10	3.28E-10
CAMSAP3	1.1525804	5.423016	2.28E-29	1.13E-28
CCDC137	1.1525668	4.784432	1.69E-41	1.29E-40
BRSK1	1.1522773	2.836726	9.26E-20	3.01E-19
MANF	1.151537	6.054478	3.78E-59	4.65E-58
GK	1.1510307	3.228349	1.11E-40	8.30E-40
KCNQ3	1.1496987	3.058381	1.24E-14	3.20E-14
ETNPPL	1.1493737	-0.83622	4.06E-05	6.17E-05
JTB	1.1484291	7.46455	6.71E-66	9.78E-65

Gene names	logFC	log2CPM	P.Value	adj.P.Val
CCDC42B	1.1482837	-0.95289	1.34E-10	2.80E-10
DEPDC7	1.1480144	0.930467	2.80E-21	9.76E-21
CAPG	1.147888	7.595886	2.47E-34	1.49E-33
NTSR2	1.1477645	-1.95367	0.0025226	0.00339148
SMS	1.1477498	6.9314	1.88E-55	2.10E-54
TUBG1	1.1477389	5.598405	1.28E-50	1.27E-49
RGR	1.1463866	-1.20878	0.0024869	0.00334612
C11orf98	1.1455111	1.814005	2.88E-34	1.73E-33
PNOC	1.1452181	-0.31026	5.37E-09	1.03E-08
BAIAP2L1	1.1449645	5.441065	2.72E-41	2.07E-40
ENSG00000273154	1.1443796	-0.0972	1.08E-20	3.65E-20
BATF2	1.1443385	2.997648	6.86E-12	1.53E-11
ZBTB12	1.1433781	3.256848	1.51E-39	1.08E-38
FAM153C	1.1423677	-2.25711	6.25E-11	1.33E-10
HPRT1	1.1420549	5.329926	5.34E-51	5.32E-50
GCAT	1.1418855	4.129167	1.20E-26	5.27E-26
CFAP57	1.1412962	1.513096	2.88E-13	6.92E-13
CCR3	1.1412672	-1.71359	4.06E-12	9.17E-12
FAM102A	1.1409138	7.685065	1.08E-48	1.01E-47
ARFGAP1	1.1408917	6.60661	3.57E-50	3.47E-49
MXRA5	1.1408526	8.550965	2.85E-22	1.04E-21
BMP8B	1.1405233	3.146994	1.00E-15	2.73E-15
C11orf85	1.1388997	-1.93952	1.20E-05	1.89E-05
FADD	1.1384082	3.921424	5.30E-29	2.58E-28
TCEAL3	1.1369419	5.688046	1.52E-20	5.11E-20
THOC6	1.1368763	5.060174	9.26E-47	8.20E-46
BRCA1	1.1363281	4.409685	1.02E-28	4.89E-28
KCTD13	1.1362757	4.536236	5.65E-54	6.06E-53
LY6E	1.135991	8.619333	3.09E-16	8.61E-16

Gene names	logFC	log2CPM	P.Value	adj.P.Val
KDELR3	1.1358832	5.41244	1.72E-24	6.90E-24
GTF2IRD1	1.1357034	5.296405	8.65E-82	1.68E-80
KCNS3	1.1355361	5.178806	1.24E-20	4.19E-20
NUDT1	1.1354418	4.000551	2.25E-31	1.21E-30
BRI3BP	1.1345027	5.304702	1.28E-49	1.23E-48
DRAP1	1.1344718	6.271201	3.75E-32	2.08E-31
TMEM52B	1.1340511	0.400149	1.12E-23	4.32E-23
DTYMK	1.1340011	4.839224	5.21E-53	5.44E-52
TRAT1	1.133824	0.786648	4.25E-09	8.22E-09
CRYBA4	1.133673	-3.20726	1.20E-08	2.26E-08
AGRP	1.1334754	-3.06221	2.24E-15	5.98E-15
USP35	1.1330606	3.992955	4.79E-19	1.51E-18
GPR132	1.1325711	2.801129	1.23E-22	4.55E-22
SFXN1	1.1322084	5.596604	5.25E-47	4.71E-46
GRTP1	1.1320225	4.364555	2.79E-38	1.92E-37
MYO1G	1.1319614	3.371328	2.02E-21	7.09E-21
CHRM4	1.1318541	-1.05706	5.09E-09	9.80E-09
CIB1	1.1304935	6.947883	8.87E-39	6.18E-38
SAP30	1.1304256	3.665509	8.06E-37	5.26E-36
ACOT6	1.1297785	-2.43247	8.15E-07	1.38E-06
TAF7L	1.1291955	-1.35063	6.48E-12	1.45E-11
RHOH	1.1289904	4.725976	5.85E-13	1.38E-12
MICALL2	1.1285642	4.958293	2.07E-31	1.11E-30
SULF2	1.1275883	8.081977	7.27E-23	2.72E-22
MAP6D1	1.1269862	2.416568	6.82E-21	2.34E-20
CISH	1.1267164	5.870418	1.14E-23	4.39E-23
TP53INP1	1.126589	6.857453	2.89E-27	1.31E-26
KNTC1	1.1262907	5.051206	1.37E-43	1.12E-42

Gene names	logFC	log2CPM	P.Value	adj.P.Val
PSPH	1.126223	4.254201	3.37E-30	1.73E-29
TXNDC2	1.1259456	-2.85155	2.43E-15	6.48E-15
MIS18A	1.1253857	4.147226	2.02E-54	2.20E-53
DAND5	1.1247062	-1.42698	2.84E-12	6.46E-12
KRTAP5_8	1.1244796	-3.09852	6.51E-09	1.25E-08
CHAF1A	1.124084	4.733842	4.37E-45	3.71E-44
CRISP2	1.1240456	-0.93075	0.0035958	0.00477499
SCUBE2	1.1229101	8.52082	7.93E-07	1.34E-06
HLA_DQA2	1.1229046	4.854646	5.37E-10	1.09E-09
EFNA1	1.1224125	7.098045	1.05E-37	7.03E-37
VAV2	1.1218153	6.021618	9.81E-67	1.45E-65
TFRC	1.1217963	7.364485	5.39E-24	2.11E-23
LYPD3	1.1215197	5.723593	1.69E-15	4.54E-15
H2AFY	1.121402	8.039235	3.09E-111	9.85E-110
MS4A1	1.1212316	3.295061	2.56E-05	3.95E-05
FAM26F	1.1210439	2.763971	2.15E-15	5.76E-15
ENSG00000279765	1.1210292	-0.4401	1.27E-10	2.66E-10
SLX1A_SULT1A3	1.1206893	-3.22398	1.74E-12	4.02E-12
MAGED1	1.1196605	8.244208	1.04E-42	8.25E-42
EDDM3A	1.1195491	-3.0463	0.0005365	0.00075728
MRPL24	1.1194048	6.507389	1.96E-39	1.40E-38
TXNDC17	1.1190772	5.202897	9.63E-51	9.55E-50
TIGD5	1.1190095	4.068807	7.52E-34	4.44E-33
DNAI2	1.1189356	-3.06475	1.41E-06	2.36E-06
TTC9B	1.1187221	-1.96822	2.90E-13	6.97E-13
GRAMD1A	1.1184218	6.601818	3.30E-68	5.05E-67
TTLL9	1.1182142	0.765691	1.23E-11	2.71E-11
APITD1	1.1166548	1.983031	1.47E-47	1.34E-46
CERS6	1.1164599	6.672098	3.71E-25	1.53E-24

Gene names	logFC	log2CPM	P.Value	adj.P.Val
OCIAD2	1.1148623	5.460859	7.02E-49	6.61E-48
KCTD5	1.1147061	5.47275	5.11E-74	8.66E-73
NOX5	1.1140954	1.170055	4.83E-09	9.31E-09
SEC61G	1.1140232	5.90765	1.23E-36	7.99E-36
POTEE	1.1139298	-2.2732	6.32E-08	1.14E-07
CNR2	1.1138403	-0.58279	1.13E-06	1.90E-06
SIPA1L3	1.1137851	6.604085	2.45E-50	2.40E-49
SNX20	1.1136747	3.073588	1.54E-17	4.58E-17
MAPKAPK2	1.1134806	8.037378	4.37E-53	4.59E-52
LRRC61	1.1127439	4.267307	4.17E-41	3.15E-40
WFDC2	1.1125716	6.610845	8.47E-07	1.43E-06
PHLDB3	1.1118741	3.994728	1.08E-29	5.42E-29
SHISA2	1.1114071	5.551735	1.62E-06	2.69E-06
PREX1	1.111251	7.845191	1.19E-15	3.24E-15
POP1	1.1109842	3.800725	3.42E-33	1.97E-32
PCDHA11	1.1102445	1.055898	1.05E-06	1.76E-06
CCDC177	1.1097871	-0.9179	3.10E-07	5.39E-07
HOPX	1.1087743	3.610405	2.37E-20	7.91E-20
WFDC3	1.1087589	-0.08406	2.81E-15	7.49E-15
POP7	1.1085671	4.894052	4.40E-45	3.74E-44
CD7	1.1082089	2.498823	1.64E-11	3.59E-11
SLC22A15	1.1080458	2.811272	3.28E-21	1.14E-20
OTUB2	1.1080163	2.164254	9.91E-37	6.44E-36
EPHX3	1.1080052	0.840468	6.24E-07	1.06E-06
HOXA11	1.1077817	0.227134	4.53E-09	8.75E-09
MFSD7	1.1070485	4.361135	9.10E-15	2.35E-14
COPE	1.1066141	6.969228	6.22E-45	5.26E-44
CALR	1.1064834	9.91912	1.60E-51	1.62E-50

Gene names	logFC	log2CPM	P.Value	adj.P.Val
KRTAP5_7	1.1062123	-3.18597	4.33E-08	7.91E-08
RCN3	1.1062121	5.696511	7.68E-18	2.31E-17
LIG1	1.1059233	5.510867	7.66E-55	8.41E-54
UBE2L6	1.1051533	6.91578	2.08E-29	1.04E-28
IL2RB	1.1047238	4.491467	5.32E-12	1.20E-11
VAMP8	1.1041387	7.059097	2.86E-49	2.72E-48
ARF1	1.1023728	9.462092	2.42E-77	4.37E-76
APOC1	1.1016794	5.826008	2.96E-16	8.25E-16
SNPH	1.1001349	4.037735	5.40E-09	1.04E-08
RBP1	1.0999499	5.622093	2.37E-09	4.64E-09
KNOP1	1.0998857	5.101347	2.59E-74	4.43E-73
HNRNPAB	1.0993059	7.491826	2.80E-71	4.58E-70
LRRC16B	1.0989832	1.203896	7.69E-14	1.90E-13
UPK3B	1.0989732	0.355627	8.89E-10	1.79E-09
HOXC4	1.0987965	3.234169	4.08E-19	1.29E-18
EPSTI1	1.0987386	4.481819	2.98E-20	9.88E-20
CSPG5	1.0987354	1.358054	4.08E-15	1.08E-14
HDAC11	1.0987233	5.610331	1.44E-26	6.32E-26
AC114494.1	1.0985601	0.048212	1.14E-13	2.80E-13
ATP7B	1.0982302	4.783177	8.33E-16	2.27E-15
UNC13D	1.0981957	4.752272	1.69E-15	4.54E-15
SMPDL3B	1.0980392	4.214546	1.46E-20	4.94E-20
POLR2H	1.0980326	5.736942	1.99E-58	2.39E-57
ZNF48	1.0977917	4.406996	1.87E-58	2.25E-57
IGSF8	1.0975652	6.644777	1.30E-37	8.70E-37
NUBP2	1.0969281	5.874837	1.85E-37	1.24E-36
EFCAB12	1.0969032	1.846664	5.34E-12	1.20E-11
PCSK6	1.0968562	5.316645	5.87E-12	1.32E-11
ARPC1B	1.0963834	7.160505	9.19E-35	5.61E-34

Gene names	logFC	log2CPM	P.Value	adj.P.Val
GEN1	1.0962273	4.289308	4.06E-46	3.55E-45
C17orf96	1.0959853	3.305794	1.62E-12	3.74E-12
SLC29A3	1.0957249	4.629892	1.13E-35	7.09E-35
NR0B1	1.0950814	-0.74049	0.016237	0.02044081
DNPH1	1.0941396	4.60918	6.96E-23	2.61E-22
FAM189B	1.0939015	5.673386	4.96E-43	3.97E-42
CORO1A	1.0932898	5.687401	7.02E-18	2.11E-17
CDH19	1.0929245	1.900848	0.0076467	0.00990458
FCGR1B	1.09254	-0.05805	9.75E-23	3.62E-22
CCDC64B	1.092184	4.956584	2.33E-19	7.45E-19
KLC2	1.0918373	5.426542	6.89E-44	5.66E-43
MRGBP	1.0917237	4.573129	5.36E-50	5.18E-49
C2orf70	1.0912904	0.234033	9.68E-08	1.73E-07
SCIN	1.0910621	4.130563	4.43E-08	8.09E-08
RHBDL2	1.090489	2.323869	2.64E-20	8.80E-20
ATAD3A	1.0899471	4.49795	8.97E-29	4.32E-28
RIBC1	1.0897171	2.043441	6.32E-21	2.17E-20
GSDMD	1.0895488	6.331073	5.19E-28	2.43E-27
CENPH	1.0894415	3.408348	1.53E-43	1.24E-42
TCIRG1	1.0894137	6.130568	1.58E-27	7.21E-27
MSI1	1.0891291	2.640373	2.98E-08	5.49E-08
TEAD4	1.0889062	4.145891	1.56E-28	7.44E-28
BUB3	1.0884895	7.266773	4.19E-54	4.52E-53
SUV420H2	1.0878422	3.818875	2.09E-33	1.21E-32
FHDC1	1.0877414	4.633466	5.37E-31	2.85E-30
NME8	1.0873591	-0.77924	3.63E-14	9.13E-14
KRTAP5_9	1.0870892	-1.8087	1.87E-12	4.30E-12
CAPN8	1.0868801	5.334805	7.30E-07	1.24E-06

Gene names	logFC	log2CPM	P.Value	adj.P.Val
RTKN	1.0867408	5.718047	2.40E-51	2.42E-50
TMEM210	1.0867173	-1.0192	2.83E-10	5.82E-10
RCC2	1.0864151	7.840141	1.19E-70	1.92E-69
ENSG00000258830	1.0863293	-2.8542	2.36E-11	5.11E-11
TARS2	1.0862627	5.771003	5.66E-61	7.29E-60
ZNF724P	1.0860138	1.303325	7.60E-19	2.37E-18
COMMD5	1.0857605	5.111284	5.44E-48	5.00E-47
SLC9C1	1.0855722	-0.35909	1.36E-08	2.56E-08
KIAA1875	1.0851867	1.002667	4.55E-11	9.74E-11
RAB40C	1.0840343	5.36517	1.11E-44	9.31E-44
RHBDD3	1.0840026	4.571944	1.15E-38	7.98E-38
ATP5EP2	1.0838445	-1.43905	2.07E-14	5.28E-14
ASPHD2	1.0834138	2.12465	4.20E-37	2.77E-36
ZNF668	1.0830861	2.596767	1.06E-72	1.77E-71
FREM2	1.0825893	3.049904	4.29E-05	6.50E-05
RASD2	1.0825834	4.310922	6.48E-11	1.38E-10
STH	1.0824794	-3.14793	5.93E-06	9.50E-06
CCDC67	1.0823344	-0.70448	1.97E-08	3.67E-08
FAIM3	1.0820972	3.904951	1.59E-16	4.49E-16
MYO19	1.0813826	5.400568	7.16E-47	6.37E-46
ZMYND19	1.0807599	4.520348	1.49E-48	1.39E-47
MEIS3	1.0806691	4.677391	1.05E-20	3.57E-20
C8orf76	1.0806663	3.179768	1.16E-42	9.14E-42
DCAF10	1.0806146	6.90934	1.68E-24	6.73E-24
ZNF587	1.0804689	5.626318	1.11E-23	4.29E-23
ALOXE3	1.0802534	0.319916	4.21E-09	8.14E-09
C9orf89	1.0801494	4.894267	2.60E-37	1.73E-36
ENSG00000173213	1.0797	-1.23463	6.06E-10	1.23E-09
IFI30	1.0794528	0.094118	2.94E-24	1.17E-23

Gene names	logFC	log2CPM	P.Value	adj.P.Val
IFI44	1.0794117	5.464492	6.79E-14	1.68E-13
RPP25	1.0788294	4.165449	8.34E-19	2.60E-18
CCDC144NL	1.0781825	-1.9937	0.0013028	0.00178984
RGS19	1.0776801	4.263521	3.56E-49	3.38E-48
CFAP74	1.0775538	-0.60616	1.75E-08	3.28E-08
DNAH10OS	1.0772125	2.221814	1.66E-18	5.09E-18
DPCD	1.0771826	4.316975	7.09E-41	5.31E-40
COCH	1.0768263	1.909725	4.40E-06	7.12E-06
CD79A	1.0767053	3.709721	1.44E-06	2.40E-06
TMEM155	1.0754015	-2.06917	5.31E-13	1.26E-12
ENSG00000261732	1.0753582	-1.64986	2.80E-16	7.83E-16
ILDR1	1.0752544	3.81801	4.90E-28	2.29E-27
REC8	1.0752351	2.924153	3.46E-12	7.85E-12
PRSS23	1.0751661	8.085004	4.68E-14	1.17E-13
SCRIB	1.0747957	6.790898	3.01E-31	1.61E-30
SLC27A4	1.0745072	5.674159	4.95E-44	4.08E-43
TELO2	1.0743575	5.584391	2.19E-34	1.32E-33
RFC2	1.0738718	5.278128	3.55E-56	4.04E-55
SCAMP3	1.073827	6.764941	1.96E-61	2.55E-60
ZC3H3	1.0734059	5.223456	6.30E-51	6.27E-50
EPPK1	1.0731983	5.656267	7.86E-14	1.94E-13
MYB	1.0729708	6.52264	8.00E-14	1.98E-13
PET100	1.0722321	4.575174	4.90E-31	2.61E-30
LRRC19	1.0721759	-2.17715	1.78E-08	3.33E-08
PTRH2	1.0721159	4.914958	8.92E-29	4.30E-28
GPR31	1.0717046	-2.30699	1.66E-11	3.64E-11
LDHC	1.0716496	-0.16891	6.89E-06	1.10E-05
TNFRSF12A	1.0715019	5.647806	8.84E-19	2.75E-18

Gene names	logFC	log2CPM	P.Value	adj.P.Val
HYLS1	1.0714373	3.418422	3.22E-43	2.59E-42
VWA1	1.0714236	6.758292	1.03E-27	4.75E-27
IL2RG	1.0705551	4.916813	2.50E-12	5.72E-12
RP11_295K3.1	1.070323	-1.17071	7.98E-15	2.07E-14
RAD21	1.0701167	8.814916	2.79E-30	1.44E-29
MICALCL	1.0700917	2.209912	3.25E-13	7.80E-13
FAM19A3	1.0700416	0.35339	1.34E-05	2.10E-05
ERBB3	1.0700407	8.64375	3.75E-27	1.69E-26
SLMO1	1.0698522	2.039021	1.45E-25	6.09E-25
ST3GAL4	1.0697451	5.248558	4.65E-28	2.18E-27
GRIN3B	1.0697153	-1.82746	5.41E-10	1.10E-09
ASNS	1.069488	5.183253	1.58E-24	6.34E-24
RUVBL1	1.0694869	5.995128	6.33E-59	7.72E-58
TRAF4	1.0691966	6.661541	2.60E-29	1.29E-28
TMEM150C	1.0683433	6.386727	3.64E-07	6.30E-07
CYB561D2	1.0680974	4.757505	4.03E-41	3.04E-40
PPFIA4	1.0672735	2.308694	4.88E-10	9.94E-10
DPM2	1.0668611	5.864389	1.20E-53	1.27E-52
CAMK1G	1.0663866	-1.09905	9.79E-12	2.17E-11
TRABD	1.065855	5.325891	7.94E-34	4.68E-33
ST14	1.065607	7.440509	1.33E-25	5.61E-25
MYH13	1.0654065	-0.80241	1.59E-11	3.48E-11
CLN3	1.065066	5.318978	3.21E-41	2.43E-40
ZNF681	1.0648538	3.55917	6.11E-28	2.85E-27
RAB3D	1.0643623	6.160148	9.42E-39	6.56E-38
WDR24	1.064062	4.149804	6.97E-42	5.39E-41
CD300LF	1.064041	1.546906	5.13E-23	1.93E-22
WDR54	1.0634079	3.885272	2.02E-31	1.09E-30
IFIT2	1.0628096	5.460693	1.05E-13	2.58E-13

Gene names	logFC	log2CPM	P.Value	adj.P.Val
HM13	1.0627781	7.658039	1.19E-63	1.64E-62
ALDH18A1	1.0627743	7.143506	1.09E-70	1.76E-69
C19orf67	1.0625933	-3.06739	1.98E-09	3.92E-09
SHB	1.0624126	4.256393	8.12E-41	6.07E-40
CGREF1	1.0614595	4.125542	1.85E-14	4.73E-14
TBCB	1.0614525	6.084168	1.30E-45	1.13E-44
ELAVL4	1.0610941	-0.59611	1.64E-08	3.07E-08
MDS2	1.0607534	-2.94345	5.52E-09	1.06E-08
SCGB2A1	1.0600909	4.852737	0.0008943	0.00124376
C19orf24	1.0599202	5.022048	9.88E-29	4.75E-28
MYDGF	1.0595075	6.449558	8.12E-44	6.65E-43
PRR5_ARHGAP8	1.059073	-0.33424	2.94E-16	8.20E-16
GFI1B	1.0584778	-2.52265	2.47E-06	4.06E-06
ISYNA1	1.0582272	6.111266	1.76E-20	5.90E-20
TRERF1	1.0580941	4.991696	1.49E-23	5.71E-23
CSF3R	1.058043	4.889604	2.51E-13	6.05E-13
TOR3A	1.0580254	6.21207	9.91E-78	1.81E-76
CD27	1.0575963	2.498421	1.02E-10	2.15E-10
TBC1D7	1.0575713	4.250704	4.99E-42	3.87E-41
FFAR2	1.0574905	1.877652	1.39E-07	2.46E-07
ELL3	1.0573312	1.200362	1.10E-25	4.67E-25
MCM6	1.0572006	6.111327	1.20E-39	8.60E-39
FAM132A	1.0571603	-0.22855	4.62E-10	9.44E-10
FUT8	1.0570261	6.272797	1.10E-25	4.67E-25
PTMS	1.0569289	8.405612	1.31E-25	5.53E-25
COL13A1	1.0568452	2.053108	4.55E-17	1.32E-16
TANC2	1.0566943	6.419911	4.52E-20	1.49E-19
CABYR	1.0563011	3.35362	1.51E-20	5.09E-20

Gene names	logFC	log2CPM	P.Value	adj.P.Val
ABCA3	1.0544426	6.82836	4.10E-20	1.35E-19
GPATCH2	1.054438	5.262392	3.26E-32	1.81E-31
GIPC1	1.0543691	6.760239	8.90E-38	5.99E-37
HAL	1.0537474	-0.35425	8.32E-08	1.49E-07
CD3D	1.0533645	2.720401	3.39E-10	6.97E-10
FBXO6	1.0530638	4.214528	2.73E-40	2.00E-39
C20orf24	1.052622	4.373762	5.94E-44	4.89E-43
NRSN2	1.0525775	5.841608	9.64E-45	8.09E-44
BCL3	1.0524686	6.079896	1.45E-35	9.07E-35
SULT2B1	1.0521328	3.836248	2.76E-10	5.69E-10
ESRP2	1.0520454	5.874172	8.23E-31	4.33E-30
ZFPM1	1.0519288	2.788	4.78E-29	2.33E-28
TBC1D9	1.0518912	8.828703	7.72E-12	1.72E-11
RAB17	1.0514763	4.895205	3.49E-19	1.11E-18
GPR89A	1.0510274	2.841632	7.32E-55	8.06E-54
SLC1A4	1.051017	6.829841	1.65E-23	6.33E-23
BYSL	1.050677	4.901962	5.65E-33	3.22E-32
KRTCAP3	1.0498744	4.703377	8.10E-16	2.21E-15
BAK1	1.0498502	4.598828	5.61E-52	5.73E-51
CFL1	1.0493388	9.758891	5.85E-62	7.77E-61
POLD4	1.0487903	4.28397	1.50E-28	7.19E-28
LIMK1	1.0486178	5.854201	1.37E-55	1.54E-54
CRELD2	1.0485786	5.392456	2.94E-32	1.63E-31
ZBTB42	1.0483245	5.159227	6.71E-28	3.12E-27
DUSP9	1.0478985	0.917098	6.78E-05	0.00010161
COL8A1	1.0476222	6.809301	8.95E-15	2.32E-14
ASIC4	1.0472823	-2.16569	9.60E-10	1.92E-09
KDM5B	1.0469844	7.557479	3.40E-51	3.41E-50
CKAP4	1.0469668	7.321542	1.06E-50	1.05E-49

Gene names	logFC	log2CPM	P.Value	adj.P.Val
GZMA	1.0469389	2.737774	8.81E-11	1.86E-10
KLHL17	1.0466222	2.78428	1.78E-29	8.87E-29
ASB13	1.0464541	6.258916	1.81E-27	8.28E-27
CEBPE	1.0451366	-2.82147	2.09E-14	5.32E-14
PARP9	1.0449597	6.87238	3.46E-34	2.07E-33
MFSD12	1.0446953	5.724598	5.46E-49	5.15E-48
DHTKD1	1.0444378	6.322607	3.15E-36	2.02E-35
BAIAP2	1.0441463	6.11354	1.44E-30	7.51E-30
IFI35	1.0436632	5.497915	1.40E-21	4.94E-21
STIP1	1.0432788	7.618563	2.24E-48	2.08E-47
RNF187	1.0428915	7.607344	4.72E-62	6.29E-61
DNMT1	1.0428796	6.784679	2.93E-50	2.86E-49
B3GNT4	1.0427843	-0.15336	6.15E-16	1.69E-15
PSD3	1.0426445	6.161145	2.55E-10	5.28E-10
DNAJB13	1.0424714	-1.27859	3.44E-12	7.79E-12
C11orf84	1.0424566	4.545259	4.35E-41	3.27E-40
LSM7	1.0422158	4.985579	6.07E-31	3.21E-30
SLC25A19	1.0419259	3.179832	3.57E-38	2.44E-37
SMIM5	1.0413129	0.612316	2.80E-14	7.10E-14
RNPEP	1.0409024	7.124967	3.11E-61	4.03E-60
EPHB2	1.0404795	3.842768	1.36E-21	4.82E-21
PRAF2	1.0404258	5.058546	9.95E-38	6.69E-37
ATP12A	1.0401353	-0.96531	0.0010748	0.00148663
HSPBP1	1.0398101	5.375566	7.46E-33	4.23E-32
ENSG00000187905	1.0395318	-3.35336	2.52E-05	3.88E-05
SLC19A1	1.039371	4.758282	6.23E-33	3.55E-32
VPS37D	1.0391784	2.211042	6.62E-21	2.27E-20
AMMECR1	1.0387656	4.535693	9.64E-32	5.25E-31

Gene names	logFC	log2CPM	P.Value	adj.P.Val
NLRP12	1.0387329	-0.18406	1.63E-13	3.98E-13
ENSG00000273259	1.0383667	1.093453	2.68E-09	5.24E-09
P4HA1	1.0380018	6.482397	3.28E-30	1.68E-29
DERL3	1.0379545	4.249359	7.39E-13	1.74E-12
POLB	1.0375669	5.325623	2.19E-28	1.04E-27
RPL26L1	1.0374752	4.255569	6.25E-59	7.63E-58
SHARPIN	1.0374204	6.229767	6.22E-35	3.82E-34
RANBP17	1.0372711	2.796581	9.72E-18	2.91E-17
E4F1	1.0372217	5.166661	2.78E-35	1.73E-34
MRPL17	1.0371477	5.848536	1.61E-53	1.70E-52
IRX5	1.0370912	5.821842	7.05E-25	2.88E-24
HMG20B	1.036844	6.73929	3.36E-39	2.38E-38
ATP6V0C	1.0365869	5.327329	2.08E-42	1.63E-41
CGN	1.0365204	6.554232	1.26E-18	3.90E-18
NOX1	1.0363891	0.378438	8.50E-27	3.78E-26
TPGS1	1.0362172	1.558113	2.85E-13	6.86E-13
C1orf159	1.0361494	3.796615	3.71E-34	2.22E-33
SCCPDH	1.0356799	6.800258	4.76E-23	1.79E-22
CCDC96	1.0353052	2.532752	3.66E-20	1.21E-19
KCNK12	1.0347659	-0.99193	3.09E-08	5.68E-08
THOC3	1.0346957	3.374409	6.70E-44	5.51E-43
LSM1	1.0334155	4.916828	4.71E-22	1.70E-21
TMEM160	1.032757	2.232735	1.55E-19	4.97E-19
PNP	1.0326321	5.576895	6.21E-32	3.40E-31
KRTCAP2	1.0325479	4.654368	2.78E-39	1.97E-38
TLE3	1.0323393	7.200662	1.11E-47	1.01E-46
ABHD2	1.0323299	8.015518	1.14E-15	3.10E-15
FANCD2OS	1.0322743	-2.0964	8.91E-09	1.69E-08
POLD1	1.0322355	4.69463	3.43E-35	2.12E-34

Gene names	logFC	log2CPM	P.Value	adj.P.Val
CYP4F3	1.0322316	0.770688	0.0001214	0.0001792
LIN9	1.0320859	3.670825	6.35E-35	3.90E-34
SPRED3	1.0320414	1.787794	1.13E-26	5.00E-26
PNKD	1.0320168	6.042799	2.82E-38	1.94E-37
BRMS1	1.0316189	5.760378	7.16E-37	4.68E-36
ASTL	1.0314773	-1.19246	1.62E-10	3.38E-10
C16orf91	1.0314493	3.834788	1.10E-40	8.18E-40
TNFSF13B	1.0314121	3.858364	7.53E-17	2.16E-16
FAM213B	1.0311175	5.715575	2.66E-39	1.89E-38
HPCA	1.0310952	-0.98006	2.72E-14	6.89E-14
KIAA1024	1.0309102	1.569033	9.31E-21	3.17E-20
SCO2	1.0303741	4.218171	5.18E-34	3.08E-33
TTC29	1.0300933	-1.86429	3.71E-05	5.66E-05
HRASLS	1.0296045	1.499306	1.50E-07	2.65E-07
GPR128	1.0292887	-0.20405	0.0423199	0.05130974
EPT1	1.0290895	6.214645	1.57E-44	1.31E-43
BOLA1	1.0290229	4.267801	6.64E-30	3.35E-29
GAPT	1.0289333	2.155797	3.42E-17	9.97E-17
DARS2	1.0287638	5.528364	3.07E-41	2.33E-40
RIPPLY2	1.0284447	-2.68328	0.0110513	0.01414271
MAP4K1	1.0282242	3.10769	1.22E-15	3.29E-15
PPAN	1.0280488	2.55604	2.68E-21	9.35E-21
LYPLA1	1.0279169	6.738299	1.05E-31	5.71E-31
GRIN1	1.0278842	1.610659	1.97E-09	3.89E-09
ORAI2	1.0270841	5.708849	3.37E-53	3.55E-52
FBXW12	1.0269278	-1.45577	2.40E-13	5.79E-13
CCL25	1.0268123	-2.29938	4.04E-05	6.14E-05
ATHL1	1.0266087	6.306137	5.67E-09	1.09E-08

Gene names	logFC	log2CPM	P.Value	adj.P.Val
PAICS	1.0264133	7.536055	1.14E-46	1.01E-45
SPINT1	1.0256478	7.788404	5.06E-35	3.12E-34
ADCY1	1.0254344	5.851112	9.23E-07	1.56E-06
AP1S3	1.0252362	2.36111	2.36E-26	1.03E-25
TMSB10	1.0249352	10.07121	2.40E-22	8.78E-22
P4HB	1.0249023	9.985771	2.09E-39	1.49E-38
VANGL1	1.0244872	5.759304	1.68E-45	1.45E-44
PRDX1	1.0243217	8.538324	5.82E-43	4.64E-42
ARMC4	1.0242168	0.287189	1.31E-05	2.06E-05
RUVBL2	1.0241418	6.573988	7.34E-43	5.83E-42
CD2	1.023851	4.052898	1.02E-10	2.14E-10
FKBPL	1.0238407	3.405352	5.82E-42	4.51E-41
PSMC4	1.0237326	6.598452	7.33E-55	8.06E-54
CERCAM	1.0236957	6.536837	7.95E-30	4.00E-29
AP2S1	1.0236921	6.413603	1.81E-40	1.34E-39
APOBEC3H	1.023107	-0.27586	1.23E-13	3.01E-13
CBX3	1.0227151	7.991247	1.95E-68	2.99E-67
GDPD1	1.0224048	2.835001	2.80E-18	8.55E-18
CCL5	1.0221265	5.44489	1.06E-10	2.23E-10
TMUB1	1.022022	5.038074	3.67E-31	1.96E-30
IGFLR1	1.0217527	0.912354	1.07E-26	4.71E-26
MMP17	1.021549	3.00893	1.43E-12	3.31E-12
HCN3	1.0212224	2.739619	4.28E-26	1.85E-25
FAM178B	1.0209785	1.530186	1.16E-06	1.95E-06
H3F3A	1.0207691	5.841346	3.23E-54	3.50E-53
APBA2	1.0207353	2.926363	8.70E-11	1.84E-10
RAB33A	1.020648	-0.08227	3.32E-20	1.10E-19
DNAJA4	1.0206282	6.33755	1.50E-21	5.29E-21
CDCP1	1.0205539	5.888227	2.79E-21	9.75E-21

Gene names	logFC	log2CPM	P.Value	adj.P.Val
CXXC5	1.0201407	6.824455	2.16E-23	8.22E-23
SIX1	1.0196693	4.905946	2.93E-08	5.41E-08
ABCB9	1.0196005	3.650148	2.60E-24	1.03E-23
ZNF93	1.0194934	3.496887	7.68E-32	4.20E-31
PDXP	1.0194522	-0.63898	9.65E-18	2.89E-17
SF3B4	1.0189271	6.959773	6.43E-55	7.08E-54
PCDHGA8	1.0188107	-0.51104	2.43E-06	3.99E-06
IRF9	1.0187218	2.777034	8.16E-22	2.91E-21
DNAAF1	1.0186164	2.247175	4.87E-10	9.93E-10
LURAP1L	1.0184561	5.476283	1.82E-13	4.42E-13
ACP5	1.0183809	6.399894	9.81E-14	2.41E-13
OVOL2	1.0183644	3.870685	1.57E-28	7.53E-28
CD72	1.0179679	2.224965	7.62E-19	2.38E-18
IL31RA	1.0175891	-2.41274	5.14E-07	8.81E-07
SKA2	1.0168507	5.462906	5.24E-29	2.56E-28
HLA_DQB1	1.0168173	7.105142	5.43E-15	1.42E-14
GAPDH	1.0163379	11.15714	9.26E-27	4.11E-26
MRPS23	1.0163324	5.848218	3.43E-41	2.60E-40
SRXN1	1.016243	0.842467	1.19E-30	6.22E-30
ITGAL	1.0160695	4.917911	2.78E-17	8.15E-17
CHML	1.0159932	5.341097	3.36E-22	1.22E-21
RGS10	1.0159603	5.404351	2.51E-31	1.34E-30
ISOC2	1.015612	5.609379	1.79E-30	9.30E-30
ILF2	1.0152119	8.110706	4.24E-50	4.11E-49
NIPSNAP1	1.0146325	6.49786	2.36E-50	2.31E-49
SPRR3	1.01461	0.762455	0.0472118	0.05695124
STAP2	1.0145888	5.32151	1.56E-25	6.54E-25
CKS1B	1.0145871	5.062176	4.71E-28	2.21E-27

Gene names	logFC	log2CPM	P.Value	adj.P.Val
TRIM3	1.0145762	5.073556	1.97E-23	7.52E-23
FAAH	1.014055	5.188484	1.32E-23	5.07E-23
IL1R2	1.0135792	0.94984	2.18E-06	3.59E-06
SP6	1.0135577	3.872595	4.74E-12	1.07E-11
BLNK	1.0131023	4.270218	2.72E-30	1.40E-29
RP11_345J4.5	1.0126877	2.326499	6.80E-41	5.09E-40
RGS14	1.0119826	4.272567	1.49E-27	6.81E-27
SMPD3	1.0119048	3.436142	5.04E-11	1.08E-10
SH3BP1	1.0118078	4.347612	5.12E-29	2.50E-28
SPTBN2	1.0115867	6.169843	3.50E-12	7.93E-12
ZNF474	1.011555	-0.94114	5.27E-14	1.32E-13
RHBDF2	1.0115289	5.097028	1.35E-33	7.92E-33
MAP7D2	1.0106003	1.113643	9.42E-06	1.49E-05
RHNO1	1.0095012	5.210651	3.24E-50	3.15E-49
ARL6IP1	1.0093181	8.435748	2.42E-46	2.13E-45
VSTM1	1.0092487	-3.14891	3.54E-06	5.75E-06
JOSD2	1.0089331	4.116444	1.61E-20	5.43E-20
NARFL	1.0088511	5.166719	3.77E-37	2.49E-36
TMC6	1.0086055	5.719384	1.08E-27	4.99E-27
WBSCR27	1.0085258	2.104025	8.03E-13	1.89E-12
ABCB8	1.0085112	5.311935	7.11E-55	7.84E-54
KCTD6	1.0084461	4.362941	1.29E-17	3.85E-17
NDUFS6	1.0080903	5.759792	3.39E-26	1.47E-25
CAPN15	1.0079761	5.27687	2.21E-44	1.84E-43
CARD14	1.0079037	3.822532	2.54E-19	8.11E-19
SLC35B1	1.0077263	5.97928	1.81E-33	1.05E-32
SCAMP5	1.0076066	4.627155	6.15E-20	2.01E-19
PSMB9	1.0070077	5.254319	6.23E-15	1.63E-14
SRRM5	1.0068309	0.231965	7.49E-29	3.62E-28

Gene names	logFC	log2CPM	P.Value	adj.P.Val
SRP9	1.0067608	7.889657	1.05E-61	1.38E-60
MFSD2A	1.0066373	3.34891	6.74E-10	1.36E-09
SLC39A7	1.0063752	7.776647	2.93E-47	2.64E-46
SSR4	1.0063635	7.587443	4.15E-31	2.21E-30
SPATC1L	1.0059948	3.144454	5.91E-15	1.54E-14
NCDN	1.0055957	5.760856	7.25E-38	4.90E-37
ZNF579	1.0055886	3.602268	2.51E-25	1.05E-24
PPP1R14D	1.0052714	-0.92143	5.28E-09	1.02E-08
SCNM1	1.0051095	5.040804	2.80E-42	2.19E-41
E2F3	1.0050361	4.992633	5.50E-29	2.68E-28
POTEF	1.0047442	-0.92318	1.05E-05	1.67E-05
GFRA3	1.0045826	0.885833	0.0003132	0.00044956
RANGAP1	1.0043056	7.103956	5.28E-42	4.10E-41
UFSP1	1.0041227	0.846123	2.50E-21	8.73E-21
UNC93B1	1.0034411	5.550348	4.24E-31	2.25E-30
ATPIF1	1.0031655	6.273946	3.60E-40	2.63E-39
CDK2AP2	1.0025949	5.762736	2.64E-29	1.30E-28
RPS6KB2	1.0024982	5.432427	3.81E-37	2.52E-36
MSI2	1.002084	7.116998	7.46E-27	3.32E-26
DLG3	1.0017835	6.428947	9.08E-53	9.43E-52
NME4	1.0017503	7.023875	4.41E-30	2.25E-29
MTFR1	1.0016565	5.377068	1.81E-27	8.27E-27
B3GAT3	1.0012859	5.454781	4.90E-33	2.80E-32
TCEB1	1.0010847	5.834454	3.81E-38	2.60E-37
NR2C2AP	1.0005578	4.826273	8.10E-38	5.47E-37
PMM2	1.0003537	4.399044	2.43E-52	2.50E-51

Table S6. Functional enrichment analysis results of the DEGs at the intersection of the P4 and MPA group with BRCA group.

GroupID	Term	Description	LogP	Log(q-value)	Symbols
1_Summary	R-HSA-1640170	Cell Cycle	-12.68505396	-8.339	BRCA1,CDKN2D,CENPA,FEN1,H2BC5,H3-3A,HMMR,POLE2,RAD21,RAD51,RRM2,H3C2,H4C3,CCNE2,KNTC1,ZWINT,MCM10,RCC2,CDCA5,CENPS,ECT2,PIK3R2,KIF14,DEPDC1B,IQGAP3
1_Member	R-HSA-1640170	Cell Cycle	-12.68505396	-8.339	BRCA1,CDKN2D,CENPA,FEN1,H2BC5,H3-3A,HMMR,POLE2,RAD21,RAD51,RRM2,H3C2,H4C3,CCNE2,KNTC1,ZWINT,MCM10,RCC2,CDCA5,CENPS
1_Member	R-HSA-69278	Cell Cycle, Mitotic	-12.17532439	-8.130	CDKN2D,CENPA,FEN1,H2BC5,H3-3A,HMMR,POLE2,RAD21,RRM2,H3C2,H4C3,CCNE2,KNTC1,ZWINT,MCM10,RCC2,CDCA5,CENPS
1_Member	R-HSA-194315	Signaling by Rho GTPases	-9.281111223	-5.537	CENPA,ECT2,H2BC5,H3-3A,PIK3R2,H3C2,H4C3,KNTC1,KIF14,ZWINT,DEPDC1B,RCC2,IQGAP3,CENPS
1_Member	R-HSA-195258	RHO GTPase Effectors	-7.768680011	-4.627	CENPA,H2BC5,H3-3A,H3C2,H4C3,KNTC1,KIF14,ZWINT,RCC2,IQGAP3,CENPS
1_Member	R-HSA-69620	Cell Cycle Checkpoints	-7.146848149	-4.123	BRCA1,CENPA,H2BC5,H4C3,CCNE2,KNTC1,ZWINT,MCM10,RCC2,CENPS
1_Member	R-HSA-68886	M Phase	-6.690219228	-3.742	CENPA,H2BC5,H3-3A,RAD21,H3C2,H4C3,KNTC1,ZWINT,RCC2,CDCA5,CENPS
1_Member	R-HSA-2500257	Resolution of Sister Chromatid Cohesion	-6.567837612	-3.669	CENPA,RAD21,KNTC1,ZWINT,RCC2,CDCA5,CENPS

1_Member	R-HSA-2467813	Separation of Sister Chromatids	-5.356322501	-2.654	CENPA,RAD21,KNTC1,ZWINT,RCC2,CDCA5,CENPS
1_Member	R-HSA-68877	Mitotic Prometaphase	-5.168719717	-2.495	CENPA,RAD21,KNTC1,ZWINT,RCC2,CDCA5,CENPS
1_Member	R-HSA-68882	Mitotic Anaphase	-4.758158372	-2.128	CENPA,RAD21,KNTC1,ZWINT,RCC2,CDCA5,CENPS
1_Member	R-HSA-2555396	Mitotic Metaphase and Anaphase	-4.746343618	-2.124	CENPA,RAD21,KNTC1,ZWINT,RCC2,CDCA5,CENPS
1_Member	R-HSA-141424	Amplification of signal from the kinetochores	-4.690552027	-2.086	CENPA,KNTC1,ZWINT,RCC2,CENPS
1_Member	R-HSA-141444	Amplification of signal from unattached kinetochores via a MAD2 inhibitory signal	-4.690552027	-2.086	CENPA,KNTC1,ZWINT,RCC2,CENPS
1_Member	R-HSA-69618	Mitotic Spindle Checkpoint	-4.349438912	-1.872	CENPA,KNTC1,ZWINT,RCC2,CENPS
1_Member	R-HSA-9648025	EML4 and NUDC in mitotic spindle formation	-4.277239841	-1.817	CENPA,KNTC1,ZWINT,RCC2,CENPS
1_Member	R-HSA-5663220	RHO GTPases Activate Formins	-3.908215753	-1.572	CENPA,KNTC1,ZWINT,RCC2,CENPS
2_Summary	GO:0071103	DNA conformation change	-10.03058168	-6.161	CENPA,H2BC5,H3-3A,HMGB2,HMGB3,RAD51,H3C2,H4C3,POLQ,CHD5,NUSAP1,CDCA5,CENPS,BRCA1,RAD21,FEN1,SAP30,ST3GAL4,CDKN2D,CCNE2,PIK3R2,RDH16,HDAC11,NEIL3,ATAD2,MCM10,SFXN1,ELOC,FANCD2
2_Member	GO:0071103	DNA conformation change	-10.03058168	-6.161	CENPA,H2BC5,H3-3A,HMGB2,HMGB3,RAD51,H3C2,H4C3,POLQ,CHD5,NUSAP1,CDCA5,CENPS
2_Member	GO:0006323	DNA packaging	-8.533166724	-5.266	CENPA,H2BC5,H3-3A,HMGB2,H3C2,H4C3,CHD5,NUSAP1,CDCA5,CENPS
2_Member	R-HSA-1500620	Meiosis	-6.762681032	-3.797	BRCA1,H2BC5,H3-3A,RAD21,RAD51,H3C2,H4C3

2_Member	GO:0034728	nucleosome organization	-6.63395606	-3.719	CENPA,H2BC5,H3-3A,HMGB2,H3C2,H4C3,CHD5,CENPS
2_Member	R-HSA-912446	Meiotic recombination	-6.281272763	-3.412	BRCA1,H2BC5,H3-3A,RAD51,H3C2,H4C3
2_Member	GO:0071824	protein-DNA complex subunit organization	-6.24632237	-3.391	CENPA,H2BC5,H3-3A,HMGB2,RAD51,H3C2,H4C3,CHD5,CENPS
2_Member	R-HSA-1474165	Reproduction	-6.194720232	-3.363	BRCA1,H2BC5,H3-3A,RAD21,RAD51,H3C2,H4C3
2_Member	GO:0006334	nucleosome assembly	-6.154004878	-3.352	CENPA,H2BC5,H3-3A,HMGB2,H3C2,H4C3,CENPS
2_Member	GO:0031497	chromatin assembly	-5.777498762	-2.999	CENPA,H2BC5,H3-3A,HMGB2,H3C2,H4C3,CENPS
2_Member	GO:0065004	protein-DNA complex assembly	-5.704763138	-2.950	CENPA,H2BC5,H3-3A,HMGB2,RAD51,H3C2,H4C3,CENPS
2_Member	GO:0006338	chromatin remodeling	-5.386426969	-2.663	CENPA,H3-3A,HMGB2,HMGB3,H4C3,CHD5,CENPS
2_Member	GO:0006333	chromatin assembly or disassembly	-5.371331262	-2.659	CENPA,H2BC5,H3-3A,HMGB2,H3C2,H4C3,CENPS
2_Member	R-HSA-73886	Chromosome Maintenance	-5.028073354	-2.381	CENPA,FEN1,H2BC5,H3-3A,H4C3,CENPS
2_Member	GO:0031055	chromatin remodeling at centromere	-4.684337456	-2.086	CENPA,H3-3A,H4C3,CENPS
2_Member	GO:0006336	DNA replication-independent nucleosome assembly	-4.47587428	-1.923	CENPA,H3-3A,H4C3,CENPS
2_Member	R-HSA-427413	NoRC negatively regulates rRNA expression	-4.463095235	-1.923	H2BC5,H3-3A,H3C2,H4C3,SAP30
2_Member	GO:0034724	DNA replication-independent nucleosome organization	-4.443589412	-1.917	CENPA,H3-3A,H4C3,CENPS
2_Member	R-HSA-1912422	Pre-NOTCH Expression and Processing	-4.424460061	-1.905	H2BC5,H3-3A,ST3GAL4,H3C2,H4C3
2_Member	R-HSA-5250941	Negative epigenetic regulation of rRNA expression	-4.405428839	-1.904	H2BC5,H3-3A,H3C2,H4C3,SAP30

2_Member	GO:0034508	centromere complex assembly	-4.380889702	-1.892	CENPA,H3-3A,H4C3,CENPS
2_Member	R-HSA-2559582	Senescence-Associated Secretory Phenotype (SASP)	-4.367921622	-1.885	CDKN2D,H2BC5,H3-3A,H3C2,H4C3
2_Member	GO:0043486	histone exchange	-4.291182668	-1.826	CENPA,H4C3,CHD5,CENPS
2_Member	R-HSA-2559583	Cellular Senescence	-4.209961518	-1.772	CDKN2D,H2BC5,H3-3A,H3C2,H4C3,CCNE2
2_Member	R-HSA-73728	RNA Polymerase I Promoter Opening	-4.178864891	-1.757	H2BC5,H3-3A,H3C2,H4C3
2_Member	R-HSA-2559580	Oxidative Stress Induced Senescence	-4.140554379	-1.729	CDKN2D,H2BC5,H3-3A,H3C2,H4C3
2_Member	R-HSA-5334118	DNA methylation	-4.125528394	-1.719	H2BC5,H3-3A,H3C2,H4C3
2_Member	R-HSA-9006931	Signaling by Nuclear Receptors	-4.106480939	-1.705	H2BC5,H3-3A,PIK3R2,RAD21,H3C2,H4C3,RDH16
2_Member	R-HSA-5625886	Activated PKN1 stimulates transcription of AR (androgen receptor) regulated genes KLK2 and KLK3	-4.073917254	-1.687	H2BC5,H3-3A,H3C2,H4C3
2_Member	R-HSA-427359	SIRT1 negatively regulates rRNA expression	-4.04872585	-1.666	H2BC5,H3-3A,H3C2,H4C3
2_Member	R-HSA-212300	PRC2 methylates histones and DNA	-3.928441321	-1.573	H2BC5,H3-3A,H3C2,H4C3
2_Member	R-HSA-8939211	ESR-mediated signaling	-3.913979058	-1.572	H2BC5,H3-3A,PIK3R2,RAD21,H3C2,H4C3
2_Member	R-HSA-2299718	Condensation of Prophase Chromosomes	-3.90544459	-1.572	H2BC5,H3-3A,H3C2,H4C3
2_Member	R-HSA-606279	Deposition of new CENPA-containing nucleosomes at the centromere	-3.90544459	-1.572	CENPA,H2BC5,H4C3,CENPS
2_Member	R-HSA-774815	Nucleosome assembly	-3.90544459	-1.572	CENPA,H2BC5,H4C3,CENPS

2_Member	R-HSA-427389	ERCC6 (CSB) and EHMT2 (G9a) positively regulate rRNA expression	-3.860434183	-1.539	H2BC5,H3-3A,H3C2,H4C3
2_Member	R-HSA-1221632	Meiotic synapsis	-3.795247276	-1.487	BRCA1,H2BC5,RAD21,H4C3
2_Member	R-HSA-212165	Epigenetic regulation of gene expression	-3.781540517	-1.487	H2BC5,H3-3A,H3C2,H4C3,SAP30
2_Member	R-HSA-157118	Signaling by NOTCH	-3.780177158	-1.487	H2BC5,H3-3A,ST3GAL4,H3C2,H4C3,HDAC11
2_Member	R-HSA-9018519	Estrogen-dependent gene expression	-3.767986886	-1.479	H2BC5,H3-3A,RAD21,H3C2,H4C3
2_Member	GO:0045652	regulation of megakaryocyte differentiation	-3.732666122	-1.447	H3-3A,HMGB2,H3C2,H4C3
2_Member	GO:0043044	ATP-dependent chromatin remodeling	-3.577133376	-1.345	CENPA,H4C3,CHD5,CENPS
2_Member	R-HSA-9616222	Transcriptional regulation of granulopoiesis	-3.577133376	-1.345	H2BC5,H3-3A,H3C2,H4C3
2_Member	R-HSA-201722	Formation of the beta-catenin:TCF transactivating complex	-3.558746508	-1.338	H2BC5,H3-3A,H3C2,H4C3
2_Member	R-HSA-5250924	B-WICH complex positively regulates rRNA expression	-3.558746508	-1.338	H2BC5,H3-3A,H3C2,H4C3
2_Member	R-HSA-73772	RNA Polymerase I Promoter Escape	-3.558746508	-1.338	H2BC5,H3-3A,H3C2,H4C3
2_Member	R-HSA-1912408	Pre-NOTCH Transcription and Translation	-3.522618656	-1.313	H2BC5,H3-3A,H3C2,H4C3
2_Member	R-HSA-3214815	HDACs deacetylate histones	-3.504868285	-1.298	H2BC5,H3C2,H4C3,SAP30
2_Member	R-HSA-5625740	RHO GTPases activate PKNs	-3.487320946	-1.289	H2BC5,H3-3A,H3C2,H4C3
2_Member	R-HSA-8936459	RUNX1 regulates genes involved in megakaryocyte	-3.452818202	-1.262	H2BC5,H3-3A,H3C2,H4C3

		differentiation and platelet function			
2_Member	GO:0000183	chromatin silencing at rDNA	-3.421337316	-1.239	H3-3A,H3C2,H4C3
2_Member	GO:0030219	megakaryocyte differentiation	-3.402483044	-1.229	H3-3A,HMGB2,H3C2,H4C3
2_Member	ko05034	Alcoholism	-3.402223182	-1.229	H2BC5,H3-3A,H3C2,H4C3,HDAC11
2_Member	GO:0034080	CENP-A containing nucleosome assembly	-3.359899575	-1.198	CENPA,H4C3,CENPS
2_Member	GO:0061641	CENP-A containing chromatin organization	-3.359899575	-1.198	CENPA,H4C3,CENPS
2_Member	hsa05034	Alcoholism	-3.347901304	-1.192	H2BC5,H3-3A,H3C2,H4C3,HDAC11
2_Member	R-HSA-5250913	Positive epigenetic regulation of rRNA expression	-3.306554452	-1.162	H2BC5,H3-3A,H3C2,H4C3
2_Member	R-HSA-5578749	Transcriptional regulation by small RNAs	-3.306554452	-1.162	H2BC5,H3-3A,H3C2,H4C3
2_Member	R-HSA-977225	Amyloid fiber formation	-3.260780989	-1.129	H2BC5,H3-3A,H3C2,H4C3
2_Member	R-HSA-73854	RNA Polymerase I Promoter Clearance	-3.245827536	-1.127	H2BC5,H3-3A,H3C2,H4C3
2_Member	R-HSA-73864	RNA Polymerase I Transcription	-3.216360496	-1.106	H2BC5,H3-3A,H3C2,H4C3
2_Member	R-HSA-157579	Telomere Maintenance	-3.201841613	-1.094	FEN1,H2BC5,H3-3A,H4C3
2_Member	ko05203	Viral carcinogenesis	-3.184549236	-1.084	H2BC5,PIK3R2,H4C3,CCNE2,HDAC11
2_Member	R-HSA-5617472	Activation of anterior HOX genes in hindbrain development during early embryogenesis	-3.090518997	-1.006	H2BC5,H3-3A,H3C2,H4C3
2_Member	R-HSA-5619507	Activation of HOX genes during differentiation	-3.090518997	-1.006	H2BC5,H3-3A,H3C2,H4C3
2_Member	hsa05203	Viral carcinogenesis	-3.089736621	-1.006	H2BC5,PIK3R2,H4C3,CCNE2,HDAC11
2_Member	R-HSA-110330	Recognition and association of DNA glycosylase with site	-3.022451736	-0.957	H2BC5,H4C3,NEIL3

		containing an affected purine			
2_Member	R-HSA-110331	Cleavage of the damaged purine	-3.022451736	-0.957	H2BC5,H4C3,NEIL3
2_Member	R-HSA-73927	Depurination	-3.022451736	-0.957	H2BC5,H4C3,NEIL3
2_Member	R-HSA-8939236	RUNX1 regulates transcription of genes involved in differentiation of HSCs	-2.974553448	-0.914	H2BC5,H3-3A,H3C2,H4C3
2_Member	ko05322	Systemic lupus erythematosus	-2.937853335	-0.884	H2BC5,H3-3A,H3C2,H4C3
2_Member	R-HSA-110328	Recognition and association of DNA glycosylase with site containing an affected pyrimidine	-2.914438808	-0.875	H2BC5,H4C3,NEIL3
2_Member	R-HSA-110329	Cleavage of the damaged pyrimidine	-2.914438808	-0.875	H2BC5,H4C3,NEIL3
2_Member	R-HSA-73928	Depyrimidination	-2.914438808	-0.875	H2BC5,H4C3,NEIL3
2_Member	GO:0006342	chromatin silencing	-2.913891136	-0.875	H3-3A,H3C2,H4C3,ATAD2
2_Member	R-HSA-211000	Gene Silencing by RNA	-2.902056863	-0.865	H2BC5,H3-3A,H3C2,H4C3
2_Member	R-HSA-73929	Base-Excision Repair, AP Site Formation	-2.873859011	-0.846	H2BC5,H4C3,NEIL3
2_Member	R-HSA-68875	Mitotic Prophase	-2.833017733	-0.811	H2BC5,H3-3A,H3C2,H4C3
2_Member	hsa05322	Systemic lupus erythematosus	-2.788767921	-0.771	H2BC5,H3-3A,H3C2,H4C3
2_Member	R-HSA-9670095	Inhibition of DNA recombination at telomere	-2.778163633	-0.764	H2BC5,H3-3A,H4C3
2_Member	R-HSA-5693571	Nonhomologous End-Joining (NHEJ)	-2.759932045	-0.752	BRCA1,H2BC5,H4C3
2_Member	GO:0045814	negative regulation of gene expression, epigenetic	-2.756457498	-0.752	H3-3A,H3C2,H4C3,ATAD2

2_Member	GO:0060968	regulation of gene silencing	-2.663722054	-0.679	H3-3A,H3C2,H4C3,ATAD2
2_Member	GO:0045637	regulation of myeloid cell differentiation	-2.660616897	-0.678	H3-3A,HMGB2,HMGB3,H3C2,H4C3
2_Member	R-HSA-5693565	Recruitment and ATM-mediated phosphorylation of repair and signaling proteins at DNA double strand breaks	-2.639768819	-0.661	BRCA1,H2BC5,H4C3
2_Member	R-HSA-5693606	DNA Double Strand Break Response	-2.623582115	-0.648	BRCA1,H2BC5,H4C3
2_Member	R-HSA-2559586	DNA Damage/Telomere Stress Induced Senescence	-2.576351965	-0.610	H2BC5,H4C3,CCNE2
2_Member	R-HSA-69481	G2/M Checkpoints	-2.567395501	-0.603	BRCA1,H2BC5,H4C3,MCM10
2_Member	GO:0030099	myeloid cell differentiation	-2.41958712	-0.473	H3-3A,HMGB2,HMGB3,H3C2,H4C3,SFXN1
2_Member	R-HSA-2262752	Cellular responses to stress	-2.398244527	-0.462	CDKN2D,H2BC5,H3-3A,ELOC,H3C2,H4C3,CCNE2
2_Member	R-HSA-69473	G2/M DNA damage checkpoint	-2.365887424	-0.433	BRCA1,H2BC5,H4C3
2_Member	R-HSA-8953897	Cellular responses to external stimuli	-2.342587301	-0.411	CDKN2D,H2BC5,H3-3A,ELOC,H3C2,H4C3,CCNE2
2_Member	R-HSA-5693607	Processing of DNA double-strand break ends	-2.32816009	-0.400	BRCA1,H2BC5,H4C3
2_Member	GO:0060964	regulation of gene silencing by miRNA	-2.125581616	-0.242	H3-3A,H3C2,H4C3
2_Member	R-HSA-9610379	HCMV Late Events	-2.125581616	-0.242	H2BC5,H3C2,H4C3
2_Member	GO:1903706	regulation of hemopoiesis	-2.121651399	-0.239	FANCD2,H3-3A,HMGB2,HMGB3,H3C2,H4C3
2_Member	GO:0060147	regulation of posttranscriptional gene silencing	-2.085294495	-0.209	H3-3A,H3C2,H4C3

2_Member	GO:0060966	regulation of gene silencing by RNA	-2.085294495	-0.209	H3-3A,H3C2,H4C3
2_Member	R-HSA-201681	TCF dependent signaling in response to WNT	-2.067873545	-0.197	H2BC5,H3-3A,H3C2,H4C3
2_Member	R-HSA-8878171	Transcriptional regulation by RUNX1	-2.030140932	-0.167	H2BC5,H3-3A,H3C2,H4C3
3_Summary	GO:0006281	DNA repair	-9.041538592	-5.394	BRCA1,CDKN2D,FANCD2,FEN1,HMGB2,POLE2,RAD21,RAD51,H4C3,PCLAF,RAD51AP1,POLQ,NEIL3,CDCA5,CENPS,H2BC5,HMGB3,MUC1,ECT2,RRM2,DEPDC1B,GJB2,KCNJ11
3_Member	GO:0006281	DNA repair	-9.041538592	-5.394	BRCA1,CDKN2D,FANCD2,FEN1,HMGB2,POLE2,RAD21,RAD51,H4C3,PCLAF,RAD51AP1,POLQ,NEIL3,CDCA5,CENPS
3_Member	R-HSA-73894	DNA Repair	-8.759005034	-5.275	BRCA1,FANCD2,FEN1,H2BC5,POLE2,RAD51,H4C3,PCLAF,RAD51AP1,POLQ,NEIL3,CENPS
3_Member	R-HSA-5693538	Homology Directed Repair	-7.58225073	-4.491	BRCA1,FEN1,H2BC5,POLE2,RAD51,H4C3,RAD51AP1,POLQ
3_Member	GO:0006310	DNA recombination	-7.065056682	-4.061	BRCA1,FANCD2,FEN1,HMGB2,HMGB3,RAD21,RAD51,RAD51AP1,POLQ,CENPS
3_Member	R-HSA-5693532	DNA Double-Strand Break Repair	-6.959058071	-3.975	BRCA1,FEN1,H2BC5,POLE2,RAD51,H4C3,RAD51AP1,POLQ
3_Member	GO:0006302	double-strand break repair	-6.513825432	-3.630	BRCA1,FANCD2,FEN1,RAD21,RAD51,H4C3,RAD51AP1,POLQ,CDCA5
3_Member	R-HSA-5693567	HDR through Homologous Recombination (HRR) or Single Strand Annealing (SSA)	-5.192434101	-2.499	BRCA1,H2BC5,POLE2,RAD51,H4C3,RAD51AP1
3_Member	GO:0000724	double-strand break repair via homologous recombination	-5.081562839	-2.417	BRCA1,FEN1,RAD21,RAD51,RAD51AP1,POLQ
3_Member	GO:0000725	recombinational repair	-5.045764651	-2.390	BRCA1,FEN1,RAD21,RAD51,RAD51AP1,POLQ

3_Member	R-HSA-5685942	HDR through Homologous Recombination (HRR)	-4.073917254	-1.687	BRCA1,POLE2,RAD51,RAD51AP1
3_Member	R-HSA-5693554	Resolution of D-loop Structures through Synthesis-Dependent Strand Annealing (SDSA)	-4.01793601	-1.640	BRCA1,RAD51,RAD51AP1
3_Member	GO:2001020	regulation of response to DNA damage stimulus	-3.882335461	-1.553	BRCA1,CDKN2D,MUC1,RAD51,RAD51AP1,POLQ
3_Member	GO:0010212	response to ionizing radiation	-3.836773553	-1.520	BRCA1,ECT2,FANCD2,RAD51,RAD51AP1
3_Member	GO:0009314	response to radiation	-3.804758469	-1.492	BRCA1,CDKN2D,ECT2,FANCD2,FEN1,RAD51,PCLAF,RAD51AP1
3_Member	R-HSA-5693568	Resolution of D-loop Structures through Holliday Junction Intermediates	-3.703592366	-1.433	BRCA1,RAD51,RAD51AP1
3_Member	R-HSA-5693537	Resolution of D-Loop Structures	-3.664554155	-1.401	BRCA1,RAD51,RAD51AP1
3_Member	R-HSA-8953750	Transcriptional Regulation by E2F6	-3.626714947	-1.372	BRCA1,RAD51,RRM2
3_Member	GO:0070317	negative regulation of G0 to G1 transition	-3.421337316	-1.239	BRCA1,RAD51,RRM2
3_Member	R-HSA-5693579	Homologous DNA Pairing and Strand Exchange	-3.390230413	-1.220	BRCA1,RAD51,RAD51AP1
3_Member	GO:0070316	regulation of G0 to G1 transition	-3.301422322	-1.162	BRCA1,RAD51,RRM2
3_Member	GO:0045023	G0 to G1 transition	-3.273210108	-1.139	BRCA1,RAD51,RRM2
3_Member	GO:0010569	regulation of double-strand break repair via homologous recombination	-3.245641934	-1.127	RAD51,RAD51AP1,POLQ
3_Member	hsa03440	Homologous recombination	-3.218690063	-1.106	BRCA1,RAD51,DEPDC1B

3_Member	GO:0006282	regulation of DNA repair	-2.949984251	-0.894	BRCA1,RAD51,RAD51AP1,POLQ
3_Member	GO:0071479	cellular response to ionizing radiation	-2.778163633	-0.764	ECT2,RAD51,RAD51AP1
3_Member	GO:2000779	regulation of double-strand break repair	-2.501744071	-0.546	RAD51,RAD51AP1,POLQ
3_Member	GO:0097327	response to antineoplastic agent	-2.315864905	-0.390	BRCA1,GJB2,RAD51
3_Member	GO:0006303	double-strand break repair via nonhomologous end joining	-2.303705256	-0.379	BRCA1,H4C3,POLQ
3_Member	GO:0000018	regulation of DNA recombination	-2.222163359	-0.320	RAD51,RAD51AP1,POLQ
3_Member	GO:0000726	non-recombinational repair	-2.210996221	-0.317	BRCA1,H4C3,POLQ
3_Member	GO:0035690	cellular response to drug	-2.012746307	-0.152	BRCA1,ECT2,GJB2,KCNJ11,RAD51
4_Summary	GO:0010564	regulation of cell cycle process	-8.816908522	-5.275	BRCA1,CALR,CDKN2D,ECT2,FEN1,HMMR,MUC1,RAD21,RAD51,RRM2,KNTC1,KIF14,GPSM2,NUSAP1,RCC2,CDCA5,E2F7,POLE2,CCNE2,MCM10,IQGAP3,FANCD2,ZWINT,H3-3A,CKAP2,ATAD2,PACSIN1
4_Member	GO:0010564	regulation of cell cycle process	-8.816908522	-5.275	BRCA1,CALR,CDKN2D,ECT2,FEN1,HMMR,MUC1,RAD21,RAD51,RRM2,KNTC1,KIF14,GPSM2,NUSAP1,RCC2,CDCA5,E2F7
4_Member	GO:0044772	mitotic cell cycle phase transition	-8.667492608	-5.275	BRCA1,CDKN2D,HMMR,MUC1,POLE2,RAD21,RRM2,CCNE2,KNTC1,KIF14,MCM10,RCC2,CDCA5,IQGAP3,E2F7
4_Member	GO:0044770	cell cycle phase transition	-8.246349603	-5.014	BRCA1,CDKN2D,HMMR,MUC1,POLE2,RAD21,RRM2,CCNE2,KNTC1,KIF14,MCM10,RCC2,CDCA5,IQGAP3,E2F7
4_Member	GO:0090068	positive regulation of cell cycle process	-8.124429438	-4.924	BRCA1,ECT2,FEN1,MUC1,RAD21,KIF14,GPSM2,NUSAP1,RCC2,CDCA5,E2F7
4_Member	GO:0045787	positive regulation of cell cycle	-7.933015464	-4.763	BRCA1,CALR,ECT2,FEN1,MUC1,RAD21,KIF14,GPSM2,NUSAP1,RCC2,CDCA5,E2F7

4_Member	GO:0007346	regulation of mitotic cell cycle	-7.150494353	-4.123	BRCA1,CDKN2D,FANCD2,HMMR,MUC1,RAD21,KNTC1,KIF14,ZWINT,GPSM2,NUSAP1,RCC2,CDCA5,E2F7
4_Member	GO:1901990	regulation of mitotic cell cycle phase transition	-5.481874991	-2.748	BRCA1,CDKN2D,HMMR,MUC1,RAD21,KNTC1,KIF14,RCC2,CDCA5,E2F7
4_Member	GO:1901987	regulation of cell cycle phase transition	-5.17114445	-2.495	BRCA1,CDKN2D,HMMR,MUC1,RAD21,KNTC1,KIF14,RCC2,CDCA5,E2F7
4_Member	GO:0010389	regulation of G2/M transition of mitotic cell cycle	-3.19432299	-1.089	BRCA1,HMMR,RAD21,KIF14,RCC2
4_Member	GO:1902749	regulation of cell cycle G2/M phase transition	-3.035264839	-0.957	BRCA1,HMMR,RAD21,KIF14,RCC2
4_Member	GO:0000086	G2/M transition of mitotic cell cycle	-2.733502055	-0.736	BRCA1,HMMR,RAD21,KIF14,RCC2
4_Member	GO:0044839	cell cycle G2/M phase transition	-2.597727649	-0.626	BRCA1,HMMR,RAD21,KIF14,RCC2
4_Member	GO:0051129	negative regulation of cellular component organization	-2.284123162	-0.368	BRCA1,H3-3A,RAD21,KIF14,CKAP2,ATAD2,PACSIN1,RCC2
4_Member	GO:0034446	substrate adhesion-dependent cell spreading	-2.233446317	-0.327	CALR,KIF14,RCC2
5_Summary	GO:0007059	chromosome segregation	-8.715255396	-5.275	BRCA1,ECT2,FANCD2,FEN1,RAD21,CCNE2,KIF14,ZWINT,NUSAP1,RCC2,CDCA5,CENPS,CENPA,KNTC1,CKAP2,GPSM2,E2F7,CALR,RAD51,POLE2,H3-3A,MUC1,ATAD2
5_Member	GO:0007059	chromosome segregation	-8.715255396	-5.275	BRCA1,ECT2,FANCD2,FEN1,RAD21,CCNE2,KIF14,ZWINT,NUSAP1,RCC2,CDCA5,CENPS
5_Member	GO:0098813	nuclear chromosome segregation	-8.546999574	-5.266	ECT2,FANCD2,FEN1,RAD21,CCNE2,KIF14,ZWINT,NUSAP1,RCC2,CDCA5,CENPS
5_Member	GO:0051301	cell division	-7.725012951	-4.609	CENPA,ECT2,RAD21,CCNE2,KNTC1,KIF14,ZWINT,CKAP2,GPSM2,NUSAP1,RCC2,CDCA5,E2F7,CENPS

5_Member	GO:0000280	nuclear division	-6.638810739	-3.719	CALR,FANCD2,RAD21,RAD51,CCNE2,KNTC1,KIF14,ZWINT,NUSAP1,CDCA5,CENPS
5_Member	GO:0048285	organelle fission	-6.177892681	-3.363	CALR,FANCD2,RAD21,RAD51,CCNE2,KNTC1,KIF14,ZWINT,NUSAP1,CDCA5,CENPS
5_Member	GO:0051983	regulation of chromosome segregation	-4.463095235	-1.923	ECT2,FEN1,RAD21,RCC2,CDCA5
5_Member	GO:0007063	regulation of sister chromatid cohesion	-4.241066784	-1.798	FEN1,RAD21,CDCA5
5_Member	GO:0000819	sister chromatid segregation	-4.185685373	-1.757	FEN1,RAD21,KIF14,ZWINT,NUSAP1,CDCA5
5_Member	GO:0051984	positive regulation of chromosome segregation	-3.703592366	-1.433	FEN1,RAD21,RCC2
5_Member	GO:0034502	protein localization to chromosome	-3.692297852	-1.425	CENPA,RAD21,RCC2,CDCA5
5_Member	R-HSA-69242	S Phase	-3.612711674	-1.366	FEN1,POLE2,RAD21,CCNE2,CDCA5
5_Member	GO:0033044	regulation of chromosome organization	-3.610232571	-1.366	BRCA1,FEN1,H3-3A,MUC1,RAD21,ATAD2,CDCA5
5_Member	GO:0000070	mitotic sister chromatid segregation	-3.588071923	-1.349	RAD21,KIF14,ZWINT,NUSAP1,CDCA5
5_Member	GO:0140014	mitotic nuclear division	-3.374442076	-1.207	RAD21,KNTC1,KIF14,ZWINT,NUSAP1,CDCA5
5_Member	GO:0051783	regulation of nuclear division	-3.295199676	-1.158	CALR,RAD21,KNTC1,NUSAP1,CDCA5
5_Member	GO:0030261	chromosome condensation	-3.245641934	-1.127	H3-3A,NUSAP1,CDCA5
5_Member	GO:0007062	sister chromatid cohesion	-2.873859011	-0.846	FEN1,RAD21,CDCA5
5_Member	GO:2001251	negative regulation of chromosome organization	-2.767146414	-0.755	BRCA1,H3-3A,RAD21,ATAD2
5_Member	GO:0007088	regulation of mitotic nuclear division	-2.595600532	-0.626	RAD21,KNTC1,NUSAP1,CDCA5
5_Member	GO:0033045	regulation of sister chromatid segregation	-2.516278118	-0.559	FEN1,RAD21,CDCA5

5_Member	GO:2001252	positive regulation of chromosome organization	-2.442909319	-0.493	BRCA1,FEN1,MUC1,RAD21
5_Member	GO:0010638	positive regulation of organelle organization	-2.185370278	-0.292	BRCA1,FEN1,MUC1,RAD21,GPSM2,NUSAP1,CDCA5
6_Summary	GO:0006260	DNA replication	-8.546999574	-5.266	BRCA1,FEN1,POLE2,RAD51,RRM2,CCNE2,PCLAF,POLQ,MCM10,E2F7,CENPS,CDKN2D,MUC1,KIF14,IQGAP3,H3-3A,H3C2,H4C3,NELL2
6_Member	GO:0006260	DNA replication	-8.546999574	-5.266	BRCA1,FEN1,POLE2,RAD51,RRM2,CCNE2,PCLAF,POLQ,MCM10,E2F7,CENPS
6_Member	GO:0006261	DNA-dependent DNA replication	-7.211073191	-4.144	FEN1,POLE2,RAD51,CCNE2,POLQ,MCM10,E2F7,CENPS
6_Member	GO:0000082	G1/S transition of mitotic cell cycle	-6.18272917	-3.363	CDKN2D,MUC1,POLE2,RRM2,CCNE2,KIF14,MCM10,IQGAP3,E2F7
6_Member	GO:0044843	cell cycle G1/S phase transition	-5.905292853	-3.115	CDKN2D,MUC1,POLE2,RRM2,CCNE2,KIF14,MCM10,IQGAP3,E2F7
6_Member	GO:0032200	telomere organization	-5.623972377	-2.880	FEN1,H3-3A,POLE2,RAD51,H3C2,H4C3,CCNE2
6_Member	GO:0044786	cell cycle DNA replication	-4.099513876	-1.703	FEN1,POLE2,RAD51,E2F7
6_Member	R-HSA-453279	Mitotic G1 phase and G1/S transition	-3.781540517	-1.487	CDKN2D,POLE2,RRM2,CCNE2,MCM10
6_Member	GO:0000723	telomere maintenance	-3.625158192	-1.372	FEN1,POLE2,RAD51,H4C3,CCNE2
6_Member	GO:0006270	DNA replication initiation	-3.486039818	-1.289	POLE2,CCNE2,MCM10
6_Member	R-HSA-69306	DNA Replication	-2.9995432	-0.937	FEN1,POLE2,CCNE2,MCM10
6_Member	R-HSA-69206	G1/S Transition	-2.962217094	-0.904	POLE2,RRM2,CCNE2,MCM10
6_Member	GO:0033260	nuclear DNA replication	-2.914438808	-0.875	FEN1,POLE2,RAD51
6_Member	GO:0060249	anatomical structure homeostasis	-2.385821162	-0.451	FEN1,NELL2,POLE2,RAD51,H4C3,CCNE2
6_Member	R-HSA-69239	Synthesis of DNA	-2.085294495	-0.209	FEN1,POLE2,CCNE2
7_Summary	GO:0045786	negative regulation of cell cycle	-5.713423793	-2.950	BRCA1,CALR,CDKN2D,FANCD2,MUC1,RAD21,RAD51,RRM2,KNTC1,ZWINT,PRR

					11,E2F7,CCNE2,ELOC,CHD5,POLQ,KIF14,HMGB2
7_Member	GO:0045786	negative regulation of cell cycle	-5.713423793	-2.950	BRCA1,CALR,CDKN2D,FANCD2,MUC1,RAD21,RAD51,RRM2,KNTC1,ZWINT,PRR11,E2F7
7_Member	GO:0045930	negative regulation of mitotic cell cycle	-4.631865119	-2.043	BRCA1,CDKN2D,FANCD2,MUC1,RAD21,KNTC1,ZWINT,E2F7
7_Member	GO:0007093	mitotic cell cycle checkpoint	-4.625448252	-2.043	BRCA1,FANCD2,MUC1,KNTC1,ZWINT,E2F7
7_Member	GO:0010948	negative regulation of cell cycle process	-4.473162154	-1.923	BRCA1,CALR,CDKN2D,MUC1,RAD21,RAD51,RRM2,E2F7
7_Member	GO:0000075	cell cycle checkpoint	-3.95692276	-1.588	BRCA1,FANCD2,MUC1,KNTC1,ZWINT,E2F7
7_Member	M40	PID E2F PATHWAY	-3.928441321	-1.573	BRCA1,RRM2,CCNE2,E2F7
7_Member	GO:0007050	cell cycle arrest	-3.790171094	-1.487	BRCA1,CALR,CDKN2D,MUC1,PRR11,E2F7
7_Member	GO:0071156	regulation of cell cycle arrest	-3.306554452	-1.162	BRCA1,CALR,MUC1,E2F7
7_Member	R-HSA-6796648	TP53 Regulates Transcription of DNA Repair Genes	-2.834635756	-0.811	BRCA1,FANCD2,ELOC
7_Member	GO:1901991	negative regulation of mitotic cell cycle phase transition	-2.756021097	-0.752	BRCA1,CDKN2D,MUC1,RAD21,E2F7
7_Member	GO:0043967	histone H4 acetylation	-2.741982217	-0.740	BRCA1,MUC1,CHD5
7_Member	GO:0000077	DNA damage checkpoint	-2.735317867	-0.736	BRCA1,FANCD2,MUC1,E2F7
7_Member	GO:0072401	signal transduction involved in DNA integrity checkpoint	-2.689743835	-0.701	BRCA1,MUC1,E2F7
7_Member	GO:0072422	signal transduction involved in DNA damage checkpoint	-2.689743835	-0.701	BRCA1,MUC1,E2F7
7_Member	GO:0072395	signal transduction involved in cell cycle checkpoint	-2.672843241	-0.686	BRCA1,MUC1,E2F7

7_Member	GO:0031570	DNA integrity checkpoint	-2.634119593	-0.657	BRCA1,FANCD2,MUC1,E2F7
7_Member	GO:1901988	negative regulation of cell cycle phase transition	-2.618417185	-0.645	BRCA1,CDKN2D,MUC1,RAD21,E2F7
7_Member	GO:0071158	positive regulation of cell cycle arrest	-2.561033213	-0.599	BRCA1,MUC1,E2F7
7_Member	GO:2001021	negative regulation of response to DNA damage stimulus	-2.531001199	-0.570	CDKN2D,MUC1,POLQ
7_Member	GO:2000045	regulation of G1/S transition of mitotic cell cycle	-2.417649658	-0.473	CDKN2D,MUC1,KIF14,E2F7
7_Member	GO:0044773	mitotic DNA damage checkpoint	-2.291678412	-0.372	FANCD2,MUC1,E2F7
7_Member	GO:0008630	intrinsic apoptotic signaling pathway in response to DNA damage	-2.268012632	-0.356	BRCA1,CDKN2D,MUC1
7_Member	GO:1902806	regulation of cell cycle G1/S phase transition	-2.253025047	-0.343	CDKN2D,MUC1,KIF14,E2F7
7_Member	GO:0030330	DNA damage response, signal transduction by p53 class mediator	-2.222163359	-0.320	BRCA1,MUC1,E2F7
7_Member	GO:0044774	mitotic DNA integrity checkpoint	-2.210996221	-0.317	FANCD2,MUC1,E2F7
7_Member	R-HSA-3700989	Transcriptional Regulation by TP53	-2.074364393	-0.201	BRCA1,FANCD2,ELOC,CCNE2,E2F7
7_Member	GO:2001234	negative regulation of apoptotic signaling pathway	-2.067873545	-0.197	BRCA1,CDKN2D,HMGB2,MUC1
7_Member	GO:2000134	negative regulation of G1/S transition of mitotic cell cycle	-2.027591154	-0.166	CDKN2D,MUC1,E2F7
8_Summary	R-HSA-73884	Base Excision Repair	-4.780308611	-2.142	FEN1,H2BC5,POLE2,H4C3,NEIL3,POLQ
8_Member	R-HSA-73884	Base Excision Repair	-4.780308611	-2.142	FEN1,H2BC5,POLE2,H4C3,NEIL3
8_Member	ko03410	Base excision repair	-3.703592366	-1.433	FEN1,POLE2,NEIL3

8_Member	hsa03410	Base excision repair	-3.554360836	-1.338	FEN1,POLE2,NEIL3
8_Member	GO:0006284	base-excision repair	-3.45325964	-1.262	FEN1,POLQ,NEIL3
9_Summary	GO:0000910	cytokinesis	-4.53868439	-1.963	CENPA,ECT2,KIF14,CKAP2,NUSAP1,E2F7,GPSM2,CHRNA5,MLPH,BICDL1,CDCA5,BRCA1,PCLAF
9_Member	GO:0000910	cytokinesis	-4.53868439	-1.963	CENPA,ECT2,KIF14,CKAP2,NUSAP1,E2F7
9_Member	GO:0000281	mitotic cytokinesis	-3.928441321	-1.573	CENPA,ECT2,CKAP2,NUSAP1
9_Member	GO:0040001	establishment of mitotic spindle localization	-3.626714947	-1.372	CENPA,GPSM2,NUSAP1
9_Member	GO:0061640	cytoskeleton-dependent cytokinesis	-3.402483044	-1.229	CENPA,ECT2,CKAP2,NUSAP1
9_Member	GO:0051293	establishment of spindle localization	-3.301422322	-1.162	CENPA,GPSM2,NUSAP1
9_Member	GO:0051656	establishment of organelle localization	-3.257284352	-1.129	CENPA,CHRNA5,KIF14,GPSM2,NUSAP1,MLPH,BICDL1,CDCA5
9_Member	GO:0051653	spindle localization	-3.166532908	-1.068	CENPA,GPSM2,NUSAP1
9_Member	GO:0051640	organelle localization	-2.410294316	-0.467	CENPA,CHRNA5,KIF14,GPSM2,NUSAP1,MLPH,BICDL1,CDCA5
9_Member	GO:0000226	microtubule cytoskeleton organization	-2.288606114	-0.370	BRCA1,CENPA,PCLAF,KIF14,CKAP2,GPSM2,NUSAP1
10_Summary	GO:0140013	meiotic nuclear division	-4.468886212	-1.923	CALR,FANCD2,RAD21,RAD51,CCNE2,CENPS,BRCA1,RAD51AP1,CDKN2D
10_Member	GO:0140013	meiotic nuclear division	-4.468886212	-1.923	CALR,FANCD2,RAD21,RAD51,CCNE2,CENPS
10_Member	ko03460	Fanconi anemia pathway	-4.443589412	-1.917	BRCA1,FANCD2,RAD51,CENPS
10_Member	GO:0007131	reciprocal meiotic recombination	-4.41193569	-1.905	FANCD2,RAD21,RAD51,CENPS
10_Member	GO:0036297	interstrand cross-link repair	-4.41193569	-1.905	FANCD2,RAD51,RAD51AP1,CENPS
10_Member	GO:0035825	homologous recombination	-4.380889702	-1.892	FANCD2,RAD21,RAD51,CENPS
10_Member	GO:1903046	meiotic cell cycle process	-4.259345882	-1.805	CALR,FANCD2,RAD21,RAD51,CCNE2,CENPS
10_Member	GO:0007127	meiosis I	-4.242137434	-1.798	FANCD2,RAD21,RAD51,CCNE2,CENPS

10_Member	hsa03460	Fanconi anemia pathway	-4.178864891	-1.757	BRCA1,FANCD2,RAD51,CENPS
10_Member	GO:0061982	meiosis I cell cycle process	-4.157111921	-1.740	FANCD2,RAD21,RAD51,CCNE2,CENPS
10_Member	GO:0070192	chromosome organization involved in meiotic cell cycle	-3.999510787	-1.626	FANCD2,RAD21,RAD51,CCNE2
10_Member	M258	PID BARD1 PATHWAY	-3.873380605	-1.548	BRCA1,FANCD2,RAD51
10_Member	GO:0051321	meiotic cell cycle	-3.608077733	-1.366	CALR,FANCD2,RAD21,RAD51,CCNE2,CENPS
10_Member	GO:0045132	meiotic chromosome segregation	-3.522618656	-1.313	FANCD2,RAD21,CCNE2,CENPS
10_Member	M1	PID FANCONI PATHWAY	-3.245641934	-1.127	BRCA1,FANCD2,CENPS
10_Member	GO:0007129	synapsis	-3.192328512	-1.089	FANCD2,RAD21,CCNE2
10_Member	GO:0045143	homologous chromosome segregation	-2.893973798	-0.859	FANCD2,RAD21,CCNE2
10_Member	ko04110	Cell cycle	-2.046477866	-0.180	CDKN2D,RAD21,CCNE2
11_Summary	GO:0032392	DNA geometric change	-4.295035952	-1.826	HMGB2,HMGB3,RAD51,POLQ,CHD5,H3-3A,STRBP
11_Member	GO:0032392	DNA geometric change	-4.295035952	-1.826	HMGB2,HMGB3,RAD51,POLQ,CHD5
11_Member	GO:0007286	spermatid development	-2.643917732	-0.663	H3-3A,HMGB2,CHD5,STRBP
11_Member	GO:0048515	spermatid differentiation	-2.586135012	-0.618	H3-3A,HMGB2,CHD5,STRBP
11_Member	GO:0032508	DNA duplex unwinding	-2.210996221	-0.317	RAD51,POLQ,CHD5
12_Summary	GO:0006813	potassium ion transport	-3.721202401	-1.439	ATP1A4,KCNG1,KCNJ11,KCNK1,FXYD3, KCNG3,CHRNA5,CNTNAP2,SLC7A8,SLC39A6,EDN2,ECT2,RAD51
12_Member	GO:0006813	potassium ion transport	-3.721202401	-1.439	ATP1A4,KCNG1,KCNJ11,KCNK1,FXYD3, KCNG3
12_Member	R-HSA-1296071	Potassium Channels	-3.353759797	-1.195	KCNG1,KCNJ11,KCNK1,KCNG3
12_Member	GO:0035094	response to nicotine	-3.301422322	-1.162	CHRNA5,KCNJ11,KCNK1
12_Member	GO:0035637	multicellular organismal signaling	-3.127038108	-1.031	ATP1A4,KCNJ11,KCNK1,FXYD3,CNTNAP2
12_Member	GO:0071804	cellular potassium ion transport	-3.02635301	-0.957	ATP1A4,KCNG1,KCNJ11,KCNK1,KCNG3

12_Member	GO:0071805	potassium ion transmembrane transport	-3.02635301	-0.957	ATP1A4,KCNG1,KCNJ11,KCNK1,KCNG3
12_Member	R-HSA-5578775	Ion homeostasis	-3.022451736	-0.957	ATP1A4,KCNJ11,FXYD3
12_Member	R-HSA-5576891	Cardiac conduction	-2.867124108	-0.841	ATP1A4,KCNJ11,KCNK1,FXYD3
12_Member	GO:0098739	import across plasma membrane	-2.788767921	-0.771	ATP1A4,KCNJ11,SLC7A8,SLC39A6
12_Member	GO:0061337	cardiac conduction	-2.756457498	-0.752	ATP1A4,KCNJ11,KCNK1,FXYD3
12_Member	GO:0008016	regulation of heart contraction	-2.711290164	-0.717	ATP1A4,EDN2,KCNJ11,KCNK1,FXYD3
12_Member	GO:1903779	regulation of cardiac conduction	-2.689743835	-0.701	ATP1A4,KCNJ11,FXYD3
12_Member	GO:0060047	heart contraction	-2.517567292	-0.559	ATP1A4,EDN2,KCNJ11,KCNK1,FXYD3
12_Member	GO:0003015	heart process	-2.453757893	-0.502	ATP1A4,EDN2,KCNJ11,KCNK1,FXYD3
12_Member	GO:0098659	inorganic cation import across plasma membrane	-2.404941352	-0.465	ATP1A4,KCNJ11,SLC39A6
12_Member	GO:0099587	inorganic ion import across plasma membrane	-2.404941352	-0.465	ATP1A4,KCNJ11,SLC39A6
12_Member	GO:1903522	regulation of blood circulation	-2.398491888	-0.462	ATP1A4,EDN2,KCNJ11,KCNK1,FXYD3
12_Member	R-HSA-397014	Muscle contraction	-2.245673849	-0.337	ATP1A4,KCNJ11,KCNK1,FXYD3
12_Member	GO:0009636	response to toxic substance	-2.03094133	-0.167	CHRNA5,ECT2,KCNJ11,KCNK1,RAD51,SLC7A8
13_Summary	ko04960	Aldosterone-regulated sodium reabsorption	-3.554360836	-1.338	ATP1A4,HSD11B2,PIK3R2
13_Member	ko04960	Aldosterone-regulated sodium reabsorption	-3.554360836	-1.338	ATP1A4,HSD11B2,PIK3R2
13_Member	hsa04960	Aldosterone-regulated sodium reabsorption	-3.486039818	-1.289	ATP1A4,HSD11B2,PIK3R2
14_Summary	GO:0033574	response to testosterone	-3.421337316	-1.239	CALR,KCNJ11,PSPH,GJB2,H3-3A,HSD11B2,BRCA1,HMGB2
14_Member	GO:0033574	response to testosterone	-3.421337316	-1.239	CALR,KCNJ11,PSPH
14_Member	GO:0007565	female pregnancy	-2.328885459	-0.400	CALR,GJB2,H3-3A,HSD11B2
14_Member	GO:1901654	response to ketone	-2.298016871	-0.377	CALR,GJB2,KCNJ11,PSPH
14_Member	GO:0048545	response to steroid hormone	-2.154052456	-0.266	BRCA1,CALR,GJB2,HMGB2,HSD11B2

14_Member	GO:0044706	multi-multicellular organism process	-2.113369608	-0.232	CALR,GJB2,H3-3A,HSD11B2
15_Summary	GO:1903827	regulation of cellular protein localization	-3.22079678	-1.106	ECT2,GPC4,KCNJ11,PIK3R2,CCNE2,LYPLA1,GPSM2,CDCA5,PACSIN1
15_Member	GO:1903827	regulation of cellular protein localization	-3.22079678	-1.106	ECT2,GPC4,KCNJ11,PIK3R2,CCNE2,LYPLA1,GPSM2,CDCA5
15_Member	GO:1904375	regulation of protein localization to cell periphery	-3.063947212	-0.983	GPC4,KCNJ11,LYPLA1,GPSM2
15_Member	GO:1990778	protein localization to cell periphery	-2.227561106	-0.322	GPC4,KCNJ11,LYPLA1,GPSM2,PACSIN1
16_Summary	GO:0000731	DNA synthesis involved in DNA repair	-3.092319533	-1.006	CDKN2D,POLE2,PCLAF,BRCA1,POLQ
16_Member	GO:0000731	DNA synthesis involved in DNA repair	-3.092319533	-1.006	CDKN2D,POLE2,PCLAF
16_Member	GO:0006301	postreplication repair	-3.092319533	-1.006	BRCA1,POLE2,PCLAF
16_Member	GO:0071897	DNA biosynthetic process	-2.298016871	-0.377	CDKN2D,POLE2,PCLAF,POLQ
17_Summary	GO:0051259	protein complex oligomerization	-2.914578662	-0.875	ECT2,KCNG1,RRM2,POLQ,KCNG3
17_Member	GO:0051259	protein complex oligomerization	-2.914578662	-0.875	ECT2,KCNG1,RRM2,POLQ,KCNG3
17_Member	GO:0051260	protein homooligomerization	-2.47733683	-0.524	ECT2,KCNG1,POLQ,KCNG3
18_Summary	GO:0016266	O-glycan processing	-2.914438808	-0.875	MUC1,ST3GAL4,GALNT6,PLA2G10,LYPLA1
18_Member	GO:0016266	O-glycan processing	-2.914438808	-0.875	MUC1,ST3GAL4,GALNT6
18_Member	R-HSA-913709	O-linked glycosylation of mucins	-2.873859011	-0.846	MUC1,ST3GAL4,GALNT6
18_Member	hsa00564	Glycerophospholipid metabolism	-2.268012632	-0.356	MUC1,PLA2G10,LYPLA1
18_Member	GO:0006493	protein O-linked glycosylation	-2.210996221	-0.317	MUC1,ST3GAL4,GALNT6
18_Member	R-HSA-5173105	O-linked glycosylation	-2.178168436	-0.287	MUC1,ST3GAL4,GALNT6

19_Summary	GO:0006820	anion transport	-2.722658148	-0.727	CA12,FXYD3,PLA2G10,SLC16A6,SLC7A8,CLIC6,SFXN1,GPR89A,KCNG1,KCNJ11,KCNG3
19_Member	GO:0006820	anion transport	-2.722658148	-0.727	CA12,FXYD3,PLA2G10,SLC16A6,SLC7A8,CLIC6,SFXN1,GPR89A
19_Member	GO:0034765	regulation of ion transmembrane transport	-2.166962393	-0.277	KCNG1,KCNJ11,FXYD3,CLIC6,KCNG3,GPR89A
20_Summary	GO:0032465	regulation of cytokinesis	-2.431757921	-0.483	ECT2,KIF14,E2F7,PIK3R2,GPSM2,DEPDC1B,IQGAP3
20_Member	GO:0032465	regulation of cytokinesis	-2.431757921	-0.483	ECT2,KIF14,E2F7
20_Member	GO:0007264	small GTPase mediated signal transduction	-2.081560782	-0.207	ECT2,PIK3R2,KIF14,GPSM2,DEPDC1B,IQGAP3
